# Continuous Flow Acylation of (Hetero)aryllithiums with Polyfunctional *N*,*N*‐Dimethylamides and Tetramethylurea in Toluene

**DOI:** 10.1002/chem.202102805

**Published:** 2021-09-09

**Authors:** Dimitrije Djukanovic, Benjamin Heinz, Francesca Mandrelli, Serena Mostarda, Paolo Filipponi, Benjamin Martin, Paul Knochel

**Affiliations:** ^1^ Department Chemie Ludwig-Maximilians-Universität München Butenandtstraße 5–13, Haus F 81377 Munich Germany; ^2^ Novartis Pharma AG, Chemical Development Fabrikstraße 4002 Basel Switzerland

**Keywords:** amide, acylation, continuous flow, lithium, toluene

## Abstract

The continuous flow reaction of various aryl or heteroaryl bromides in toluene in the presence of THF (1.0 equiv) with *sec*‐BuLi (1.1 equiv) provided at 25 °C within 40 sec the corresponding aryllithiums which were acylated with various functionalized *N*,*N*‐dimethylamides including easily enolizable amides at −20 °C within 27 sec, producing highly functionalized ketones in 48–90 % yield (36 examples). This method was well suited for the preparation of α‐chiral ketones such as naproxene and ibuprofen derived ketones with 99 % *ee*. A one‐pot stepwise bis‐addition of two different lithium organometallics to 1,1,3,3‐tetramethyurea (TMU) provided unsymmetrical ketones in 69–79 % yield (9 examples).

## Graphical abstract

***N,N*** **‐Dimethylamides for continuous flow acylations**: A new acylation of (hetero)aryllithiums with polyfunctional and enolizable *N,N*‐dimethylamides in toluene in continuous flow was reported. The required lithium species was prepared at 25 °C from (hetero)aryl bromides and *sec*‐BuLi in toluene. Additionally, a new telescoped procedure, using tetramethylurea as C1‐building block, was described providing highly functionalized unsymmetrical ketones.

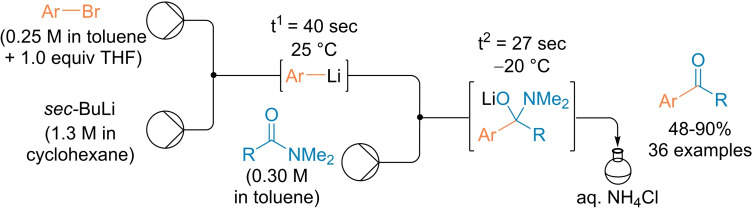

The acylation of organometallics with carbonyl derivatives represents an excellent preparation of ketones which are of high interest in medicinal, agrochemical and material chemistry.^[1]^ Although acid chlorides were often used as acylation reagents,^[1]^ alternative carboxyl derivatives such as 2‐thiopyridyl esters,^[2]^ Weinreb amides,^[3]^ 2‐pyridylamides,^[4]^ morpholino‐amides,^[5]^ *N*‐acylpyrroles^[6]^ or *N*,*N*‐dimethylamides^[7]^ have been used successfully in combination with appropriate organometallics^[8]^ or transition metal catalysts.^[9]^

The performance of organometallic reactions in continuous flow has recently given a novel dimension to a range of these synthetic methods.^[10]^ The accurate control of residence times, temperatures and concentrations greatly improved many reactions involving organometallic intermediates.^[11]^ Thus, Nagaki and Yoshida have recently reported the synthesis of functionalized ketones from acid chlorides and lithium reagents by extremely fast micro‐mixing.^[12]^ Although functionalized ketones were prepared, this method required the use of water sensitive acid chlorides as well as extremely fast mixing not accessible on commercial flow apparatus.^[12]^ The use of ecologically and industrially friendly halide free acylation reagents would be highly desirable. Hattan and Jamison have described double additions to carbon dioxide for the preparation of various ketones (Scheme 1a).^[13]^ Kappe has used mixed anhydrides for a continuous flow synthesis of α‐haloketones.^[14]^ The continuous flow mode has also allowed a convenient use of esters as acylating agents.^[15]^

**Scheme 1:**
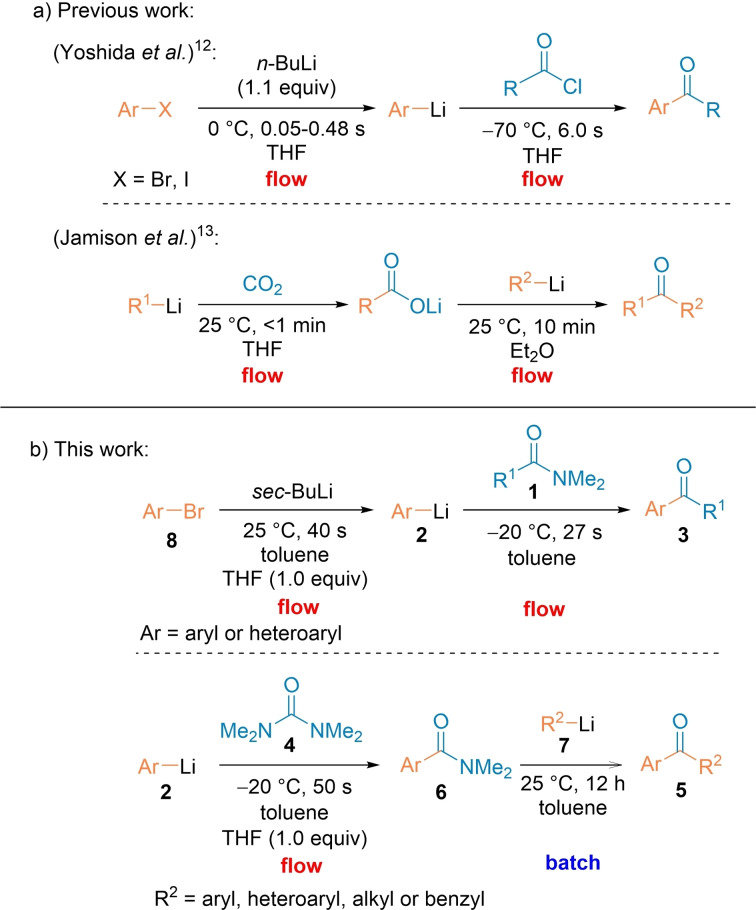
a) Previous work on acylations in continuous flow starting from acyl chlorides and CO_2_. b) Acylations of aryl and heteroaryllithiums of type **2** prepared via Br/Li‐exchange in continuous flow with *N,N*‐dimethylamides of type **1** affording ketones of type **3** and selective stepwise double acylation with 1,1,3,3‐tetramethylurea (**4**) leading to ketones of type **5**.

Herein, we report the use of readily available and convenient *N*,*N*‐dimethylamides of type **1**[^16^, ^17^] as convenient and effective reagents for the acylation of various (hetero)aryllithiums of type **2** ^[17]^ in toluene using a continuous flow set‐up leading to various functionalized ketones of type **3** including halogenomethyl ketones and α‐chiral ketones (Scheme 1b).

We have shown that TMU (1,1,3,3‐tetramethylurea, **4**) allows an efficient and selective synthesis of new unsymmetrical ketones of type **5** via in situ generated arylated *N*,*N*‐dimethylamides **6** and batch‐prepared R^2^‐Li species of type **7** (Scheme 1b).

Thus, in preliminary experiments, we have optimized the preparation of aryllithiums of type **2**. In order to achieve a fast exchange with a stable aryllithium intermediate of type **2**, we have explored the metal‐exchange and electrophilic quench of 1‐bromo‐4‐methylthiobenzene (**8** **a**)^[17]^ in both THF and toluene at ambient temperatures. Therefore, we treated **8** **a** with *sec*‐BuLi (1.1 equiv) in THF or toluene. We found that the Br/Li‐exchange was fast in THF leading to the aryllithium **2** **a**, but that this lithium organometallic was not stable at 25 °C as shown by quenching experiments with 4‐fluorobenzaldehyde (**9**), leading to the expected alcohol **10** in only 24–27 % yield; (Table 1, entries 1–2). Switching to the common solvent toluene[^18^, ^19^, ^20^] afforded the aryllithium species **2** **a** in better yields, but the Br/Li‐exchange reaction was too sluggish and required up to 2 h reaction time for completion (Table 1, entries 3–5). In balance, we found that simply adding 1.0 equiv. of THF to the toluene solution of **8** **a** led to a fast Br/Li‐exchange within 1 min at 25 °C and produced, after quenching with **9**, the alcohol **10** in 95 % calibrated GC‐yield (Table 1, entry 6).

**Table 1:** Optimization of the aryllithium generation in batch and flow.

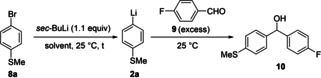					
entry	set‐up	solvent	time [min]	Conversion of **8** **a** [GC‐%]	Formation of **10** [GC‐%]
1	batch	THF	1	90	24
2	batch	THF	30	93	27
3	batch	toluene	1	18	8
4	batch	toluene	30	75	49
5	batch	toluene	120	94	57
6	batch	toluene^[a]^	1	96	95
7	batch	toluene^[a]^	10	98	85
8	batch	toluene^[a]^	30	>99	60
9	flow	toluene^[a]^	1	>99	99

[a] 1.0 equiv. of THF was added which corresponded to a ca. 50 : 1 toluene:THF mixture.

In contrast, using *n*‐BuLi led to a slower Br/Li exchange of **8** **a** incompatible with the stability of the generated metal species. Longer storage time of **2** **a** at 25 °C (10‐30 min) afforded lower yields of **10** showing the instability of **2** **a** over time (Table 1, entries 7 and 8). In counterpoint, performing this reaction at this temperature in flow led to a quantitative formation of **10**, showing that a flow set‐up using toluene in the presence of 1.0 equiv. of THF was most advantageous (entry 9). The low stability of aryllithiums at ambient temeratures justified this “on‐demand” preparation in continuous flow and enabled potential scale‐ups. In preliminary reactions, we observed that proton‐quenching via amide enolization in THF led to proto‐desbrominated products (thioanisole). The present solvent system (toluene containing 1.0 equiv. of THF) also reduced this enolization side‐reactions on amides bearing acidic protons.[^21^, ^22^]

By optimizing the concentration of **8** **a** and *sec*‐BuLi, the residence times for the Br/Li‐exchange as well as the acylation temperature, a high GC‐yield of the ketone **3** **aa** was achieved. Thus, performing the acylation reaction in continuous flow at either 25 °C or 0 °C led only to 50–67 % of the ketone **3** **aa** (Table 2, entries 1 and 2). However, lowering the reaction temperature to −20 °C or −40 °C gave satisfactory yields (82‐84 %; entries 3 and 4).

**Table 2:** Optimization of the acylation temperature continuous flow.

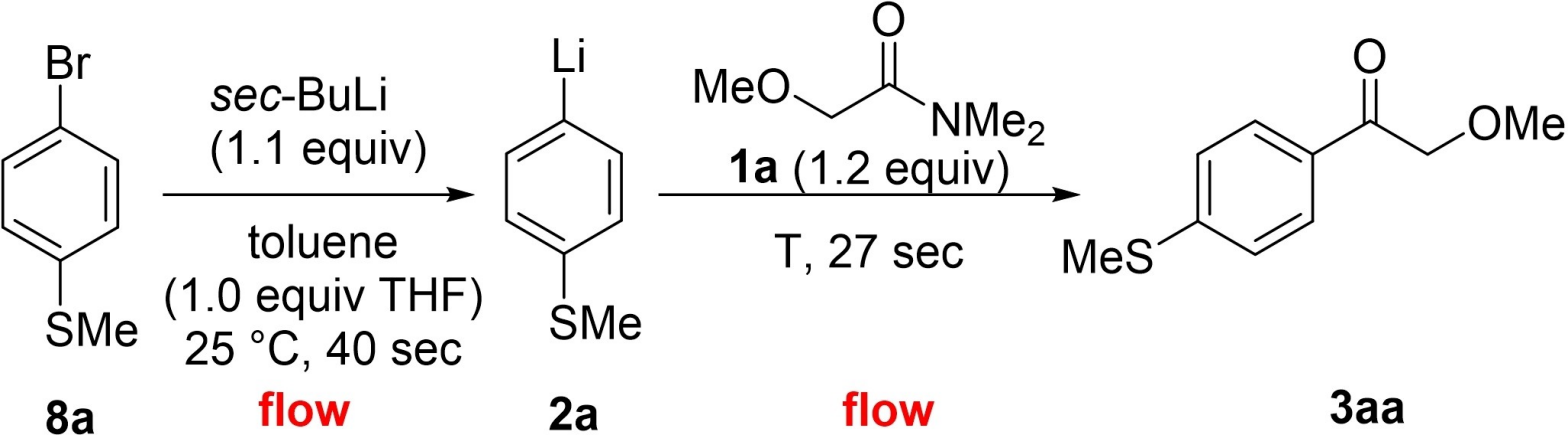			
entry	T [°C]	conversion of **8** **a** [GC‐%]	product formation **3** **aa** [GC‐%]
1	25	>99	50
2	0	>99	67
3	−20	>99	82
4	−40	>99	84

With these conditions in hand, using the aryl bromide **8** **a** (0.25 M in toluene containing 1.0 equiv. of THF) with a flow rate of 5.0 mL/min and *sec*‐BuLi (1.1 equiv, 1.35 M in *n*‐hexane) with a flow rate of 1.1 mL/min, we have quantitatively generated the corresponding aryllithium **2** **a** at 25 °C (t^1^= 40 sec). After precooling the lithium species for 10 sec, the acylation step was performed at −20 °C (t^2^ = 27 sec) affording, via the formation of the tetrahedral intermediate **11** and subsequent quenching with *sat. aq*. NH_4_Cl, the desired ketone **3** **aa** in 82 % isolated yield. A scale‐up of this reaction in continuous flow was easily achieved by simply prolonging the collecting time (from 0.5 min to 6.5 min) and led to a comparable yield (78 %, Scheme 2).

**Scheme 2:**
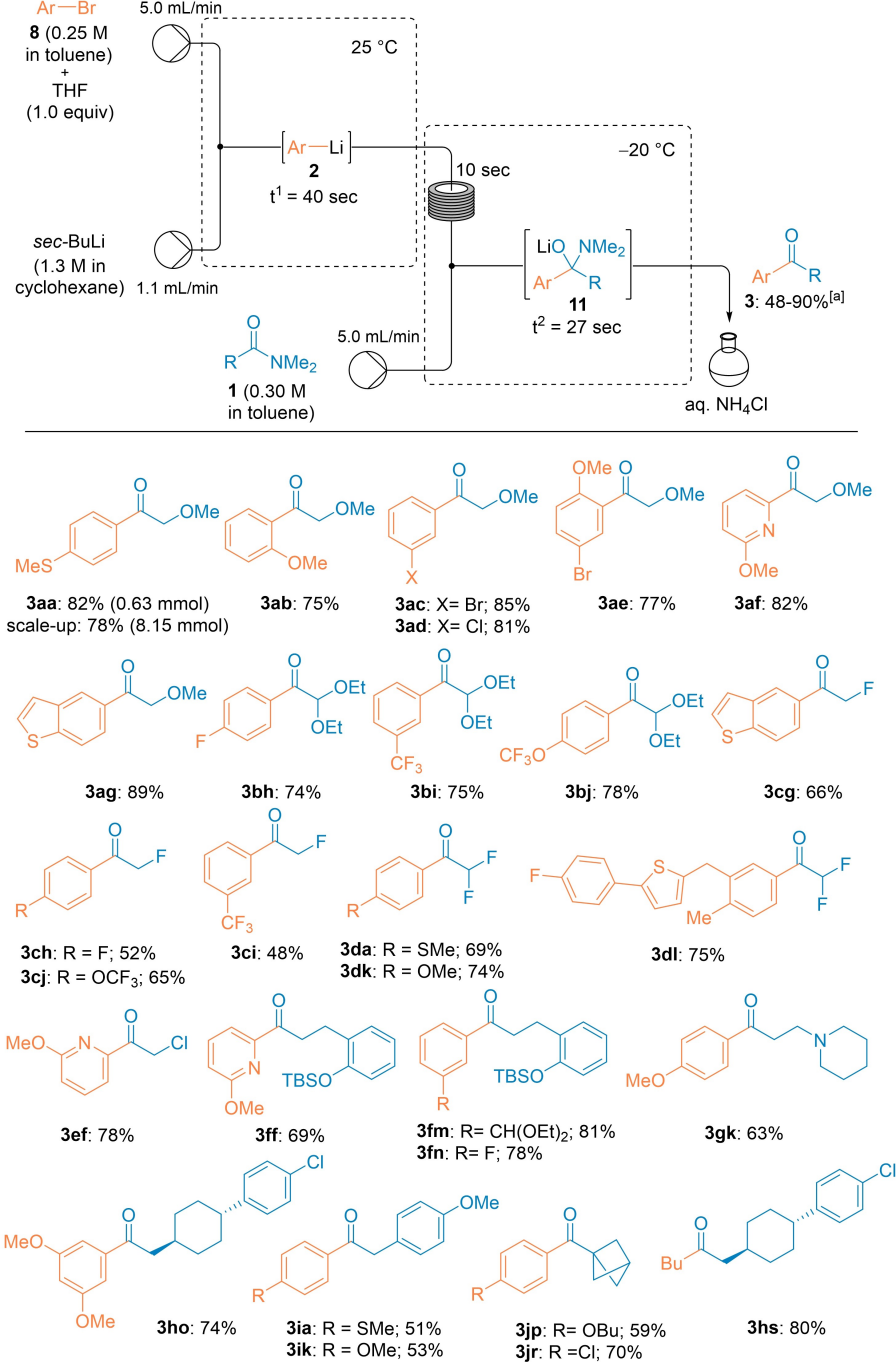
A continuous flow acylation of various amides **1** with in situ generated lithium organometallics **2** leading to polyfunctional ketones **3**. [a] The indicated yields refer to yields of isolated products.

Various aryllithiums (**2** **b‐e**) bearing MeO, Br or Cl as substituents were quantitatively prepared by Br/Li‐exchange from the corresponding aryl bromides and their acylation with **1** **a** afforded the expected ketones **3** **ab‐ae** in 75–85 % isolated yield. Also, heterocyclic lithium species were generated in this way and the acylation with **1** **a** produced the heterocyclic ketones **3** **af** and **3** **ag** in 82–89 % yield. A related functionalized amide such as 2,2‐diethoxy‐*N,N*‐dimethylacetamide (**1** **b**) behaved in the same way providing, after the reaction with fluoro‐substituted aryllithiums, the ketones **3** **bh**–**bj** in 74–78 % yield. Also, various α‐monofluoro‐, difluoro‐ or monochloro‐substituted amides **1** **c**, **1** **d** and **1** **e** gave the expected ketones despite the presence of readily enolizable protons at the α‐position to the amide group. The use of the non‐polar solvent toluene significantly reduced such enolization side‐reactions as mentioned above.^[21]^ Thus, the α‐halogenated ketones **3** **cg**–**cj**, **3** **da**–**dl** and **3** **ef** were obtained in 48–78 % yield. *N,N*‐Dimethylamides such as **1** **f**, **1** **g** and **1** **h**, bearing remote oxygen‐ or nitrogen‐containing functional groups, provided aromatic and heterocyclic ketones **3** **ff**–**fn**, **3** **gk** and **3** **ho** in 63–81 % isolated yield. As a limitation, we have found that *N,N*‐dimethyl‐phenylacetamide (**1** **i**) gave in this procedure only average yields of the desired aryl benzyl ketones **3** **ia** and **3** **ik** due to competitive enolization and consequent proto‐debromination of the starting material (ca. 25 % of enolization was noticed in the present solvent system, whereas over 70 % enolization was found in pure THF). [1,1,1]‐bicyclopentane carboxamide **1** **j** was also a suitable substrate and the reaction with various lithiums of type **2** furnished the bicyclopent‐1‐yl ketones **3** **jp** and **3** **jr** in 59–70 % isolated yield.^[23]^ Finally, the dialkyl ketone **3** **hs** was prepared by directly using *n*‐BuLi as organolithium species via a 2‐pump system (Scheme 2).

Next, we turned our attention to the preparation of highly functionalized benzophenone derivatives and heterocyclic ketones (Scheme 3). Thus, the cyano group in *N,N*‐dimethyl‐4‐cyanobenzamide (**6** **a**)^[17]^ was well tolerated leading to the cyano‐substituted benzophenones **12** **ae‐an** in 61–79 % isolated yield. Remarkably, by using *N,N*‐dimethyl‐4‐iodobenzamide (**6** **b**), no competitive I/Li‐exchange was observed and the desired iodo‐substituted benzophenones **12** **bq** and **12** **br** were obtained in 63–79 % yield. Also, commercially available *N,N*‐diethylnicotinamide (**6** **c**) provided the heterocyclic ketone **12** **cr** in 58 % yield after the usual sequence in continuous flow.

**Scheme 3:**
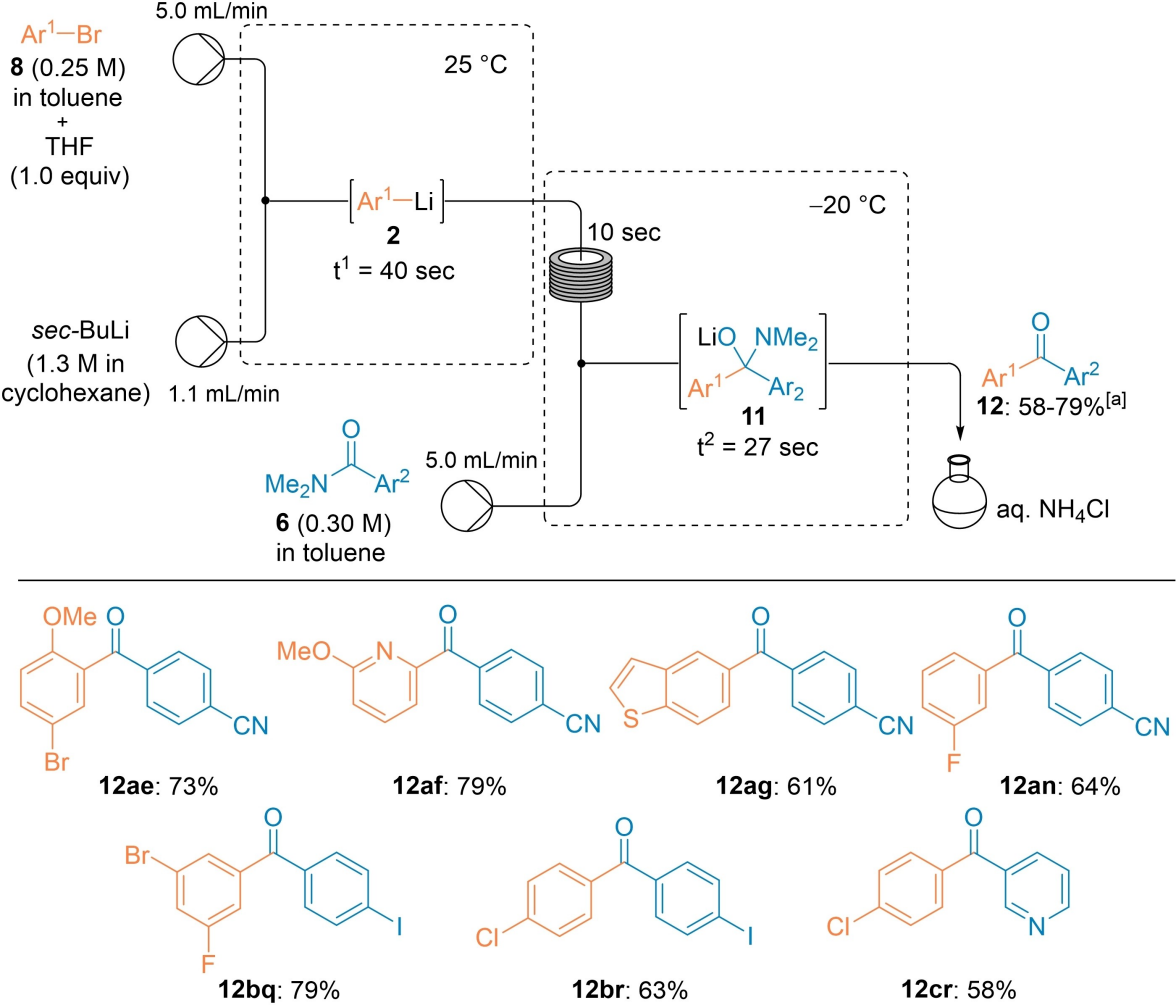
Preparation of functionalized benzophenones and heterocyclic ketones in continuous flow by acylation of (hetero)aryllithiums of type **2** with ArCONMe_2_ **6**. [a] The indicated yields refer to yields of isolated products.

The preparation of racemizable α‐chiral ketones was readily achieved with this new acylation procedure (Scheme 4). This is demonstrated in the case of naproxen and ibuprofen derived α‐chiral ketones. Those analogues of non‐steroidal anti‐inflammatory drugs (NSAIDs) were of interest in the pursuit of antivirals^[24]^ and to tackle gastrointestinal side‐effects such as ulceration.^[25]^ Thus, the readily available chiral *N,N*‐dimethylamide of naproxen **13** **a** (99 % *ee*) was treated under standard continuous flow conditions with various functionalized aryllithiums of type **2** leading to the desired chiral ketones **14** **ac‐an** in 65–88 % yield with complete retention of chirality (99 % *ee*).^[26]^ Also, the chiral *N,N*‐dimethylamide of ibuprofen **13** **b** (99 % *ee*) was acylated with (hetero)aryllithiums to give the chiral ketones **14** **bh**–**bs** in 75–89 % isolated yield (98‐99 % *ee*).

**Scheme 4:**
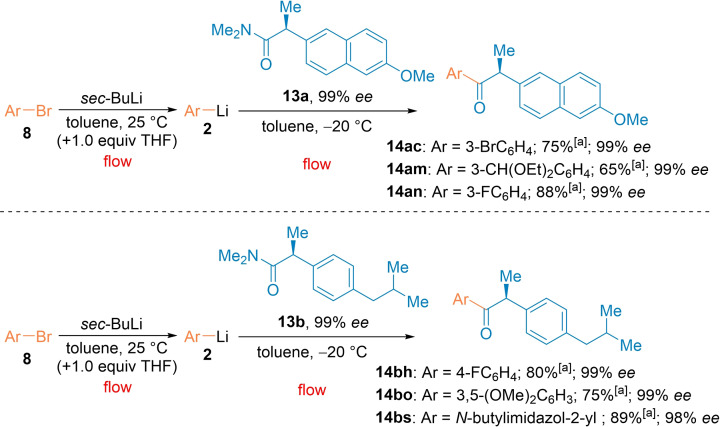
Preparation of chiral naproxene or ibuprofen ketone derivatives of type **14** by the reaction of aryllithiums **2** with naproxene or ibuprofen derived *N*,*N*‐dimethylamides **13** in continuous flow. [a] The indicated yields refer to yields of isolated products.

Finally, we have extended this acylation in continuous flow to a semi‐batch telescoped procedure for the preparation of unsymmetrical ketones of type **5** using TMU (**4**) as a C1‐building block (Scheme 5).[^8a^, ^27^] Thus, the treatment of a mixture of ArBr (**8**) and TMU (**4**) in toluene with *sec*‐BuLi at −20 °C for 50 sec in continuous flow provided the tetrahedral intermediate **15** which was poured into a toluene solution of various organolithiums R−Li (**7**, R = Bu, (Het)Ar or Bn). These organolithiums were conveniently prepared via direct metalation, using *sec*‐BuLi and TMEDA (1.0 equiv) in toluene at −20 °C (10‐30 min) in batch. Presumably, due to a high stability of the intermediate **15**, the second addition was quite slow and took up to 12 h at 25 °C. After aqueous workup, the corresponding ketones **5** **a**–**5** **f** were obtained in 69–79 % yield. Remarkably, no additional equivalent of THF was needed to ensure a fast Br/Li‐exchange, showing that TMU played a similar activator role as THF for the fast formation of the lithium species.^[28]^

**Scheme 5:**
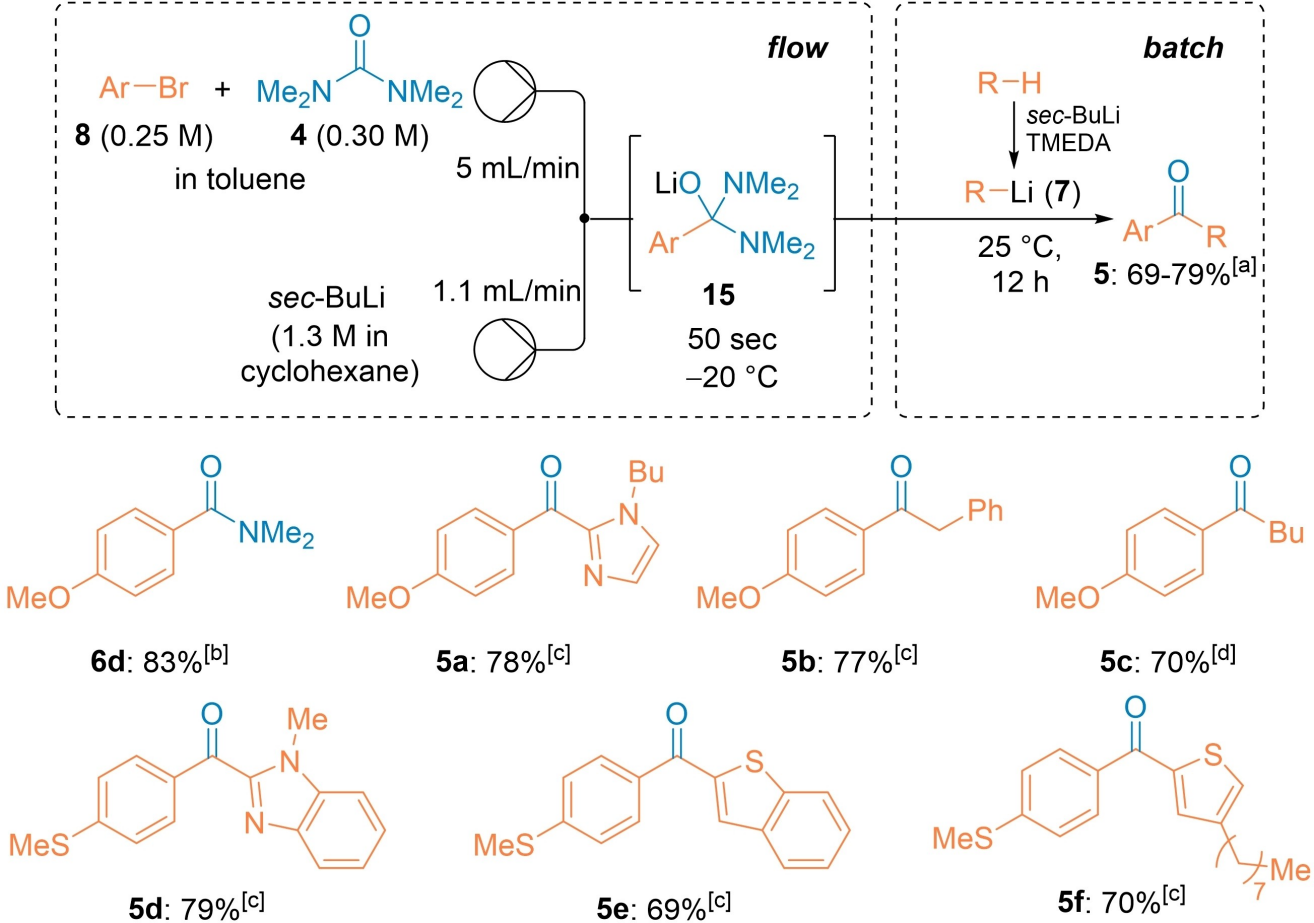
Semi‐batch telescoped preparation of unsymmetrical ketones of type **5** by two successive acylations of TMU with various lithium organometallics. [a] The indicated yields refer to yields of isolated products. [b] The reaction mixture was quenched with *sat. aq*. NH_4_Cl solution. [c] R−Li was prepared via direct metalation with *sec*‐BuLi (1.2 equiv) in batch at −20 °C in toluene in the presence of 1 equiv. of TMEDA (10–30 min). [d] The reaction mixture was poured into *n*‐BuLi (1.5 equiv) and stirred for 12 h at 25 °C.

In summary, we have reported a new convenient acylation of organolithiums **2** with various enolizable and functionalized *N*,*N*‐dimethylamides **1** in continuous flow at −20 °C. The required aryllithiums (**2**) were also prepared in continuous flow at 25 °C using a Br/Li‐exchange mediated by *sec*‐BuLi with toluene as solvent in the presence of 1.0 equiv. of THF. This acylation was scalable without further optimization and was found to be suitable for the preparation for a broad range of polyfunctional ketones, including α‐chiral ketones of type **14** with excellent enantioselectivities. Furthermore, this method was extended to a semi‐batch telescoped preparation of unsymmetrical ketones using TMU (**4**) as C1‐building block. Compared to previous acylation procedures, readily prepared and stable *N,N*‐dimethylamides^[16]^ of moderate toxicity, tolerating many functionalities, were used. The solvent toluene in the presence of 2 vol % THF minimized enolization side reactions and allowed ambient reaction temperatures. Further applications are underway.

## Conflict of interest

The authors declare no conflict of interest.

## Supporting information

As a service to our authors and readers, this journal provides supporting information supplied by the authors. Such materials are peer reviewed and may be re‐organized for online delivery, but are not copy‐edited or typeset. Technical support issues arising from supporting information (other than missing files) should be addressed to the authors.

Supporting Information
