# Supplementary material for: Continuous Flow Acylation of (Hetero)aryllithiums with Polyfunctional N,N‐Dimethylamides and Tetramethylurea in Toluene
Source: Chemistry. 2021 Sep 9;27(56):13977–81. doi: 10.1002/chem.202102805 (PMC8519161; doi:10.1002/chem.202102805)

Chemistry–A European Journal

Supporting Information

Continuous Flow Acylation of (Hetero)aryllithiums with Polyfunctional *N*,*N*-Dimethylamides and Tetramethylurea in Toluene

Dimitrije Djukanovic[+], Benjamin Heinz[+], Francesca Mandrelli, Serena Mostarda, Paolo Filipponi, Benjamin Martin, and Paul Knochel*

Table of Content

General Information SI-2

List of bromides of type **8** and the corresponding aryllithiums of type **2**............................SI-5

List of amides of type **1**, **6** and **13**…………………………………………………………......SI-6

Optimizations and Screenings…………………………………………………………………SI-7

Typical Procedures…………………………………………………………………...….SI-18

Preparation of Products…………………………………………………………………SI-21

Chiral HPLC Analysis………………………………………………………………...….SI-70

NMR Data………………………………………………...……………..…………SI-86

General Information

All reactions were carried out under argon or nitrogen atmosphere in glassware dried with a heat gun (650 °C) under high vacuum (<1 mbar). Syringes which were used to transfer anhydrous solvents or reagents were purged thrice with argon or nitrogen prior to use. Indicated yields are isolated yields of compounds estimated to be >95% pure as determined by ^{1}H-NMR (25 °C) and capillary GC analyses. Unless otherwise indicated, all reagents were obtained from commercial sources.

Solvents

Solvents were dried according to standard procedures by distillation over drying agents and stored under argon.

THF was continuously refluxed and freshly distilled from sodium benzophenone ketyl under nitrogen.

***n*-Hexane** was continuously refluxed and freshly distilled from sodium benzophenone ketyl under nitrogen.

Toluene was purchased from Arcros Organics (anhydrous, 99.85%)

Solvents for column chromatography were distilled on a rotary evaporator prior to use.

Content determination of organometallic reagents

***sec*-BuLi** was titrated with *iso*propanol and 1,10-phenanthroline as indicator in THF.[1]

Chromatography

Flash column chromatography was performed using silica gel 60 (0.040-0.063 mm) from MERCK.

Thin layer chromatography was performed using SiO_2 pre-coated aluminum plates (Merck 60, F-254). The chromatograms were examined under 254 nm UV irradiation, by incubating the plates in an iodine chamber and/or by staining the TLC plate with a $KMnO_4$ solution followed by heating with a heat gun.

---

[1] a) H.-S. Lin, L. A. Paquette, *Synth. Commun.* **1994**, *24*, 2503-2506; b) S. C. Watson, J. F. Eastham, *J. Organomet. Chem.* **1967**, *9*, 165-168.

$KMnO_4$ solution: $KMnO_4$ (3.0 g), 5 drops of conc. H_2SO_4 in water (300mL).

Flow reactions

Flow reactions were carried out with solutions of the reactants in dry solvents (toluene, THF, *n*-hexane). Flame-dried glassware was used for the reagent solutions and kept under an argon atmosphere during the reactions. Undecane or tetradecane were used as internal standards. For all flow reactions a Vapourtec E-series Integrated Flow Chemistry System with 3rd Pump Kit, Organometallic Kit and Collection Valve Kit was used. Reactions were performed in coiled tube reactors. Coiled reactors (1.0, 2.0, 4.0, 5.0, 10.0 or 20.0 mL) were made from PFA or PTFE Teflon (i.d. = 0.8 mm, o.d. = 1.6 mm or i.d = 1.6 mm, o.d. = 3.2 mm). Prior to performing reactions, the system was dried by flushing it with MeOH, followed by toluene (blue tubing) or *n*-hexane (red tubing) (flow rate of all pumps: 1.00 mL·min-1; run-time: 30 min).

Analytical Data

^{1}H-NMR and **^{13}C-NMR** spectra were recorded on VARIAN Mercury 200, BRUKER ARX 300, VARIAN VXR 400 S and BRUKER AMX 600 instruments. Chemical shifts are reported as values in ppm relative to tetramethylsilane. $CDCl_3$ peaks were set to 7.26 ppm in ^{1}H NMR and 77.16 ppm in ^{13}C NMR experiments. The following abbreviations were used to characterize signal multiplicities: s (singlet), d (doublet), dd (doublet of doublets), t (triplet), q (quartet), hept (heptett) as well as m (multiplet).

Mass spectroscopy: High resolution (HRMS) and low resolution (MS) spectra were recorded on a FINNIGAN MAT 95Q instrument. Electron impact ionization (EI) was conducted with an ionization energy of 70 eV. For coupled gas chromatography/mass spectrometry, a HEWLETT-PACKARD HP 6890/MSD 5973 GC/MS system was used. Molecular fragments are reported starting at a relative intensity of 10-30%.

Infrared spectra (IR) were recorded from 4500 cm^{-1} to 650 cm^{-1} on a PERKIN ELMER Spectrum BX-59343 instrument. For detection a SMITHS DETECTION DuraSampl*IR* II Diamond ATR sensor was used. The main absorption peaks are reported in cm^{-1}.

Melting points (m.p.) were determined on a BÜCHI B-540 melting point apparatus and are uncorrected.

Enantiomeric excess (*ee*) of the compounds were measured by *Shimazu* HPLC Prominence with *Daicel* Chiralcelor using chiral GC. Optical rotation values were recorded on

a *PerkinElmer* 241 or *Anton Paar* MCP 200 polarimeter. The specific rotation is calculated as follows:

$$[\propto]_D^{20} = \frac{[\propto] \cdot 100}{c \cdot d}$$

Thereby, the wavelength λ is reported in nm and the measuring temperature φin °C. α represents the recorded optical rotation, *c* the concentration of the analyte in 10 mg/mL and d the length of the cuvette in dm. Thus, the specific rotation is given in $10^{-1}\cdot deg\cdot cm^2\cdot g^{-1}$. Usage of the sodium D line (λ = 589 nm) is indicated by D instead of the wavelength in nm. The respective concentration as well as the solvent is reported at the relevant section of the experimental section.

List of bromides of type 8 and the corresponding aryllithiums of type 2

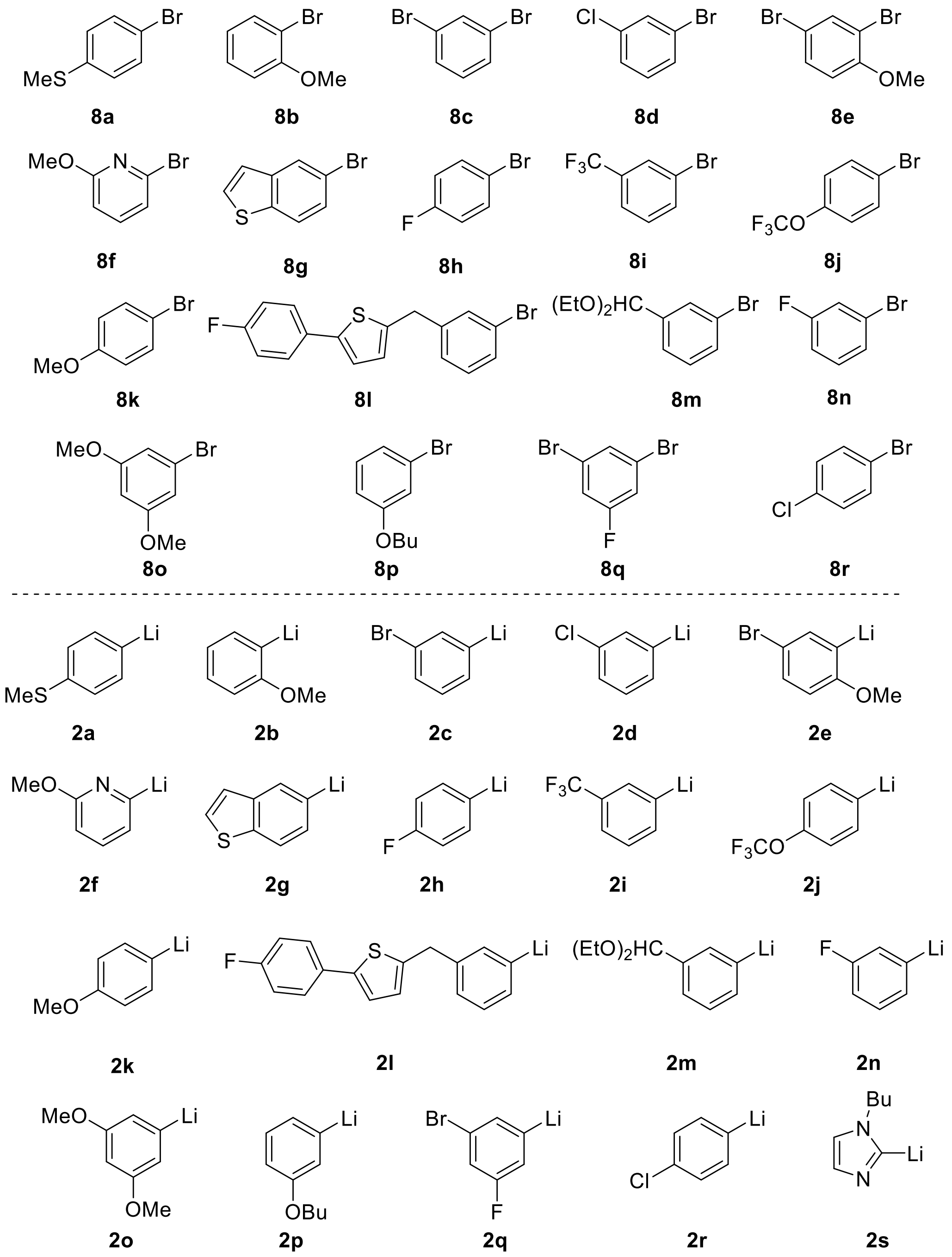

Figure 1 List of bromides of type **8** and the corresponding aryllithiums of type **2**.

List of amides of type 1, 6 and 13

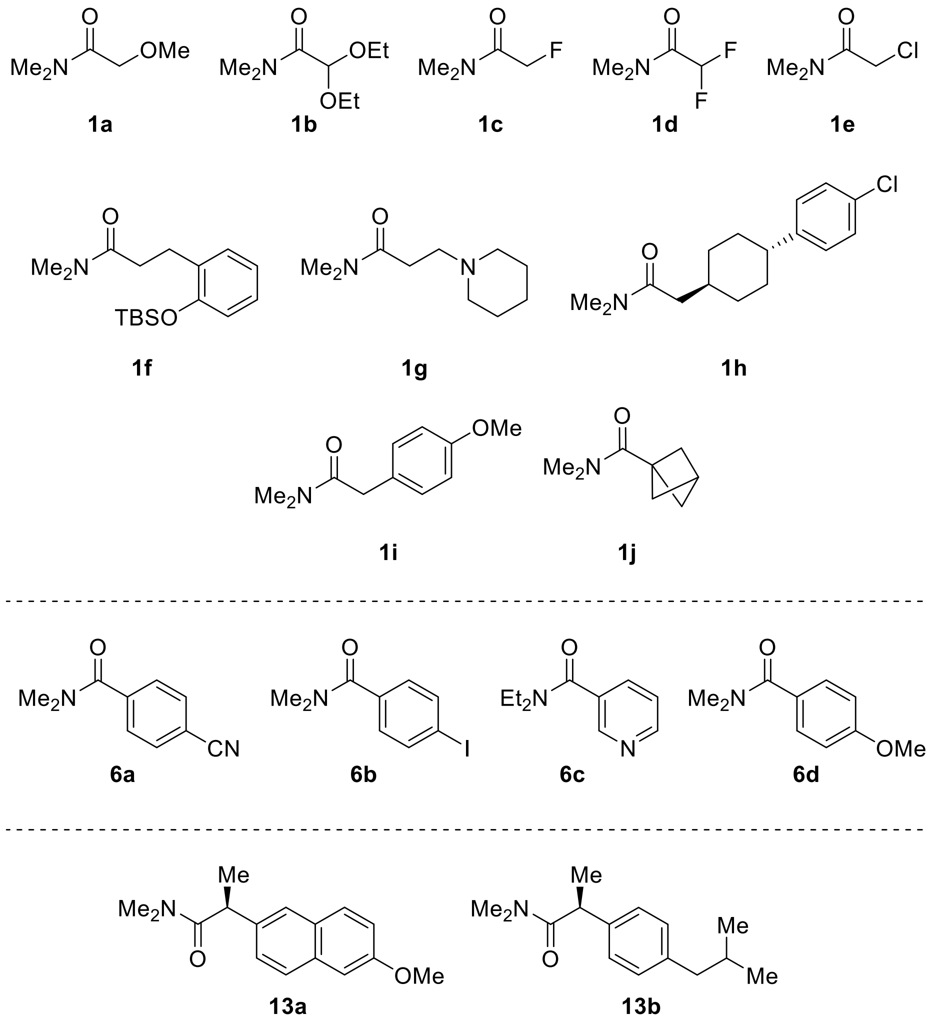

Figure 2 List of amides of type **1**, **6** and **13**

Optimizations and Screenings

A) Development of Barbier-type reactions of aryl bromides with tetramethylurea (TMU) using *sec*-BuLi in continuous flow

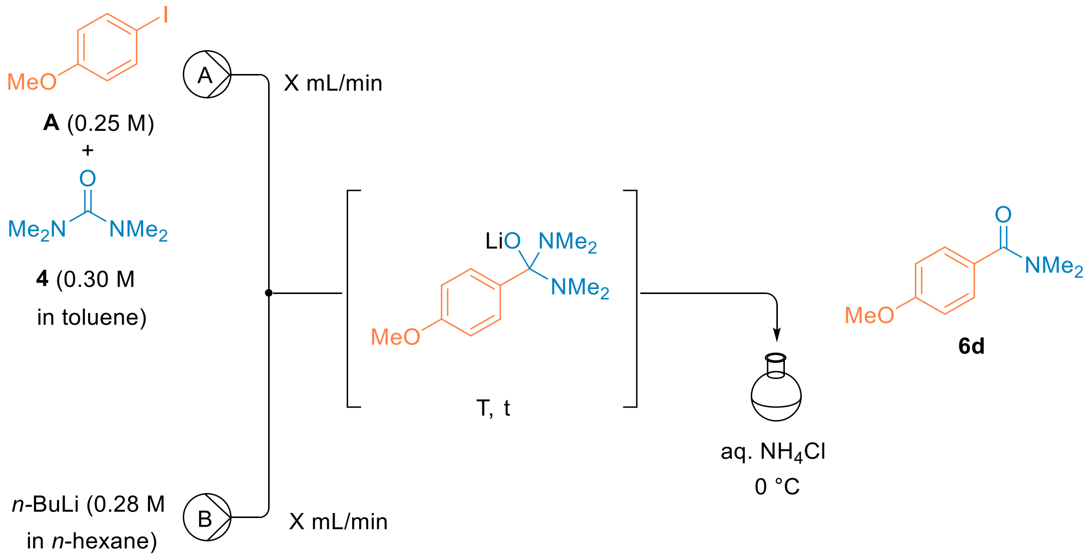

Scheme 1 Screening for Barbier-type acylations in continuous flow starting from aryl iodide **A** in toluene, tetramethylurea **4** and *n*-BuLi.

Table 1

entry	T [°C]	t [sec]	Flowrate A+B [X mL/min]	Leftover of **A** [GC-%]	Product **6d** [GC-%]
1	0	60	5+5	2	82
2	0	300	1+1	11	69
3	0	60	1+1	9	73
4	0	12	5+5	1	82
5	10	12	5+5	<1	83
6	–10	12	5+5	<1	80
7	–20	12	5+5	<1	79
8	–30	12	5+5	<1	75

- Isolated yield for entry 5: **83%**
- We have started our study by noticing the positive effect of tetramethylurea on the solubility and on its rate enhancing role for the I/Li-exchange reaction.

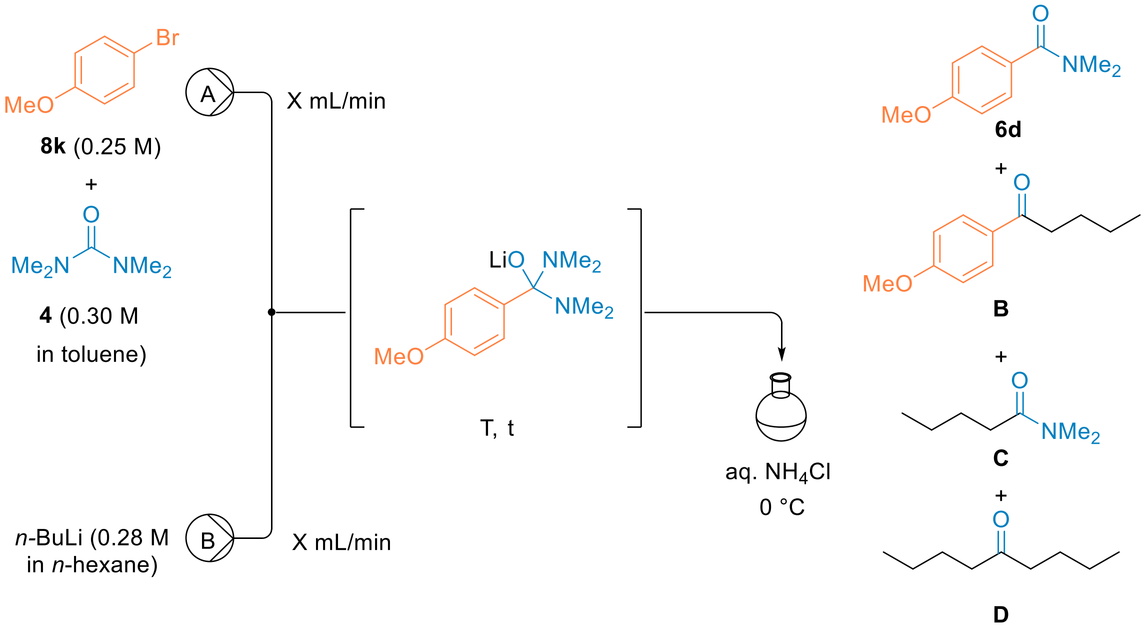

Scheme 2 Screening for Barbier-type acylations in continuous flow starting from aryl bromide **8k** in toluene, tetramethylurea **4** and *n*-BuLi.

Table 2

entry	T [°C]	t [sec]	Flowrate A+B [X mL/min]	Leftover **8k** [GC-%]	Product **6d** [GC-%]	BuLi-Addition **B** [GC-%]
1	22	12	5+5	66	17	11
2	0	12	5+5	61	22	14
3	−20	12	5+5	52	43	8
4	−40	12	5+5	36	54	7
5	−54	12	5+5	32	42	6
6	−20	60	5+5	54	43	7

- Aryl bromides proved to be more challenging than aryl iodides. Side-products of addition of *n*-BuLi to the urea have been observed due to a slow exchange.
- Lower temperatures helped to control all side-reactions, but longer reaction time did not lead to a complete reaction conversion.

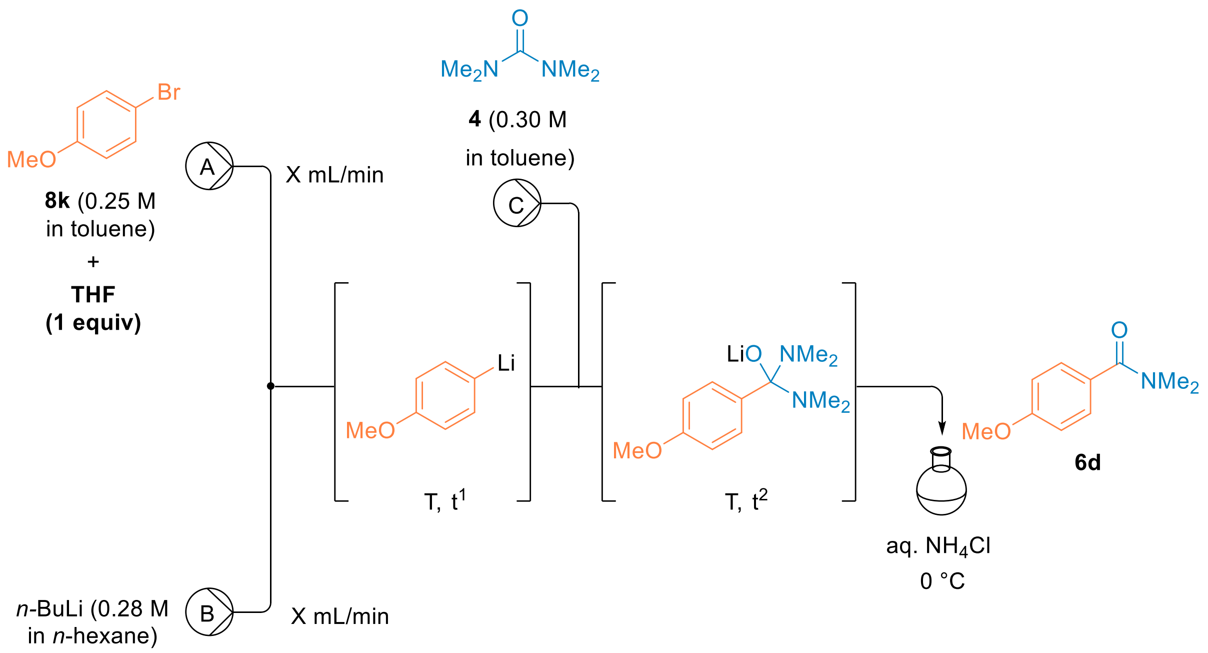

Scheme 3 Screening for stepwise acylations in continuous flow starting from aryl bromide **8k** in toluene with 1.0 equiv of THF, *n*-BuLi and tetramethylurea **4**.

Table 3

entry	T [°C]	t^1 [sec]	t^2 [sec]	Flowrate A+B+C [X mL/min]	Leftover **8k** [GC-%]
1	–10	60	20	5+5+5	57
2	40	60	20	5+5+5	56
3	50	300	100	1+1+1	25
4*	60	300	100	1+1+1	17

- A stepwise reaction, using a 3-pump system, with THF (1.0 equiv) as additive was examined but the Br/Li-exchange on the challenging *p*-bromoanisole was slow even with elevated temperatures.

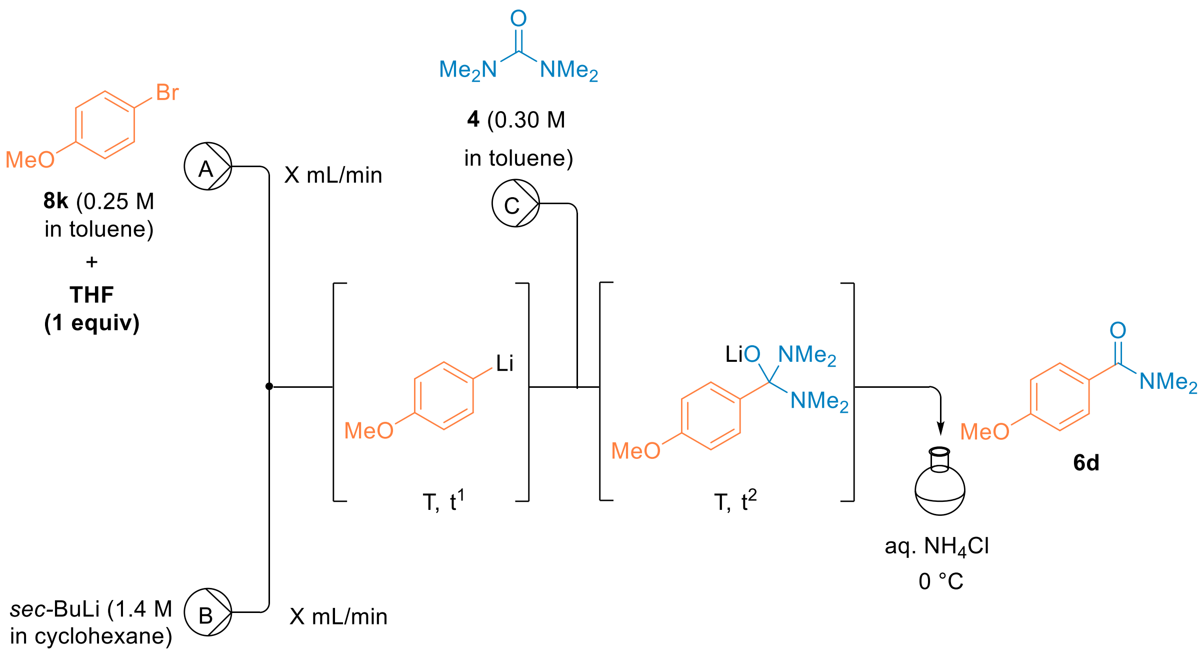

Scheme 4 Screening for stepwise acylations in continuous flow starting from aryl bromide **8k** in toluene with 1.0 equiv of THF, *sec*-BuLi and tetramethylurea **4**.

Table 4

entry	T [°C]	t^1 [sec]	t^2 [sec]	Flowrate A+B+C [X mL/min]	Leftover **8k** [GC-%]	Product **6d** [GC-%]
1	25	50	27	5+1+5	8	75
2	25	49	100	8+1.6+8	5	77
3	25	49	27	5+1.1+5.1	3	79
4	25	49	27	5+1.2+5.2	1	82

- Isolated yield for entry 4: **81%**
- The more reactive *sec*-BuLi exchange reagent enabled a clean Br/Li-exchange within less than 1 min residence time at 25 °C.

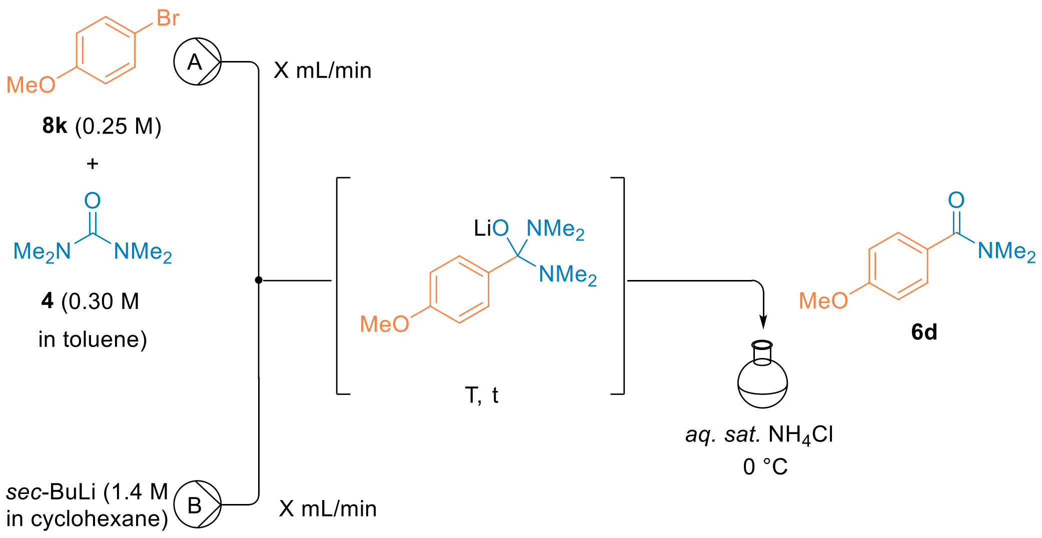

Scheme 5 Screening for Barbier-type acylations in continuous flow starting from aryl bromide **8k** in toluene, tetramethylurea **4** and *sec*-BuLi.

Table 5

Entry	T [°C]	t [sec]	Flow rate A+B [mL/min]	Leftover **8k** [GC-%]	Product **6d** [GC-%]
1	25	50	5+1	25	41
2	25	25	10+2	22	52
3	0	25	10+2	13	73
4	−10	25	10+2	7	83
5	−20	25	10+2	4	83
6	−30	25	10+2	1	82

- Isolated yield for entry 5: **83%**
- Finally, we discovered that a Barbier-type reaction with *sec*-BuLi led to high conversion and high yield of **6d**, showing that tetramethylurea is playing a similar activation role as THF.

B) Development of stepwise reactions of aryl bromides with *N,N*-dimethylamides using *sec*-BuLi in continuous flow

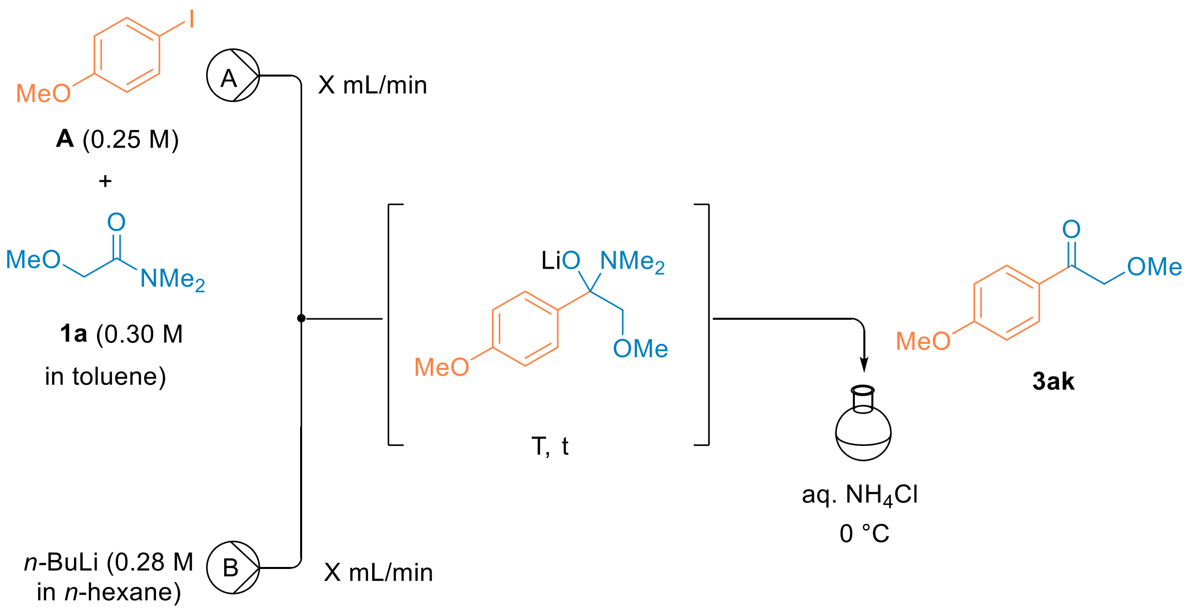

Scheme 6 Screening for Barbier-type acylations in continuous flow starting from aryl iodide **A** in toluene, *N,N*-dimethylamide **1a** and *n*-BuLi.

Table 6

Entry	T [°C]	t [sec]	Flow rate A+B [mL/min]	Leftover **A** [GC-%]	Product **3ak** [GC-%]
1	–20	50	5+5	1	65
2	–30	50	5+5	1	74
3	–40	50	5+5	1	73
4	–50	50	5+5	1	72

- Isolated yield for entry 2: **76%**
- Reactions using Barbier-type conditions with aryl iodide as substrate were optimised. However, low temperatures (–30 °C) were necessary to ensure a good reaction outcome.

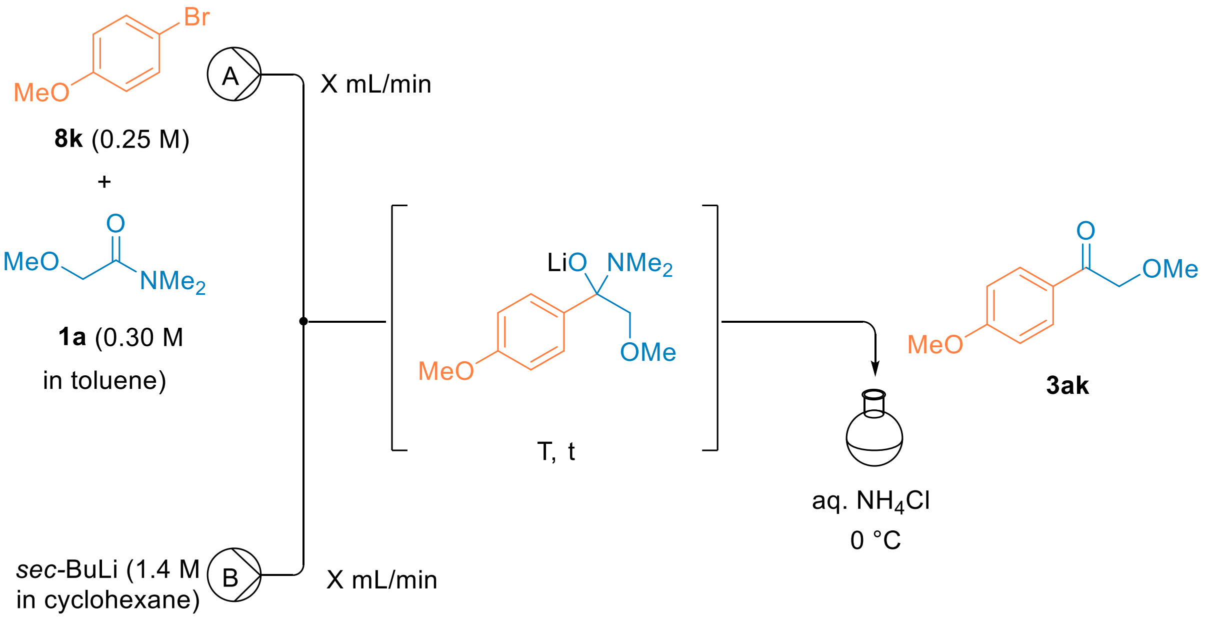

Scheme 7 Screening for Barbier-type acylations in continuous flow starting from aryl bromide **8k** in toluene, *N,N*-dimethylamide **1a** and *sec*-BuLi.

Table 7

Entry	T [°C]	t [sec]	Flow rate A+B [mL/min]	Leftover **8k** [GC-%]	Product **3ak** [GC-%]
1	0	50	5+5	40	23
2	−20	50	5+5	36	34
3	−40	50	5+5	clogging	

- Barbier-type conditions with electron rich aryl bromides failed to deliver a product in good yield even when *sec*-BuLi was used as exchange reagent.
- Conclusion: Amides are less good promotors of the Br/Li-exchange compared to TMU, leading to incomplete conversion and side-reactions.

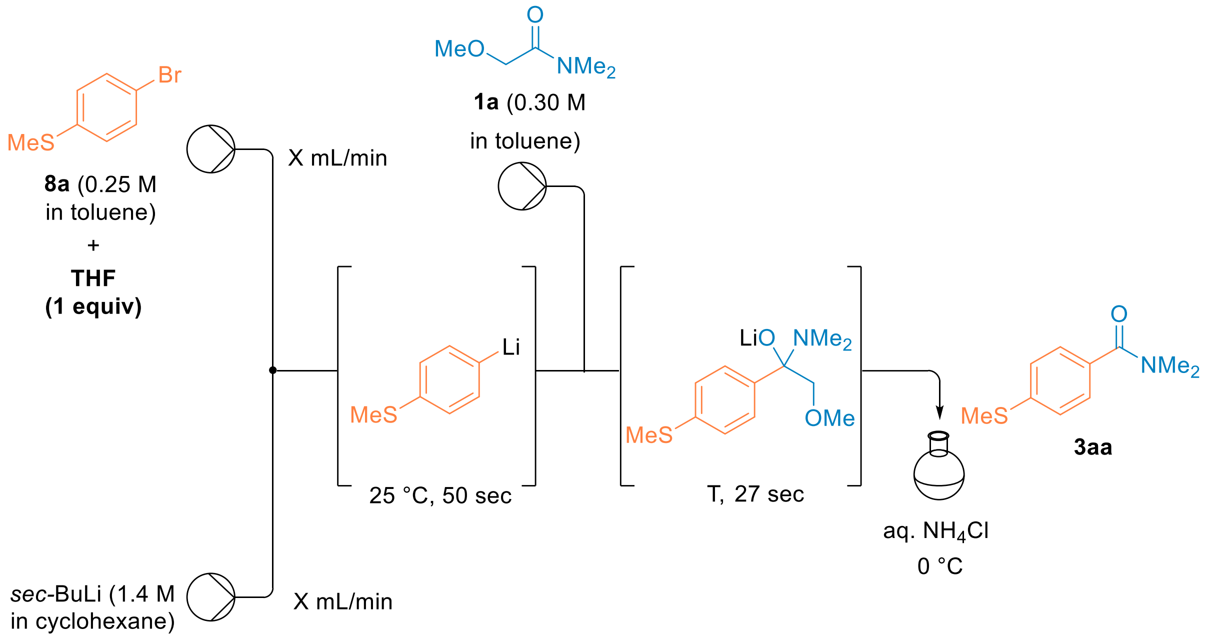

Scheme 8 Screening for stepwise acylations in continuous flow starting from aryl bromide **8a** in toluene with 1.0 equiv of THF, *sec*-BuLi and *N,N*-dimethylamide **1a**.

Table 8

Entry	T [°C]	Leftover **8a** [GC-%]	Product **3aa** [GC-%]
1	25	1	50
2	0	1	67
3	-20	1	82
4	-40	1	84

- Isolated yield for entry 3: **82%**
- A stepwise approach, preparing the Li-species first at 25 °C to ensure a complete exchange, led to a high conversion of **8a** and high yields of **3aa**.

C) Batch screening for Br/Li-exchange in toluene and THF

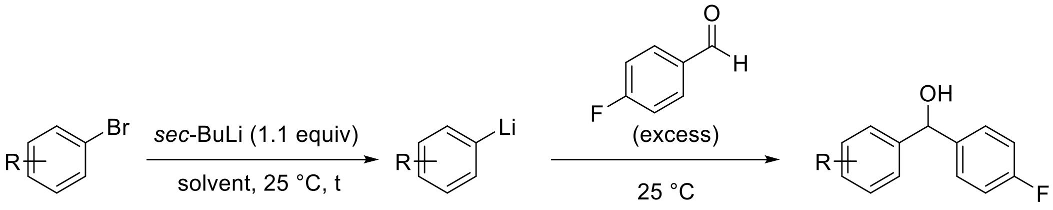

8 E

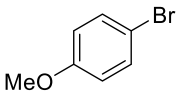

entry	set-up	solvent	time [min]	conversion of **8k** [GC-%]	Product **E** [GC-%]
1	batch	THF	10	91	40
1	batch	THF	60	95	9
1	batch	toluene*	10	99	81
1	batch	toluene*	60	99	61
1	flow	toluene*	1	99	99

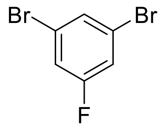

entry	set-up	solvent	time [min]	conversion of **8q** [GC-%]	Product **E** [GC-%]
1	batch	THF	10	93	-
1	batch	THF	60	94	-
1	batch	toluene*	10	99	5
1	batch	toluene*	60	99	-
1	flow	toluene*	1	99	98

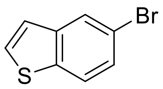

entry	set-up	solvent	time [min]	conversion of **8g** [GC-%]	Product **E** [GC-%]
1	batch	THF	10	95	10
1	batch	THF	60	94	8
1	batch	toluene*	10	99	25
1	batch	toluene*	60	99	23
1	flow	toluene*	1	99	98

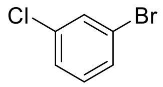

entry	set-up	solvent	time [min]	conversion of **8d** [GC-%]	Product **E** [GC-%]
1	batch	THF	10	91	-
1	batch	THF	60	93	-
1	batch	toluene*	10	99	85
1	batch	toluene*	60	99	76
1	flow	toluene*	1	99	99

* with 1.0 equiv of THF

Scheme 9 Br/Li-exchange screening for various aryl bromides of type **8** with *sec*-BuLi at 25 °C in THF or toluene with 1.0 equiv of THF.

D) Screening for the effect of toluene or THF on the enolization reaction during the addition of ArLi to amides

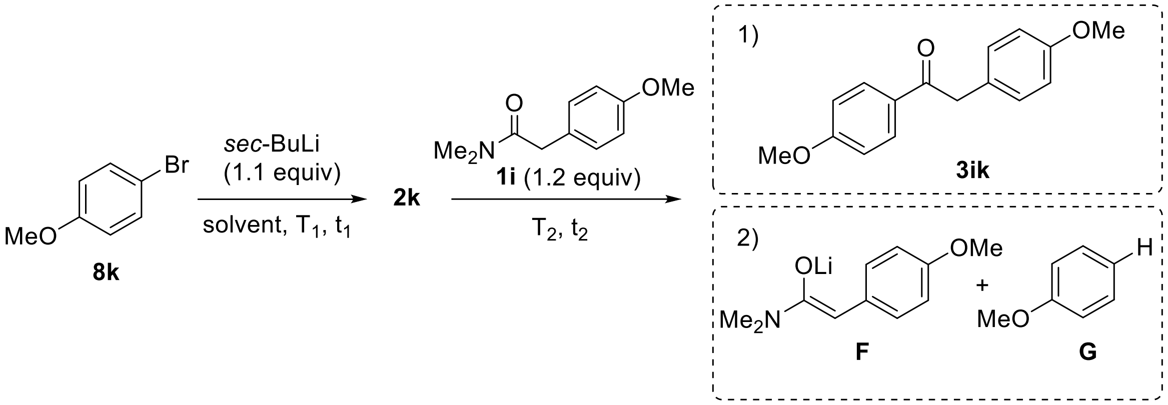

entry	set-up	solvent	t_1 [min]	T_1 [°C]	t_2 [min]	T_2 [°C]	conversion of **8k** [GC-%]	Ratio **3ik/G** [GC-%]	Product **3ik** [GC-%]	Hydrolysis **G** [GC-%]
1	batch	THF	1	25	5	-20	80	0.8/1	33	40
2	batch	THF	5	-20	5	-20	88	0.9/1[a]	35	38
3	batch	THF	30	-78	30	-78	>99	1.6/1	45	28
4	batch	toluene[b]	1	25	5	-20	97	3.3/1	59	18
5	flow	toluene[b]	0.67	25	0.5	0	>99	3.0/1	53	18
6	flow	toluene[b]	0.67	25	0.5	-20	>99	3.4/1	60	17
7	flow	toluene[b]	0.67	25	0.5	-40	>99	3.0/1	59	20

Scheme 10: Screening for the effect of toluene or THF on the enolization reaction during the addition of ArLi **2k** to the amide **1i**. [a] Reaction was quenched with benzaldehyde instead of *sat. aq.* NH_4Cl to proove that **G** is formed during the reaction and not when quenched. [b] 1.0 Equiv of THF was added.

E) Comparison experiments for different amides and esters for the acylation of the aryllithium 8a

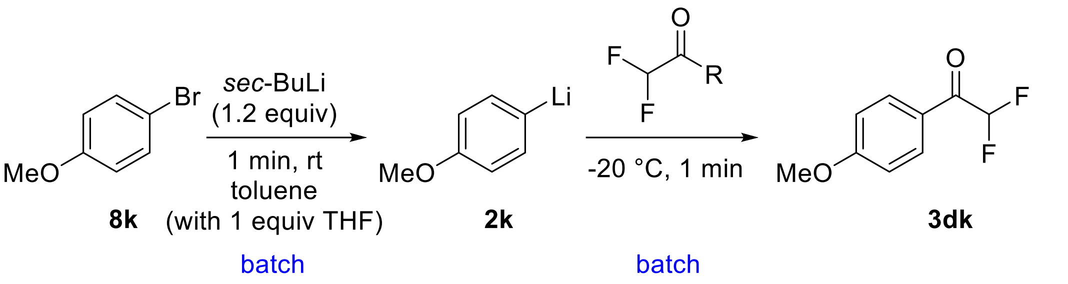

entry	R	conversion of **8k** [GC-%]	product formation **3dk** [GC-%]	double addition [GC-%]
1	NMe_2	>99	79	-
2	NEt_2	>99	78	-
3	Morpholine	>99	81	-
4	OEt	97	43	14

Scheme 11: Comparison experiments for different amides and esters for the acylation of the aryllithium **8a**.

Typical Procedures

Typical Procedure 1A: Preparation of *N,N*-dimethylamides of type **1, 6** or **13** starting from the corresponding methyl or ethyl carboxylates.

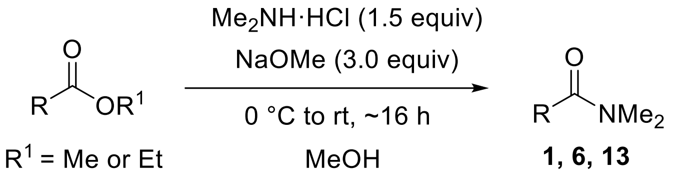

To a 1 M solution of ethyl or methyl ester in MeOH was added $Me_2NH{\cdot}HCl$ (1.5 equiv). Then, 30% NaOMe in MeOH (3.0 equiv) was added at 0 °C while stirring. After full conversion to the corresponding *N,N*-dimethylamide (checked *via* GC or TCL analysis), the reaction mixture was quenched with *sat. aq.* NH_4Cl. Methanol was removed under *vacuo* (300 mbar) and water was added under stirring until a clear solution was obtained. After extraction with the indicated solvent (depending on the volatility of the compound), the combined organic layers were dried with $MgSO_4$ and evaporated to give the crude dimethylamide. The crude compounds were either directly used or purified *via* distillation under reduced pressure or column chromatography.

Typical Procedure 1B: Preparation of *N,N*-dimethylamides of type **6** and **13** starting from the corresponding carboxylic acids.

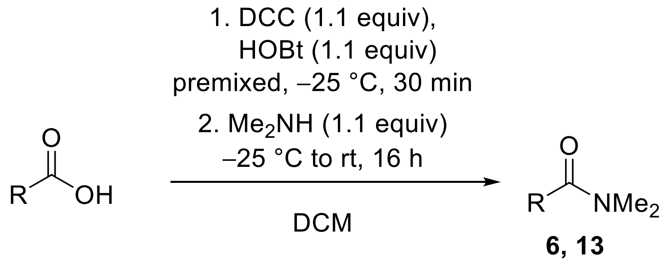

To DCC (dicyclohexylcarbodiimide, 1.1 equiv), dissolved in 60 ml of dry DCM, was added HOBt (hydroxybenzotriazole, 1.1 equiv) in one portion at 25 °C. After 20-30 min, a clear solution was obtained and cooled to –25 °C. In a separate flask, carboxylic acid (0.5 M, 1.1 equiv) was dissolved or suspended in dry DCM and cooled to –25 °C. The solution of DCC/HOBt was cannulated over 15 min into the solution of carboxylic acid. After stirring for

30 min at –25 °C, Me_2NH (2 M in THF, 1.1 equiv) was added dropwise. The suspension was allowed to warm to 25 °C and stirred for 16 h. The reaction mixture was filtered over sinter to remove *N,N'*-dicyclohexylurea and the DCM layer was washed with 10% $Na_2CO_{3(aq)}$ (3 x 30 mL). The combined aqueous layers were washed with DCM. After drying, filtrating and concentrating the organic layers, flash column purification gave pure *N,N*-dimethylamide.

Typical Procedure 2: A continuous flow acylation of various amides **1**, **6** or **13** with *in situ* generated aryllithiums **2** leading to polyfunctional ketones of type **3**, **12** and **14**.

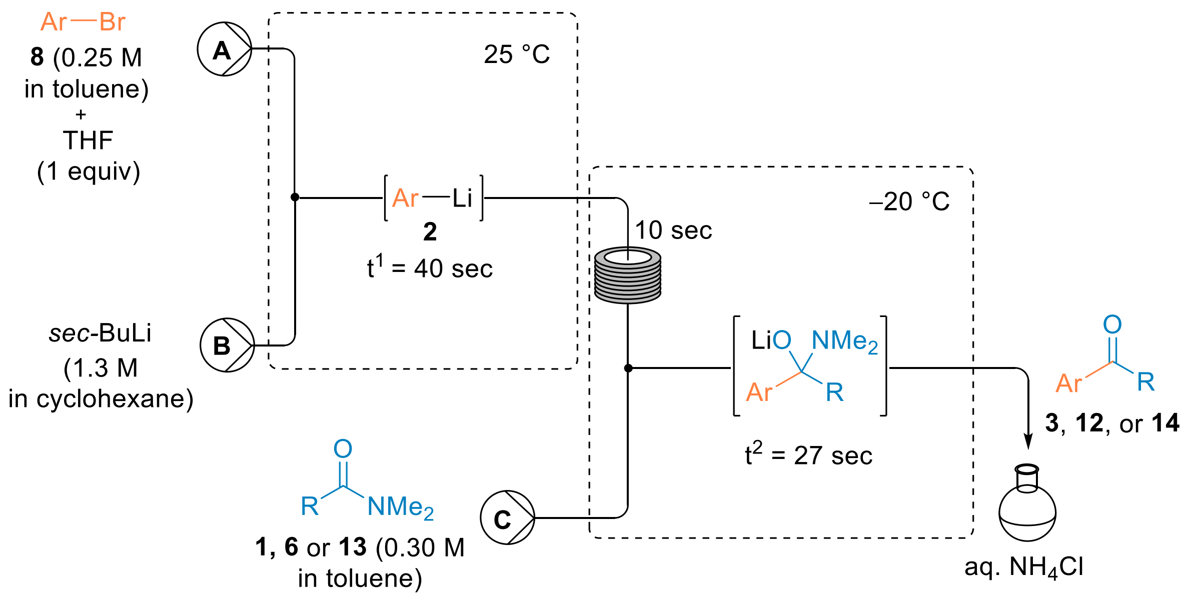

A solution of **8** (0.25 M, 1.0 equiv) and THF (1.0 equiv) in toluene and a solution of *sec*-BuLi in cyclohexane (1.3 M, 1.2 equiv) were prepared. The solutions were pumped from their flasks through a suction needle at flowrate A = 5.0 $mL·min^{-1}$ and flowrate B = 1.15 $mL·min^{-1}$. The solutions were mixed in a T-mixer (PFA or PTFE, I.D. = 0.5 mm) and the combined stream passed a PTFE reactor tube (i.d = 0.8 mm, Vol_{R1} = 4 mL; residence time: t = 40 sec, T = 25 °C), followed by a PTFE reactor tube (i.d = 0.8 mm, Vol_{R1} = 1 mL; residence time: t = 10 sec, T = –20 °C) for precooling the reaction mixture. A *N,N*-dimethylamide solution (0.3 M, 1.2 equiv) in toluene was added *via* a third pump (flowrate C = 5.0 $mL·min^{-1}$, i.d = 0.8 mm Vol_{pre} = 2.0 mL, T_{pre} = –20 °C, residence $time_{pre:}$ t = 24 sec). The combined stream passed a

PTFE reactors tube (i.d = 1.6 mm, Vol_{R2} = 5 mL; residence time: t = 27 sec, T = –20 °C) and the reaction mixture was subsequently quenched with *sat. aq.* NH_4Cl at 0 °C. After extraction with EtOAc or DCM, the combined organic phases were dried over Na_2SO_4 and filtrated. After removal of the solvent *in vacuo*, flash column chromatography purification with *n*-pentane:EtOAc mixtures afforded the pure products of type **3**, **12** and **14**.

Typical Procedure 3: One-pot preparation of unsymmetrical ketones of type **5** by two successive acylations of TMU with various lithium organometallics.

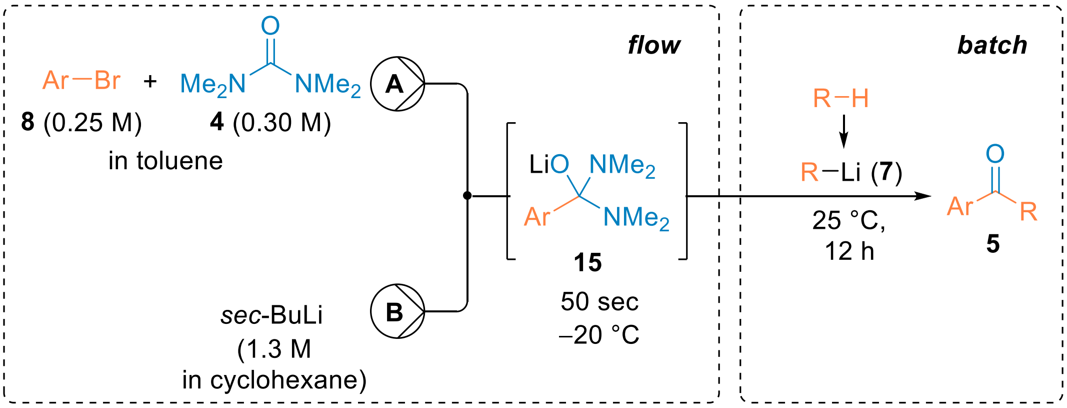

A solution of **8** (0.25 M, 1.0 equiv) and 1,1,3,3-tetramethylurea (**4**, 1.2 equiv) in toluene and a solution of *sec*-BuLi in cyclohexane (1.5 M, 1.2 equiv) were prepared. The solutions were pumped from their flasks through a suction needle at flowrate A = 5.0 $mL \cdot min^{-1}$ and flowrate B =1 $mL \cdot min^{-1}$. The single streams passed a PTFE reactor tube (i.d = 0.8 mm, Vol_{pre} = 2 mL; residence time: t = 20 sec, T = –20 °C) for precooling the solutions and were subsequently mixed in a T-mixer (PFA or PTFE, I.D. = 0.5 mm). The combined stream passed a PTFE reactor tube (i.d = 0.8 mm, Vol_{R1} = 5 mL; residence time: t = 50 sec, T = –20 °C). Then, the combined stream was poured into a flask at 25 °C , containing 1.5 equiv of aryllithiums which were prepared in batch *via* direct metalation of the corresponding starting materials in toluene plus TMEDA (1.0 equiv) with *sec*-BuLi (1.2 equiv) at -20 °C for 30 min. After stirring at 25 °C for 12 h, the reaction mixture was extracted with EtOAc. The combined organic phases were dried over Na_2SO_4 and filtrated. After removal of the solvent *in vacuo*, flash column chromatography purification with *iso*hexane (or pentane):EtOAc mixtures afforded the pure product of type **5**.

Preparation of Products

Preparation of *N,N*-dimethylamides of type 1, 6 and 13

2-Methoxy-*N,N*-dimethylacetamide (1a)

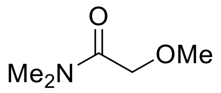

Following **TP1A**, methyl 2-methoxyacetate (20.8 g, 200 mmol) was mixed with $Me_2NH{\cdot}HCl$ (24.4 g, 300 mmol) and NaOMe (113 mL, 600 mmol) in methanol. Thereafter, the reaction mixture was quenched with *sat. aq.* NH_4Cl. After workup (extraction with DCM), the crude product was purified *via* fractional distillation (0.1 mbar, 64 °C) to give 2-methoxy-*N,N*-dimethylacetamide (**1a**) (19.4 g, 166 mmol, 83% yield) as a colorless liquid.

^{1}H-NMR (400 MHz, $CDCl_3$): δ / ppm = 4.06 (s, 2H), 3.40 (s, 3H), 2.94 (d, J = 13.6 Hz, 6H).
^{13}C-NMR (101 MHz, $CDCl_3$): δ / ppm = 169.1, 71.5, 59.1, 36.2, 35.5.
IR (Diamond-ATR, neat): $\tilde{\nu}$ / cm^{-1} = 3500, 2930, 2822, 1641, 1505, 1452, 1415, 1401, 1346, 1263, 1199, 1110, 1012, 927.
MS (EI, 70 eV): *m/z* (%) = 87 (96), 72 (100), 45 (23).
HRMS (EI): *m/z* calc. for [$C_5H_{12}O_2N$]: 118.0868; found 118.0863 [M+H].

2,2-Diethoxy-*N,N*-dimethylacetamide (1b)

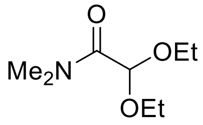

Following **TP1A**, methyl 2,2-diethoxyacetate (32.4g, 200 mmol) was mixed with $Me_2NH{\cdot}HCl$ (24.4 g, 300 mmol) and NaOMe (113 mL, 600 mmol) in methanol. Thereafter, the reaction mixture was quenched with *sat. aq.* NH_4Cl. After workup (extraction with DCM), the crude

product was purified *via* fractional distillation (0.3 mbar, 100 °C) to give 2,2-diethoxy-*N,N*-dimethylacetamide (**1b**) (19.1 mg, 108 mmol, 54% yield) as a colorless liquid.

^{1}H-NMR (400 MHz, $CDCl_3$): δ / ppm = 4.97 (s, 1H), 3.72 (dq, *J* = 9.6, 7.1 Hz, 2H), 3.58 (dq, *J* = 9.5, 7.0 Hz, 2H), 3.13 (s, 3H), 2.94 (s, 3H), 1.24 (t, *J* = 7.1 Hz, 6H).
^{13}C-NMR (101 MHz, $CDCl_3$): δ / ppm = 167.5, 101.1, 63.2 (2C), 36.4, 35.8, 15.1 (2C).
IR (Diamond-ATR, neat): $\tilde{\nu}$ / cm^{-1} = 2976, 2932, 2891, 2882, 1650, 1418, 1401, 1373, 1325, 1152, 1104, 1058, 1027, 985, 681.
MS (EI, 70 eV): *m/z* (%) = 130 (15), 103 (100), 102 (62), 75 (84), 74 (24), 72 (28), 47 (77).
HRMS (EI): *m/z* calc. for [$C_8H_{18}O_3N$]: 176.1287; found 16.1281 [M+H].

2-Fluoro-*N,N*-dimethylacetamide (1c)

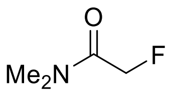

Following **TP1A**, ethyl 2-fluoroacetate (10.6 g, 100 mmol) was mixed with Me_2NH·HCl (12.2 g, 150 mmol) and 30% NaOMe (56.6 mL, 300 mmol) in methanol. Thereafter, the reaction mixture was quenched with 50 ml of *sat. aq.* NH_4Cl. After workup (extraction with Et_2O), the crude product was purified *via* column chromatography (pure ethyl acetate) to give 2-fluoro-*N,N*-dimethylacetamide (**1c**) (8.20 g, 78.0 mmol, 78% yield) as a colorless liquid.

^{1}H-NMR (400 MHz, $CDCl_3$): δ / ppm = 4.95 (d, *J* = 47.2 Hz, 2H), 2.95 (dd, *J* = 2.7, 1.3 Hz, 6H).
^{13}C-NMR (101 MHz, $CDCl_3$): δ / ppm = 166.8 (d, *J* = 18.4 Hz), 79.7 (d, *J* = 178.7 Hz), 35.8 (d, *J* = 4.6 Hz), 35.6.
^{19}F-NMR (377 MHz, $CDCl_3$): δ / ppm = −225.3.
IR (Diamond-ATR, neat): $\tilde{\nu}$ / cm^{-1} = 3481, 2944, 1650, 1645, 1505, 1447, 1425, 1405, 1357, 1263, 1058, 1011, 806.
MS (EI, 70 eV): *m/z* (%) = 105 (53), 72 (100), 44 (9).
HRMS (EI): *m/z* calc. for [C_4H_8ONF]: 105.0590; found 105.0585.

2,2-Difluoro-*N,N*-dimethylacetamide (1d)

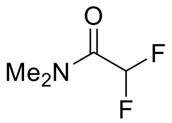

Following **TP1A**, ethyl 2-fluoroacetate (24.8 g, 200 mmol) was mixed with $Me_2NH{\cdot}HCl$ (24.5 g, 300 mmol) and 30% NaOMe (113 mL, 600 mmol) in methanol. Thereafter, the reaction mixture was quenched with 100 ml of *sat. aq.* NH_4Cl. After concentration (1 h at 280 mbar, 40 °C), salts were dissolved with distilled water and product was extracted with Et_2O. After drying with $MgSO_4$ over night solvents were evaporated (atmospheric pressure, 40 °C, then 200 mbar, 40 °C, 15 min) to give 2,2-difluoro-*N,N*-dimethylacetamide (**1d**) (21.3 g, 173 mmol, 87% yield) as a colorless liquid.

^{1}H-NMR (400 MHz, CDCl$_3$): δ / ppm = 6.09 (t, J = 53.7 Hz, 1H), 3.12 (t, J = 1.6 Hz, 3H), 2.99 (t, J = 1.1 Hz, 3H).
^{13}C-NMR (101 MHz, CDCl$_3$): δ / ppm = 162.1 (t, J = 25.0 Hz), 110.4 (t, J = 253.5 Hz), 36.0, 35.9 (t, J = 4.4 Hz).
^{19}F-NMR (377 MHz, CDCl$_3$): δ / ppm = –121.8.
IR (Diamond-ATR, neat): $\tilde{\nu}$ / cm^{-1} = 1671, 1668, 1105, 1049, 863.
MS (EI, 70 eV): *m/z* (%) = 123 (31), 72 (100).
HRMS (EI): *m/z* calc. for [$C_4H_7ONF_2$]: 123.0496; 123.0491.

3-(2-((*Tert*-butyldimethylsilyl)oxy)phenyl)-*N,N*-dimethylpropanamide (1f)

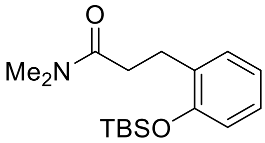

Following **TP1A**, dihydrocoumarine (7.40 g, 50.0 mmol) was mixed with $Me_2NH{\cdot}HCl$ (6.10 g, 75.0 mmol) and NaOMe (28.3 mL, 150 mmol) in methanol. Thereafter, the reaction mixture was quenched with *sat. aq.* NH_4Cl. After workup (extraction with DCM), the crude product 3-(2-hydroxyphenyl)-*N,N*-dimethylpropanamide was directly used for the protection step.

To a solution of 3-(2-hydroxyphenyl)-*N,N*-dimethylpropanamide in THF (100 mL) was added TBDMSCl (7.50 g, 50.0 mmol) and Et_3N (8.40 mL, 60 mmol). The reaction mixture was stirred for 24 h at rt. Then, the mixture was extracted with EtOAc, dried over $MgSO_4$ and filtrated. After removing the solvents in *vacuo*, the crude product was purified *via* column chromatography (pentane:ethyl acetate = 8:2) to give 3-(2-((*tert*-butyldimethylsilyl)oxy)-phenyl)-*N,N*-dimethylpropanamide (**1f**) (10.3 g, 33.5 mmol, 67% overall yield) as a colorless liquid.

^{1}H-NMR (400 MHz, $CDCl_3$): δ / ppm = 7.20 (dd, *J* = 7.4, 1.8 Hz, 1H), 7.11 (ddd, *J* = 8.0, 7.4, 1.8 Hz, 1H), 6.90 (td, *J* = 7.4, 1.2 Hz, 1H), 6.81 (dd, *J* = 8.1, 1.2 Hz, 1H), 2.99 – 2.90 (m, 8H), 2.64 – 2.58 (m, 2H), 1.03 (s, 9H), 0.27 (s, 6H).
^{13}C-NMR (101 MHz, $CDCl_3$): δ / ppm = 172.4, 153.5, 131.8, 130.3, 127.0, 121.0, 118.2, 36.9, 35.2, 33.2, 26.6, 25.6 (3C), 18.1, -4.3 (2C).
IR (Diamond-ATR, neat): $\tilde{\nu}$ / cm^{-1} = 2951, 2928, 2857, 1649, 1490, 1471, 1462, 1452, 1409, 1395, 1250, 1138, 1103, 921, 837, 824, 809, 779, 755.
MS (EI, 70 eV): *m/z* (%) = 251 (39), 250 (100), 102 (17), 73 (14).
HRMS (EI): *m/z* calc. for $[C_{17}H_{28}O_2NSi]^+$: 306.1884; found 306.1888 $[M-H]^+$.

N,N-Dimethyl-3-(piperidin-1-yl)propanamide (1g)

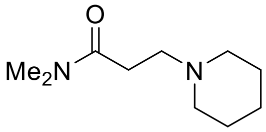

Following **TP1A**, ethyl 3-(piperidin-1-yl)propanoate (18.5 g, 100 mmol) was mixed with $Me_2NH{\cdot}HCl$ (12.2 g, 150 mmol) and NaOMe (60.0 mL, 300 mmol) in methanol. Thereafter, the reaction mixture was quenched with *sat. aq.* NH_4Cl. After workup (extraction with DCM), the crude product was purified *via* column chromatography (pentane:ethyl acetate =3:7) to give *N,N*-dimethyl-3-(piperidin-1-yl)propanamide (**1g**) (13.1 g, 71.1 mmol, 71% yield) as a yellow oil.

^{1}H-NMR (400 MHz, $CDCl_3$): δ / ppm = 2.98 (s, 3H), 2.90 (s, 3H), 2.68 – 2.61 (m, 2H), 2.53 – 2.47 (m, 2H), 2.38 (t, *J* = 5.3 Hz, 4H), 1.59 – 1.50 (m, 4H), 1.40 (ddt, *J* = 7.9, 4.5, 2.6 Hz, 2H).

^{13}C-NMR (101 MHz, $CDCl_3$): δ / ppm = 172.0, 54.8, 54.6 (2C), 37.2, 35.3, 31.2, 26.0 (2C), 24.3.

IR (Diamond-ATR, neat): $\tilde{\nu}$ / cm^{-1} = 3475, 2931, 2853, 2797, 1629, 1497, 1443, 1398, 1393, 1263, 1149, 1116, 1042, 992.

MS (EI, 70 eV): *m/z* (%) = 98 (100), 96 (20), 84 (58), 70 (21).

HRMS (EI): *m/z* calc. for [$C_{10}H_{20}ON_2$]: 184.1576; found 184.1569.

2-((1*r*,4*r*)-4-(4-Chlorophenyl)cyclohexyl)-*N,N*-dimethylacetamide (1h)

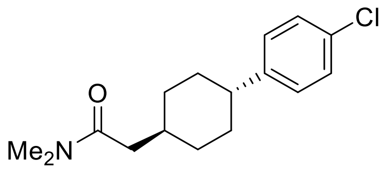

Following **TP1A**, methyl 2-(4-(4-chlorophenyl)cyclohexyl)acetate (13.3 g, 50.0 mmol) was mixed with $Me_2NH{\cdot}HCl$ (8.10 g, 100 mmol) and NaOMe (30.0 mL, 150 mmol) in methanol (100 mL). Thereafter, the reaction mixture was quenched with *sat. aq.* NH_4Cl. After workup, the crude product was purified *via* column chromatography (pentane:ethyl acetate = 7:3) to give 2-((1*r*,4*r*)-4-(4-chlorophenyl)cyclohexyl)-*N,N*-dimethylacetamide (**1h**) (7.80 g, 28.0 mmol, 56% yield) as a white solid.

^{1}H-NMR (400 MHz, $CDCl_3$): δ / ppm = 7.25 – 7.21 (m, 2H), 7.13 – 7.09 (m, 2H), 3.02 (s, 3H), 2.95 (s, 3H), 2.43 (tt, *J* = 12.2, 3.4 Hz, 1H), 2.25 (d, *J* = 6.4 Hz, 2H), 1.97 – 1.81 (m, 5H), 1.53 – 1.41 (m, 2H), 1.19 – 1.07 (m, 2H).

^{13}C-NMR (101 MHz, $CDCl_3$): δ / ppm = 172.3, 145.9, 131.4, 128.4 (2C), 128.2 (2C), 43.7, 40.5, 37.6, 35.4, 34.5, 34.0 (2C), 33.5 (2C).

IR (Diamond-ATR, neat): $\tilde{\nu}$ / cm^{-1} = 2918, 2846, 1637, 1490, 1446, 1408, 1394, 1150, 1127, 1089, 1012, 820.

MS (EI, 70 eV): *m/z* (%) = 279 (12), 125 (10), 87 (100), 72 (11), 44 (21).

HRMS (EI): *m/z* calc. for [$C_{16}H_{22}ONCl$]: 279.1390; found 279.1387.

m.p: 93.3-93.7 °C.

2-(4-Methoxyphenyl)-*N,N*-dimethylacetamide (1i)

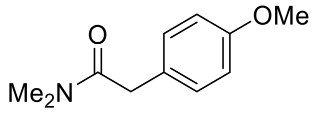

Following **TP1A**, methyl 2-(4-methoxyphenyl)acetate (16.0 mL, 100 mmol) was mixed with $Me_2NH\cdot HCl$ (12.2 g, 150 mmol) and NaOMe (60.0 mL, 300 mmol) in methanol (200 mL). Thereafter, the reaction mixture was quenched with *sat. aq.* NH_4Cl. After workup, the crude product was purified *via* column chromatography (pentane:ethyl acetate = 8:2) to give 2-(4-methoxyphenyl)-*N,N*-dimethylacetamide (**1i**) (15.1 g, 78.1 mmol, 78% yield) as a yellow oil.

^{1}H-NMR (400 MHz, $CDCl_3$): δ / ppm = 7.22 – 7.15 (m, 2H), 6.90 – 6.83 (m, 2H), 3.80 (s, 3H), 3.67 (s, 2H), 2.99 (d, J = 14.4 Hz, 6H).
^{13}C-NMR (101 MHz, $CDCl_3$): δ / ppm = 171.4, 158.4, 129.8 (2C), 127.1, 114.1 (2C), 55.3, 40.1, 37.7, 35.6.
IR (Diamond-ATR, neat): $\tilde{\nu}$ / cm^{-1} = 2934, 1633, 1611, 1511, 1393, 1244, 1177, 1126, 1030, 793.
MS (EI, 70 eV): *m/z* (%) = 193 (29), 148 (12), 121 (100), 72 (11).
HRMS (EI): *m/z* calc. for [$C_{11}H_{15}O_2N$]: 193.1103; found 193.1097.

N,N-Dimethylbicyclo[1.1.1]pentane-1-carboxamide (1j)

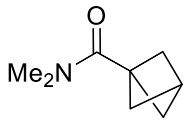

Bicyclo[1.1.1]pentane-1-carboxylic acid[2] (2.24 g, 20.0 mmol) was mixed with CDI (carbonyldiimidazole, 4.86 g, 35.0 mmol, 1.5 equiv) in DCM (50 mL). After stirring for 30 min at rt and degassing the reaction mixture with N_2, Me_2NH (2 M in THF, 40.0 mL, 80.0 mmol, 4.0 equiv) was added. After 16 h of stirring, the reaction mixture was quenched with H_2O and extracted with DCM. After workup, the crude product was purified *via* column chromatography

[2] a) K. Mondanaro, W. P. Dailey, *Org. Synth.* **1998**, *75*, 98. b) M. T. Hossain, J. W. Timberlake, *J. Org. Chem.* **2001**, *66*, 6282-6285. c) I. S. Makarov, C. E. Brocklehurst, K. Karaghiosoff, G. Koch, P. Knochel, *Angew. Chem. Int. Ed.* **2017**, *56*, 12774-12777.

(DCM:MeOH = 9.8:0.2) to give *N,N*-dimethylbicyclo[1.1.1]pentane-1-carboxamide (**1j**) (2.50 g, 18.0 mmol, 90% yield) as a colorless liquid.

^{1}H-NMR (400 MHz, $CDCl_3$): δ / ppm = 3.05 (s, 3H), 2.86 (s, 3H), 2.42 (s, 1H), 2.12 (s, 6H).
^{13}C-NMR (101 MHz, $CDCl_3$): δ / ppm = 169.4, 52.6 (3C), 45.2, 37.3, 35.9, 28.6.
IR (Diamond-ATR, neat): $\tilde{\nu}$ / cm^{-1} = 2971, 2916, 2878, 1613, 1513, 1497, 1393, 1211, 1103, 674.
MS (EI, 70 eV): *m/z* (%) = 138 (73), 124 (59), 94 (70), 72 (97), 67 (100), 66 (52), 65 (57).
HRMS (EI): *m/z* calc. for $[C_9H_{12}ON]^+$: 138.0913; found 138.0913 [M-H$^+$].

4-Cyano-*N,N*-dimethylbenzamide (6a)

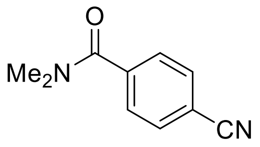

4-Cyanobenzoic acid (11.2 g, 75.8 mmol) was mixed with CDI (13.5 g, 83.4 mmol) in DCM (100 mL). After stirring for 30 min at rt, Me_2NH (2 M in THF, 40.0 mL, 84.0 mmol) was added. After 16 h of stirring, the reaction mixture was quenched with H_2O and extracted with DCM. After workup, the crude product was purified *via* column chromatography (pentane:ethyl acetate = 9:1) to give 4-cyano-*N,N*-dimethylbenzamide (**6a**) (11.2 g, 71.0 mmol, 85% yield) as a yellow solid.

^{1}H-NMR (400 MHz, $CDCl_3$): δ / ppm = 7.75 – 7.65 (m, 2H), 7.55 – 7.45 (m, 2H), 3.10 (s, 3H), 2.93 (s, 3H).
^{13}C-NMR (101 MHz, $CDCl_3$): δ / ppm = 169.5, 140.7, 132.3 (2C), 127.8 (2C), 118.2, 113.3, 39.3, 35.4.
IR (Diamond-ATR, neat): $\tilde{\nu}$ / cm^{-1} = 2934, 2229, 1628, 1512, 1490, 1396, 1266, 1080, 850, 761.
MS (EI, 70 eV): *m/z* (%) = 173 (67), 130 (100), 102 (54), 44 (18), 43 (21).
HRMS (EI): *m/z* calc. for $[C_{10}H_9ON_2]$: 173.0709; found 173.0711.
m.p: 88.3-89.1 °C.

4-Iodo-N,N-dimethylbenzamide (6b)

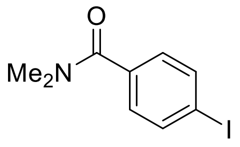

Following **TP1A**, ethyl 4-iodobenzoate (19.3 g, 70 mmol) was mixed with Me_2NH·HCl (8.60 g, 105 mmol) and NaOMe (40 mL, 210 mmol) in methanol. Thereafter, the reaction mixture was quenched with *sat. aq.* NH_4Cl. After workup, the crude product was purified *via* column chromatography (pentane:ethyl acetate = 8:2) to give 4-iodo-*N,N*-dimethylbenzamide (**6b**) (14.9 g, 54.2 mmol, 77% yield) as a white solid.

^{1}H-NMR (400 MHz, $CDCl_3$): δ / ppm = 7.71 (d, *J* = 8.4 Hz, 2H), 7.17 – 7.07 (m, 2H), 2.99 (d, *J* = 49.7 Hz, 6H).
^{13}C-NMR (101 MHz, $CDCl_3$): δ / ppm = 170.6, 137.5 (2C), 135.7, 128.9 (2C), 95.7, 39.5, 35.4.
IR (Diamond-ATR, neat): $\tilde{\nu}$ / cm^{-1} = 2925, 1622, 1584, 1475, 1393, 1264, 1080, 1006, 831, 751.
MS (EI, 70 eV): *m/z* (%) = 274 (80), 230 (100), 202 (24), 76 (11).
HRMS (EI): *m/z* calc. for $[C_9H_9ONI]^+$: 273.9723; found 273.9729 $[M-H]^+$.
m.p: 105.4-106.3 °C.

(*S*)-2-(6-Methoxynaphthalen-2-yl)-*N,N*-dimethylpropanamide (13a)

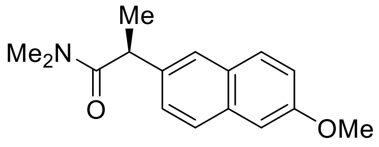

Following **TP1B**, ethyl (*S*)-2-(6-methoxynaphthalen-2-yl)propanoate (4.60 g, 20.0 mmol) was mixed with DCC (4.50 g, 22.0 mmol), HOBt (2.97 g, 22.0 mmol) and Me_2NH (2 M in THF, 11.0 mL, 22.0 mmol). After workup, the crude product was purified *via* column chromatography (pentane:ethyl acetate = 8.5:1.5) to give (*S*)-2-(6-methoxynaphthalen-2-yl)-*N,N*-dimethylpropanamide (**13a**) (4.50 g, 17,6 mmol, 88% yield) as a white solid.

^{1}H-NMR (400 MHz, CDCl$_3$): δ / ppm = 7.67 (dd, *J* = 8.6, 6.8 Hz, 2H), 7.59 (d, *J* = 1.9 Hz, 1H), 7.36 (dd, *J* = 8.5, 1.9 Hz, 1H), 7.14 – 7.06 (m, 2H), 3.97 (q, *J* = 6.8 Hz, 1H), 3.86 (s, 3H), 2.89 (d, *J* = 32.6 Hz, 6H), 1.49 (d, *J* = 6.9 Hz, 3H).
^{13}C-NMR (101 MHz, CDCl$_3$): δ / ppm = 173.7, 157.5, 137.0, 133.4, 129.1, 129.0, 127.4, 126.2, 125.5, 118.9, 105.5, 55.2, 43.1, 37.1, 35.8, 20.7.
IR (Diamond-ATR, neat): $\tilde{\nu}$ / cm^{-1} = 2930, 1640, 1604, 1504, 1484, 1391, 1264, 1228, 1213, 1031, 854.
MS (EI, 70 eV): *m/z* (%) = 257 (11), 186 (12), 185 (100), 170 (27), 153 (12), 141 (14).
HRMS (EI): *m/z* calc. for [$C_{16}H_{19}O_2N$]: 257.1408; found 257.1416.
m.p: 86.8-87.1 °C.
Optical rotation: $[\propto]_D^{20}$ = 111 (c 1.02, CHCl$_3$)
Chiral HPLC: >99% *ee*, OD-H column, heptane:*i*-PrOH = 99:1, 1.5 mL/min, 30 °C.

(*S*)-2-(4-*Iso*butylphenyl)-*N,N*-dimethylpropanamide (13b)

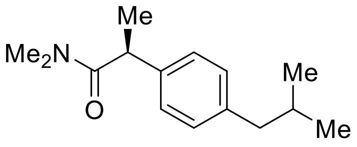

Following **TP1B**, (*S*)-2-(4-*iso*butylphenyl)propanoic acid (5.00 g, 24.2 mmol) was mixed with DCC (5.50 g, 26.6 mmol), HOBt (3.60 g, 26.6 mmol) and Me$_2$NH (2 M in THF, 15.0 mL, 26.6 mmol). After workup, the crude product was purified *via* column chromatography (pentane:ethyl acetate = 8:2 to 7:3) to give (*S*)-2-(4-*iso*butylphenyl)-N,N-dimethylpropanamide (**13b**) (5.13 g, 22.0 mmol, 91% yield) as a colorless liquid.

^{1}H-NMR (400 MHz, CDCl$_3$): δ / ppm = 7.15 (d, *J* = 8.1 Hz, 2H), 7.10 – 7.05 (m, 2H), 3.84 (q, *J* = 6.9 Hz, 1H), 2.94 (s, 3H), 2.88 (s, 3H), 2.43 (d, *J* = 7.2 Hz, 2H), 1.83 (dt, *J* = 13.5, 6.8 Hz, 1H), 1.41 (d, *J* = 6.9 Hz, 3H), 0.88 (dd, *J* = 6.6, 0.9 Hz, 6H).
^{13}C-NMR (101 MHz, CDCl$_3$): δ / ppm = 174.0, 140.2, 139.2, 129.6 (2C), 127.1 (2C), 45.1, 43.0, 37.3, 36.0, 30.3, 22.5, 20.9.
IR (Diamond-ATR, neat): $\tilde{\nu}$ / cm^{-1} = 2951, 2927, 1642, 1509, 1464, 1393, 1146, 1060, 848.
MS (EI, 70 eV): *m/z* (%) = 233 (21), 161 (100), 119 (15), 117 (14), 72 (53).
HRMS (EI): *m/z* calc. for [$C_{15}H_{23}ON$]: 233.1780; found 233.1771.

Optical rotation: $[\alpha]_D^{20}$ = 87 (c 1.09, $CHCl_3$)

Chiral HPLC: >99% *ee*, OD-H column, heptane:*i*-PrOH = 99:1, 1.5 mL/min, 30 °C.

Preparation of Products of type 3, 5, 12 and 14

2-Methoxy-1-(4-(methylthio)phenyl)ethan-1-one (3aa)

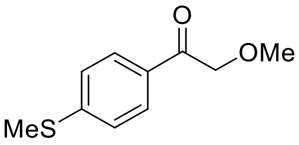

Following **TP2**, solutions of 4-bromothioanisole (**8a**) (0.25 M, 1.0 equiv) with THF (1.0 equiv) in toluene, *sec*-BuLi (1.4 M, 1.2 equiv) and 2-methoxy-*N,N*-dimethylacetamide (**1a**) (0.3 M, 1.2 equiv) in toluene were mixed in continuous flow. After reaching a steady state, the combined stream was collected into *sat. aq.* NH_4Cl for 30 sec, corresponding to 0.625 mmol of the bromide **8a**. After workup, the crude product was purified *via* column chromatography (pentane:ethyl acetate= 9:1) to give **3aa** (101 mg, mmol, 82%) as a colorless solid.

^{1}H-NMR (400 MHz, $CDCl_3$): δ / ppm = 7.89 – 7.82 (m, 2H), 7.27 (d, *J* = 8.3 Hz, 2H), 4.65 (s, 2H), 3.50 (s, 3H), 2.52 (s, 3H).

^{13}C-NMR (101 MHz, $CDCl_3$): δ / ppm = 195.4, 146.7, 131.3, 128.5 (2C), 125.2 (2C), 75.4, 59.6, 14.9.

IR (Diamond-ATR, neat): $\tilde{\nu}$ / cm^{-1} = 2990, 2924, 2832, 1683, 1588, 1235, 1190, 1131, 1095, 981, 976, 920, 815.

MS (EI, 70 eV): *m/z* (%) = 166 (15), 151 (100), 123 (11).

HRMS (EI): *m/z* calc. for [$C_{10}H_{12}O_2S$]:196.0558; found 196.0553.

m.p: 61.1-61.9 °C.

Scale Up of 2-Methoxy-1-(4-(methylthio)phenyl)ethan-1-one (3aa)

Following **TP2**, solutions of 4-bromothioanisole (**7a**) (0.25 M, 1.0 equiv) with THF (1.0 equiv) in toluene, *sec*-BuLi (1.4 M, 1.2 equiv) and 2-methoxy-*N,N*-dimethylacetamide (**1a**) (0.3 M, 1.2 equiv) in toluene were mixed in continuous flow. After reaching a steady state, the combined stream was collected into *sat. aq.* NH_4Cl for 6.5 min, corresponding to 8.125 mmol of the bromide **8a**. After workup, the crude product was purified *via* column chromatography (pentane:ethyl acetate= 9:1) to give **3aa** (1.25 g, 6.37 mmol, 78%) as a colorless solid.

2-Methoxy-1-(2-methoxyphenyl)ethan-1-one (3ab)

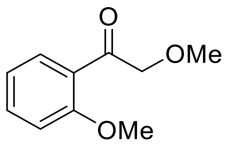

Following **TP2**, solutions of 2-bromoanisole (**8b**) (0.25 M, 1.0 equiv) with THF (1.0 equiv) in toluene, *sec*-BuLi (1.4 M, 1.2 equiv) and 2-methoxy-*N,N*-dimethylacetamide (**1a**) (0.3 M, 1.2 equiv) in toluene were mixed in continuous flow. After reaching a steady state, the combined stream was collected into *sat. aq.* NH_4Cl for 30 sec, corresponding to 0.625 mmol of the bromide **8b**. After workup, the crude product was purified *via* column chromatography (pentane:ethyl acetate= 9:1) to give **3ab** (85.0 mg, 0.47 mmol, 75%) as a yellow solid.

^{1}H-NMR (400 MHz, $CDCl_3$): δ / ppm = 7.84 (dd, *J* = 7.8, 1.9 Hz, 1H), 7.43 (ddd, *J* = 8.4, 7.3, 1.9 Hz, 1H), 6.96 (ddd, *J* = 8.0, 7.3, 1.0 Hz, 1H), 6.90 (dd, *J* = 8.4, 1.0 Hz, 1H), 4.58 (s, 2H), 3.85 (s, 3H), 3.43 (s, 3H).

^{13}C-NMR (101 MHz, $CDCl_3$): δ / ppm = 197.6, 159.3, 134.5, 130.8, 125.4, 121.0, 111.6, 79.2, 59.4, 55.6.

IR (Diamond-ATR, neat): $\tilde{\nu}$ / cm^{-1} = 2942, 1681, 1597, 1484, 1466, 1437, 1286, 1243, 1193, 1182, 1163, 1129, 1108, 1022, 757.

MS (EI, 70 eV): *m/z* (%) = 136 (9), 135 (100), 77 (16).

HRMS (EI): *m/z* calc. for [$C_{10}H_{12}O_3$]: 180.0768; found 180.0781.

m.p: 102.1-102.8 °C.

2-Methoxy-1-(2-methoxyphenyl)ethan-1-one (3ac)

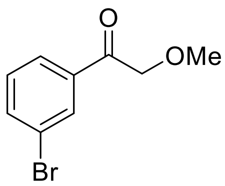

Following **TP2**, solutions of 1,3-dibromobenzene (**8c**) (0.25 M, 1.0 equiv) with THF (1.0 equiv) in toluene, *sec*-BuLi (1.4 M, 1.2 equiv) and 2-methoxy-*N,N*-dimethylacetamide (**1a**) (0.3 M, 1.2 equiv) in toluene were mixed in continuous flow. After reaching a steady state, the combined stream was collected into *sat. aq.* NH_4Cl for 30 sec, corresponding to 0.625 mmol of the bromide **8c**. After workup, the crude product was purified *via* column chromatography (pentane:ethyl acetate= 9.3:0.7) to give **3ac** (121 mg, 0.53 mmol, 85%) as a yellow oil.

^{1}H-NMR (400 MHz, $CDCl_3$): δ / ppm = 8.06 (t, *J* = 1.9 Hz, 1H), 7.85 (ddd, *J* = 7.8, 1.6, 1.1 Hz, 1H), 7.70 (ddd, *J* = 8.0, 2.0, 1.1 Hz, 1H), 7.38 – 7.31 (m, 1H), 4.66 (s, 2H), 3.50 (s, 3H).
^{13}C-NMR (101 MHz, $CDCl_3$): δ / ppm = 195.1, 136.6, 131.1, 130.4, 126.6, 123.2, 75.4, 59.6, 29.8.
IR (Diamond-ATR, neat): $\tilde{\nu}$ / cm^{-1} = 2928, 1704, 1566, 1422, 1220, 1196, 1130, 705, 681.
MS (EI, 70 eV): *m/z* (%) = 200 (36), 184 (100), 183 (80), 157 (44), 155 (45), 76 (28).
HRMS (EI): *m/z* calc. for [$C_9H_9O_2Br$]: 227.9686; found 227.9779.

2-Methoxy-1-(2-methoxyphenyl)ethan-1-one (3ad)

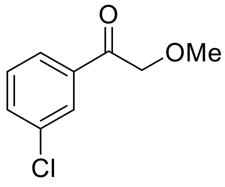

Following **TP2**, solutions of 1-bromo-3-chlorobenzene (**8d**) (0.25 M, 1.0 equiv) with THF (1.0 equiv) in toluene, *sec*-BuLi (1.4 M, 1.2 equiv) and 2-methoxy-*N,N*-dimethylacetamide (**1a**) (0.3 M, 1.2 equiv) in toluene were mixed in continuous flow. After reaching a steady state, the combined stream was collected into *sat. aq.* NH_4Cl for 30 sec, corresponding to 0.625 mmol

of the bromide **8d**. After workup, the crude product was purified *via* column chromatography (pentane:ethyl acetate= 9.3:0.7) to give **3ad** (94.0 mg, 0.51 mmol, 82%) as a yellow solid.

^{1}H-NMR (400 MHz, CDCl$_3$): δ / ppm = 7.91 (ddd, *J* = 2.1, 1.6, 0.5 Hz, 1H), 7.81 (ddd, *J* = 7.7, 1.6, 1.0 Hz, 1H), 7.55 (ddd, *J* = 8.0, 2.1, 1.1 Hz, 1H), 7.44 – 7.37 (m, 1H), 4.66 (s, 2H), 3.50 (s, 3H).
^{13}C-NMR (101 MHz, CDCl$_3$): δ / ppm = 195.2, 136.4, 135.2, 133.6, 130.2, 128.2, 126.1, 75.5, 59.6.
IR (Diamond-ATR, neat): $\tilde{\nu}$ / cm^{-1} = 2927, 2824, 1703, 1571, 1424, 1418, 1223, 1196, 1131, 789, 724, 681.
MS (EI, 70 eV): *m/z* (%) = 156 (11), 154 (32), 141 (33), 139 (100), 111 (21), 75 (11).
HRMS (EI): *m/z* calc. for [$C_{10}H_{12}O_2Cl$]: 184.0291; found 184.0286.
m.p: 45.7-46.2 °C.

1-(5-Bromo-2-methoxyphenyl)-2-methoxyethan-1-one (3ae)

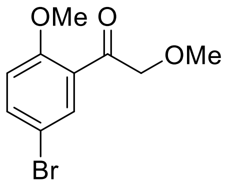

Following **TP2**, solutions of 2,4-dibromo-1-methoxybenzene (**7e**) (0.25 M, 1.0 equiv) with THF (1.0 equiv) in toluene, *sec*-BuLi (1.4 M, 1.2 equiv) and 2-methoxy-*N,N*-dimethylacetamide (**1a**) (0.3 M, 1.2 equiv) in toluene were mixed in continuous flow. After reaching a steady state, the combined stream was collected into *sat. aq.* NH_4Cl for 30 sec, corresponding to 0.625 mmol bromide. After workup, the crude product was purified *via* column chromatography (pentane:ethyl acetate= 9:1) to give **3ae** (124 mg, 0.48 mmol, 77%) as a white solid.

^{1}H-NMR (400 MHz, CDCl$_3$): δ / ppm = 7.99 (d, *J* = 2.6 Hz, 1H), 7.57 (dd, *J* = 8.8, 2.6 Hz, 1H), 6.86 (d, *J* = 8.9 Hz, 1H), 4.60 (s, 2H), 3.91 (s, 3H), 3.48 (s, 3H).
^{13}C-NMR (101 MHz, CDCl$_3$): δ / ppm = 196.3, 158.3, 136.9, 133.4, 126.9, 113.7, 113.6, 79.1, 59.5, 56.0.
IR (Diamond-ATR, neat): $\tilde{\nu}$ / cm^{-1} = 2938, 2822, 1684, 1588, 1479, 1463, 1439, 1395, 1269, 1247, 1177, 1138, 1116, 1016, 988, 928, 810, 661.
MS (EI, 70 eV): *m/z* (%) = 215 (99), 213 (100), 172 (17), 170 (18).

HRMS (EI): *m/z* calc. for [$C_{10}H_{11}O_3Br$]: 257.9892; found 257.9883.
m.p: 65.0-65.6 °C.

2-Methoxy-1-(6-methoxypyridin-2-yl)ethan-1-one (3af)

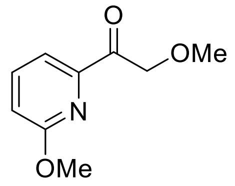

Following **TP2**, solutions of 2-bromo-6-methoxypyridine (**8f**) (0.25 M, 1.0 equiv) with THF (1.0 equiv) in toluene, *sec*-BuLi (1.4 M, 1.2 equiv) and 2-methoxy-*N,N*-dimethylacetamide (**1a**) (0.3 M, 1.2 equiv) in toluene were mixed in continuous flow. After reaching a steady state, the combined stream was collected into *sat. aq.* NH_4Cl for 30 sec, corresponding to 0.625 mmol of the bromide **8f**. After workup, the crude product was purified *via* column chromatography (pentane:ethyl acetate= 9:1) to give **3af** (93.0 mg, 0.51 mmol, 82%) as a yellow oil.

^{1}H-NMR (400 MHz, $CDCl_3$): δ / ppm = 7.71 (dd, *J* = 8.1, 7.3 Hz, 1H), 7.65 (dd, *J* = 7.3, 1.1 Hz, 1H), 6.95 (dd, *J* = 8.1, 1.1 Hz, 1H), 4.99 (s, 2H), 3.95 (s, 3H), 3.53 (s, 3H).
^{13}C-NMR (101 MHz, $CDCl_3$): δ / ppm = 196.9, 163.4, 149.5, 139.4, 116.1, 115.1, 75.3, 59.6, 53.6.
IR (Diamond-ATR, neat): $\tilde{\nu}$ / cm^{-1} = 2952, 2823, 1713, 1590, 1468, 1431, 1325, 1275, 1230, 1200, 1131, 1049, 1037, 986, 809.
MS (EI, 70 eV): *m/z* (%) = 166 (100), 152 (12), 108 (55), 93 (19).
HRMS (EI): *m/z* calc. for [$C_9H_{11}O_3N$]: 181.0739; found 181.0732.

1-(Benzo[*b*]thiophen-5-yl)-2-methoxyethan-1-one (3ag)

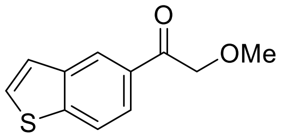

Following **TP2**, solutions of 5-bromobenzo[*b*]thiophene (**8g**) (0.25 M, 1.0 equiv) with THF (1.0 equiv) in toluene, *sec*-BuLi (1.4 M, 1.2 equiv) and 2-methoxy-*N,N*-dimethylacetamide (**1a**) (0.3 M, 1.2 equiv) in toluene were mixed in continuous flow. After reaching a steady state, the combined stream was collected into *sat. aq.* NH_4Cl for 30 sec, corresponding to 0.625 mmol of the bromide **8g**. After workup, the crude product was purified *via* column chromatography (pentane:ethyl acetate= 9.5:0.5) to give **3ag** (134 mg, 0.55 mmol, 89%) as a yellow solid.

^{1}H-NMR (400 MHz, $CDCl_3$): δ / ppm = 8.42 (dd, *J* = 1.7, 0.8 Hz, 1H), 7.97 – 7.86 (m, 2H), 7.56 – 7.50 (m, 1H), 7.43 (dd, *J* = 5.5, 0.7 Hz, 1H), 4.78 (s, 2H), 3.53 (s, 3H).
^{13}C-NMR (101 MHz, $CDCl_3$): δ / ppm = 196.1, 144.8, 139.5, 131.4, 128.1, 124.7, 124.0, 123.0, 122.9, 75.6, 59.6.
IR (Diamond-ATR, neat): $\tilde{\nu}$ / cm^{-1} = 3087, 2926, 2820, 1688, 1594, 1319, 1240, 1201, 1171, 1154, 1122, 1087, 1050, 817, 779, 755, 697.
MS (EI, 70 eV): *m/z* (%) = 176 (17), 162 (10), 161 (28), 161 (100), 133 (18), 89 (16).
HRMS (EI): *m/z* calc. for [$C_{11}H_{10}O_2S$]: 206.0402; found 206.0393.
m.p: 77.3-77.9 °C.

2,2-Diethoxy-1-(4-fluorophenyl)ethan-1-one (3bh)

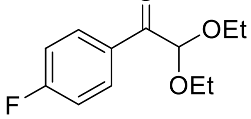

Following **TP2**, solutions of 1-bromo-4-fluorobenzene (**8h**) (0.25 M, 1.0 equiv) with THF (1.0 equiv) in toluene, *sec*-BuLi (1.4 M, 1.2 equiv) and 2,2-diethoxy-*N,N*-dimethylacetamide

(**1b**) (0.3 M, 1.2 equiv) in toluene were mixed in continuous flow. After reaching a steady state, the combined stream was collected into *sat. aq.* NH_4Cl for 30 sec, corresponding to 0.625 mmol of the bromide **8h**. After workup, the crude product was purified *via* column chromatography (pentane:ethyl acetate= 9.8:0.2) to give **3bh** (104 mg, 0.46 mmol, 74%) as a colorless oil.

^{1}H-NMR (400 MHz, $CDCl_3$): δ / ppm = 8.30 – 8.14 (m, 2H), 7.18 – 7.07 (m, 2H), 5.19 (s, 1H), 3.79 (dq, *J* = 9.6, 7.1 Hz, 2H), 3.65 (dq, *J* = 9.6, 7.0 Hz, 2H), 1.26 (t, *J* = 7.0 Hz, 6H).
^{13}C-NMR (101 MHz, $CDCl_3$): δ / ppm = 192.8, 166.1 (d, *J* = 255.4 Hz), 132.8 (d, *J* = 9.3 Hz, 2C), 130.1 (d, *J* = 3.0 Hz), 115.6 (d, *J* = 21.8 Hz, 2C), 103.3, 63.6 (2C), 15.3 (2C).
^{19}F-NMR (377 MHz, $CDCl_3$): δ / ppm = δ -104.3.
IR (Diamond-ATR, neat): $\tilde{\nu}$ / cm^{-1} = 2925, 1695, 1684, 1599, 1235, 1158, 1058, 904, 846, 725, 724, 685.
MS (EI, 70 eV): *m/z* (%) = 153 (49), 123 (44), 123 (100), 103 (50), 97 (72), 95 (34), 75 (77).
HRMS (EI): *m/z* calc. for $[C_{10}H_{10}O_2F]^+$: 181. 0659; found 181.0659 [M-OEt].

2,2-Diethoxy-1-(3-(trifluoromethyl)phenyl)ethan-1-one (3bi)

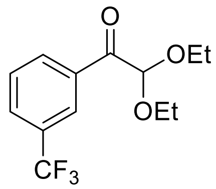

Following **TP2**, solutions of 1-bromo-3-(trifluoromethyl)benzene (**8i**) (0.25 M, 1.0 equiv) with THF (1.0 equiv) in toluene, *sec*-BuLi (1.4 M, 1.2 equiv) and 2,2-diethoxy-*N,N*-dimethylacetamide (**1b**) (0.3 M, 1.2 equiv) in toluene were mixed in continuous flow. After reaching a steady state, the combined stream was collected into *sat. aq.* NH_4Cl for 30 sec, corresponding to 0.625 mmol of the bromide **8i**. After workup, the crude product was purified *via* column chromatography (pentane:ethyl acetate= 9.8:0.2) to give **3bi** (130 mg, 0.47 mmol, 75%) as a colorless oil.

^{1}H-NMR (400 MHz, $CDCl_3$): δ / ppm = 1H NMR (400 MHz, Chloroform-d) δ 8.46 – 8.42 (m, 1H), 8.39 – 8.35 (m, 1H), 7.86 – 7.78 (m, 1H), 7.63 – 7.53 (m, 1H), 5.19 (s, 1H), 3.80 (dq, *J* = 9.6, 7.1 Hz, 2H), 3.65 (dq, *J* = 9.5, 7.0 Hz, 2H), 1.25 (t, *J* = 7.0 Hz, 6H).

^{13}**C-NMR (101 MHz, CDCl$_3$):** δ / ppm = 193.1, 134.2, 133.3, 131.1 (q, *J* = 32.8 Hz), 129.9 (q, *J* = 3.6 Hz), 129.1, 127.0 (q, *J* = 3.8 Hz), 123.9 (q, *J* = 272.5 Hz), 103.4, 63.9 (2C), 29.9, 15.3 (2C).
^{19}**F-NMR (377 MHz, CDCl$_3$):** δ / ppm = –62.9.
IR (Diamond-ATR, neat): $\tilde{\nu}$ / cm^{-1} = 2981, 2927, 1698, 1332, 1262, 1168, 1124, 1096, 1071, 1057, 1002, 693.
MS (EI, 70 eV): *m/z* (%) = 190 (10), 173 (100), 145 (32), 47 (11).
HRMS (EI): *m/z* calc. for $[C_{13}H_{14}O_3F_3]^+$: 275.0890; found 275.0888 [M-H].

2,2-Diethoxy-1-(4-(trifluoromethoxy)phenyl)ethan-1-one (3bj)

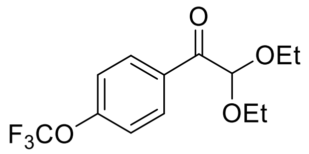

Following **TP2**, solutions of 1-bromo-4-(trifluoromethoxy)benzene (**8j**) (0.25 M, 1.0 equiv) with THF (1.0 equiv) in toluene, *sec*-BuLi (1.4 M, 1.2 equiv) and 2,2-diethoxy-*N,N*-dimethylacetamide (**1b**) (0.3 M, 1.2 equiv) in toluene were mixed in continuous flow. After reaching a steady state, the combined stream was collected into *sat. aq.* NH_4Cl for 30 sec, corresponding to 0.625 mmol of the bromide **8j**. After workup, the crude product was purified *via* column chromatography (pentane:ethyl acetate= 9.8:0.2) to give **3bj** (143 mg, 0.49 mmol, 78%) as a colorless oil.

^{1}**H-NMR (400 MHz, CDCl$_3$):** δ / ppm = 8.31 – 8.17 (m, 2H), 7.35 – 7.15 (m, 2H), 5.16 (s, 1H), 3.77 (dq, *J* = 9.5, 7.1 Hz, 2H), 3.63 (dq, *J* = 9.5, 7.0 Hz, 2H), 1.24 (t, *J* = 7.0 Hz, 6H).
^{13}**C-NMR (101 MHz, CDCl$_3$):** δ / ppm = 192.8, 132.2 (4C), 131.9, 120.2, 103.5, 63.8 (2C), 15.3 (2C).
^{19}**F-NMR (377 MHz, CDCl$_3$):** δ / ppm = -57.5.
IR (Diamond-ATR, neat): $\tilde{\nu}$ / cm^{-1} = 2980, 2873, 1692, 1254, 1208, 1162, 1128, 1111, 1057, 1017, 736, 704.
MS (EI, 70 eV): *m/z* (%) = 219 (34), 189 (100), 163 (38), 123 (28), 103 (43), 95 (26), 77 (31), 75 (61), 47 (39).

HRMS (EI): *m/z* calc. for $[C_{11}H_{10}O_3F_3]^+$: 247.0577; found 247.0579 [M-OEt].

1-(Benzo[*b*]thiophen-5-yl)-2-fluoroethan-1-one (3cg)

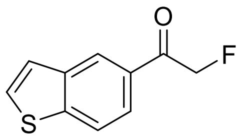

Following **TP2**, solutions of 5-bromobenzo[*b*]thiophene (**8g**) (0.25 M, 1.0 equiv) with THF (1.0 equiv) in toluene, *sec*-BuLi (1.4 M, 1.2 equiv) and 2-fluoro-*N,N*-dimethylacetamide (**1c**) (0.3 M, 1.2 equiv) in toluene were mixed in continuous flow. After reaching a steady state, the combined stream was collected into *sat. aq.* NH_4Cl for 30 sec, corresponding to 0.625 mmol of the bromide **8g**. After workup, the crude product was purified *via* column chromatography (pentane:ethyl acetate= 9.5:0.5) to give **3cg** (80.0 mg, 0.41 mmol, 66%) as a white solid.

^{1}H-NMR (400 MHz, $CDCl_3$): δ / ppm = 8.37 (d, *J* = 1.7 Hz, 1H), 7.97 (dt, *J* = 8.5, 0.8 Hz, 1H), 7.86 (dd, *J* = 8.5, 1.7 Hz, 1H), 7.56 (dd, *J* = 5.5, 0.5 Hz, 1H), 7.44 (dd, *J* = 5.5, 0.8 Hz, 1H), 5.60 (d, *J* = 47.0 Hz, 2H).

^{13}C-NMR (101 MHz, $CDCl_3$): δ / ppm = 193.3 (d, *J* = 15.5 Hz), 145.2, 139.4, 130.2, 128.4, 124.6, 123.7 (d, *J* = 3.1 Hz), 123.0, 122.75 (d, *J* = 2.4 Hz), 83.7 (d, *J* = 182.5 Hz).

IR (Diamond-ATR, neat): $\tilde{\nu}$ / cm^{-1} = 3084, 2941, 1694, 1594, 1547, 1439, 1383, 1321, 1244, 1232, 1178, 1096, 1080, 1047, 1004, 994, 977, 897, 811, 776, 752, 717, 694, 683.

MS (EI, 70 eV): *m/z* (%) = 194 (26), 162 (10), 161 (31), 161 (100), 133 (22), 89 (20).

HRMS (EI): *m/z* calc. for $[C_{10}H_7OFS]$: 194.0195; found 194.0202.

m.p: 87.9-88.5 °C.

2-Fluoro-1-(4-fluorophenyl)ethan-1-one (3ch)

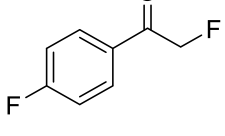

Following **TP2**, solutions of 1-bromo-4-fluorobenzene (**8h**) (0.25 M, 1.0 equiv) with THF (1.0 equiv) in toluene, *sec*-BuLi (1.4 M, 1.2 equiv) and 2-fluoro-*N,N*-dimethylacetamide (**1c**) (0.3 M, 1.2 equiv) in toluene were mixed in continuous flow. After reaching a steady state, the combined stream was collected into *sat. aq.* NH_4Cl for 30 sec, corresponding to 0.625 mmol of the bromide **8h**. After workup, the crude product was purified *via* column chromatography (pentane:ethyl acetate= 9.6:0.4) to give **3ch** (51.0 mg, 0.33 mmol, 52%) as a white solid.

^{1}H-NMR (400 MHz, $CDCl_3$): δ / ppm = 8.03 – 7.89 (m, 2H), 7.21 – 7.14 (m, 2H), 5.48 (d, *J* = 46.9 Hz, 2H).
^{13}C-NMR (101 MHz, $CDCl_3$): δ / ppm = 192.2 (d, *J* = 15.9 Hz), 166.4 (d, *J* = 256.6 Hz), 130.9 (dd, *J* = 9.5, 3.1 Hz, 2C), 130.4 (d, *J* = 3.1 Hz), 116.3 (d, *J* = 22.0 Hz, 2C), 83.7 (d, *J* = 183.2 Hz).
^{19}F-NMR (377 MHz, $CDCl_3$): δ / ppm = –102.8 (m), –229.39 (t, *J* = 46.9 Hz).
IR (Diamond-ATR, neat): $\tilde{\nu}$ / cm^{-1} = 2921, 1698, 1683, 1593, 1507, 1407, 1231, 1163, 1158, 1102, 1081, 975, 832.
MS (EI, 70 eV): *m/z* (%) = 123 (100), 95 (49), 75 (17), 57 (20).
HRMS (EI): *m/z* calc. for [$C_8H_6OF_2$]: 156.0387; found 156.0378.
m.p: 50.9-51.4 °C.

2-Fluoro-1-(4-(trifluoromethoxy)phenyl)ethan-1-one (3cj)

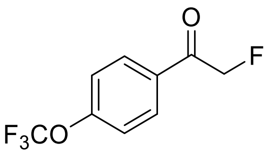

Following **TP2**, solutions of 1-bromo-4-(trifluoromethoxy)benzene (**8j**) (0.25 M, 1.0 equiv) with THF (1.0 equiv) in toluene, *sec*-BuLi (1.4 M, 1.2 equiv) and 2-fluoro-*N,N*-dimethylacetamide (**1c**) (0.3 M, 1.2 equiv) in toluene were mixed in continuous flow. After reaching a steady state, the combined stream was collected into *sat. aq.* NH_4Cl for 30 sec, corresponding to 0.625 mmol of the bromide **8j**. After workup, the crude product was purified *via* column chromatography (pentane:ethyl acetate= 9.6:0.4) to give **3cj** (90.0 mg, 0.41 mmol, 65%) as a colorless oil.

^{1}H-NMR (400 MHz, CDCl$_3$): δ / ppm = 8.03 – 7.91 (m, 2H), 7.32 (dp, *J* = 8.0, 1.1 Hz, 2H), 5.49 (d, *J* = 46.9 Hz, 2H).
^{13}C-NMR (101 MHz, CDCl$_3$): δ / ppm = 192.4 (d, *J* = 16.3 Hz), 153.4 (t, *J* = 1.8 Hz), 132.1, 130.3 (d, *J* = 3.1 Hz, 2C), 120.8 (2C), 117.8 (q, *J* = 259.3 Hz), 83.8 (d, *J* = 183.7 Hz).
^{19}F-NMR (377 MHz, CDCl$_3$): δ / ppm = –57.6, –229.4 (t, *J* = 46.9 Hz).
IR (Diamond-ATR, neat): $\tilde{\nu}$ / cm^{-1} = 2934, 1708, 1605, 1253, 1205, 1159, 1110, 1091, 973, 854, 840, 824, 814.
MS (EI, 70 eV): *m/z* (%) = 189 (100), 123 (13), 95 (12).
HRMS (EI): *m/z* calc. for [$C_9H_6O_2F_4$]: 222.0304; found 222.0299.

2-Fluoro-1-(3-(trifluoromethyl)phenyl)ethan-1-one (3ci)

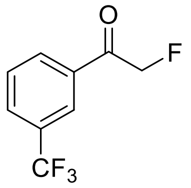

Following **TP2**, solutions of 1-bromo-3-(trifluoromethyl)benzene (**8i**) (0.25 M, 1.0 equiv) with THF (1.0 equiv) in toluene, *sec*-BuLi (1.4 M, 1.2 equiv) and 2-fluoro-*N,N*-dimethylacetamide (**1c**) (0.3 M, 1.2 equiv) in toluene were mixed in continuous flow. After reaching a steady state, the combined stream was collected into *sat. aq.* NH_4Cl for 30 sec, corresponding to 0.625 mmol of the bromide **8i**. After workup, the crude product was purified *via* column chromatography (pentane:ethyl acetate= 9.6:0.4) to give **3ci** (62.0 mg, 0.30 mmol, 48%) as a colorless oil.

^{1}H-NMR (400 MHz, CDCl$_3$): δ / ppm = 8.22 – 8.17 (m, 1H), 8.15 – 8.09 (m, 1H), 7.91 (dddd, *J* = 7.8, 1.8, 1.2, 0.6 Hz, 1H), 7.68 (tt, *J* = 7.9, 0.7 Hz, 1H), 5.55 (d, *J* = 46.8 Hz, 2H).
^{13}C-NMR (101 MHz, CDCl$_3$): δ / ppm = 192.6 (d, *J* = 16.4 Hz), 134.3, 131.6 (q, *J* = 33.3 Hz), 131.2 (t, *J* = 2.3 Hz), 130.5 (q, *J* = 3.6 Hz), 129.7, 125.1 – 124.9 (m), 123.5 (q, *J* = 272.5 Hz), 83.7 (d, *J* = 184.2 Hz).
^{19}F-NMR (377 MHz, CDCl$_3$): δ / ppm = –63.0, –229.6.
IR (Diamond-ATR, neat): $\tilde{\nu}$ / cm^{-1} = 2922, 2849, 1713, 1615, 1436, 1328, 1265, 1218, 1167,

1122, 1097, 1089, 1070, 1042, 1001, 981, 802, 765, 692, 681.
MS (EI, 70 eV): *m/z* (%) = 187 (11), 173 (100), 145 (58), 125 (10).
HRMS (EI): *m/z* calc. for [$C_9H_7OF_4$]: 207.0424; found 207.0433 [M+H].

2,2-Difluoro-1-(4-(methylthio)phenyl)ethan-1-one (3da)

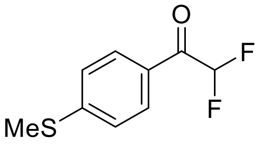

Following **TP2**, solutions of 4-bromothioanisole (**8a**) (0.25 M, 1.0 equiv) with THF (1.0 equiv) in toluene, *sec*-BuLi (1.4 M, 1.2 equiv) and 2,2-difluoro-*N,N*-dimethylacetamide (**1d**) (0.3 M, 1.2 equiv) in toluene were mixed in continuous flow. After reaching a steady state, the combined stream was collected into *sat. aq.* NH_4Cl for 30 sec, corresponding to 0.625 mmol of the bromide **8a**. After workup, the crude product was purified *via* column chromatography (pentane:ethyl acetate= 9.7:0.3) to give **3da** (87.0 mg, 0.43 mmol, 69%) as a white solid.

^{1}H-NMR (400 MHz, $CDCl_3$): δ / ppm = 7.96 (dt, *J* = 8.8, 1.0 Hz, 2H), 7.32 – 7.28 (m, 2H), 6.25 (t, *J* = 53.6 Hz, 1H), 2.53 (s, 3H).
^{13}C-NMR (101 MHz, $CDCl_3$): δ / ppm = 186.7 (t, *J* = 25.3 Hz), 149.1, 130.0 (t, *J* = 2.4 Hz), 127.6 (t, *J* = 1.9 Hz), 125.1, 111.5 (t, *J* = 253.8 Hz), 14.6.
^{19}F-NMR (377 MHz, $CDCl_3$): δ / ppm = –121.5.
IR (Diamond-ATR, neat): $\tilde{\nu}$ / cm^{-1} = 1692, 1587, 1575, 1553, 1256, 1142, 1093, 1071, 1049, 978, 969, 964, 956, 870, 815, 747, 672.
MS (EI, 70 eV): *m/z* (%) = 202 (15), 151 (100), 123 (12).
HRMS (EI): *m/z* calc. for [$C_9H_8OF_2S$]: 202.0257; found 202.0264.
m.p: 100.6-101.6 °C.

2,2-Difluoro-1-(4-methoxyphenyl)ethan-1-one (3dk)

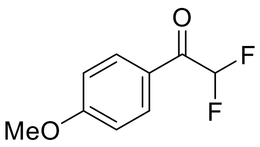

Following **TP2**, solutions of 4-bromoanisole (**8k**) (0.25 M, 1.0 equiv) with THF (1.0 equiv) in toluene, *sec*-BuLi (1.4 M, 1.2 equiv) and 2,2-difluoro-*N,N*-dimethylacetamide (**1d**) (0.3 M, 1.2 equiv) in toluene were mixed in continuous flow. After reaching a steady state, the combined stream was collected into *sat. aq.* NH_4Cl for 30 sec, corresponding to 0.625 mmol of the bromide **8k**. After workup, the crude product was purified *via* column chromatography (pentane:ethyl acetate= 9.5:0.5) to give **3dk** (86.0 mg, 0.46 mmol, 74%) as a colorless oil.

^{1}H-NMR (400 MHz, $CDCl_3$): δ / ppm = 8.06 (dt, *J* = 9.1, 1.0 Hz, 2H), 7.04 – 6.91 (m, 2H), 6.25 (t, *J* = 53.7 Hz, 1H), 3.90 (s, 3H).
^{13}C-NMR (101 MHz, $CDCl_3$): δ / ppm = 186.0 (t, *J* = 25.0 Hz), 164.0, 132.1 (t, *J* = 2.4 Hz, 2C), 124.4 (2C), 114.3, 111.5 (t, *J* = 253.7 Hz), 55.6.
^{19}F-NMR (377 MHz, $CDCl_3$): δ / ppm = -121.4 (d, *J* = 53.6 Hz).
IR (Diamond-ATR, neat): $\tilde{\nu}$ / cm^{-1} = 1692, 1595, 1572, 1513, 1310, 1250, 1176, 1129, 1119, 1053, 1022, 976, 871.
MS (EI, 70 eV): *m/z* (%) = 135 (100), 77 (17).
HRMS (EI): *m/z* calc. for [$C_9H_8O_2F_2$]: 186.0492; found 186.0489.

2,2-Difluoro-1-(3-((5-(4-fluorophenyl)thiophen-2-yl)methyl)-4-methylphenyl)ethan-1-one (3dl)

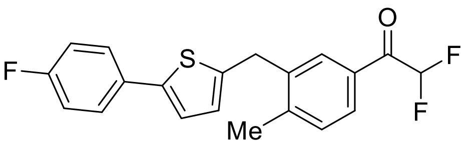

Following **TP2**, solutions of 2-(5-bromo-2-methylbenzyl)-5-(4-fluorophenyl)thiophene (**8l**) (0.25 M, 1.0 equiv) with THF (1.0 equiv) in toluene, *sec*-BuLi (1.4 M, 1.2 equiv) and 2,2-difluoro-*N,N*-dimethylacetamide (**1d**) (0.3 M, 1.2 equiv) in toluene were mixed in continuous flow. After reaching a steady state, the combined stream was collected into *sat. aq.* NH_4Cl for 30 sec, corresponding to 0.625 mmol of the bromide **8l**. After workup, the crude product was purified *via* column chromatography (pentane:ethyl acetate= 9:1) to give **3dl** (169 mg, 0.47 mmol, 75%) as a green solid.

^{1}H-NMR (400 MHz, CDCl$_3$): δ / ppm = 7.95 (d, *J* = 1.9 Hz, 1H), 7.90 (dq, *J* = 7.9, 1.4 Hz, 1H), 7.51 – 7.44 (m, 2H), 7.34 (d, *J* = 8.0 Hz, 1H), 7.06 – 7.00 (m, 3H), 6.67 (dt, *J* = 3.6, 1.1 Hz, 1H), 6.28 (t, *J* = 53.6 Hz, 1H), 4.20 (d, *J* = 1.1 Hz, 2H), 2.42 (s, 3H).
^{13}C-NMR (101 MHz, CDCl$_3$): δ / ppm = 187.2 (t, *J* = 25.1 Hz), 163.4, 161.0, 144.9, 141.9 (d, *J* = 8.0 Hz), 139.3, 131.2, 130.6 (q, *J* = 2.4, 2.0 Hz), 129.9 – 129.8 (m), 128.4 (t, *J* = 2.6 Hz), 127.2 (d, *J* = 7.9 Hz, 2C), 126.3, 122.8 (d, *J* = 1.3 Hz), 115.8 (d, *J* = 21.7 Hz, 2C), 111.2 (t, *J* = 253.7 Hz), 34.1, 20.0.
^{19}F-NMR (377 MHz, CDCl$_3$): δ / ppm = −114.8 – −114.9 (m), −121.8 (d, *J* = 53.7 Hz).
IR (Diamond-ATR, neat): $\tilde{\nu}$ / cm^{-1} = 1705, 1704, 1699, 1694, 1605, 1508, 1231, 1159, 1098, 1057, 833, 809, 802.
MS (EI, 70 eV): *m/z* (%) = 360 (84), 309 (48), 233 (20), 191 (59), 178 (100), 131 (50).
HRMS (EI): *m/z* calc. for [$C_{20}H_{15}OSF_3$]: 360.0796; found 360.0792.
m.p: 58.8-59.4 °C.

2-Chloro-1-(6-methoxypyridin-2-yl)ethan-1-one (3ef)

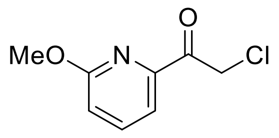

Following **TP2**, solutions of 2-bromo-6-methoxypyridine (**8f**) (0.25 M, 1.0 equiv) with THF (1.0 equiv) in toluene, *sec*-BuLi (1.4 M, 1.2 equiv) and 2-chloro-*N,N*-dimethylacetamide (**1e**) (0.3 M, 1.2 equiv) in toluene were mixed in continuous flow. After reaching a steady state, the combined stream was collected into *sat. aq.* NH_4Cl for 30 sec, corresponding to 0.625 mmol

of the bromide **8f**. After workup, the crude product was purified *via* column chromatography (pentane:ethyl acetate= 9.5:0.5) to give **3ef** (90.0 mg, 0.49 mmol, 78%) as a yellow solid.

^{1}H-NMR (400 MHz, CDCl$_3$): δ / ppm = 7.79 – 7.69 (m, 2H), 6.99 (dd, *J* = 7.9, 1.3 Hz, 1H), 5.07 (s, 2H), 3.98 (s, 3H).
^{13}C-NMR (101 MHz, CDCl$_3$): δ / ppm = 192.0, 163.5, 149.1, 139.5, 116.6, 115.9, 53.7, 47.6.
IR (Diamond-ATR, neat): $\tilde{\nu}$ / cm^{-1} = 2951, 1709, 1601, 1589, 1470, 1430, 1380, 1346, 1285, 1209, 1187, 1155, 1041, 1010, 986, 808, 781, 735, 729.
MS (EI, 70 eV): *m/z* (%) = 187 (20), 185 (65), 136 (57), 126 (22), 108 (100), 93 (20).
HRMS (EI): *m/z* calc. for [$C_8H_8O_2ClN$]: 185.0244; found 185.0238.
m.p: 82.1-82.9 °C.

3-(2-((*Tert*-butyldimethylsilyl)oxy)phenyl)-1-(6-methoxypyridin-2-yl)propan-1-one (3ff)

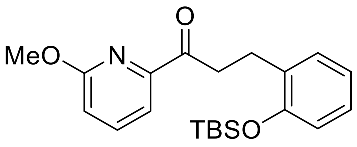

Following **TP2**, solutions of 2-bromo-6-methoxypyridine (**8f**) (0.25 M, 1.0 equiv) with THF (1.0 equiv) in toluene, *sec*-BuLi (1.4 M, 1.2 equiv) and 3-(2-((*tert*-butyldimethy-lsilyl)oxy)phenyl)-*N,N*-dimethylpropanamide (**1f**) (0.3 M, 1.2 equiv) in toluene were mixed in continuous flow. After reaching a steady state, the combined stream was collected into *sat. aq.* NH_4Cl for 30 sec, corresponding to 0.625 mmol of the bromide **8f**. After workup, the crude product was purified *via* column chromatography (pentane:ethyl acetate= 9.7:0.3) to give **3fc** (161 mg, 0.43 mmol, 69%) as a colorless oil.

^{1}H-NMR (400 MHz, CDCl$_3$): δ / ppm = 7.67 (dd, *J* = 8.1, 7.3 Hz, 1H), 7.62 (dd, *J* = 7.3, 1.0 Hz, 1H), 7.21 (dd, *J* = 7.5, 1.8 Hz, 1H), 7.08 (td, *J* = 7.7, 1.8 Hz, 1H), 6.91 – 6.86 (m, 2H), 6.79 (dd, *J* = 8.1, 1.2 Hz, 1H), 3.93 (s, 3H), 3.48 (dd, *J* = 8.3, 7.0 Hz, 2H), 3.01 (t, *J* = 7.7 Hz, 2H), 0.98 (s, 9H), 0.23 (s, 6H).
^{13}C-NMR (101 MHz, CDCl$_3$): δ / ppm = 201.3, 163.4, 153.9, 151.1, 139.2, 132.3, 130.6, 127.2, 121.2, 118.5, 115.3, 115.0, 53.6, 38.2, 25.9, 25.3 (3C), 18.3, -4.0 (2C).
IR (Diamond-ATR, neat): $\tilde{\nu}$ / cm^{-1} = 2951, 2928, 2857, 1698, 1589, 1490, 1467, 1453, 1250,

1027, 921, 837, 824, 811, 808, 778, 755, 731.

MS (EI, 70 eV): *m/z* (%) = 315 (31), 314 (58), 208 (100), 109 (13).

HRMS (EI): *m/z* calc. for $[C_{21}H_{28}O_3NSi]^+$: 370.1833; found 370.1823 $[M-H]^+$.

3-(2-((*Tert*-butyldimethylsilyl)oxy)phenyl)-1-(3-(diethoxymethyl)phenyl)propan-1-one (3fm)

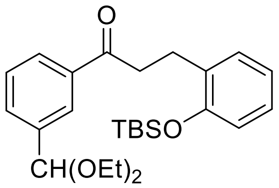

Following **TP2**, solutions of 1-bromo-3-(diethoxymethyl)benzene (**8m**) (0.25 M, 1.0 equiv) with THF (1.0 equiv) in toluene, *sec*-BuLi (1.4 M, 1.2 equiv) and 3-(2-((*tert*-butyldimethyl-silyl)oxy)phenyl)-*N,N*-dimethylpropanamide (**1f**) (0.3 M, 1.2 equiv) in toluene were mixed in continuous flow. After reaching a steady state, the combined stream was collected into *sat. aq.* NH_4Cl for 30 sec, corresponding to 0.625 mmol of the bromide **8m**. After workup, the crude product was purified *via* column chromatography (pentane:ethyl acetate= 9.7:0.3) to give **3fm** (225 mg, 0.51 mmol, 81%) as a colorless oil.

^{1}H-NMR (400 MHz, $CDCl_3$): δ / ppm = 8.05 (dd, *J* = 2.1, 1.3 Hz, 1H), 7.91 (dt, *J* = 7.8, 1.5 Hz, 1H), 7.68 (dq, *J* = 7.7, 0.9 Hz, 1H), 7.45 (t, *J* = 7.7 Hz, 1H), 7.21 (dd, *J* = 7.5, 1.8 Hz, 1H), 7.10 (td, *J* = 7.9, 1.8 Hz, 1H), 6.90 (td, *J* = 7.4, 1.2 Hz, 1H), 6.82 (dd, *J* = 8.1, 1.2 Hz, 1H), 5.53 (s, 1H), 3.66 – 3.51 (m, 4H), 3.30 (dd, *J* = 8.6, 6.9 Hz, 2H), 3.05 (dd, *J* = 8.5, 6.9 Hz, 2H), 1.25 (t, *J* = 7.1 Hz, 6H), 0.99 (s, 9H), 0.26 (d, *J* = 0.8 Hz, 6H).

^{13}C-NMR (101 MHz, $CDCl_3$): δ / ppm = 199.6, 153.9, 139.9, 137.2, 131.9, 131.3, 130.6, 128.6, 128.0, 127.3, 126.5, 121.3, 118.6, 101.2, 61.3 (2C), 39.0, 25.9 (3C), 25.6, 18.3, 15.3 (2C), -4.0 (2C).

IR (Diamond-ATR, neat): $\tilde{\nu}$ / cm^{-1} = 2930, 2858, 1687, 1490, 1453, 1251, 1156, 1103, 1052, 919, 837, 824, 810, 779, 755.

MS (EI, 70 eV): *m/z* (%) = 386 (46), 385 (100), 311 (22), 177 (12), 165 (11).

HRMS (EI): *m/z* calc. for $[C_{25}H_{35}O_4Si]^+$: 427.2299; found 427.2302 $[M-CH_3]^+$.

3-(2-((*Tert*-butyldimethylsilyl)oxy)phenyl)-1-(3-fluorophenyl)propan-1-one (3fn)

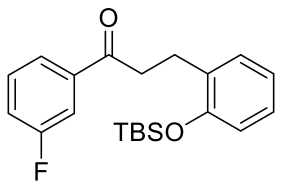

Following **TP2**, solutions of 1-bromo-3-fluorobenzene (**8n**) (0.25 M, 1.0 equiv) with THF (1.0 equiv) in toluene, *sec*-BuLi (1.4 M, 1.2 equiv) and 3-(2-((*tert*-butyldimethy-lsilyl)oxy)phenyl)-*N,N*-dimethylpropanamide (**1f**) (0.3 M, 1.2 equiv) in toluene were mixed in continuous flow. After reaching a steady state, the combined stream was collected into *sat. aq.* NH_4Cl for 30 sec, corresponding to 0.625 mmol of the bromide **8n**. After workup, the crude product was purified *via* column chromatography (pentane:ethyl acetate= 9.7:0.3) to give **3fn** (176 mg, 0.49 mmol, 78%) as a colorless oil.

^{1}H-NMR (400 MHz, $CDCl_3$): δ / ppm = 7.72 (dt, *J* = 7.8, 1.3 Hz, 1H), 7.63 (ddd, *J* = 9.5, 2.7, 1.6 Hz, 1H), 7.42 (td, *J* = 8.0, 5.5 Hz, 1H), 7.26 – 7.22 (m, 1H), 7.20 (dd, *J* = 7.5, 1.8 Hz, 1H), 7.11 (td, *J* = 7.7, 1.8 Hz, 1H), 6.90 (td, *J* = 7.4, 1.2 Hz, 1H), 6.82 (dd, *J* = 8.0, 1.2 Hz, 1H), 3.26 (dd, *J* = 8.6, 6.9 Hz, 2H), 3.04 (dd, *J* = 8.5, 6.9 Hz, 2H), 0.99 (s, 9H), 0.26 (s, 6H).
^{13}C-NMR (101 MHz, $CDCl_3$): δ / ppm = 198.3 (d, *J* = 2.1 Hz), 162.8 (d, *J* = 247.8 Hz), 153.7, 139.1 (d, *J* = 6.0 Hz), 131.5, 130.4, 130.2 (d, *J* = 7.6 Hz), 127.3, 123.7 (d, *J* = 3.0 Hz), 121.2, 119.9 (d, *J* = 21.5 Hz), 118.5, 114.7 (d, *J* = 22.2 Hz), 39.0 (d, *J* = 0.6 Hz), 25.7 (3C), 25.5, 18.2, –4.1 (2C).
^{19}F-NMR (377 MHz, $CDCl_3$): δ / ppm = –112.0.
IR (Diamond-ATR, neat): $\tilde{\nu}$ / cm^{-1} = 2954, 2930, 2858, 1690, 1589, 1490, 1453, 1442, 1250, 1239, 918, 835, 806, 779, 755, 732, 680.
MS (EI, 70 eV): *m/z* (%) = 302 (20), 301 (100), 177 (13), 151 (13), 109 (12), 75 (23).
HRMS (EI): *m/z* calc. for $[C_{20}H_{24}O_2FSi]^+$: 343.1524; found 343.1522 $[M-CH_3]^+$.

1-(4-Methoxyphenyl)-3-(piperidin-1-yl)propan-1-one (3gk)

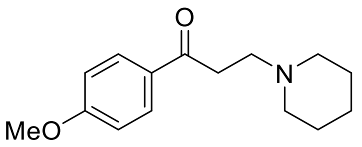

Following **TP2**, solutions of 4-bromoanisole (**8k**) (0.25 M, 1.0 equiv) with THF (1.0 equiv) in toluene, *sec*-BuLi (1.4 M, 1.2 equiv) and *N,N*-dimethyl-3-(piperidin-1-yl)propanamide (**1g**) (0.3 M, 1.2 equiv) in toluene were mixed in continuous flow. After reaching a steady state, the combined stream was collected into *sat. aq.* NH_4Cl for 30 sec, corresponding to 0.625 mmol of the bromide **8k**. After workup, the crude product was purified *via* column chromatography (pentane:ethyl acetate= 4:6) to give **3gk** (94.0 mg, 0.39 mmol, 63%) as a white solid.

^{1}H-NMR (400 MHz, $CDCl_3$): δ / ppm = 7.92 (d, *J* = 8.9 Hz, 2H), 6.90 (d, *J* = 8.8 Hz, 2H), 3.83 (s, 3H), 3.16 (dd, *J* = 8.3, 6.8 Hz, 2H), 2.81 (dd, *J* = 8.2, 6.8 Hz, 2H), 2.48 (s, 4H), 1.60 (p, *J* = 5.7 Hz, 4H), 1.44 (q, *J* = 5.7 Hz, 2H).
^{13}C-NMR (101 MHz, $CDCl_3$): δ / ppm = 197.8, 163.6, 130.4 (2C), 130.1, 113.8 (2C), 55.6 (2C), 54.6, 54.1, 35.8, 25.8 (2C), 24.2.
IR (Diamond-ATR, neat): $\tilde{\nu}$ / cm^{-1} = 2932, 2841, 1672, 1598, 1575, 1509, 1304, 1256, 1241, 1208, 1167, 1154, 1109, 1028, 977, 836.
MS (EI, 70 eV): *m/z* (%) = 162 (16), 135 (63), 98 (100), 97 (45), 92 (12), 84 (15), 77 (15).
HRMS (EI): *m/z* calc. for [$C_{15}H_{21}O_2N$]: 247.1572; found 247.1570.
m.p: 68.1-68.5 °C.

2-((1*r*,4*r*)-4-(4-Chlorophenyl)cyclohexyl)-1-(3,5-dimethoxyphenyl)ethan-1-one (3ho)

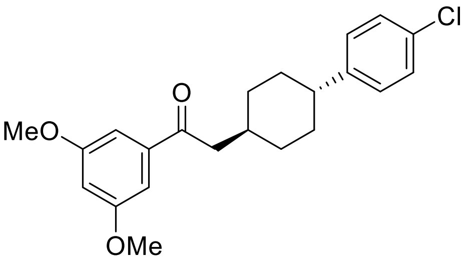

Following **TP2**, solutions of 1-bromo-3,5-dimethoxybenzene (**8o**) (0.25 M, 1.0 equiv) with THF (1.0 equiv) in toluene, *sec*-BuLi (1.4 M, 1.2 equiv) and 2-((1*r*,4*r*)-4-(4-chlorophenyl)-cyclohexyl)-*N,N*-dimethylacetamide (**1h**) (0.3 M, 1.2 equiv) in toluene were mixed in continuous flow. After reaching a steady state, the combined stream was collected into *sat. aq.* NH_4Cl for 30 sec, corresponding to 0.625 mmol of the bromide **8o**. After workup, the crude product was purified *via* column chromatography (pentane:ethyl acetate= 9.7:0.3) to give **3ho** (172 mg, 0.46 mmol, 74%) as a white solid.

^{1}H-NMR (400 MHz, $CDCl_3$): δ / ppm = 7.24 (d, *J* = 8.4 Hz, 2H), 7.14 – 7.09 (m, 4H), 6.66 (t, *J* = 2.3 Hz, 1H), 3.85 (s, 6H), 2.46 (tt, *J* = 12.0, 3.4 Hz, 1H), 2.04 (ddt, *J* = 11.7, 7.0, 4.4 Hz, 1H), 1.97 – 1.83 (m, 4H), 1.48 (qd, *J* = 13.6, 13.0, 3.8 Hz, 2H), 1.27 – 1.11 (m, 2H).
^{13}C-NMR (101 MHz, $CDCl_3$): δ / ppm = 199.7, 161.0, 145.9, 139.5, 131.6, 128.5 (2C), 128.3 (2C), 106.2 (2C), 105.2, 55.7 (2C), 46.1, 43.7, 34.1 (2C), 33.6 (2C).
IR (Diamond-ATR, neat): $\tilde{\nu}$ / cm^{-1} = 2920, 2843, 1686, 1682, 1601, 1591, 1492, 1454, 1447, 1424, 1356, 1314, 1296, 1204, 1153, 1089, 1065, 1028, 1013, 909, 846, 829, 730, 719, 715, 679.
MS (EI, 70 eV): *m/z* (%) = 207 (49), 192 (31), 190 (100), 165 (40), 152 (99), 138 (37).
HRMS (EI): *m/z* calc. for [$C_{22}H_{25}O_3Cl$]: 372.1492; found 372.1487.
m.p: 85.3-85.8 °C.

2-(4-Methoxyphenyl)-1-(4-(methylthio)phenyl)ethan-1-one (3ia)

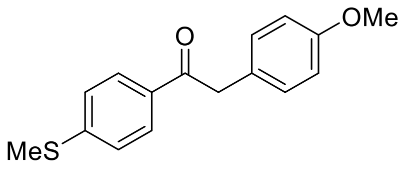

Following **TP2**, solutions of 4-bromothioanisole (**8a**) (0.25 M, 1.0 equiv) with THF (1.0 equiv) in toluene, *sec*-BuLi (1.4 M, 1.2 equiv) and 2-(4-methoxyphenyl)-*N,N*-dimethylacetamide (**1i**) (0.3 M, 1.2 equiv) in toluene were mixed in continuous flow. After reaching a steady state, the combined stream was collected into *sat. aq.* NH_4Cl for 30 sec, corresponding to 0.625 mmol

of the bromide **8a**. After workup, the crude product was purified *via* column chromatography (pentane:ethyl acetate= 9.5:0.5) to give **3ia** (87.0 mg, 0.32 mmol, 51%) as a white solid.

^{1}H-NMR (400 MHz, CDCl$_3$): δ / ppm = 7.96 – 7.87 (m, 2H), 7.26 – 7.24 (m, 2H), 7.20 – 7.12 (m, 2H), 6.91 – 6.80 (m, 2H), 4.17 (s, 2H), 3.78 (s, 3H), 2.51 (s, 3H).
^{13}C-NMR (101 MHz, CDCl$_3$): δ / ppm = 197.1, 158.7, 146.1, 133.1, 130.5 (2C), 129.2 (2C), 126.8, 125.2 (2C), 114.3 (2C), 55.4, 44.7, 14.9.
IR (Diamond-ATR, neat): $\tilde{\nu}$ / cm^{-1} = 2833, 2364, 1681, 1586, 1518, 1250, 1034, 824, 813, 795, 668.
MS (EI, 70 eV): *m/z* (%) = 151 (100), 121 (12).
HRMS (EI): *m/z* calc. for [$C_{16}H_{16}O_2S$]: 272.0871; found 272.0865.
m.p: 111.8-112.2 °C.

1,2-Bis(4-methoxyphenyl)ethan-1-one (3ik)

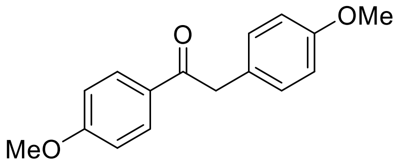

Following **TP2**, solutions of 4-bromoanisole (**8k**) (0.25 M, 1.0 equiv) with THF (1.0 equiv) in toluene, *sec*-BuLi (1.4 M, 1.2 equiv) and 2-(4-methoxyphenyl)-*N,N*-dimethylacetamide (**1i**) (0.3 M, 1.2 equiv) in toluene were mixed in continuous flow. After reaching a steady state, the combined stream was collected into *sat. aq.* NH_4Cl for 30 sec, corresponding to 0.625 mmol of the bromide **8k**. After workup, the crude product was purified *via* column chromatography (pentane:ethyl acetate= 9.2:0.8) to give **3ik** (84.0 mg, 0.33 mmol, 53%) as a white solid.

^{1}H-NMR (400 MHz, CDCl$_3$): δ / ppm = 8.02 – 7.96 (m, 2H), 7.20 – 7.15 (m, 2H), 6.95 – 6.90 (m, 2H), 6.88 – 6.83 (m, 2H), 4.17 (s, 2H), 3.86 (s, 3H), 3.78 (s, 3H).
^{13}C-NMR (101 MHz, CDCl$_3$): δ / ppm = 196.7, 163.6, 158.6, 131.1 (2C), 130.5 (2C), 129.8, 127.1, 114.2 (2C), 113.9 (2C), 55.6, 55.4, 44.5.
IR (Diamond-ATR, neat): $\tilde{\nu}$ / cm^{-1} = 2957, 2904, 2836, 1679, 1601, 1578, 1514, 1257, 1249, 1228, 1202, 1174, 1031, 994, 828, 807.
MS (EI, 70 eV): *m/z* (%) = 135 (100), 77 (10).

HRMS (EI): *m/z* calc. for [$C_{16}H_{16}O_3$]: 256.1099; found 256.1093.
m.p: 129.6-131.0 °C.

Bicyclo[1.1.1]pentan-1-yl(4-butoxyphenyl)methanone (3jp)

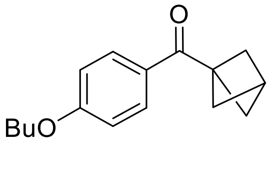

Following **TP2**, solutions of 1-bromo-4-butoxybenzene (**8p**) (0.25 M, 1.0 equiv) with THF (1.0 equiv) in toluene, *sec*-BuLi (1.4 M, 1.2 equiv) and *N,N*-dimethylbicyclo[1.1.1]pentane-1-carboxamide (**1j**) (0.3 M, 1.2 equiv) in toluene were mixed in continuous flow. After reaching a steady state, the combined stream was collected into *sat. aq.* NH_4Cl for 30 sec, corresponding to 0.625 mmol of the bromide **8p**. After workup, the crude product was purified *via* column chromatography (pentane:ethyl acetate= 9.8:0.2) to give **3jp** (91.0 mg, 0.37 mmol, 59%) as a colorless oil.

^{1}H-NMR (400 MHz, $CDCl_3$): δ / ppm = 7.99 (d, *J* = 8.9 Hz, 2H), 6.90 (d, *J* = 8.9 Hz, 2H), 4.02 (t, *J* = 6.5 Hz, 2H), 2.55 (s, 1H), 2.30 (s, 6H), 1.78 (ddt, *J* = 8.9, 7.8, 6.4 Hz, 2H), 1.55 – 1.45 (m, 2H), 0.98 (t, *J* = 7.4 Hz, 3H).
^{13}C-NMR (101 MHz, $CDCl_3$): δ / ppm = 195.9, 163.0, 131.4, 129.4, 114.2, 68.0, 53.5 (3C), 49.5, 31.3, 28.5, 19.3, 13.9.
IR (Diamond-ATR, neat): $\tilde{\nu}$ / cm^{-1} = 2963, 2874, 1655, 1597, 1572, 1508, 1420, 1309, 1252, 1214, 1162, 1135, 984, 969, 884, 843, 793.
MS (EI, 70 eV): *m/z* (%) = 244 (16), 177 (48), 171 (17), 121 (100), 93 (13), 65 (16), 41 (15).
HRMS (EI): *m/z* calc. for [$C_{16}H_{20}O_2$]: 244.1463; found 244.1460.

Bicyclo[1.1.1]pentan-1-yl(4-butoxyphenyl)methanone (3jr)

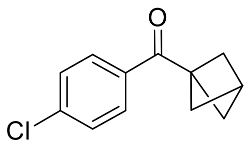

Following **TP2**, solutions of 1-bromo-3-chlorobenzene (**8r**) (0.25 M, 1.0 equiv) with THF (1.0 equiv) in toluene, *sec*-BuLi (1.4 M, 1.2 equiv) and *N,N*-dimethylbicyclo[1.1.1]pentane-1-carboxamide (**1j**) (0.3 M, 1.2 equiv) in toluene were mixed in continuous flow. After reaching a steady state, the combined stream was collected into *sat. aq.* NH_4Cl for 30 sec, corresponding to 0.625 mmol of the bromide **8r**. After workup, the crude product was purified *via* column chromatography (pentane:ethyl acetate= 9.8:0.2) to give **3jr** (90.0 mg, 0.44 mmol, 70%) as a colorless oil.

^{1}H-NMR (400 MHz, $CDCl_3$): δ / ppm = 7.98 – 7.91 (m, 2H), 7.45 – 7.37 (m, 2H), 2.57 (d, *J* = 0.9 Hz, 1H), 2.31 (d, *J* = 0.7 Hz, 6H).
^{13}C-NMR (101 MHz, $CDCl_3$): δ / ppm = 196.2, 139.4, 134.9, 130.5, 128.9, 53.5 (3C), 49.4, 28.7.
IR (Diamond-ATR, neat): $\tilde{\nu}$ / cm^{-1} = 2979, 2878, 1663, 1586, 1569, 1487, 1400, 1314, 1295, 1212, 1170, 1089, 1014, 985, 882, 854, 842, 789, 726.
MS (EI, 70 eV): *m/z* (%) = 171 (15), 141 (27), 139 (100), 111 (34), 75 (18), 42 (17).
HRMS (EI): *m/z* calc. for $[C_{12}H_{10}OCl]^+$: 205.0415; found 205.0432 $[M-H^+]$.

1-((1*r*,4*r*)-4-(4-Chlorophenyl)cyclohexyl)hexan-2-one (3hs)

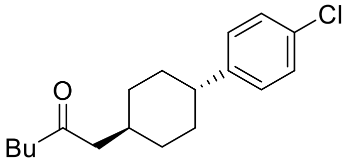

A solution of 2-((1*r*,4*r*)-4-(4-chlorophenyl)-cyclohexyl)-*N,N*-dimethylacetamide (**1h,** 0.30 M, 1.0 equiv) and THF (1.0 equiv) in toluene and a solution of *n*-BuLi in *n*-hexane (0.25 M,

1.2 equiv) were prepared. The solutions were pumped from their flasks through a suction needle at flowrate A = 5.0 mL·min^{-1} and flowrate B = 5.0 mL·min^{-1}. The solutions passed a PTFE reactor tube (i.d = 0.8 mm, Vol_{R1} = 2 mL; residence time: t = 24 sec, T = –20 °C) for precooling the reaction mixture, and then mixed in a T-mixer (PFA or PTFE, I.D. = 0.5 mm). The combined stream passed a PTFE reactor tube (i.d = 0.8 mm, Vol_{R1} = 5 mL; residence time: t = 30 sec, T = –20 °C) and the reaction mixture was subsequently quenched with *sat. aq.* NH_4Cl at 0 °C for 30 sec corresponding to 0.625 mmol of *n*-BuLi. After workup, the crude product was purified *via* column chromatography (pentane:ethyl acetate= 9.8:0.2) to give **3hs** (146 mg, 0.50 mmol, 80%) as a white solid.

^{1}H-NMR (400 MHz, $CDCl_3$): δ / ppm = 7.25 – 7.21 (m, 2H), 7.14 – 7.09 (m, 2H), 2.48 – 2.36 (m, 3H), 2.32 (d, J = 6.7 Hz, 2H), 1.95 – 1.77 (m, 5H), 1.61 – 1.39 (m, 4H), 1.37 – 1.24 (m, 2H), 1.16 – 1.02 (m, 2H), 0.91 (t, J = 7.3 Hz, 3H).
^{13}C-NMR (101 MHz, $CDCl_3$): δ / ppm = 211.1, 145.9, 131.6, 128.5, 128.3, 50.3, 43.7, 43.4, 34.0, 33.5, 33.4, 26.0, 22.5, 14.0.
IR (Diamond-ATR, neat): $\tilde{\nu}$ / cm^{-1} = 2929, 2871, 2849, 1698, 1491, 1406, 1380, 1355, 1128, 1089, 1046, 1012, 960, 823.
MS (EI, 70 eV): *m/z* (%) = 192 (33), 191 (13), 155 (49), 140 (11), 138 (33), 127 (11), 125 (35), 115 (10).
HRMS (EI): *m/z* calc. for [$C_{18}H_{25}OCl$]: 292.1594; found 292.1586.
m.p: 44.5-45.9 °C.

4-(5-Bromo-2-methoxybenzoyl)benzonitrile (12ae)

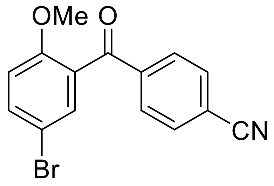

Following **TP2**, solutions of 2,4-dibromo-1-methoxybenzene (**8e**) (0.25 M, 1.0 equiv) with THF (1.0 equiv) in toluene, *sec*-BuLi (1.4 M, 1.2 equiv) and 4-cyano-*N,N*-dimethylbenzamide (**6a**) (0.3 M, 1.2 equiv) in toluene were mixed in continuous flow. After reaching a steady state,

the combined stream was collected into *sat. aq.* NH_4Cl for 30 sec, corresponding to 0.625 mmol of the bromide **8e**. After workup, the crude product was purified *via* column chromatography (pentane:ethyl acetate= 9.3:0.7) to give **12ae** (144 mg, 0.46 mmol, 73%) as a white solid.

^{1}H-NMR (400 MHz, $CDCl_3$): δ / ppm = 7.86 – 7.82 (m, 2H), 7.75 – 7.71 (m, 2H), 7.60 (dd, *J* = 8.8, 2.5 Hz, 1H), 7.52 (d, *J* = 2.5 Hz, 1H), 6.89 (d, *J* = 8.8 Hz, 1H), 3.67 (s, 3H).
^{13}C-NMR (101 MHz, $CDCl_3$): δ / ppm = 193.5, 156.7, 140.8, 135.7, 132.6, 132.3 (2C), 129.9 (2C), 129.2, 118.2, 116.3, 113.5, 113.3, 55.9.
IR (Diamond-ATR, neat): $\tilde{\nu}$ / cm^{-1} = 2935, 2840, 2229, 1672, 1590, 1480, 1460, 1408, 1391, 1293, 1289, 1259, 1235, 1182, 1121, 1020, 949, 937, 859, 818, 770, 675.
MS (EI, 70 eV): *m/z* (%) = 315 (19), 219 (32), 214 (95), 212 (100), 200 (41), 199 (43), 172 (48), 170 (48), 130 (65).
HRMS (EI): *m/z* calc. for [$C_{15}H_{10}O_2NBr$]: 314.9895; found 314.9886.
m.p: 113.6-115.2 °C.

4-(6-Methoxypicolinoyl)benzonitrile (12af)

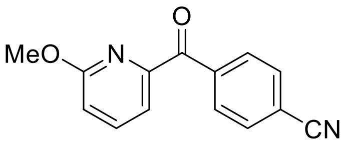

Following **TP2**, solutions of 2-bromo-6-methoxypyridine (**8f**) (0.25 M, 1.0 equiv) with THF (1.0 equiv) in toluene, *sec*-BuLi (1.4 M, 1.2 equiv) and 4-cyano-*N,N*-dimethylbenzamide (**6a**) (0.3 M, 1.2 equiv) in toluene were mixed in continuous flow. After reaching a steady state, the combined stream was collected into *sat. aq.* NH_4Cl for 30 sec, corresponding to 0.625 mmol of the bromide **8f**. After workup, the crude product was purified *via* column chromatography (pentane:ethyl acetate= 9.5:0.5) to give **12af** (118 mg, 0.49 mmol, 79%) as a white solid.

^{1}H-NMR (400 MHz, $CDCl_3$): δ / ppm = 8.32 – 8.15 (m, 2H), 7.87 – 7.66 (m, 4H), 7.00 (dd, *J* = 8.2, 1.0 Hz, 1H), 3.88 (s, 3H).
^{13}C-NMR (101 MHz, $CDCl_3$): δ / ppm = 191.8, 163.1, 151.0, 140.5, 139.6, 131.7 (2C), 131.3 (2C), 118.4, 118.3, 115.7, 115.5, 53.8.

IR (Diamond-ATR, neat): $\tilde{\nu}$ / cm^{-1} = 2991, 2949, 2230, 1662, 1590, 1464, 1336, 1290, 1267, 1151, 1031, 989, 974, 868, 851, 814, 762.

MS (EI, 70 eV): *m/z* (%) = 237 (43), 210 (67), 209 (57), 195 (29), 179 (44), 130 (100), 93 (39), 79 (35).

HRMS (EI): *m/z* calc. for [$C_{14}H_{10}O_2N_2$]: 238.0742; found 238.0743.

m.p: 127.1-127.8 °C.

4-(Benzo[*b*]thiophene-5-carbonyl)benzonitrile (12ag)

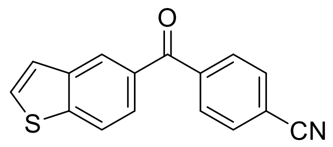

Following **TP2**, solutions of 5-bromobenzo[*b*]thiophene (**8g**) (0.25 M, 1.0 equiv) with THF (1.0 equiv) in toluene, *sec*-BuLi (1.4 M, 1.2 equiv) and 4-cyano-*N,N*-dimethylbenzamide (**6a**) (0.3 M, 1.2 equiv) in toluene were mixed in continuous flow. After reaching a steady state, the combined stream was collected into *sat. aq.* NH_4Cl for 30 sec, corresponding to 0.625 mmol of the bromide **8g**. After workup, the crude product was purified *via* column chromatography (pentane:ethyl acetate= 9.5:0.5) to give **12ag** (100 mg, 0.38 mmol, 61%) as a white solid.

^{1}H-NMR (400 MHz, $CDCl_3$): δ / ppm = 8.20 (d, *J* = 1.7 Hz, 1H), 8.00 (d, *J* = 8.5 Hz, 1H), 7.91 – 7.86 (m, 2H), 7.80 (dd, *J* = 8.3, 1.6 Hz, 3H), 7.57 (d, *J* = 5.4 Hz, 1H), 7.42 (d, *J* = 5.5 Hz, 1H).

^{13}C-NMR (101 MHz, $CDCl_3$): δ / ppm = 195.1, 144.7, 141.8, 139.3, 132.8, 132.3 (2C), 130.3 (2C), 128.5, 126.5, 125.1, 124.6, 122.9, 118.2, 115.6.

IR (Diamond-ATR, neat): $\tilde{\nu}$ / cm^{-1} = 3085, 2229, 1651, 1592, 1402, 1325, 1289, 1274, 1251, 1198, 1088, 1048, 979, 956, 853, 817, 758, 728, 712, 691.

MS (EI, 70 eV): *m/z* (%) = 263 (25), 161 (100), 133 (15), 89 (20).

HRMS (EI): *m/z* calc. for [$C_{16}H_9ONS$]: 263.0405; found 263.0398.

m.p: 157.0-158.0 °C.

4-(3-Fluorobenzoyl)benzonitrile (12an)

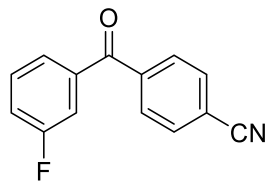

Following **TP2**, solutions of 1-bromo-3-fluorobenzene (**8n**) (0.25 M, 1.0 equiv) with THF (1.0 equiv) in toluene, *sec*-BuLi (1.4 M, 1.2 equiv) and 4-cyano-*N,N*-dimethylbenzamide (**6a**) (0.3 M, 1.2 equiv) in toluene were mixed in continuous flow. After reaching a steady state, the combined stream was collected into *sat. aq.* NH_4Cl for 30 sec, corresponding to 0.625 mmol of the bromide **8n**. After workup, the crude product was purified *via* column chromatography (pentane:ethyl acetate= 9.3:0.7) to give **12an** (90.0 mg, 0.40 mmol, 64%) as a white solid.

^{1}H-NMR (400 MHz, $CDCl_3$): δ / ppm = 7.94 – 7.88 (m, 2H), 7.85 – 7.81 (m, 2H), 7.59 – 7.49 (m, 3H), 7.37 (tdd, *J* = 8.2, 2.6, 1.3 Hz, 1H).
^{13}C-NMR (101 MHz, $CDCl_3$): δ / ppm = 193.8 (d, *J* = 2.2 Hz), 162.8 (d, *J* = 249.2 Hz), 140.7, 138.5 (d, *J* = 6.5 Hz), 132.4 (2C), 130.5 (d, *J* = 7.8 Hz), 130.3 (2C), 126.01(d, *J* = 3.1 Hz), 120.6 (d, *J* = 21.4 Hz), 118.0, 116.9 (d, *J* = 22.6 Hz), 116.2.
^{19}F-NMR (377 MHz, $CDCl_3$): δ / ppm = –111.0.
IR (Diamond-ATR, neat): $\tilde{\nu}$ / cm^{-1} = 3069, 2232, 1664, 1585, 1482, 1440, 1404, 1311, 1294, 1277, 1272, 1208, 857, 839, 758, 710.
MS (EI, 70 eV): *m/z* (%) = 225 (30), 130 (65), 123 (100), 95 (19), 75 (23).
HRMS (EI): *m/z* calc. for [$C_{14}H_8ONF$]: 225.0590; found 225.0582.
m.p: 92.4-93.3 °C.

(3-Bromo-5-fluorophenyl)(4-iodophenyl)methanone (12bq)

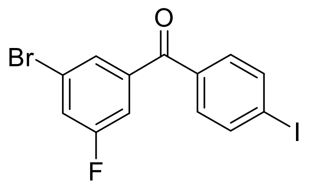

Following **TP2**, solutions of 1,3-dibromo-5-fluorobenzene (**8q**) (0.25 M, 1.0 equiv) with THF (1.0 equiv) in toluene, *sec*-BuLi (1.4 M, 1.2 equiv) and 4-iodo-*N,N*-dimethylbenzamide (**6b**) (0.3 M, 1.2 equiv) in toluene were mixed in continuous flow. After reaching a steady state, the combined stream was collected into *sat. aq.* NH_4Cl for 30 sec, corresponding to 0.625 mmol of the bromide **8q**. After workup, the crude product was purified *via* column chromatography (pentane:ethyl acetate= 9.9:0.1) to give **12bp** (201 mg, 0.49 mmol, 79%) as a white solid.

^{1}H-NMR (400 MHz, $CDCl_3$): δ / ppm = 7.90 – 7.85 (m, 2H), 7.67 (t, *J* = 1.7 Hz, 1H), 7.51 – 7.45 (m, 3H), 7.40 (ddd, *J* = 8.5, 2.4, 1.4 Hz, 1H).
^{13}C-NMR (101 MHz, $CDCl_3$): δ / ppm = 193.0 (d, *J* = 2.1 Hz), 162.4 (d, *J* = 253.7 Hz), 140.3 (d, *J* = 6.7 Hz), 138.1 (2C), 135.7, 131.4 (2C), 128.8 (d, *J* = 3.3 Hz), 123.2 (d, *J* = 24.5 Hz), 123.0, 115.8 (d, *J* = 22.5 Hz), 101.3.
^{19}F-NMR (377 MHz, $CDCl_3$): δ / ppm = –108.9.
IR (Diamond-ATR, neat): $\tilde{\nu}$ / cm^{-1} = 3076, 1661, 1575, 1557, 1427, 1390, 1304, 1278, 1214, 1179, 1001, 989, 864, 835, 766, 750, 658.
MS (EI, 70 eV): *m/z* (%) = 406 (13), 404 (13), 230 (100), 202 (19).
HRMS (EI): *m/z* calc. for [$C_{13}H_7OBrFI$]: 403.8709, found 403.8706.
m.p: 80.5-81.1 °C.

(4-Chlorophenyl)(4-iodophenyl)methanone (12br)

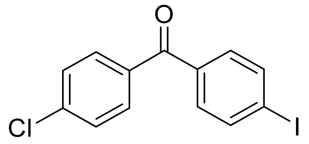

Following **TP2**, solutions of 1-bromo-4-chlorobenzene (**8r**) (0.25 M, 1.0 equiv) with THF (1.0 equiv) in toluene, *sec*-BuLi (1.4 M, 1.2 equiv) and 4-iodo-*N,N*-dimethylbenzamide (**6b**) (0.3 M, 1.2 equiv) in toluene were mixed in continuous flow. After reaching a steady state, the combined stream was collected into *sat. aq.* NH_4Cl for 30 sec, corresponding to 0.625 mmol of the bromide **8r**. After workup, the crude product was purified *via* column chromatography (pentane:ethyl acetate= 9.9:0.1) to give **12bq** (134 mg, 0.39 mmol, 63%) as a white solid.

^{1}H-NMR (400 MHz, $CDCl_3$): δ / ppm = 7.87 – 7.83 (m, 2H), 7.75 – 7.69 (m, 2H), 7.51 – 7.44 (m, 4H).
^{13}C-NMR (101 MHz, $CDCl_3$): δ / ppm = 194.7, 139.3, 137.8 (2C), 136.6, 135.5, 131.5 (2C), 131.4 (2C), 128.9 (2C), 100.5.
IR (Diamond-ATR, neat): $\tilde{\nu}$ / cm^{-1} = 1643, 1580, 1391, 1306, 1302, 1287, 1282, 1093, 1058, 1015, 1008, 926, 905, 853, 825, 748, 730, 665.
MS (EI, 70 eV): *m/z* (%) = 341 (44), 306 (25), 230 (100), 215 (20), 140 (25), 138 (77).
HRMS (EI): *m/z* calc. for [$C_{13}H_8OClI$]: 341.9308; found 341.9303.
m.p: 169.0-170.0 °C.

(4-Chlorophenyl)(4-iodophenyl)methanone (12cr)

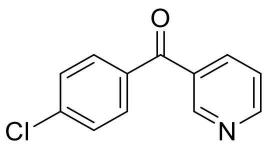

Following **TP2**, solutions of 1-bromo-4-chlorobenzene (**8r**) (0.25 M, 1.0 equiv) with THF (1.0 equiv) in toluene, *sec*-BuLi (1.4 M, 1.2 equiv) and *N,N*-diethylnicotinamide (**6c**) (0.3 M, 1.2 equiv) in toluene were mixed in continuous flow. After reaching a steady state, the combined stream was collected into *sat. aq.* NH_4Cl for 30 sec, corresponding to 0.625 mmol of the bromide **8r**. After workup, the crude product was purified *via* column chromatography (pentane:ethyl acetate= 7:3) to give **12cq** (79.0 mg, 0.36 mmol, 58%) as a white solid.

¹H-NMR (400 MHz, CDCl$_3$): δ / ppm = 8.96 (dd, *J* = 2.3, 0.9 Hz, 1H), 8.82 (dd, *J* = 4.9, 1.7 Hz, 1H), 8.09 (ddd, *J* = 7.9, 2.2, 1.7 Hz, 1H), 7.84 – 7.69 (m, 2H), 7.51 – 7.47 (m, 2H), 7.45 (ddd, *J* = 7.9, 4.8, 0.9 Hz, 1H).
¹³C-NMR (101 MHz, CDCl$_3$): δ / ppm = 193.7, 153.2, 150.9, 139.9, 137.2, 135.1, 133.0, 131.5 (2C), 129.1 (2C), 123.6.
IR (Diamond-ATR, neat): $\tilde{\nu}$ / cm^{-1} = 3050, 1644, 1581, 1570, 1484, 1418, 1401, 1337, 1299, 1282, 1149, 1093, 1024, 1011, 935, 923, 850, 845, 819, 745, 711, 678.
MS (EI, 70 eV): *m/z* (%) = 183 (13), 182 (100), 141 (11), 139 (34).
HRMS (EI): *m/z* calc. for $[C_{12}H_7ONCl]^+$: 216.0211; found 216.0211 $[M-H]^+$.
m.p: 91.0-91.2 °C.

(*S*)-1-(3-Bromophenyl)-2-(6-methoxynaphthalen-2-yl)propan-1-one (14ac)

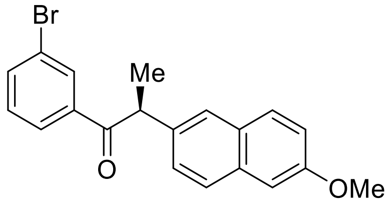

Following **TP2**, solutions of 1,3-bromobenzene (**8c**) (0.25 M, 1.0 equiv) with THF (1.0 equiv) in toluene, *sec*-BuLi (1.4 M, 1.2 equiv) and (*S*)-2-(6-methoxynaphthalen-2-yl)-*N,N*-dimethylpropanamide (**13a**) (0.3 M, 1.2 equiv) in toluene were mixed in continuous flow. After reaching a steady state, the combined stream was collected into *sat. aq.* NH_4Cl for 30 sec, corresponding to 0.625 mmol of the bromide **8c**. After workup, the crude product was purified *via* column chromatography (pentane:ethyl acetate= 9.9:0.1) to give **14ac** (173 mg, 0.47 mmol, 75%, 99% *ee*) as a colorless oil.

¹H-NMR (400 MHz, CDCl$_3$): δ / ppm = 8.14 (t, *J* = 1.8 Hz, 1H), 7.87 (ddd, *J* = 7.8, 1.7, 1.0 Hz, 1H), 7.69 (t, *J* = 9.1 Hz, 2H), 7.63 (d, *J* = 1.8 Hz, 1H), 7.55 (ddd, *J* = 8.0, 2.0, 1.0 Hz, 1H), 7.37 (dd, *J* = 8.4, 1.9 Hz, 1H), 7.20 (t, *J* = 7.9 Hz, 1H), 7.14 (dd, *J* = 8.9, 2.6 Hz, 1H), 7.09 (d, *J* = 2.5 Hz, 1H), 4.74 (q, *J* = 6.8 Hz, 1H), 3.89 (s, 3H), 1.61 (d, *J* = 6.8 Hz, 3H).
¹³C-NMR (101 MHz, CDCl$_3$): δ / ppm = 199.1, 157.9, 138.4, 136.1, 135.7, 133.7, 131.9, 130.1, 129.3, 129.3, 127.9, 127.4, 126.4, 126.4, 123.0, 119.3, 105.7, 55.4, 48.2, 19.5.
IR (Diamond-ATR, neat): $\tilde{\nu}$ / cm^{-1} = 2973, 2931, 1682, 1603, 1483, 1391, 1265, 1224, 1201,

1172, 1162, 1030, 907, 852, 730, 728, 673.

MS (EI, 70 eV): *m/z* (%) = 185 (100), 170 (24), 153 (10), 141 (12).

HRMS (EI): *m/z* calc. for [$C_{20}H_{17}O_2Br$]: 368.0412; found 368.0406.

Optical rotation: $[\alpha]_D^{20}$ = 125 (c 1.09, $CHCl_3$)

Chiral HPLC: 99% *ee*, OD-H column, heptane:*i*-PrOH = 95:5, 1.0 mL/min, 30 °C.

(*S*)-1-(3-(diethoxymethyl)phenyl)-2-(6-methoxynaphthalen-2-yl)propan-1-one (14am)

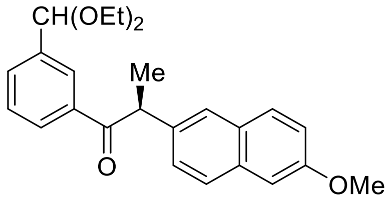

Following **TP2**, solutions of 1-bromo-3-(diethoxymethyl)benzene (**8m**) (0.25 M, 1.0 equiv) with THF (1.0 equiv) in toluene, *sec*-BuLi (1.4 M, 1.2 equiv) and (*S*)-2-(6-methoxynaphthalen-2-yl)-*N,N*-dimethylpropanamide (**13a**) (0.3 M, 1.2 equiv) in toluene were mixed in continuous flow. After reaching a steady state, the combined stream was collected into *sat. aq.* NH_4Cl for 30 sec, corresponding to 0.625 mmol of the bromide **8m**. After workup, the crude product was purified *via* column chromatography (pentane:ethyl acetate= 9.9:0.1) to give **14am** (159 mg, 0.41 mmol, 65%, 99% *ee*) as a colorless oil.

^{1}H-NMR (400 MHz, $CDCl_3$): δ / ppm = 8.14 (t, *J* = 1.8 Hz, 1H), 7.95 (dt, *J* = 7.8, 1.5 Hz, 1H), 7.71 – 7.63 (m, 3H), 7.58 (dt, *J* = 7.6, 1.6 Hz, 1H), 7.44 – 7.33 (m, 2H), 7.16 – 7.02 (m, 2H), 5.48 (s, 1H), 4.84 (q, *J* = 6.8 Hz, 1H), 3.87 (s, 3H), 3.49 (ddtt, *J* = 18.6, 14.1, 9.4, 7.0 Hz, 4H), 1.62 (d, *J* = 6.8 Hz, 3H), 1.19 (td, *J* = 7.1, 2.5 Hz, 6H).

^{13}C-NMR (101 MHz, $CDCl_3$): δ / ppm = 200.2, 157.6, 139.5, 136.7, 136.4, 133.5, 131.1, 129.2, 129.2, 128.7, 128.4, 127.6, 127.2, 126.4, 126.3, 119.0, 105.6, 100.8, 61.1, 60.9, 55.2, 47.9, 19.4 (2C), 15.1 (2C).

IR (Diamond-ATR, neat): $\tilde{\nu}$ / cm^{-1} = 2973, 2931, 1681, 1604, 1263, 1170, 1162, 1113, 1052, 1031, 907, 852, 727.

MS (EI, 70 eV): *m/z* (%) = 207 (11), 185 (100), 183 (17), 170 (24), 153 (13).

HRMS (EI): *m/z* calc. for [$C_{25}H_{28}O_4$]: 392.1988, found 392.1984.

Optical rotation: $[\alpha]_D^{20}$ = 158 (c 0.99, $CHCl_3$)

Chiral HPLC: 99% *ee*, OD-H column, heptane:*i*-PrOH = 98:2, 1.0 mL/min, 30 °C.

(*S*)-1-(3-Fluorophenyl)-2-(6-methoxynaphthalen-2-yl)propan-1-one (14an)

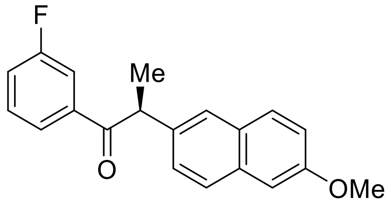

Following **TP2**, solutions of 1-bromo-3-fluorobenzene (**8n**) (0.25 M, 1.0 equiv) with THF (1.0 equiv) in toluene, *sec*-BuLi (1.4 M, 1.2 equiv) and (*S*)-2-(6-methoxynaphthalen-2-yl)-*N,N*-dimethylpropanamide (**13a**) (0.3 M, 1.2 equiv) in toluene were mixed in continuous flow. After reaching a steady state, the combined stream was collected into *sat. aq.* NH_4Cl for 30 sec, corresponding to 0.625 mmol of the bromide **8n**. After workup, the crude product was purified *via* column chromatography (pentane:ethyl acetate= 9.9:0.1) to give **14an** (170 mg, 0.55 mmol, 88%, 99% *ee*) as a colorless oil.

^{1}H-NMR (400 MHz, $CDCl_3$): δ / ppm = 7.75 (ddd, *J* = 7.8, 1.6, 1.0 Hz, 1H), 7.72 – 7.62 (m, 4H), 7.40 – 7.28 (m, 2H), 7.17 – 7.07 (m, 3H), 4.74 (q, *J* = 6.8 Hz, 1H), 3.89 (s, 3H), 1.61 (d, *J* = 6.8 Hz, 3H).
^{13}C-NMR (101 MHz, $CDCl_3$): δ / ppm = 199.1, 162.7 (d, *J* = 247.6 Hz), 157.7, 138.7 (d, *J* = 6.1 Hz), 136.1, 133.5, 130.1 (d, *J* = 7.6 Hz), 129.2, 129.2, 127.8, 126.3, 126.2, 124.5 (d, *J* = 3.0 Hz), 119.7 (d, *J* = 21.5 Hz), 119.1, 115.5 (d, *J* = 22.4 Hz), 105.6, 55.3, 48.2, 19.4.
^{19}F-NMR (377 MHz, $CDCl_3$): δ / ppm = –108.9.
IR (Diamond-ATR, neat): $\tilde{\nu}$ / cm^{-1} = 2973, 2934, 1682, 1604, 1587, 1483, 1439, 1391, 1260, 1246, 1226, 1211, 1173, 1162, 1151, 1030, 924, 907, 889, 886, 852, 815, 806, 803, 768, 751, 727, 673.
MS (EI, 70 eV): *m/z* (%) = 185 (100), 170 (42), 154 (14), 153 (21), 141 (16).
HRMS (EI): *m/z* calc. for [$C_{20}H_{17}O_2F$]: 308.1213; found 308.1204.
Optical rotation: $[\alpha]_D^{20}$ = 96 (c 1.00, $CHCl_3$)
Chiral HPLC: 99% *ee*, OD-H column, heptane:*i*-PrOH = 99.5:0.5, 1.0 mL/min, 30 °C.

(2*S*)-1-(4-Fluorophenyl)-2-(4-(2-methoxypropyl)phenyl)propan-1-one (14bh)

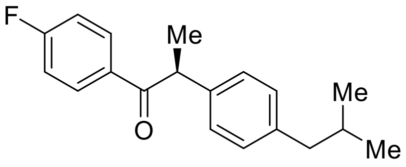

Following **TP2**, solutions of 1-bromo-4-fluorobenzene (**8h**) (0.25 M, 1.0 equiv) with THF (1.0 equiv) in toluene, *sec*-BuLi (1.4 M, 1.2 equiv) and (*S*)-2-(4-*iso*butylphenyl)-*N,N*-dimethylpropanamide (**13b**) (0.3 M, 1.2 equiv) in toluene were mixed in continuous flow. After reaching a steady state, the combined stream was collected into *sat. aq.* NH_4Cl for 30 sec, corresponding to 0.625 mmol of the bromide **8h**. After workup, the crude product was purified *via* column chromatography (pentane:ethyl acetate= 9.8:0.2) to give **14bh** (150 mg, 0.50 mmol, 80%, 99% *ee*) as a colorless oil.

^{1}H-NMR (400 MHz, $CDCl_3$): δ / ppm = 8.03 – 7.87 (m, 2H), 7.19 – 7.13 (m, 2H), 7.10 – 7.00 (m, 4H), 4.60 (q, *J* = 6.8 Hz, 1H), 2.41 (d, *J* = 7.2 Hz, 2H), 1.81 (dt, *J* = 13.3, 6.7 Hz, 1H), 1.51 (d, *J* = 6.9 Hz, 3H), 0.87 (d, *J* = 6.6 Hz, 6H).

^{13}C-NMR (101 MHz, $CDCl_3$): δ / ppm = 198.2, 165.4 (d, *J* = 254.5 Hz), 140.5, 138.5, 132.9 (d, *J* = 3.0 Hz), 131.4 (d, *J* = 9.2 Hz, 2C), 129.8 (2C), 127.4 (2C), 115.5 (d, *J* = 21.9 Hz, 2C), 47.6, 45.0, 30.1, 22.4 (d, *J* = 1.3 Hz), 19.5.

^{19}F-NMR (377 MHz, $CDCl_3$): δ / ppm = −105.8.

IR (Diamond-ATR, neat): $\tilde{\nu}$ / cm^{-1} = 2955, 2929, 2926, 1682, 1596, 1505, 1224, 1155, 1006, 953, 847, 836, 819, 802, 788, 779.

MS (EI, 70 eV): *m/z* (%) = 162 (13), 161 (100), 123 (57), 119 (20), 117 (14), 105 (13), 91 (11).

HRMS (EI): *m/z* calc. for [$C_{19}H_{21}OF$]: 284.1576; found 284.1571.

Optical rotation: $[\alpha]_D^{20}$ = 106 (c 1.05, $CHCl_3$)

Chiral HPLC: 99% *ee*, OD-H column, heptane:*i*-PrOH = 99.5:0.5, 1.0 mL/min, 30 °C.

(2*S*)-1-(3,5-Dimethoxyphenyl)-2-(4-(2-methoxypropyl)phenyl)propan-1-one (14bo)

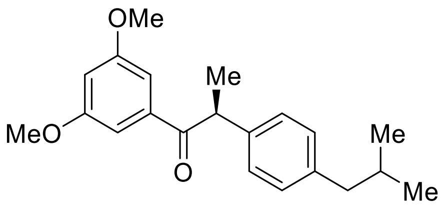

Following **TP2**, solutions of 1-bromo-3,5-dimethoxybenzene (**8o**) (0.25 M, 1.0 equiv) with THF (1.0 equiv) in toluene, *sec*-BuLi (1.4 M, 1.2 equiv) and (*S*)-2-(4-*iso*butylphenyl)-*N,N*-dimethylpropanamide (**13b**) (0.3 M, 1.2 equiv) in toluene were mixed in continuous flow. After reaching a steady state, the combined stream was collected into *sat. aq.* NH_4Cl for 30 sec, corresponding to 0.625 mmol of the bromide **80**. After workup, the crude product was purified *via* column chromatography (pentane:ethyl acetate= 9.9:0.1) to give **14bo** (160 mg, 0.47 mmol, 75%, 99% *ee*) as a colorless oil.

^{1}H-NMR (400 MHz, $CDCl_3$): δ / ppm = 7.20 – 7.15 (m, 2H), 7.12 – 7.04 (m, 4H), 6.56 (t, *J* = 2.3 Hz, 1H), 4.59 (q, *J* = 6.8 Hz, 1H), 3.77 (s, 6H), 2.40 (d, *J* = 7.2 Hz, 2H), 1.81 (hept, *J* = 6.8 Hz, 1H), 1.51 (d, *J* = 6.9 Hz, 3H), 0.87 (dd, *J* = 6.6, 1.3 Hz, 6H).
^{13}C-NMR (101 MHz, $CDCl_3$): δ / ppm = 200.3, 160.8, 140.5, 138.8, 138.7, 129.9 (2C), 127.5 (2C), 106.8 (2C), 105.3, 55.6 (2C), 47.8, 45.1, 30.3, 22.5, 19.7.
IR (Diamond-ATR, neat): $\tilde{\nu}$ / cm^{-1} = 2954, 2930, 1682, 1601, 1592, 1457, 1425, 1354, 1346, 1308, 1293, 1205, 1196, 1155, 1069, 1017, 854.
MS (EI, 70 eV): *m/z* (%) = 165 (100), 137 (24), 122 (10).
HRMS (EI): *m/z* calc. for [$C_{21}H_{26}O_3$]: 326.1877; found 326.1882.
Optical rotation: $[\propto]_D^{20}$ = 84 (c 1.12, $CHCl_3$)
Chiral HPLC: 99% *ee*, OD-H column, heptane:*i*-PrOH = 95:5, 1.0 mL/min, 30 °C.

(*S*)-1-(1-butyl-1H-imidazol-2-yl)-2-(4-isobutylphenyl)propan-1-one (14bs)

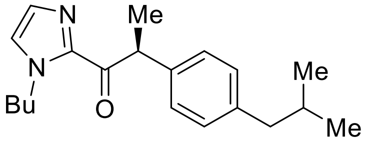

A solution of 1-butyl-1*H*-imidazole (0.25 M, 1.0 equiv.) and TMEDA (1.0 equiv.) in toluene and a solution of *sec*-BuLi in cyclohexane (1.3 M, 1.2 equiv) were prepared. The solutions were pumped from their flasks through a suction needle at flowrate A = 5.0 mL·min^{-1} and flowrate B = 1.15 mL·min^{-1}. The solutions were mixed in a T-mixer (PFA or PTFE, I.D. = 0.5 mm) and the combined stream passed a PTFE reactor tube (i.d = 0.8 mm, Vol_{R1} = 4 mL; residence time: t = 40 sec, T = 25 °C), followed by a PTFE reactor tube (i.d = 0.8 mm, Vol_{R1} = 1 mL; residence time: t = 10 sec, T = –20 °C) for precooling the reaction mixture. A (*S*)-2-(4-*iso*butylphenyl)-*N,N*-dimethylpropanamide (**13b**) solution (0.3 M, 1.2 equiv) in toluene was added *via* a third pump (flowrate C = 5.0 mL·min^{-1}, i.d = 0.8 mm Vol_{pre} = 2.0 mL, T_{pre} = –20 °C, residence $time_{pre:}$ t = 24 sec). The combined stream passed a PTFE reactors tube (i.d = 1.6 mm, Vol_{R2} = 5 mL; residence time: t = 27 sec, T = –20 °C) and the reaction mixture was subsequently quenched with *sat. aq.* NH_4Cl at 0 °C. After extraction with EtOAc, the combined organic phases were dried over Na_2SO_4 and filtrated. After removal of the solvent *in vacuo*, the crude product was purified *via* column chromatography (pentane:ethyl acetate= 9.7:0.3) to give **14bs** (174 mg, 0.56 mmol, 89%, 98% *ee*) as a colorless oil.

^{1}H-NMR (400 MHz, $CDCl_3$): δ / ppm = 7.36 – 7.31 (m, 2H), 7.16 (d, *J* = 1.0 Hz, 1H), 7.12 – 7.02 (m, 3H), 5.29 (q, *J* = 7.1 Hz, 1H), 4.34 (t, *J* = 7.3 Hz, 2H), 2.43 (d, *J* = 7.2 Hz, 2H), 1.84 (dp, *J* = 13.6, 6.8 Hz, 1H), 1.76 – 1.62 (m, 2H), 1.55 (d, *J* = 7.1 Hz, 3H), 1.33 – 1.22 (m, 2H), 0.94 – 0.86 (m, 9H).
^{13}C-NMR (101 MHz, $CDCl_3$): δ / ppm = 193.5, 142.4, 140.3, 138.1, 129.4 (2C), 129.3, 128.1 (2C), 126.3, 48.6, 46.6, 45.2, 33.2, 30.3, 22.5, 19.8, 18.2, 13.7.
IR (Diamond-ATR, neat): $\tilde{\nu}$ / cm^{-1} = 2956, 2929, 1674, 1464, 1404, 1382, 955, 911.
MS (EI, 70 eV): *m/z* (%) = 311 (100), 295 (52), 283 (24), 255 (31), 161 (42), 151 (50), 123 (35), 117 (52), 91 (27).
HRMS (EI): *m/z* calc. for [$C_{20}H_{28}ON_2$]: 312.2197; found 312.2202.
Optical rotation: $[\propto]_D^{20}$ = 39 (c 0.99, $CHCl_3$)
Chiral HPLC: 98% *ee*, OD-H column, heptane:*i*-PrOH = 99:1, 1.0 mL/min, 30 °C.

4-Methoxy-*N,N*-dimethylbenzamide (6d)

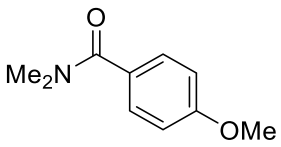

Following **TP3**, solutions of 4-bromoanisole (**8k**) (0.25 M, 1.0 equiv) plus tetramethylurea **4** (0.3 M, 1.2 equiv) in toluene and *sec*-BuLi (1.4 M, 1.2 equiv) were mixed in continuous flow. After reaching a steady state, the combined stream was collected into *sat. aq.* NH_4Cl for 30 sec, corresponding to 0.625 mmol of the bromide **8k**. After workup, the crude product was purified *via* column chromatography (pentane:ethyl acetate= 9.9:0.1) to give **6d** (93.0 mg, 0.52 mmol, 83%) as a colorless oil.

^{1}H-NMR (400 MHz, $CDCl_3$): δ / ppm = 7.42 (d, *J* = 8.7 Hz, 2H), 6.92 (d, *J* = 8.7 Hz, 2H), 3.85 (s, 3H), 3.08 (s, 6H).
^{13}C-NMR (101 MHz, $CDCl_3$): δ / ppm = 171.7, 160.8, 129.3 (2C), 128.4, 113.7 (2C), 55.5.
IR (Diamond-ATR, neat): $\tilde{\nu}$ / cm^{-1} = 3469, 2933, 2839, 1605, 1490, 1440, 1389, 1300, 1246, 1172, 1082, 1024, 840, 764.
MS (EI, 70 eV): *m/z* (%) = 178 (42), 135 (100), 77 (14).
HRMS (EI): *m/z* calc. for [$C_{10}H_{13}O_2N$]: 179.0946; found 179.0939.
m.p:

(1-Butyl-1*H*-imidazol-2-yl)(4-methoxyphenyl)methanone (5a)

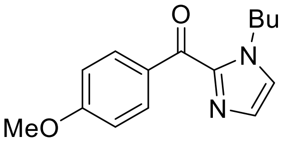

Following **TP3**, solutions of 4-bromoanisole (**8k**) (0.25 M, 1.0 equiv) plus tetramethylurea **4** (0.3 M, 1.2 equiv) in toluene and *sec*-BuLi (1.4 M, 1.2 equiv) were mixed in continuous flow. After reaching a steady state, the combined stream was was poured into an organolithiums

species for 30 sec (corresponding to 0.625 mmol bromide), which was prepared in batch starting from 1-butyl-1H-imidazole (1.00 mmol, 1.6 equiv) plus TMEDA (1.00 mmol, 1.6 equiv) in toluene and *sec*-BuLi (1.4 M, 1.1 mmol, 1.8 equiv) at −20 °C. After workup, the crude product was purified *via* column chromatography (pentane:ethyl acetate= 9:1) to give **5a** (126 mg, 0.49 mmol, 78%) as an orange oil.

^{1}H-NMR (400 MHz, $CDCl_3$): δ / ppm = 8.31 (d, *J* = 9.0 Hz, 2H), 7.21 (d, *J* = 1.0 Hz, 1H), 7.12 (d, *J* = 1.0 Hz, 1H), 6.98 – 6.93 (m, 2H), 4.55 – 4.28 (m, 2H), 3.88 (s, 3H), 1.91 – 1.74 (m, 2H), 1.47 – 1.29 (m, 2H), 0.95 (t, *J* = 7.4 Hz, 3H).
^{13}C-NMR (101 MHz, $CDCl_3$): δ / ppm = 183.0, 163.5, 143.1, 133.4 (2C), 130.5, 129.1, 125.3, 113.6 (2C), 55.6, 48.7, 33.5, 20.0, 13.8.
IR (Diamond-ATR, neat): $\tilde{\nu}$ / cm^{-1} = 2958, 2932, 1636, 1596, 1461, 1420, 1396, 1381, 1309, 1251, 1180, 1159, 1144, 1029, 929, 903, 843, 776.
MS (EI, 70 eV): *m/z* (%) = 257 (57), 229 (44), 173 (32), 135 (70), 123 (24), 121 (100), 77 (34).
HRMS (EI): *m/z* calc. for [$C_{15}H_{18}O_2N_2$]: 258.1368; found 258.1363.
m.p:

1-(4-Methoxyphenyl)-2-phenylethan-1-one (5b)

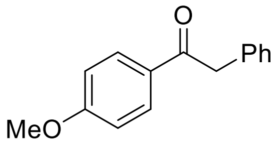

Following **TP3**, solutions of 4-bromoanisole (**8k**) (0.25 M, 1.0 equiv) plus tetramethylurea **4** (0.3 M, 1.2 equiv) in toluene and *sec*-BuLi (1.4 M, 1.2 equiv) were mixed in continuous flow. After reaching a steady state, the combined stream was was poured into an organolithiums species for 30 sec (corresponding to 0.625 mmol bromide), which was prepared in batch starting from toluene plus TMEDA (1.00 mmol, 1.6 equiv) and *sec*-BuLi (1.4 M, 1.1 mmol, 1.8 equiv) at −20 °C. After workup, the crude product was purified *via* column chromatography (pentane:ethyl acetate= 9.3:0.7) to give **5b** (109 mg, 0.48 mmol, 77%) as a yellow solid.

^{1}H-NMR (400 MHz, $CDCl_3$): δ / ppm = 8.04 – 7.96 (m, 2H), 7.39 – 7.22 (m, 5H), 6.99 – 6.90 (m, 2H), 4.25 (s, 2H), 3.87 (s, 3H).

^{13}C-NMR (101 MHz, $CDCl_3$): δ / ppm = 196.3, 163.6, 135.1, 131.0 (2C), 129.7, 129.5 (2C), 128.7 (2C), 126.9, 113.9 (2C), 55.6, 45.4.
IR (Diamond-ATR, neat): $\tilde{\nu}$ / cm^{-1} = 3028, 2932, 2838, 1676, 1670, 1667, 1598, 1574, 1509, 1257, 1223, 1167, 1029, 990, 828, 727, 705, 696.
MS (EI, 70 eV): *m/z* (%) = 135 (100), 77 (23).
HRMS (EI): *m/z* calc. for [$C_{15}H_{14}O_2$]: 226.0994; found 226.0984.
m.p: 71.3-71.8 °C.

1-(4-Methoxyphenyl)pentan-1-one (5c)

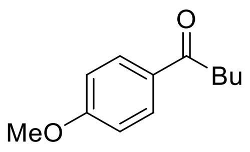

Following **TP3**, solutions of 4-bromoanisole (**8k**) (0.25 M, 1.0 equiv) plus tetramethylurea **4** (0.3 M, 1.2 equiv) in toluene and *sec*-BuLi (1.4 M, 1.2 equiv) were mixed in continuous flow. After reaching a steady state, the combined stream was was poured into a *n*-BuLi solution (1.7 M, 1.0 mmol, 1.6 equiv) for 30 sec (corresponding to 0.625 mmol bromide),. After workup, the crude product was purified *via* column chromatography (pentane:ethyl acetate= 9.9:0.1) to give **5c** (84.0 mg, 0.44 mmol, 70%) as a colorless oil.

^{1}H-NMR (400 MHz, $CDCl_3$): δ / ppm = 8.00 – 7.85 (m, 2H), 6.98 – 6.85 (m, 2H), 3.86 (s, 3H), 3.03 – 2.82 (m, 2H), 1.76 – 1.64 (m, 2H), 1.47 – 1.32 (m, 2H), 0.95 (t, *J* = 7.4 Hz, 3H).
^{13}C-NMR (101 MHz, $CDCl_3$): δ / ppm = 199.3, 163.3, 130.3 (2C), 113.7 (2C), 55.5, 38.0, 26.8, 22.6, 14.0.
IR (Diamond-ATR, neat): $\tilde{\nu}$ / cm^{-1} = 2956, 2932, 1674, 1599, 1509, 1460, 1417, 1307, 1254, 1211, 1168, 1029, 839.
MS (EI, 70 eV): *m/z* (%) = 150 (48), 135 (100).
HRMS (EI): *m/z* calc. for [$C_{12}H_{16}O_2$]: 192.1150; found 192.1145.

(1-Methyl-1H-benzo[*d*]imidazol-2-yl)(4-(methylthio)phenyl)methanone (5d)

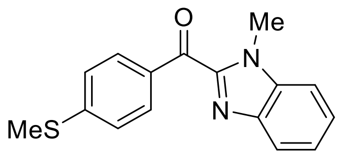

Following **TP3**, solutions of 4-bromothioanisole (**8a**) (0.25 M, 1.0 equiv) plus tetramethylurea **4** (0.3 M, 1.2 equiv) in toluene and *sec*-BuLi (1.4 M, 1.2 equiv) were mixed in continuous flow. After reaching a steady state, the combined stream was was poured into an organolithiums species for 30 sec (corresponding to 0.625 mmol bromide), which was prepared in batch starting from 1-methyl-1H-benzo[*d*]imidazole (1.00 mmol, 1.6 equiv) plus TMEDA (1.00 mmol, 1.6 equiv) in toluene and *sec*-BuLi (1.4 M, 1.1 mmol, 1.8 equiv) at –20 °C. After workup, the crude product was purified *via* column chromatography (pentane:ethyl acetate= 9.2:0.8) to give **5d** (138 mg, 0.49 mmol, 79%) as a yellow oil.

^{1}H-NMR (400 MHz, CDCl$_3$): δ / ppm = 8.36 – 8.26 (m, 2H), 7.92 (dt, *J* = 8.1, 0.9 Hz, 1H), 7.52 – 7.44 (m, 2H), 7.41 – 7.32 (m, 3H), 4.15 (s, 3H), 2.55 (s, 3H).

^{13}C-NMR (101 MHz, CDCl$_3$): δ / ppm = 185.3, 147.0, 147.0, 142.0, 136.7, 133.2, 131.8 (2C), 125.7, 125.0 (2C), 123.7, 122.1, 110.5, 32.4, 14.9.

IR (Diamond-ATR, neat): $\tilde{\nu}$ / cm^{-1} = 3056, 2919, 1641, 1585, 1477, 1456, 1400, 1390, 1335, 1266, 1234, 1186, 1091, 943, 897, 767, 743.

MS (EI, 70 eV): *m/z* (%) = 281 (100), 238 (23), 207 (14), 206 (16), 151 (14).

HRMS (EI): *m/z* calc. for $[C_{16}H_{13}ON_2S]^+$: 281.0743; found 281.0743 $[M-H]^+$.

Benzo[*b*]thiophen-2-yl(4-(methylthio)phenyl)methanone (5e)

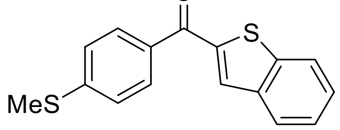

Following **TP3**, solutions of 4-bromothioanisole (**8a**) (0.25 M, 1.0 equiv) plus tetramethylurea **4** (0.3 M, 1.2 equiv) in toluene and *sec*-BuLi (1.4 M, 1.2 equiv) were mixed in continuous flow. After reaching a steady state, the combined stream was was poured into an organolithiums species for 30 sec (corresponding to 0.625 mmol bromide), which was prepared in batch starting from benzo[*b*]thiophene (1.00 mmol, 1.6 equiv) plus TMEDA (1.00 mmol, 1.6 equiv) in toluene and *sec*-BuLi (1.4 M, 1.1 mmol, 1.8 equiv) at –20 °C. After workup, the crude product was purified *via* column chromatography (pentane:ethyl acetate= 9.5:0.5) to give **5e** (123 mg, 0.43 mmol, 69%) as a yellow solid.

^{1}H-NMR (400 MHz, $CDCl_3$): δ / ppm = 7.94 – 7.84 (m, 5H), 7.52 – 7.40 (m, 2H), 7.39 – 7.33 (m, 2H), 2.57 (s, 3H).
^{13}C-NMR (101 MHz, $CDCl_3$): δ / ppm = 188.7, 145.6, 143.3, 142.7, 139.2, 134.1, 131.7, 130.0 (2C), 127.5, 126.1, 125.3 (2C), 125.2, 123.1, 15.1.
IR (Diamond-ATR, neat): $\tilde{\nu}$ / cm^{-1} = 3053, 2917, 1621, 1589, 1513, 1296, 754, 742, 727, 723.
MS (EI, 70 eV): *m/z* (%) = 284 (77), 281 (38), 237 (40), 225 (26), 207 (100), 161 (28), 151 (54).
HRMS (EI): *m/z* calc. for [$C_{16}H_{12}OS_2$]: 284.0330; found 283.0324.
m.p: 124.6-126.0 °C.

(4-(Methylthio)phenyl)(4-octylthiophen-2-yl)methanone (5f)

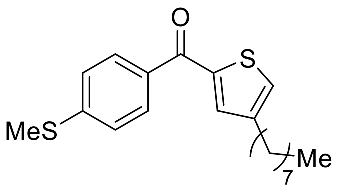

Following **TP3**, solutions of 4-bromothioanisole (**8a**) (0.25 M, 1.0 equiv) plus tetramethylurea **4** (0.3 M, 1.2 equiv) in toluene and *sec*-BuLi (1.4 M, 1.2 equiv) were mixed in continuous flow. After reaching a steady state, the combined stream was was poured into an organolithiums species for 30 sec (corresponding to 0.625 mmol bromide), which was prepared in batch starting from 3-octylthiophene (1.00 mmol, 1.6 equiv) plus TMEDA (1.00 mmol, 1.6 equiv) in toluene and *sec*-BuLi (1.4 M, 1.1 mmol, 1.8 equiv) at –20 °C. After workup, the crude product

was purified *via* column chromatography (pentane:ethyl acetate= 9.8:0.2) to give **5f** (170 mg, 0.44 mmol, 70%) as a yellow oil.

^{1}H-NMR (400 MHz, $CDCl_3$): δ / ppm = 7.81 (d, *J* = 8.5 Hz, 2H), 7.47 (d, *J* = 1.4 Hz, 1H), 7.34 – 7.29 (m, 3H), 2.62 (t, *J* = 7.7 Hz, 2H), 2.55 (s, 3H), 1.62 (p, *J* = 7.4 Hz, 2H), 1.29 (d, *J* = 14.3 Hz, 10H), 0.92 – 0.80 (m, 3H).
^{13}C-NMR (101 MHz, $CDCl_3$): δ / ppm = 187.3, 144.9, 144.5, 143.2, 135.6, 134.6, 129.9 (2C), 129.3, 125.2 (2C), 32.0, 30.6, 30.5, 29.5, 29.4, 29.4, 22.8, 15.1, 14.2.
IR (Diamond-ATR, neat): $\tilde{\nu}$ / cm^{-1} = 2922, 2853, 1629, 1589, 1418, 1290, 1185, 1087, 855.
MS (EI, 70 eV): *m/z* (%) = 346 (16), 248 (41), 247 (14), 151 (100).
HRMS (EI): *m/z* calc. for [$C_{20}H_{26}OS_2$]: 346.1425; found 346.1420.

Chiral HPLC Analysis

(*R*/*S*)-2-(6-Methoxynaphthalen-2-yl)-*N,N*-dimethylpropanamide (13a)

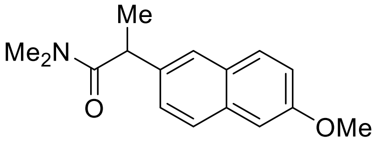

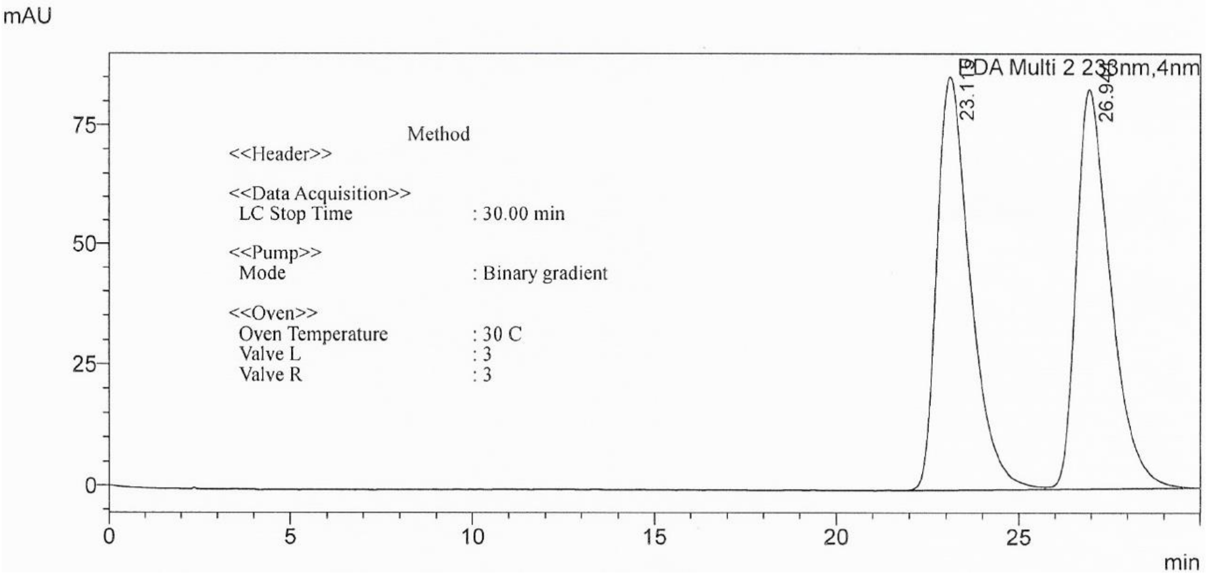

PDA Ch2 233nm

Peak#	Ret. Time	Area	Height	Area%
1	23.115	5446727	85898	49.990
2	26.944	5448902	83150	50.010
Total		10895629	169048	100.000

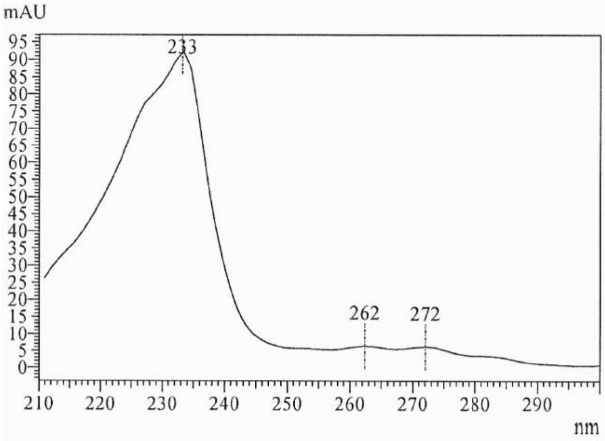

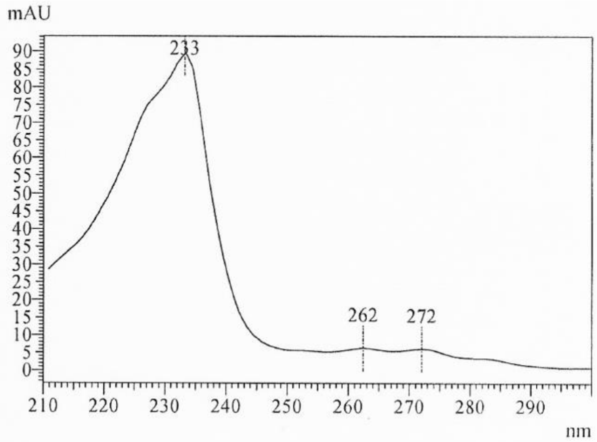

(*S*)-2-(6-Methoxynaphthalen-2-yl)-*N*,*N*-dimethylpropanamide (13a)

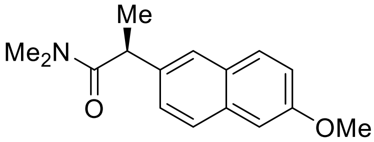

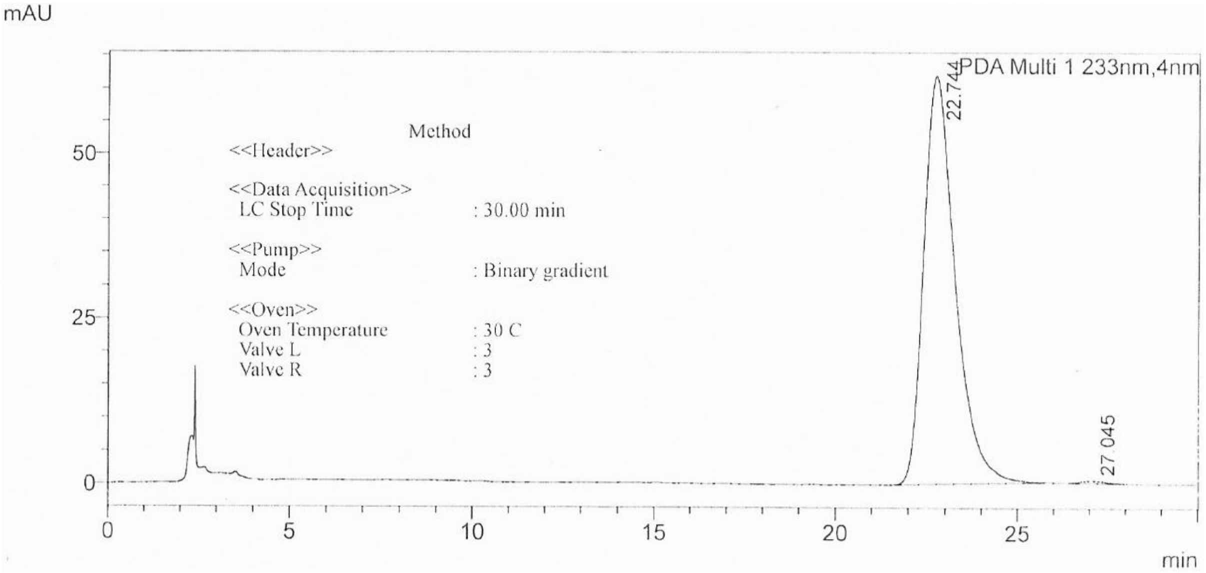

PDA Ch1 233nm

Peak#	Ret. Time	Area	Height	Area%
1	22.744	3706565	61766	99.513
2	27.045	18158	355	0.487
Total		3724723	62121	100.000

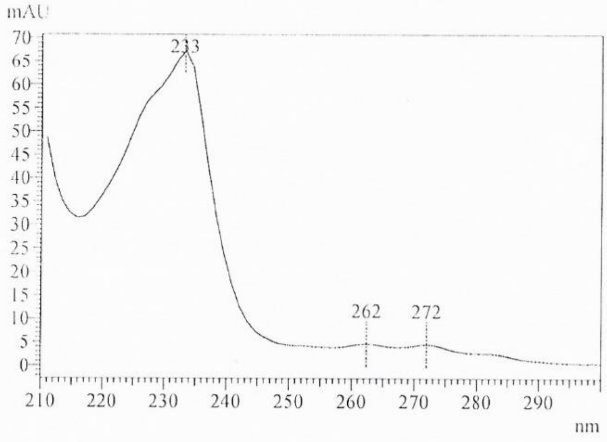

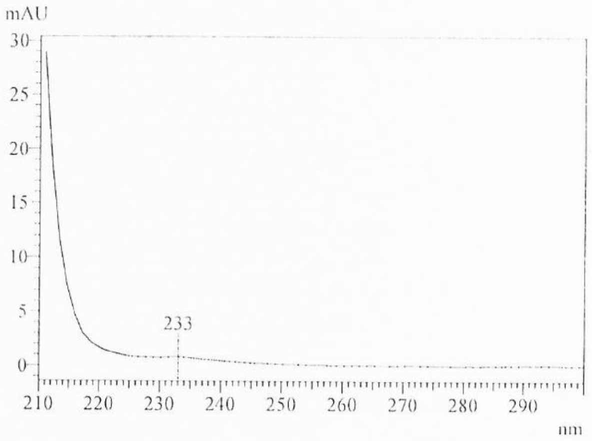

(*R*/*S*)-2-(4-*Iso*butylphenyl)-*N,N*-dimethylpropanamide (13b)

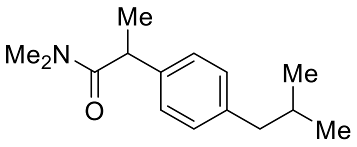

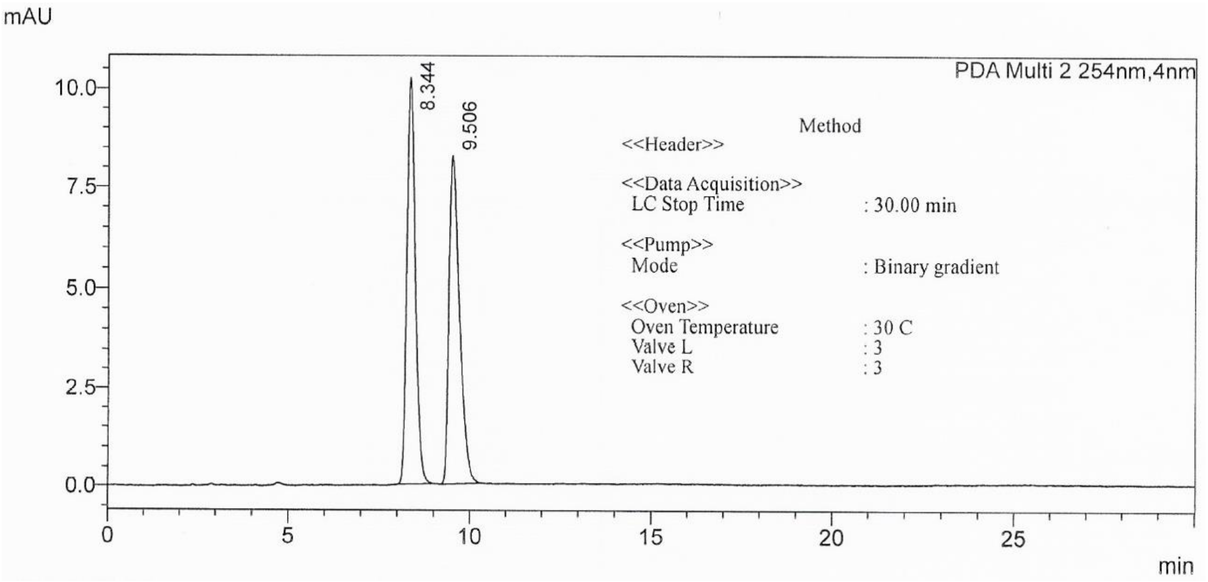

PDA Ch2 254nm

Peak#	Ret. Time	Area	Height	Area%
1	8.344	166570	10232	49.768
2	9.506	168125	8248	50.232
Total		334695	18480	100.000

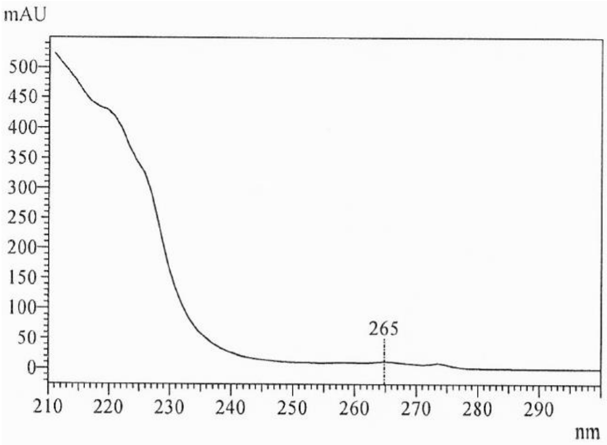

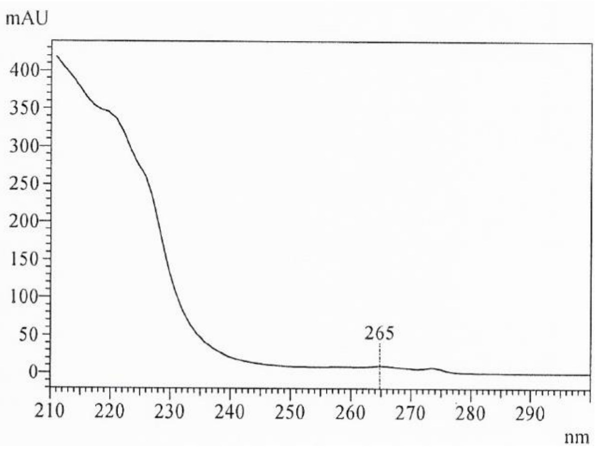

(*S*)-2-(4-*Iso*butylphenyl)-*N,N*-dimethylpropanamide (13b)

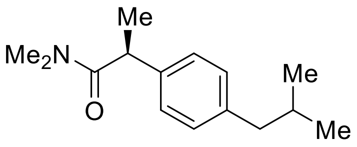

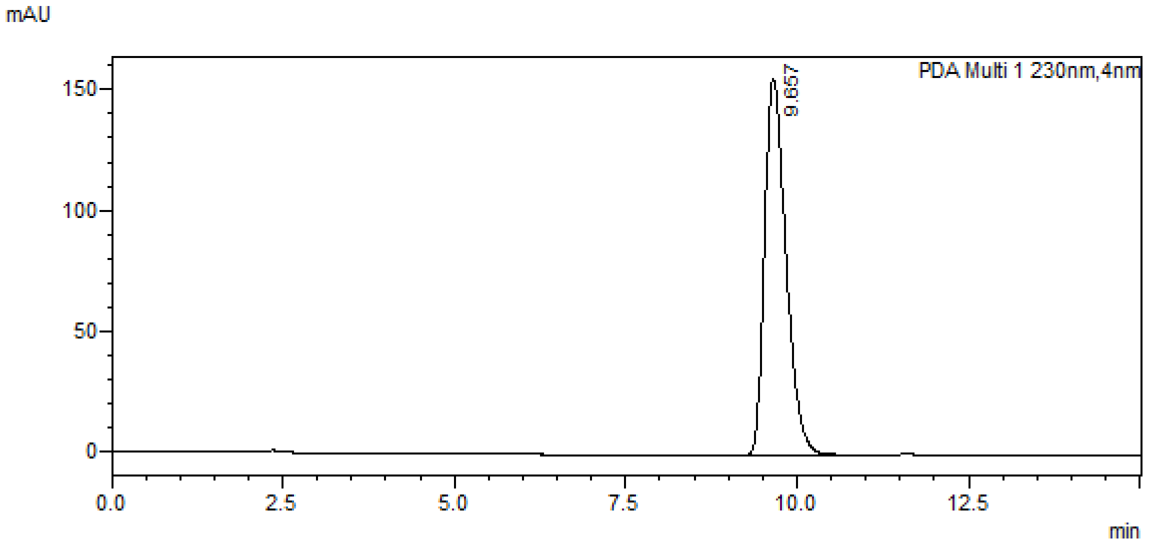

PDA Ch1 230nm

Peak#	Ret. Time	Area	Height	Area%
1	9.657	3409172	156051	100.000
Total		3409172	156051	100.000

UV Spectrum

Peak# : 1 Retention Time : 9.657 min

Lambda max : 259/488/433/390/604

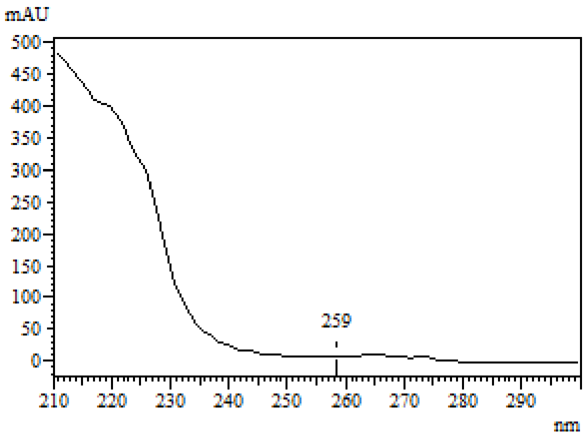

(*R/S*)-1-(3-Bromophenyl)-2-(6-methoxynaphthalen-2-yl)propan-1-one (14ac)

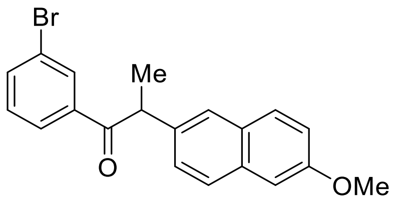

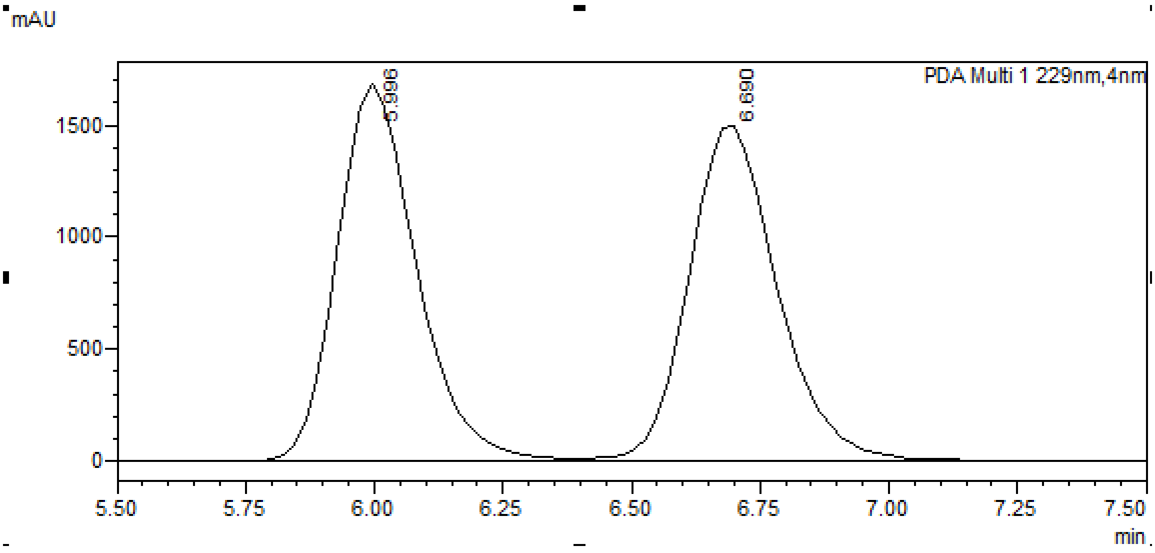

PDA Ch1 229nm

Peak#	Ret. Time	Area	Height	Area%
1	5.996	18093980	1685217	50.016
2	6.690	18082376	1495748	49.984
Total		36176356	3180965	100.000

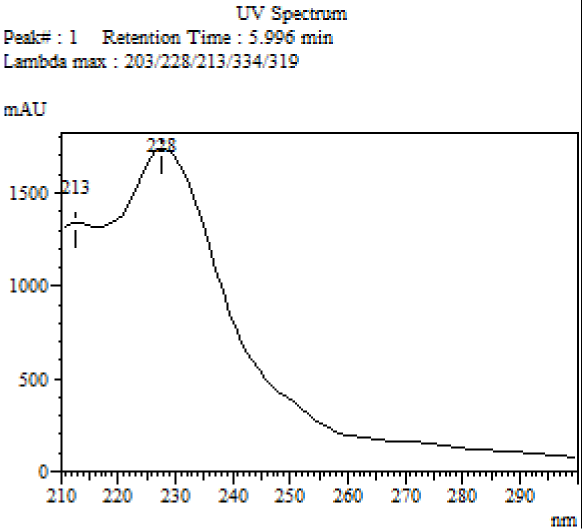

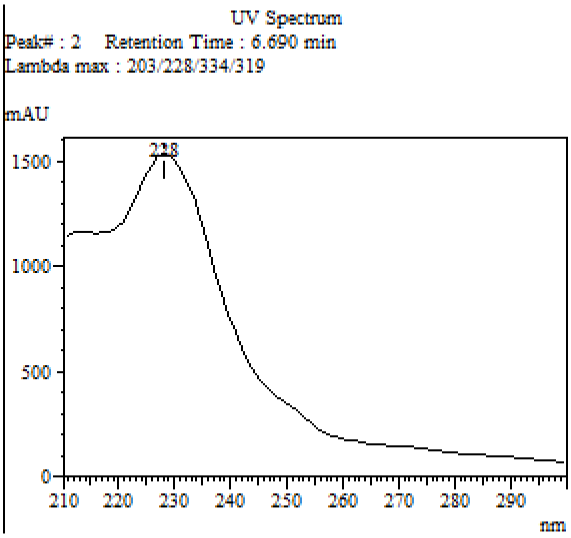

(*S*)-1-(3-Bromophenyl)-2-(6-methoxynaphthalen-2-yl)propan-1-one (14ac)

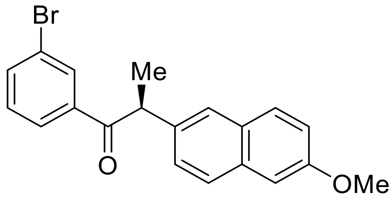

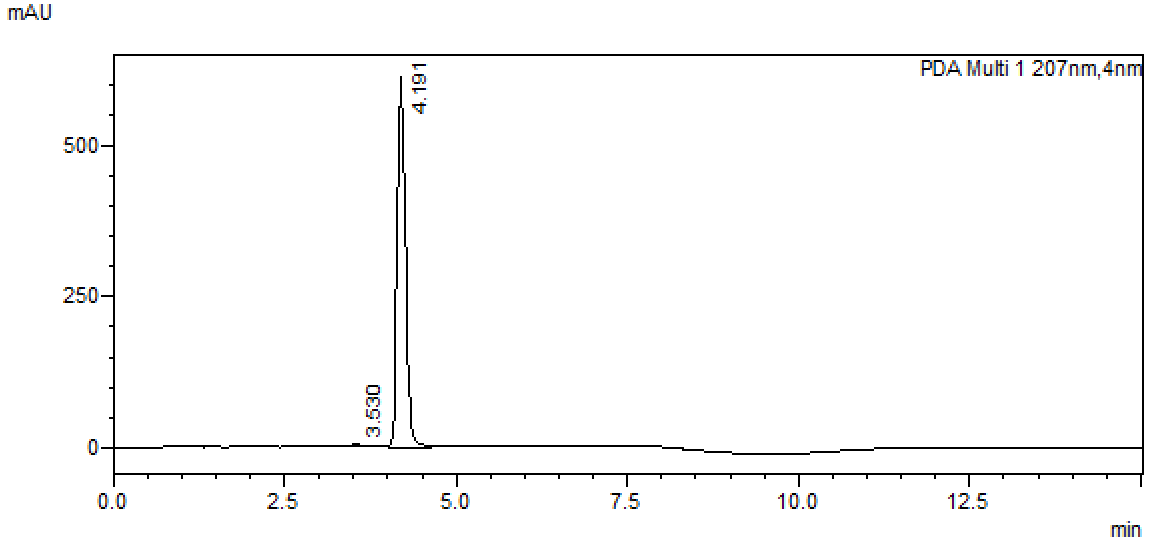

PDA Ch1 207nm

Peak#	Ret. Time	Area	Height	Area%
1	3.530	32158	4156	0.622
2	4.191	5135829	613076	99.378
Total		5167987	617232	100.000

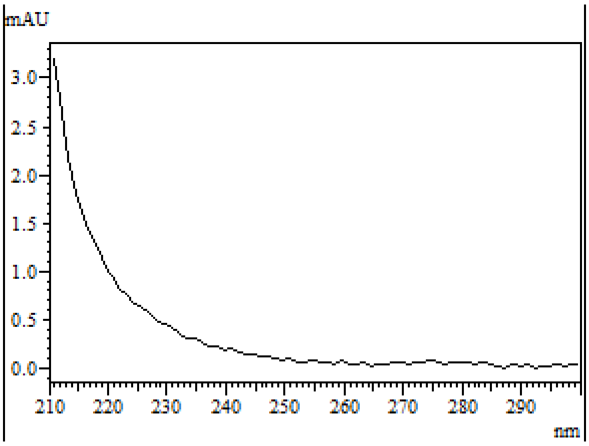

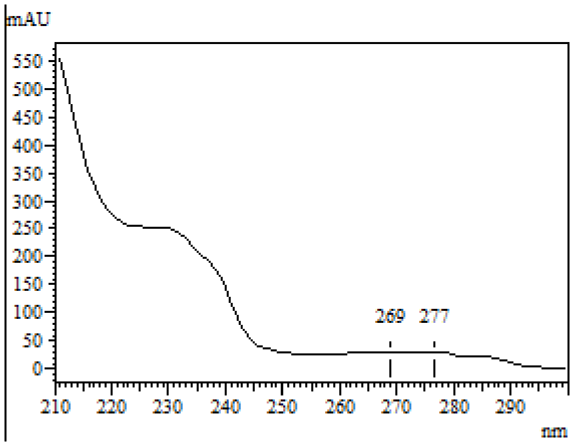

(*R*/*S*)-1-(3-(Diethoxymethyl)phenyl)-2-(6-methoxynaphthalen-2-yl)propan-1-one (14am)

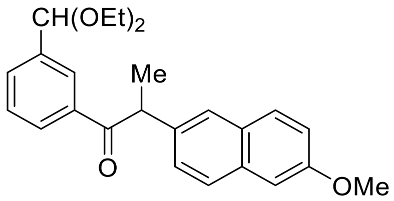

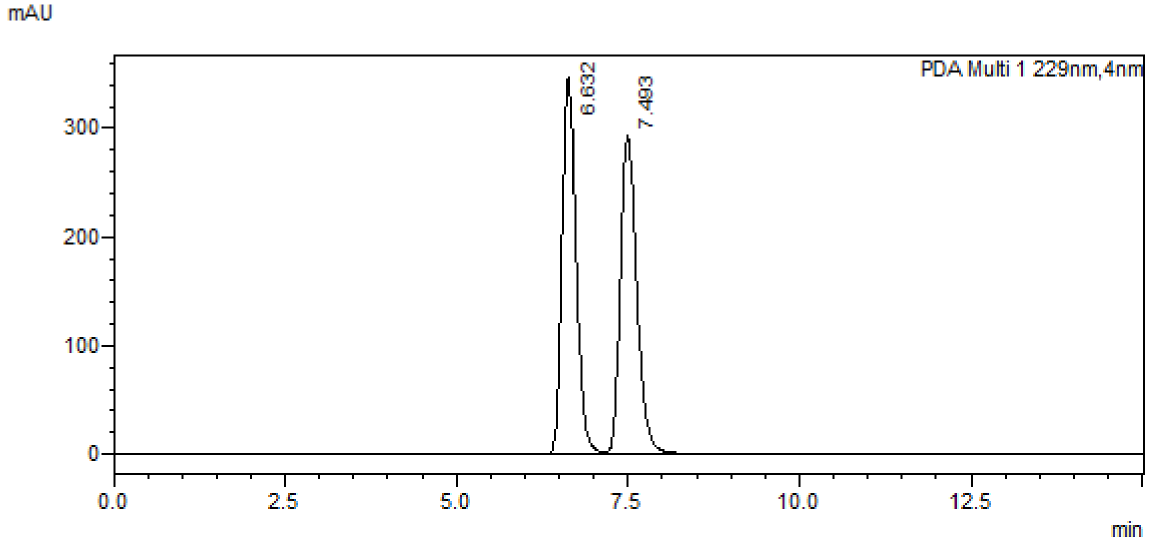

PDA Ch1 229nm

Peak#	Ret. Time	Area	Height	Area%
1	6.632	4870437	347024	49.932
2	7.493	4883779	292491	50.068
Total		9754217	639515	100.000

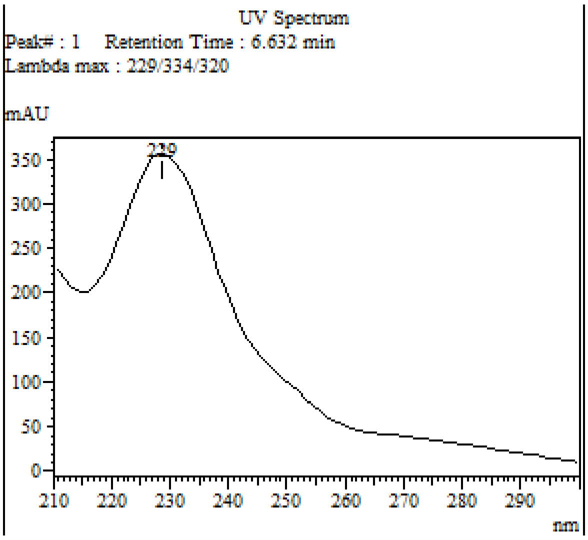

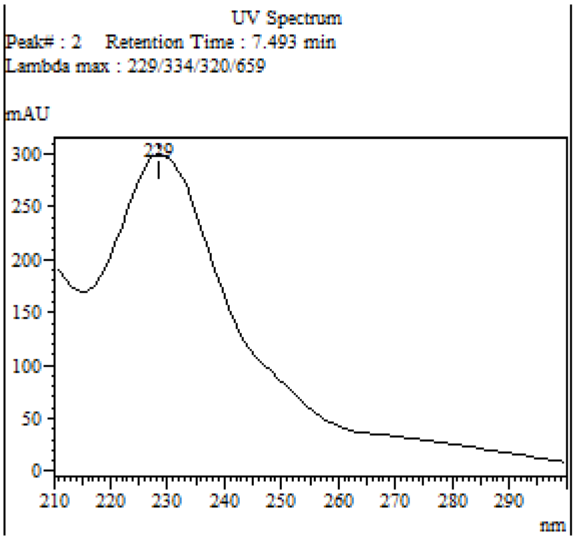

(*S*)-1-(3-(Diethoxymethyl)phenyl)-2-(6-methoxynaphthalen-2-yl)propan-1-one (14am)

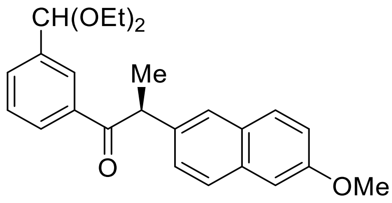

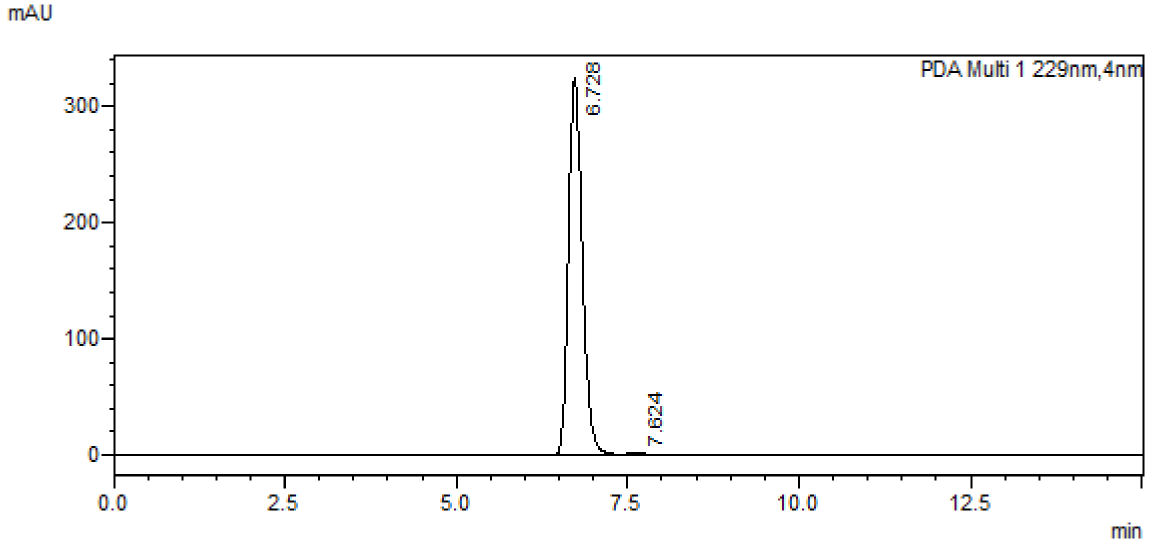

PDA Ch1 229nm

Peak#	Ret. Time	Area	Height	Area%
1	6.728	4593107	324655	99.517
2	7.624	22304	1345	0.483
Total		4615410	326000	100.000

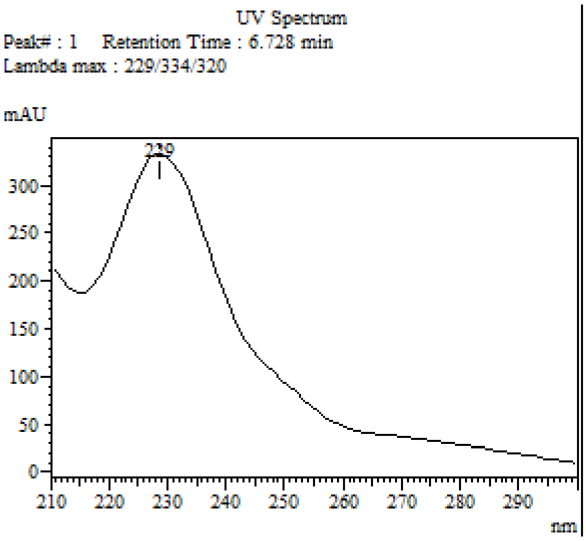

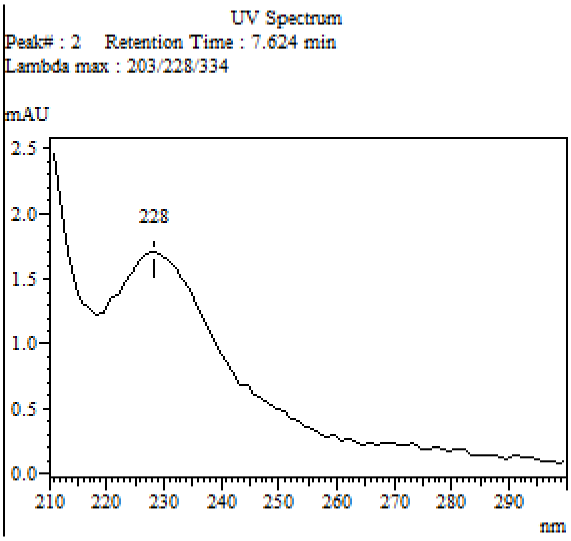

(*R*/*S*)-1-(3-Fluorophenyl)-2-(6-methoxynaphthalen-2-yl)propan-1-one (14an)

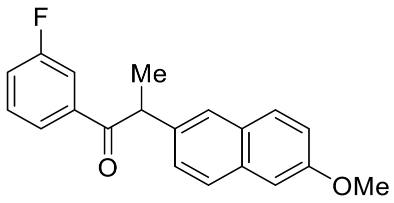

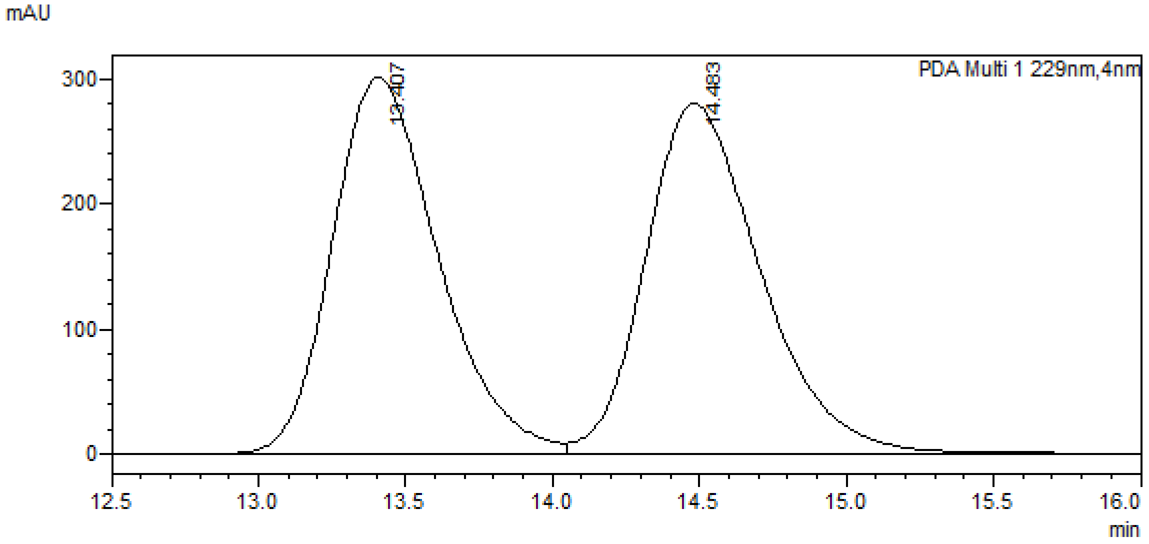

PDA Ch1 229nm

Peak#	Ret. Time	Area	Height	Area%
1	13.407	7540672	300842	49.567
2	14.483	7672267	279782	50.433
Total		15212940	580625	100.000

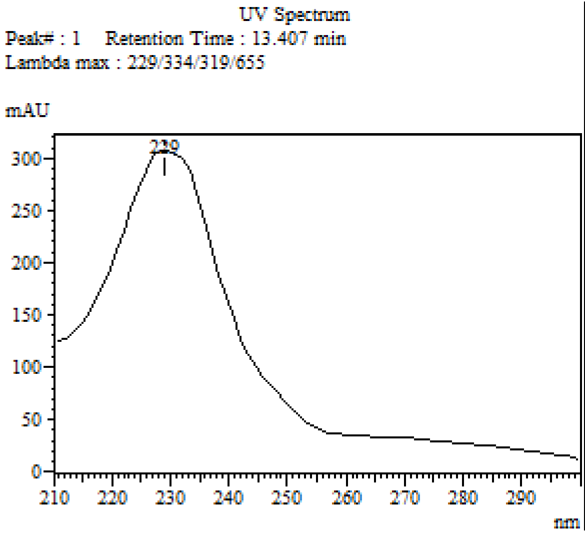

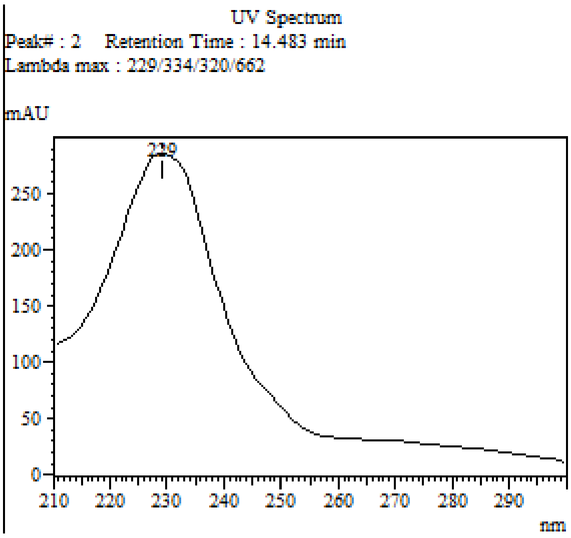

(*S*)-1-(3-Fluorophenyl)-2-(6-methoxynaphthalen-2-yl)propan-1-one (14an)

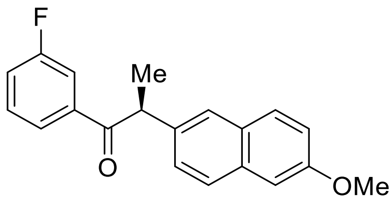

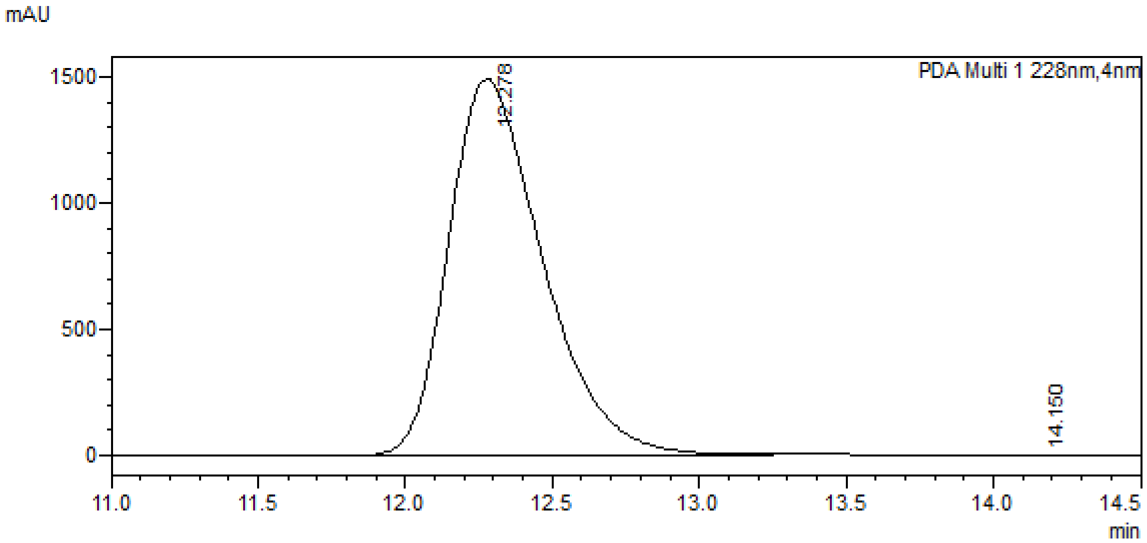

PDA Ch1 228nm

Peak#	Ret. Time	Area	Height	Area%
1	12.278	33481637	1494172	99.733
2	14.150	89620	2669	0.267
Total		33571257	1496841	100.000

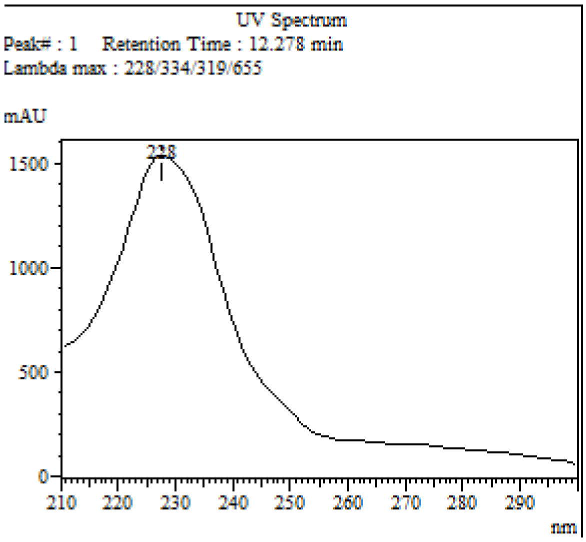

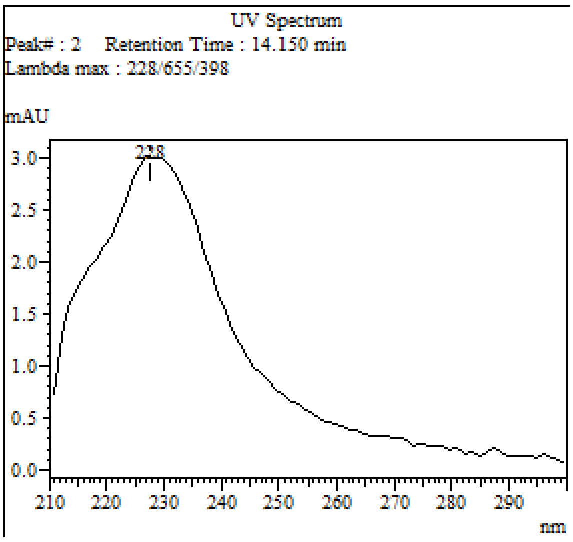

(*R*/*S*)-1-(4-Fluorophenyl)-2-(4-(2-methoxypropyl)phenyl)propan-1-one (14bh)

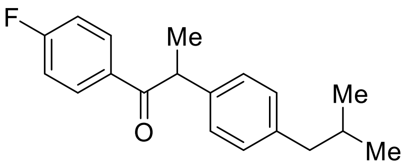

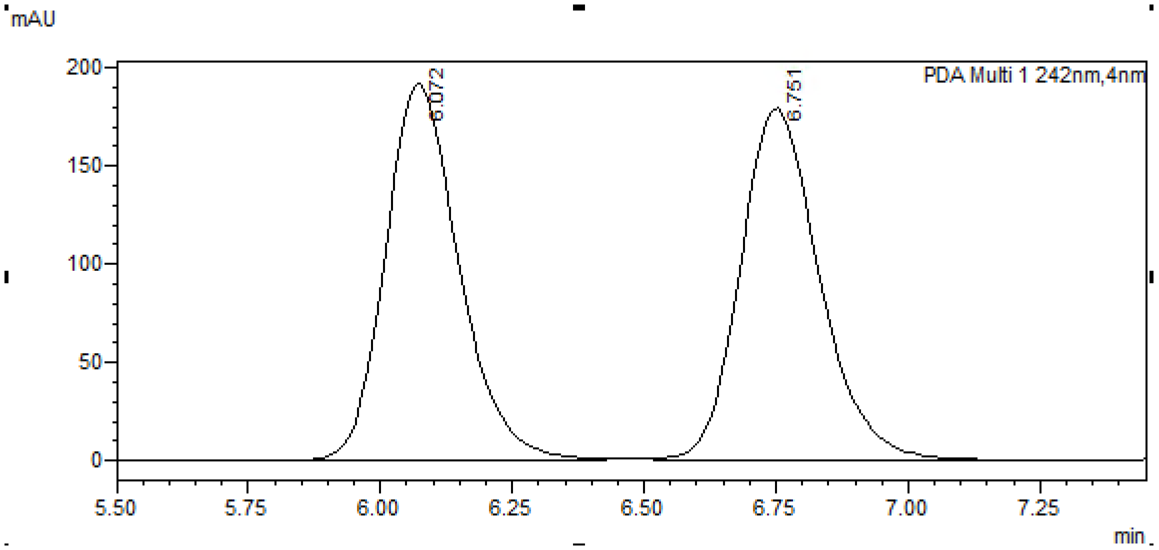

PDA Ch1 242nm

Peak#	Ret. Time	Area	Height	Area%
1	6.072	1880990	192205	49.908
2	6.751	1887911	179719	50.092
Total		3768901	371925	100.000

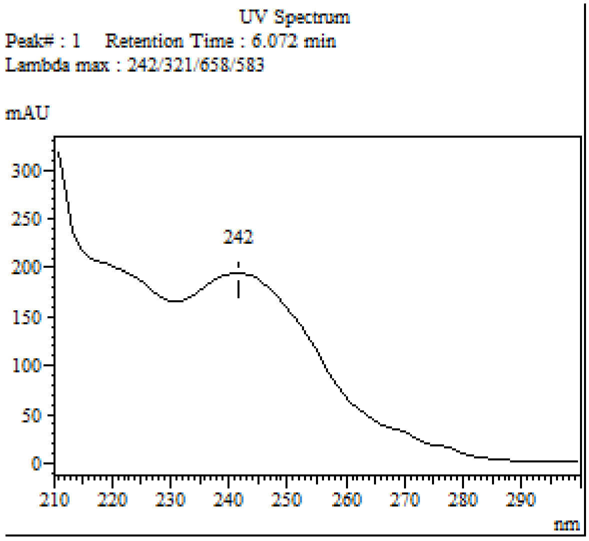

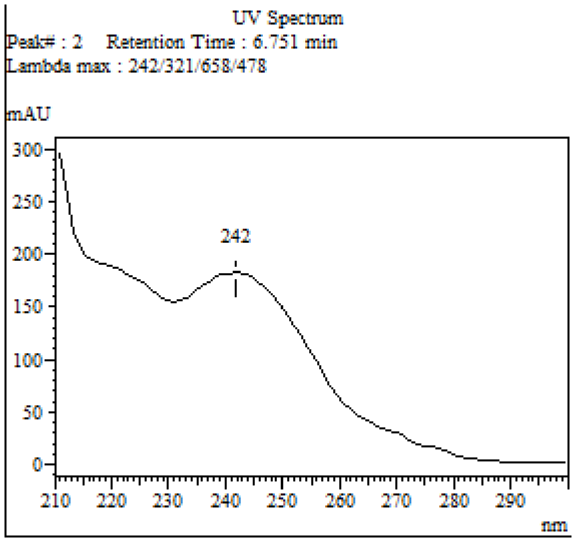

(*S*)-1-(4-Fluorophenyl)-2-(4-(2-methoxypropyl)phenyl)propan-1-one (14bh)

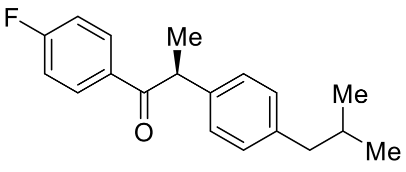

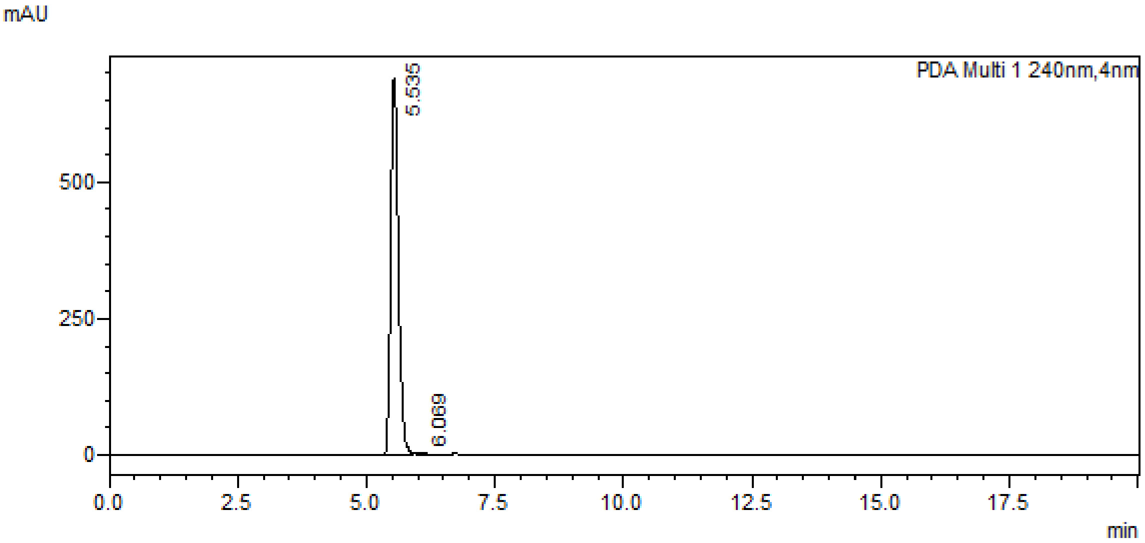

PDA Ch1 240nm

Peak#	Ret. Time	Area	Height	Area%
1	5.535	7226041	690405	99.335
2	6.069	48347	4292	0.665
Total		7274388	694696	100.000

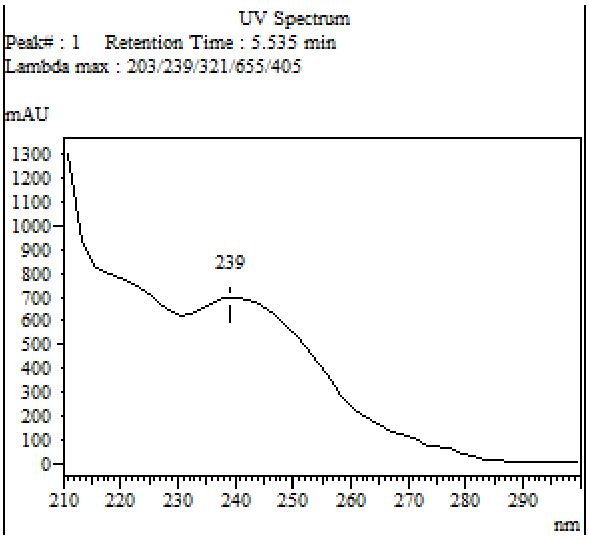

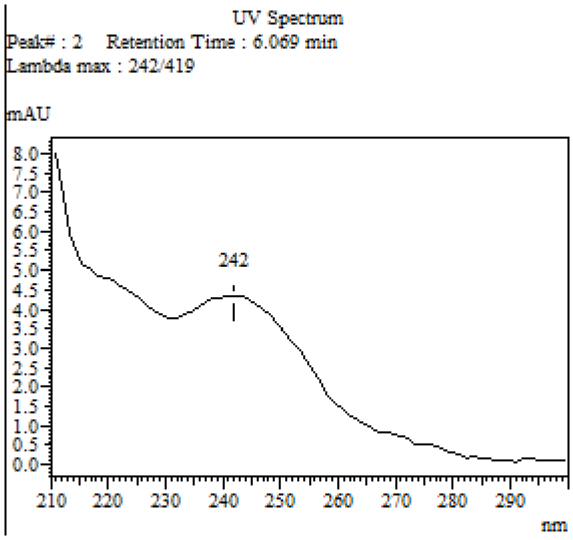

(*R*/*S*)-1-(3,5-Dimethoxyphenyl)-2-(4-(2-methoxypropyl)phenyl)propan-1-one (14bo)

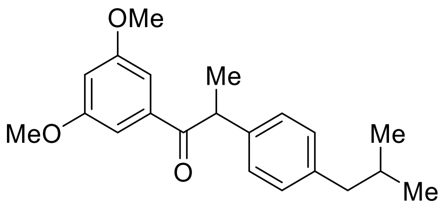

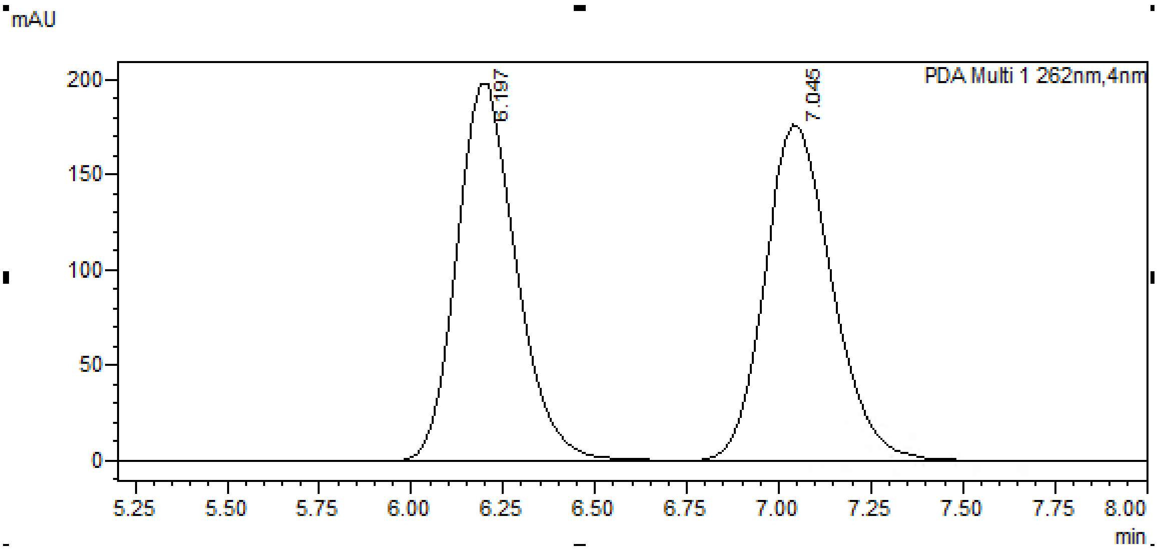

PDA Ch1 262nm

Peak#	Ret. Time	Area	Height	Area%
1	6.197	2242498	198179	49.966
2	7.045	2245589	176629	50.034
Total		4488087	374807	100.000

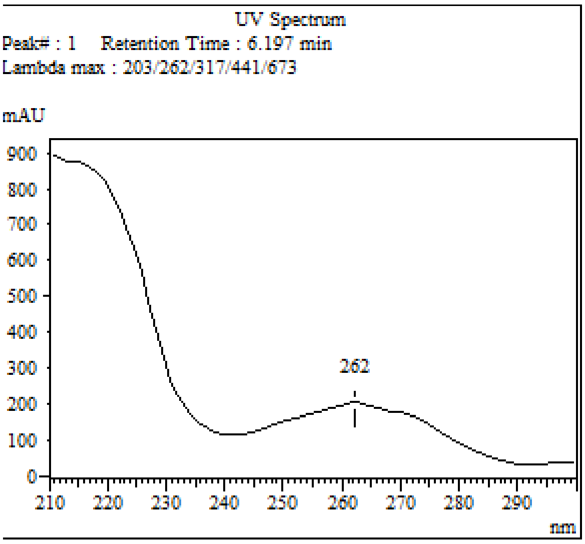

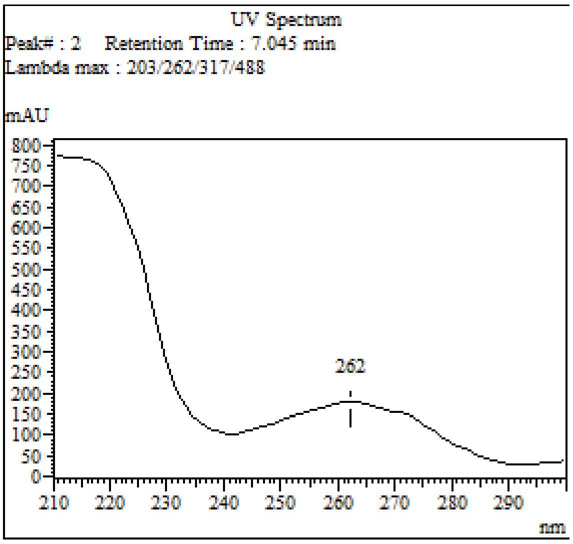

(*S*)-1-(3,5-Dimethoxyphenyl)-2-(4-(2-methoxypropyl)phenyl)propan-1-one (14bo)

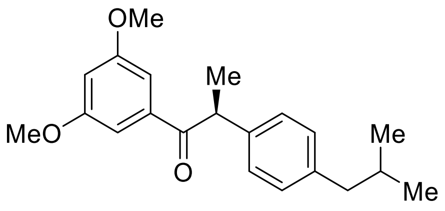

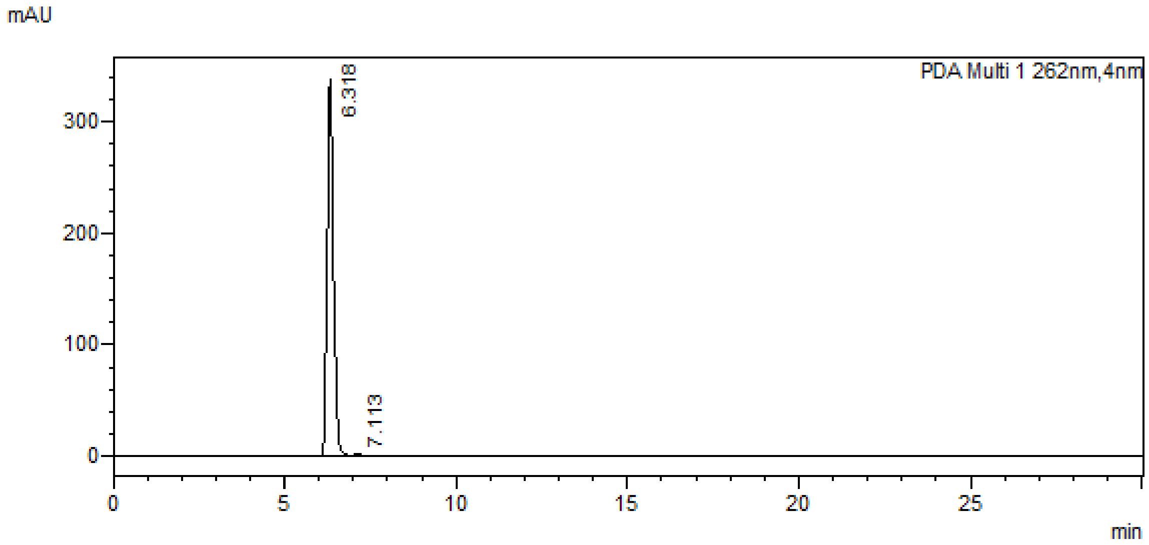

PDA Ch1 262nm				
Peak#	Ret. Time	Area	Height	Area%
1	6.318	4360137	338124	99.585
2	7.113	18160	1282	0.415
Total		4378297	339406	100.000

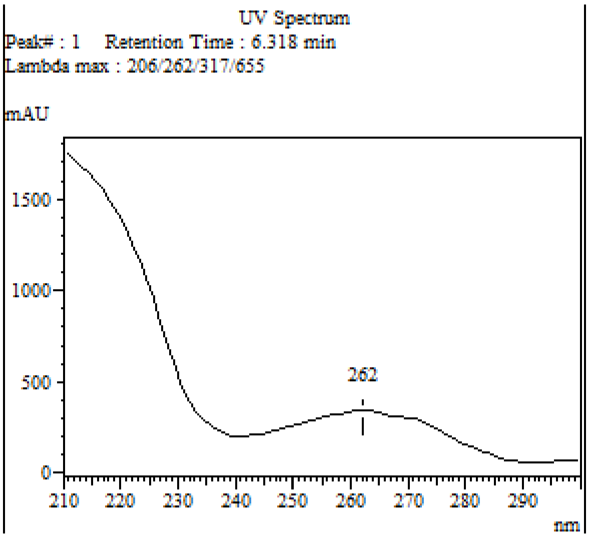

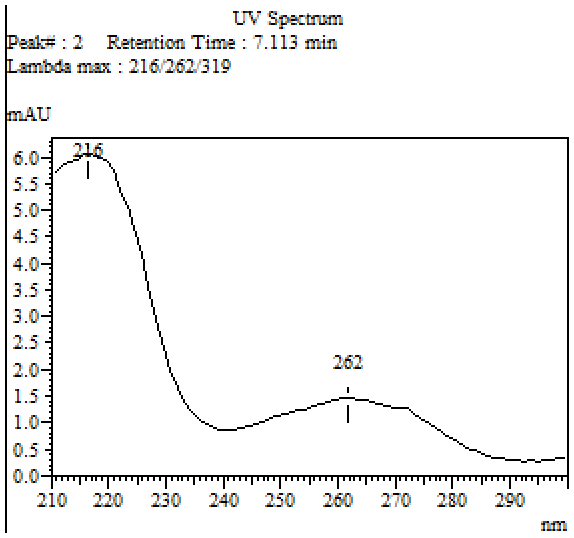

(*R*/*S*)-1-(1-butyl-1H-imidazol-2-yl)-2-(4-isobutylphenyl)propan-1-one (14bs)

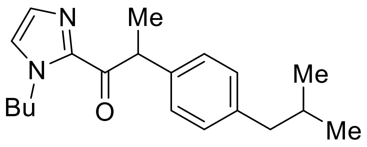

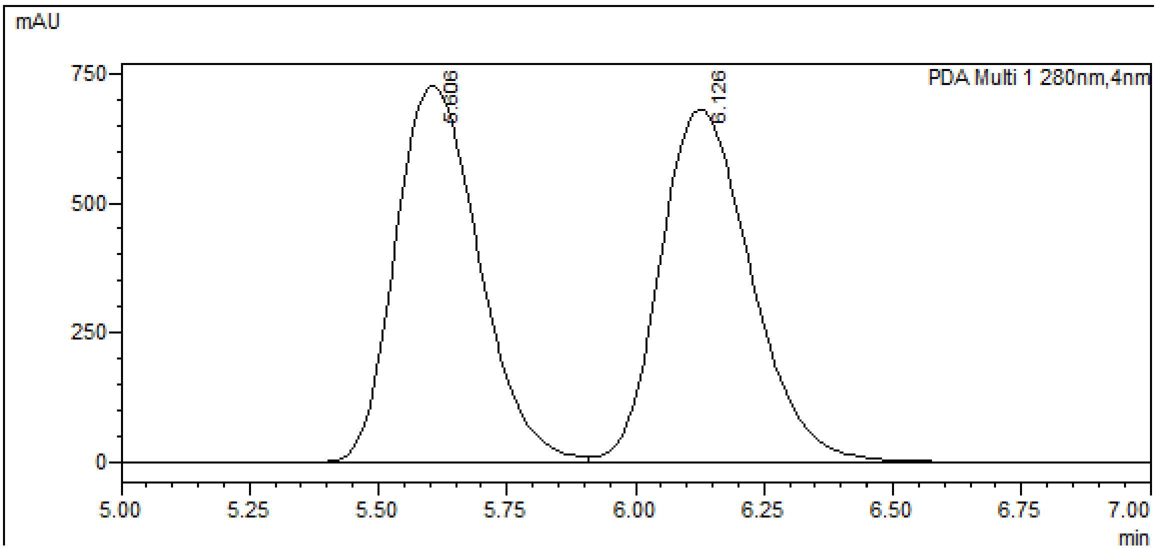

PDA Ch1 280nm

Peak#	Ret. Time	Area	Height	Area%
1	5.606	8267332	726798	49.442
2	6.126	8454054	680959	50.558
Total		16721385	1407757	100.000

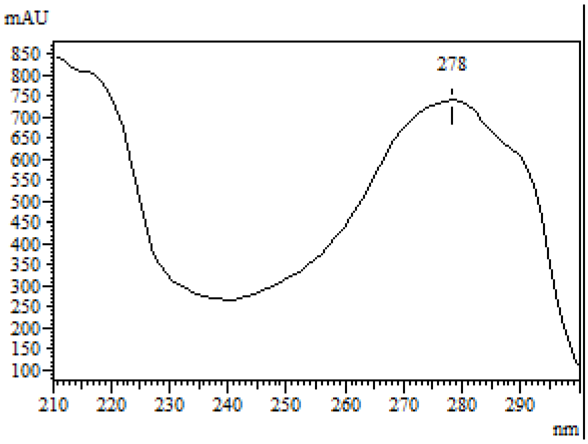

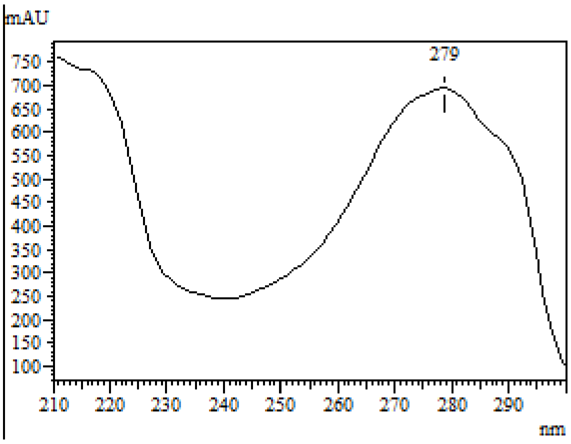

(*S*)-1-(1-butyl-1H-imidazol-2-yl)-2-(4-isobutylphenyl)propan-1-one (14bs)

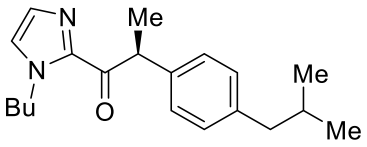

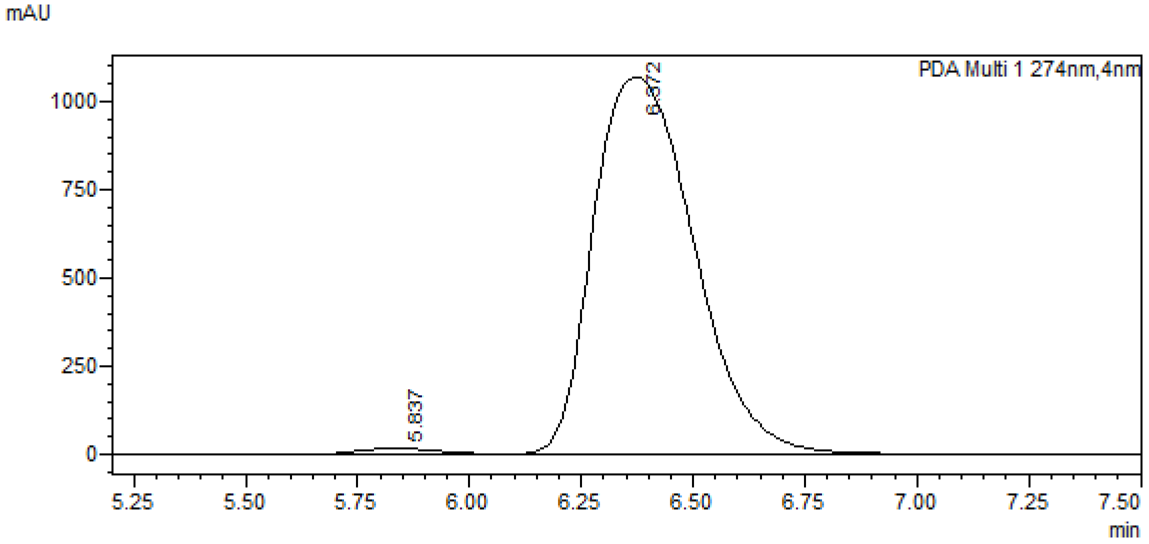

PDA Ch1 274nm

Peak#	Ret. Time	Area	Height	Area%
1	5.837	210373	16982	1.237
2	6.372	16802645	1068054	98.763
Total		17013018	1085036	100.000

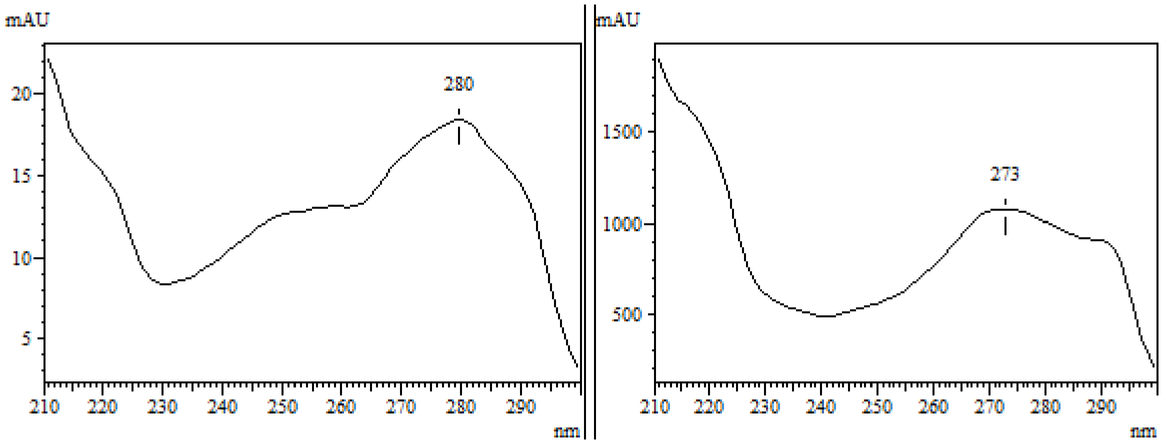

2-Methoxy-*N*,*N*-dimethylacetamide (1a)

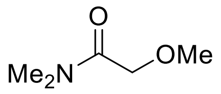

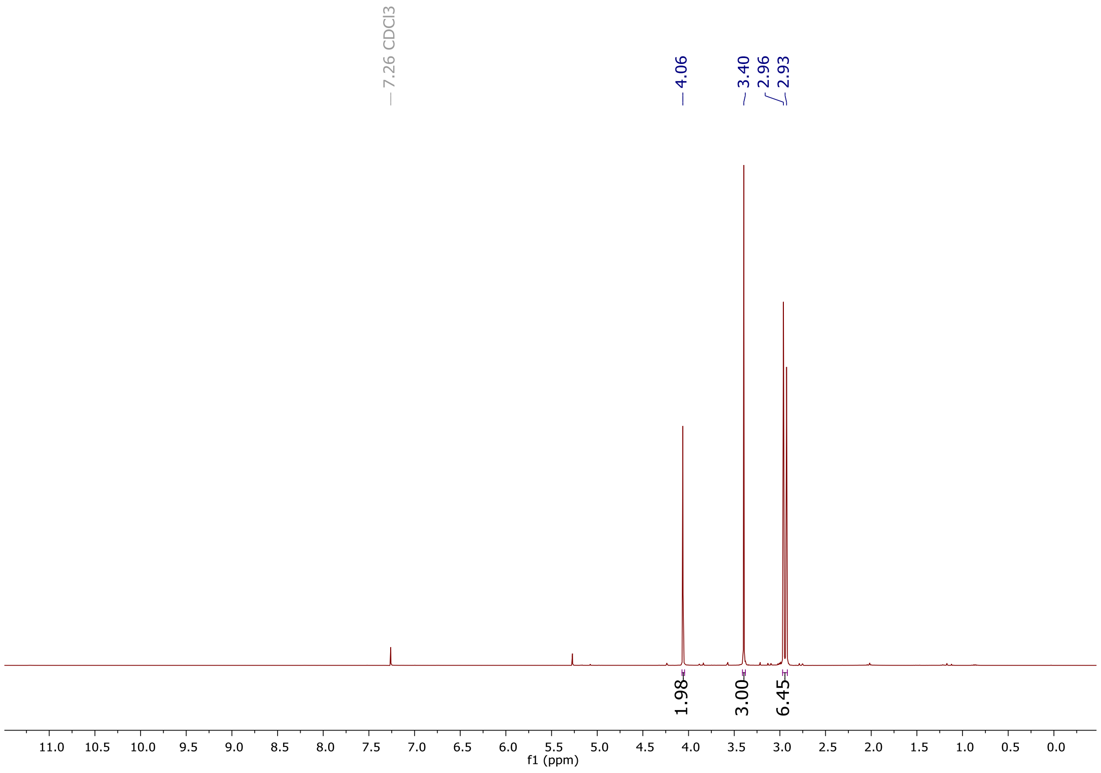

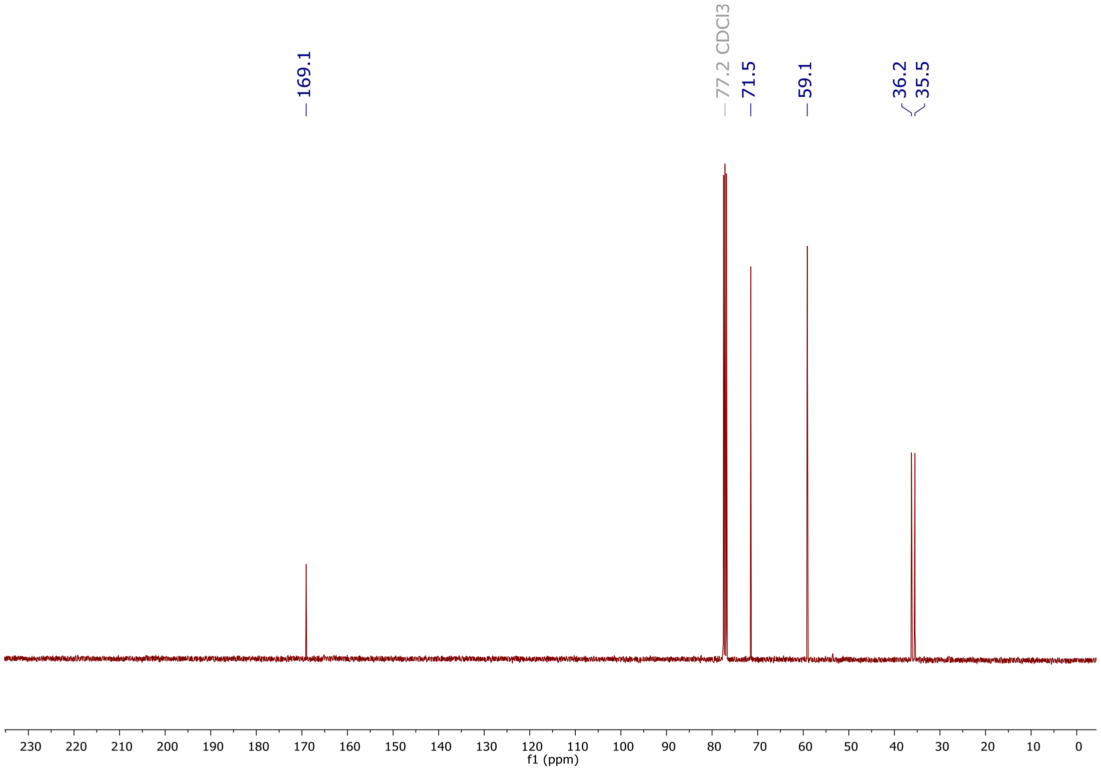

2,2-Diethoxy-*N*,*N*-dimethylacetamide (1b)

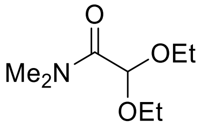

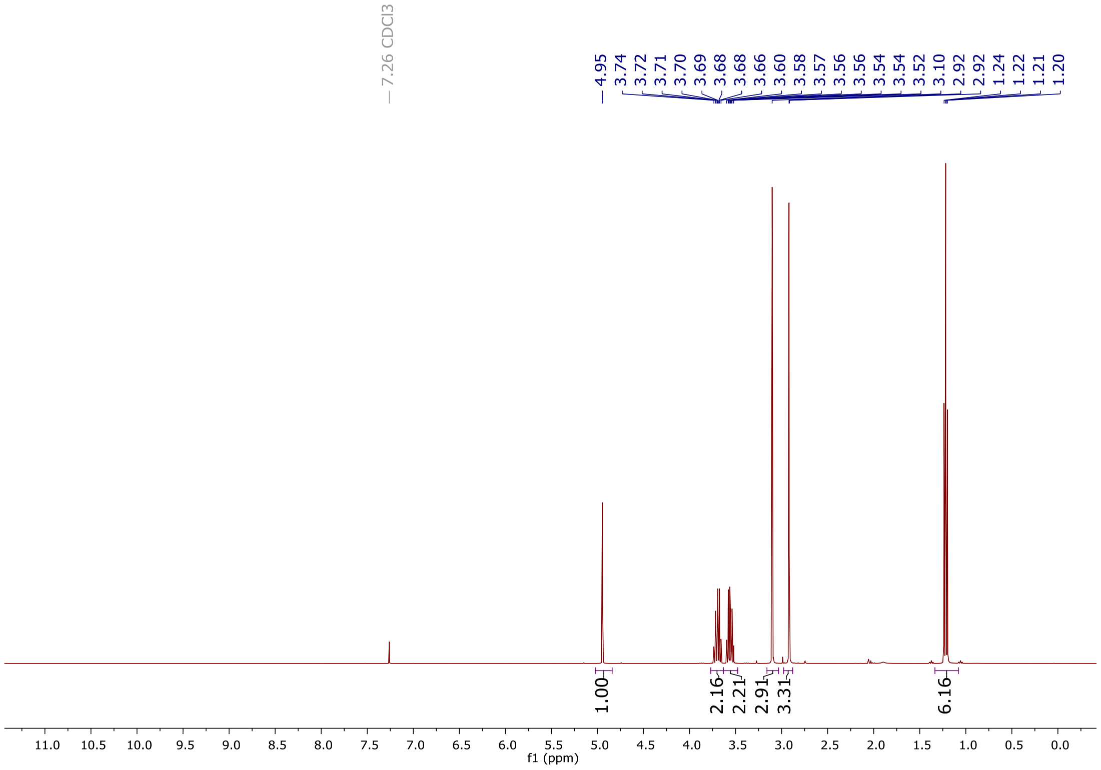

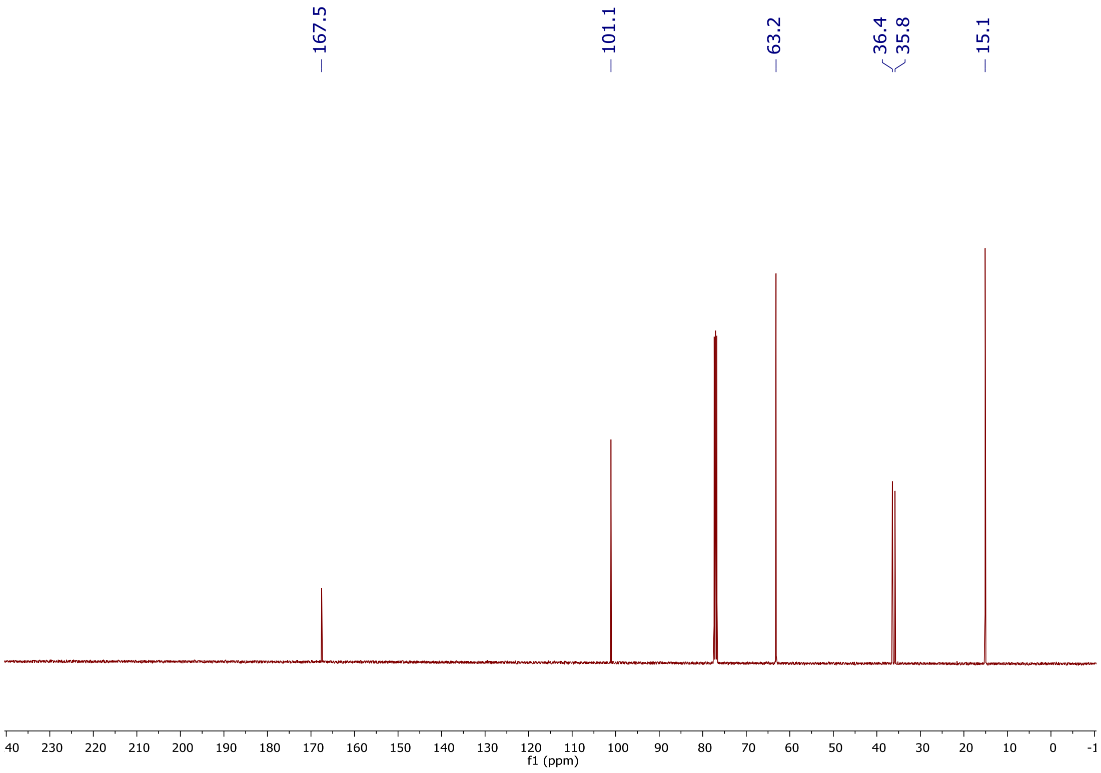

2-Fluoro-*N,N*-dimethylacetamide (1c)

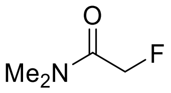

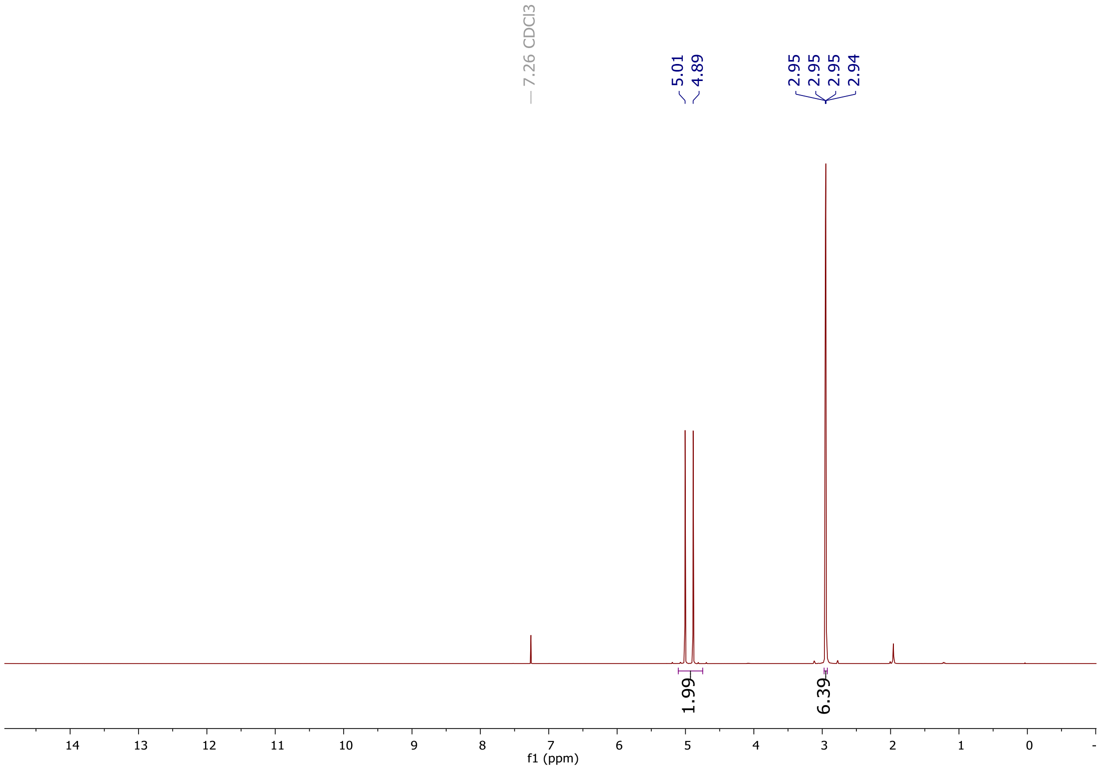

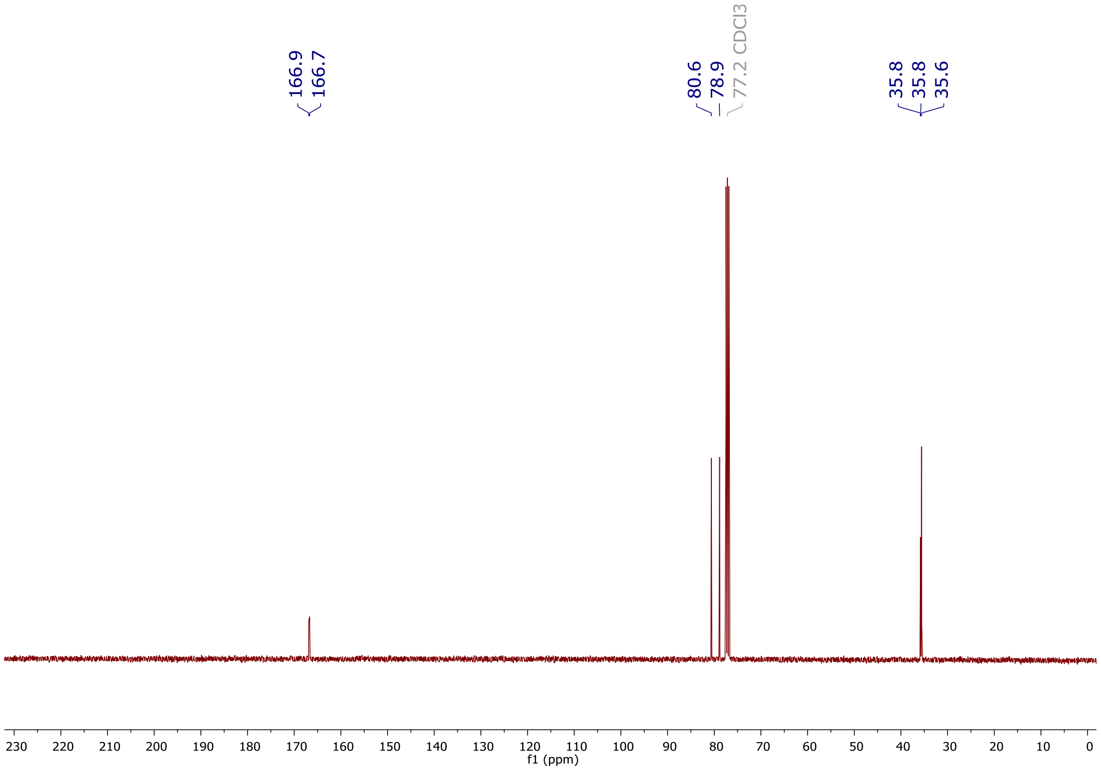

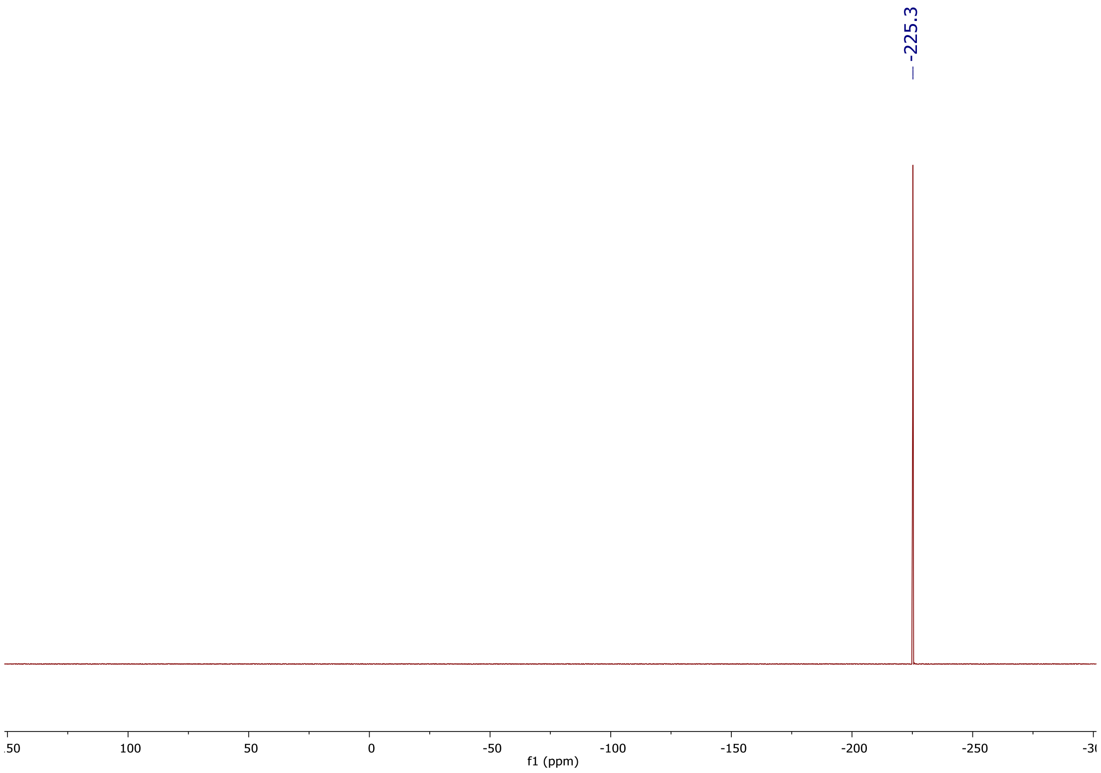
-225.3
50
100
50
0
-50
-100
-150
-200
-250
f1 (ppm)

2,2-Difluoro-*N,N*-dimethylacetamide (1d)

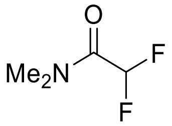

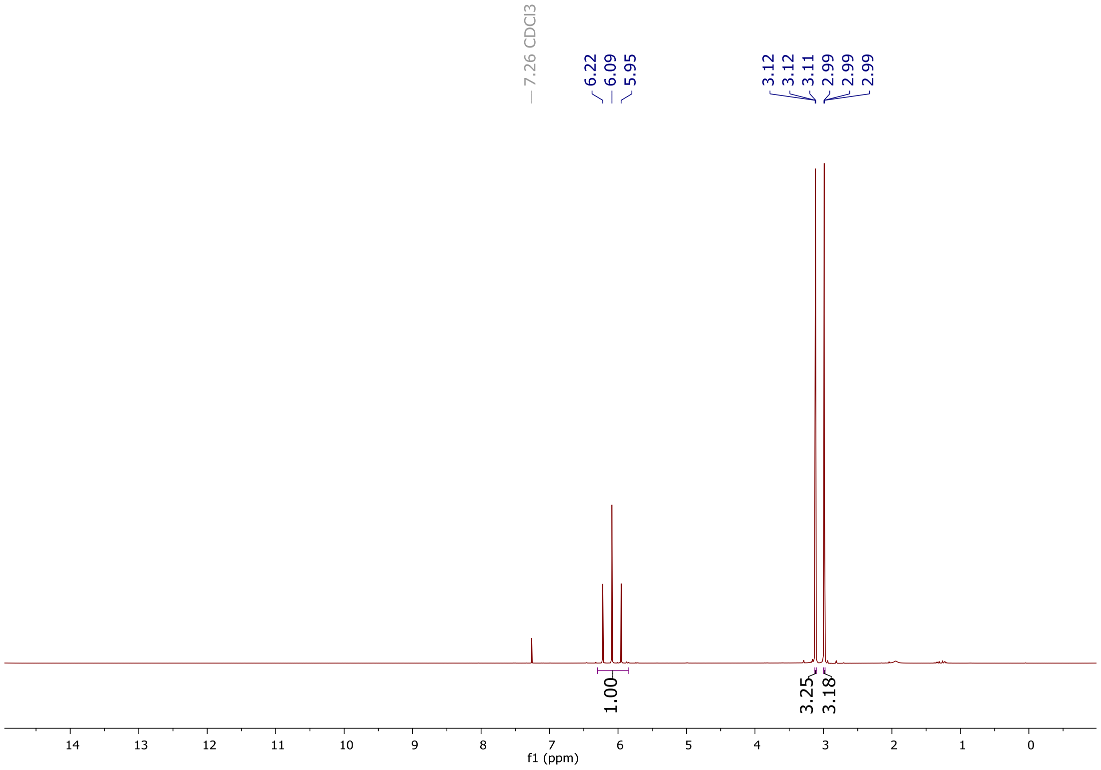

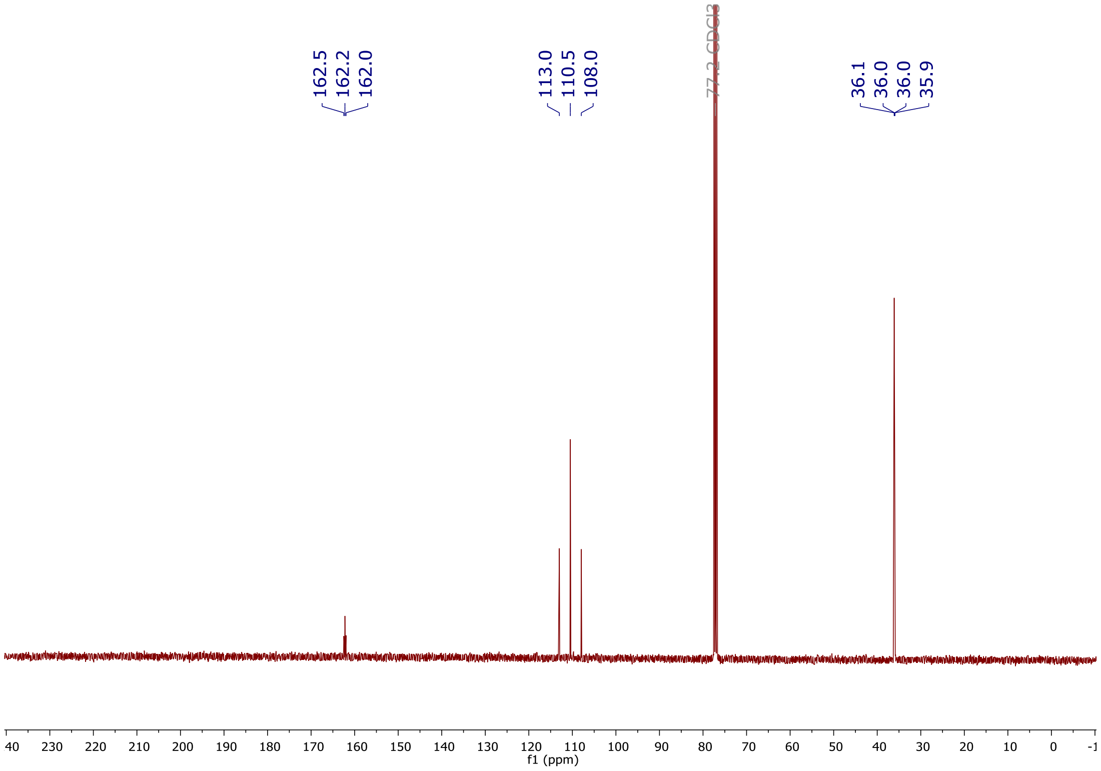

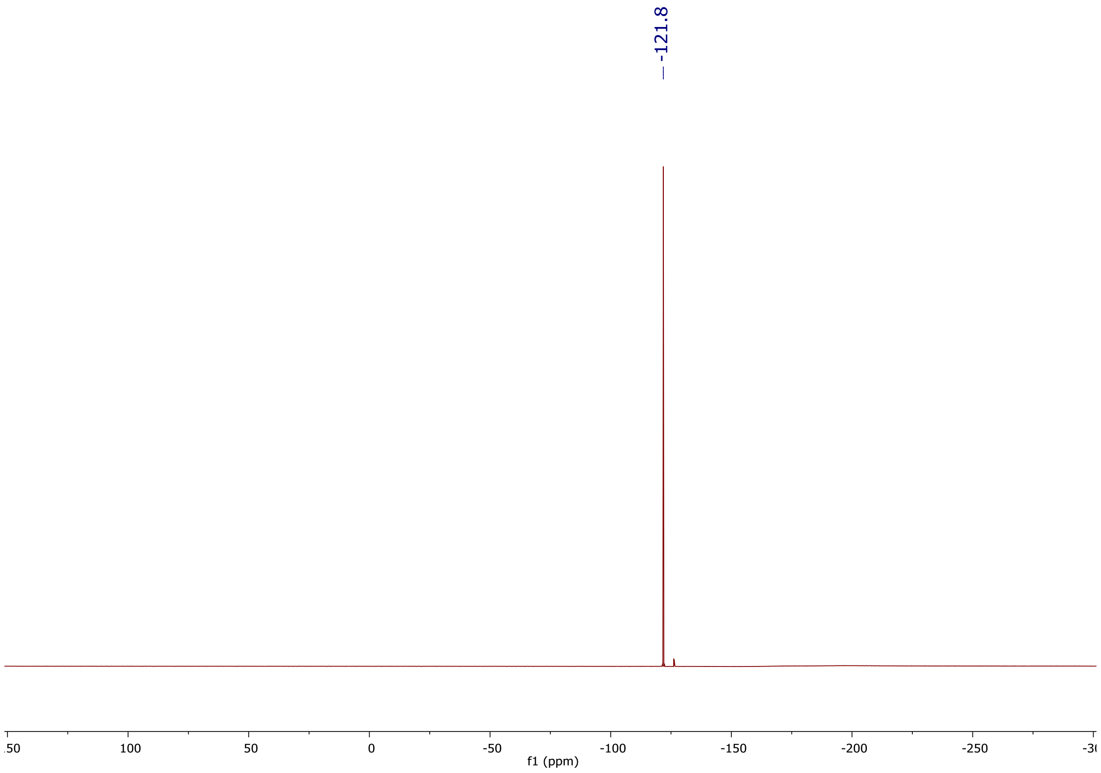
-121.8
50
100
50
0
-50
-100
-150
-200
-250
f1 (ppm)

3-(2-((*Tert*-butyldimethylsilyl)oxy)phenyl)-*N,N*-dimethylpropanamide (1f)

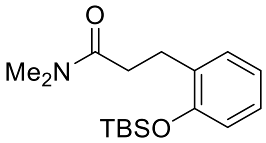

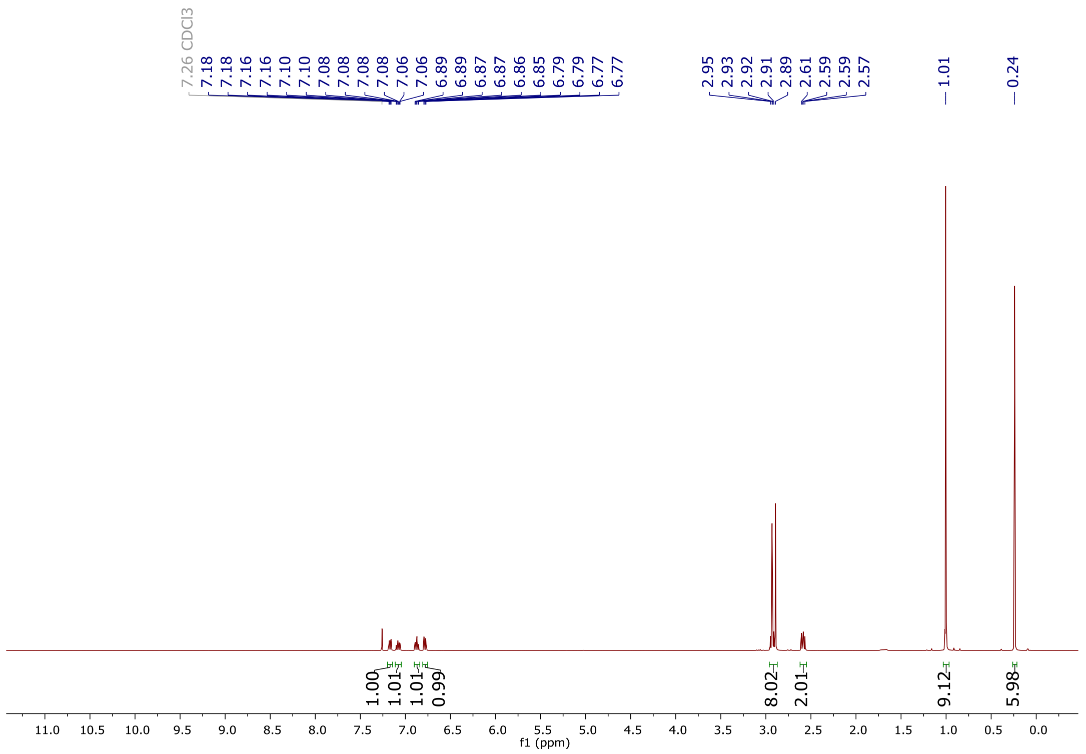

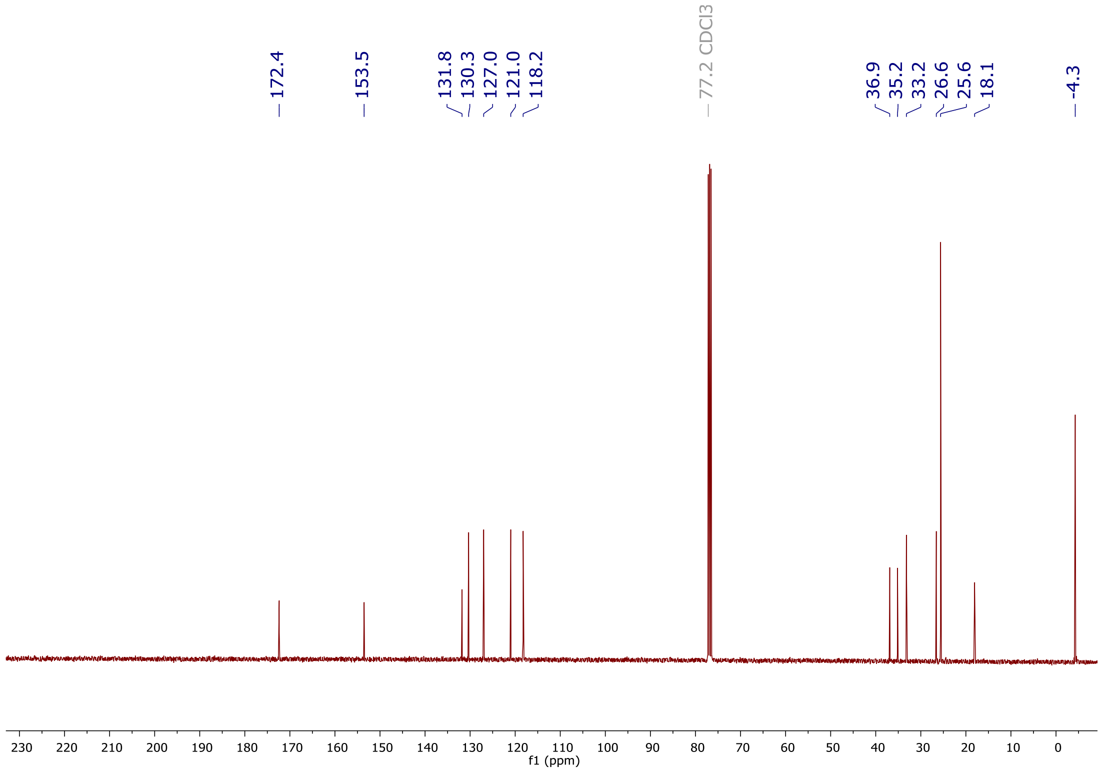

***N,N*-Dimethyl-3-(piperidin-1-yl)propanamide (1g)**

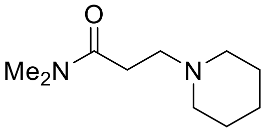

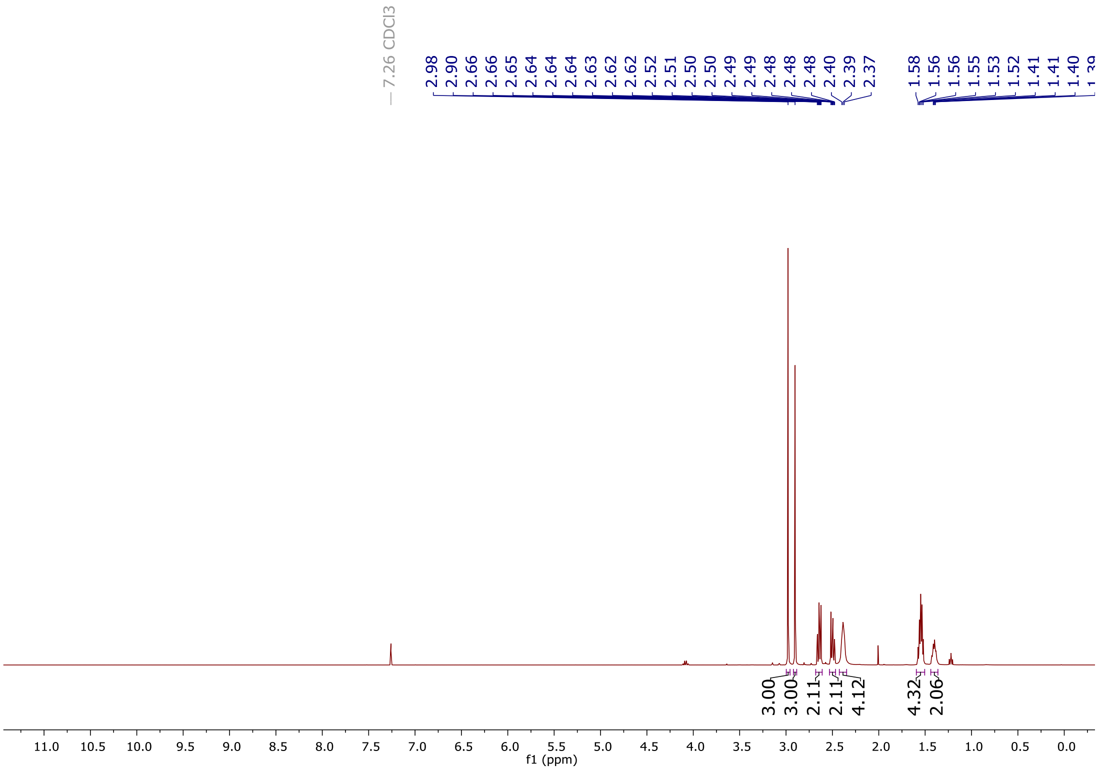

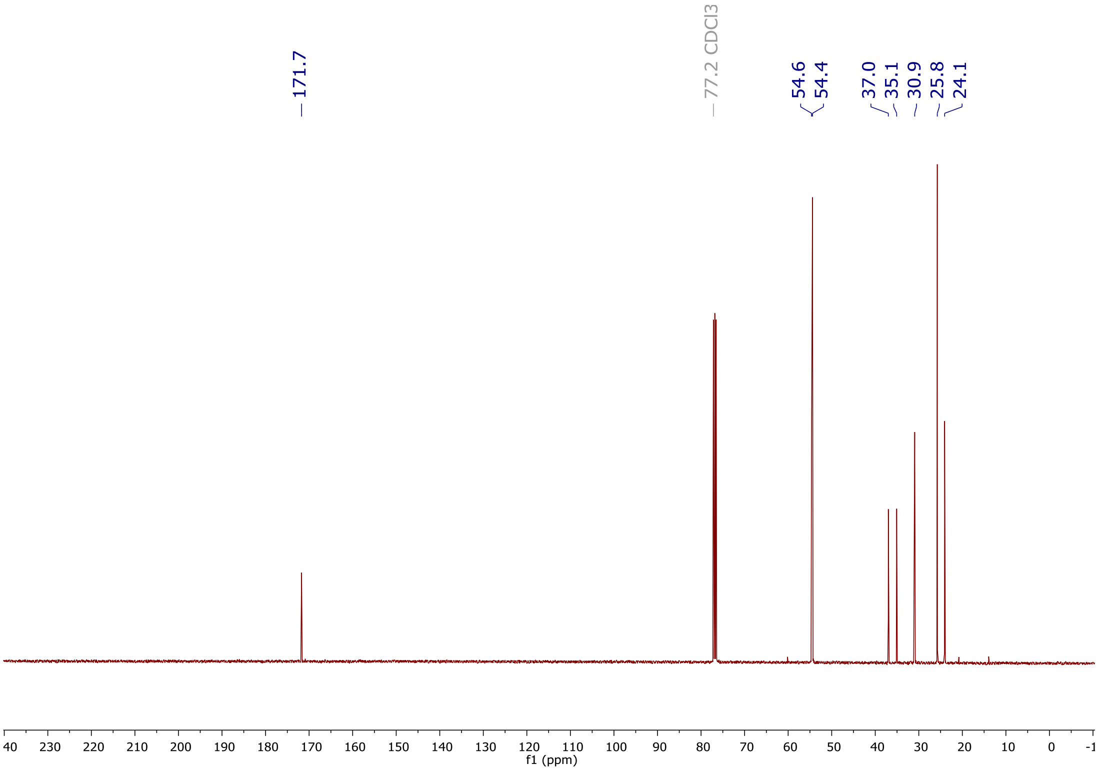

2-((1*r*,4*r*)-4-(4-Chlorophenyl)cyclohexyl)-*N,N*-dimethylacetamide (1h)

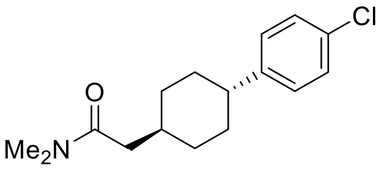

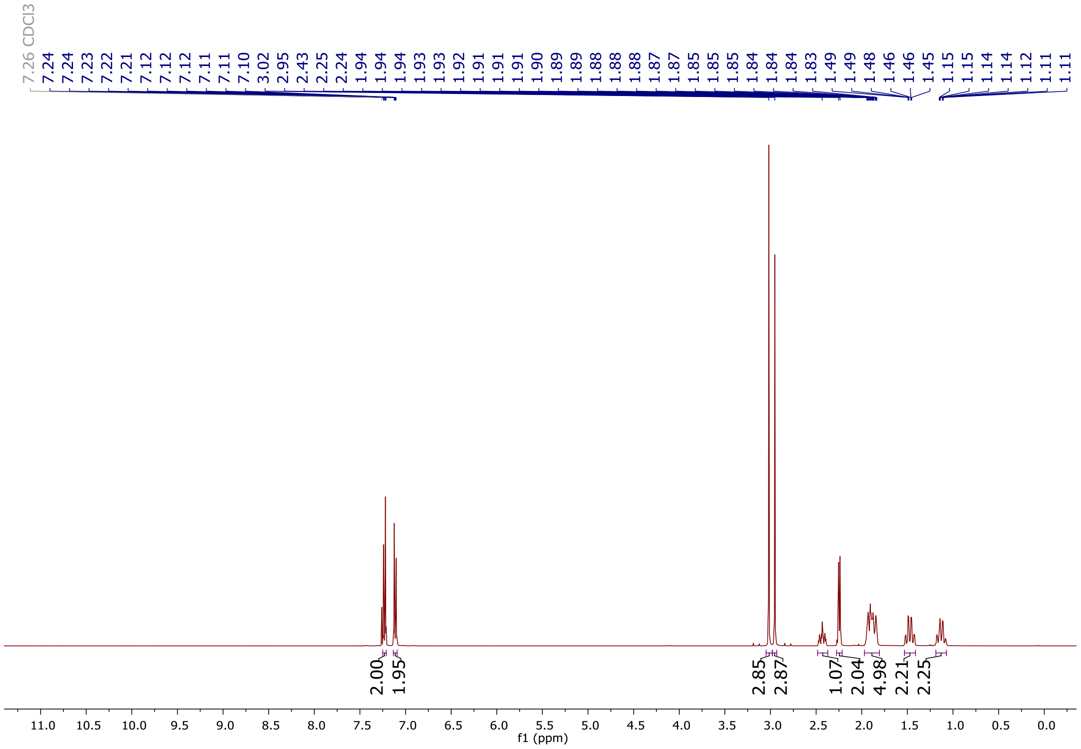

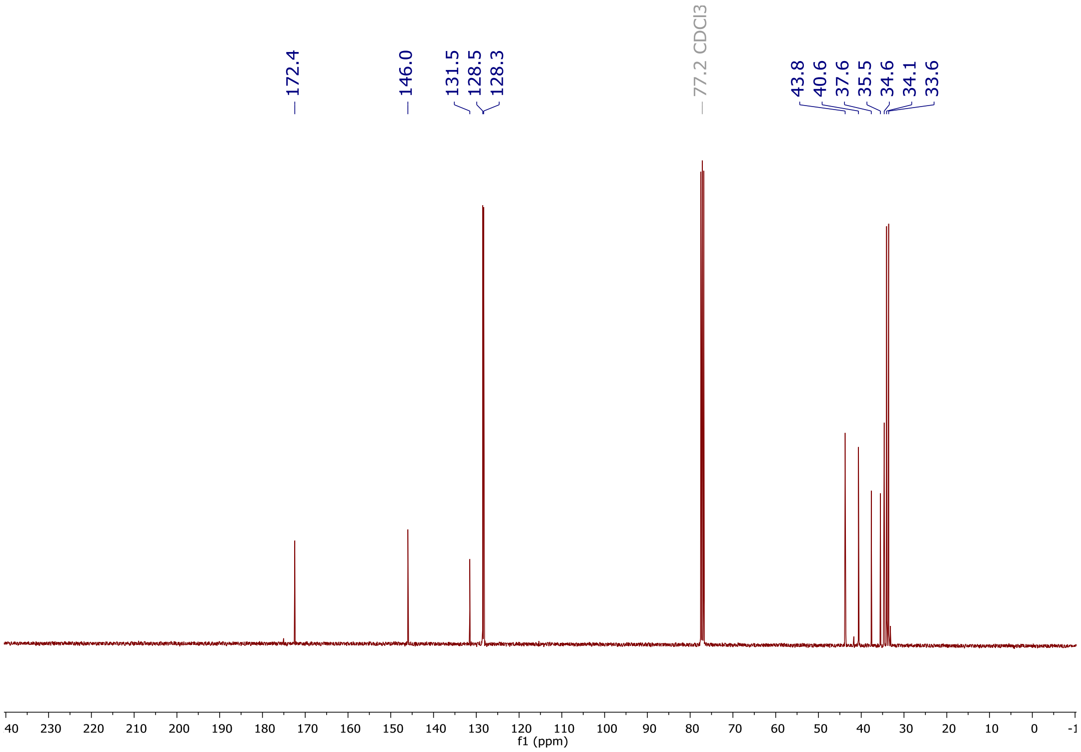

2-(4-Methoxyphenyl)-*N,N*-dimethylacetamide (1i)

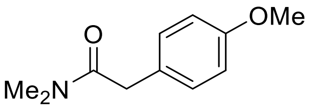

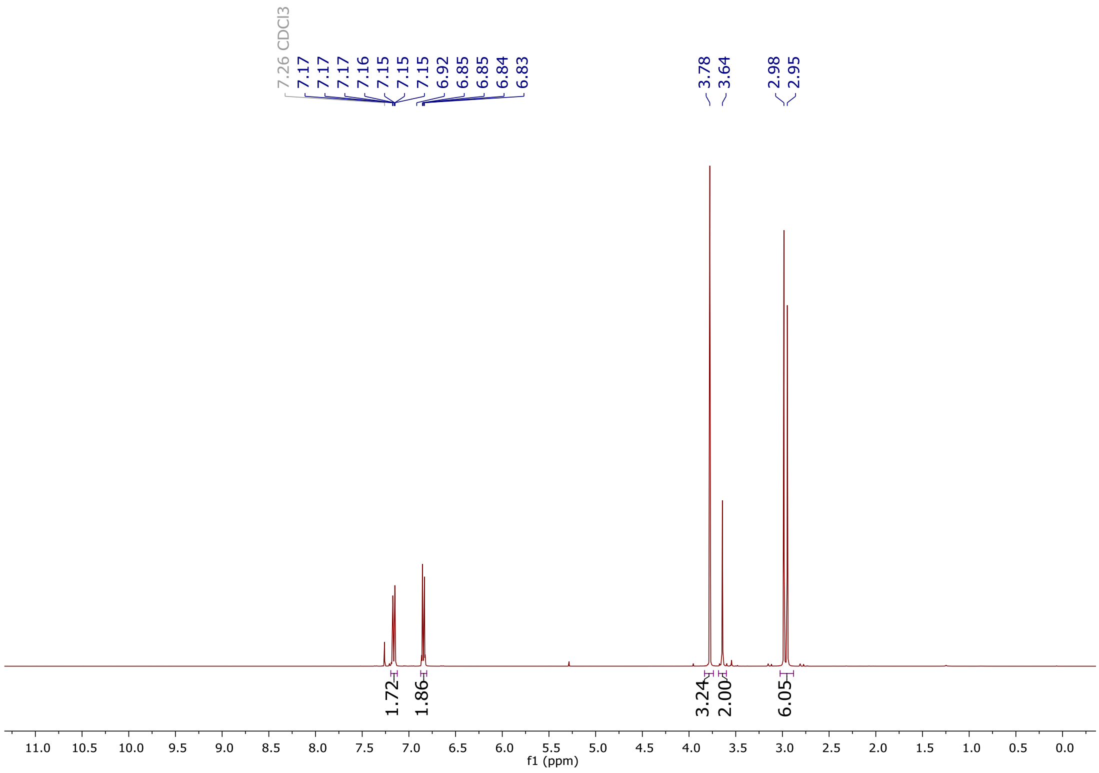

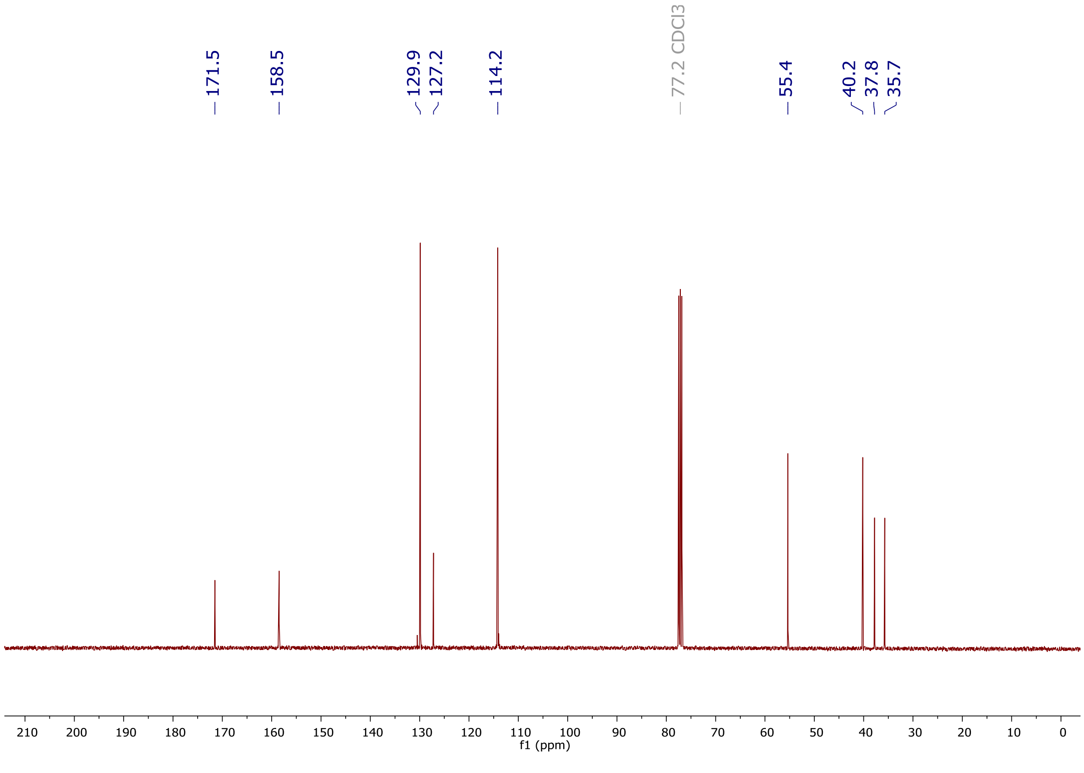

_N,N_-Dimethylbicyclo[1.1.1]pentane-1-carboxamide (1j)

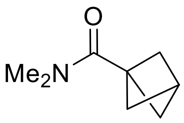

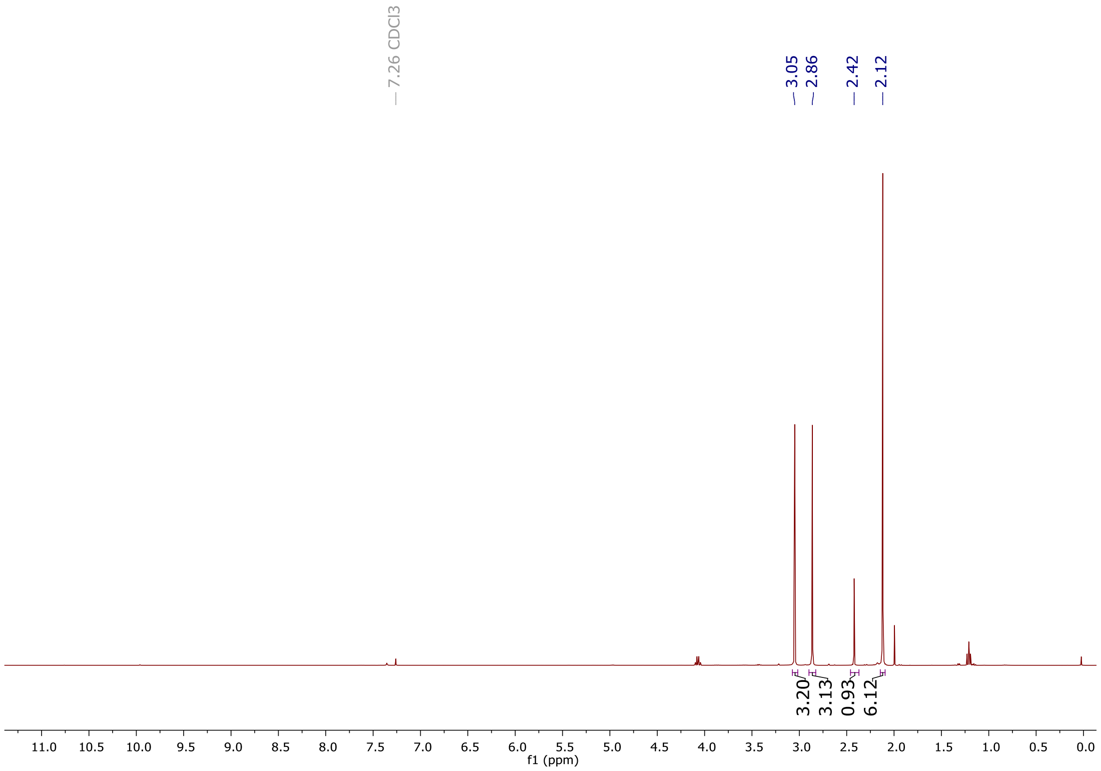

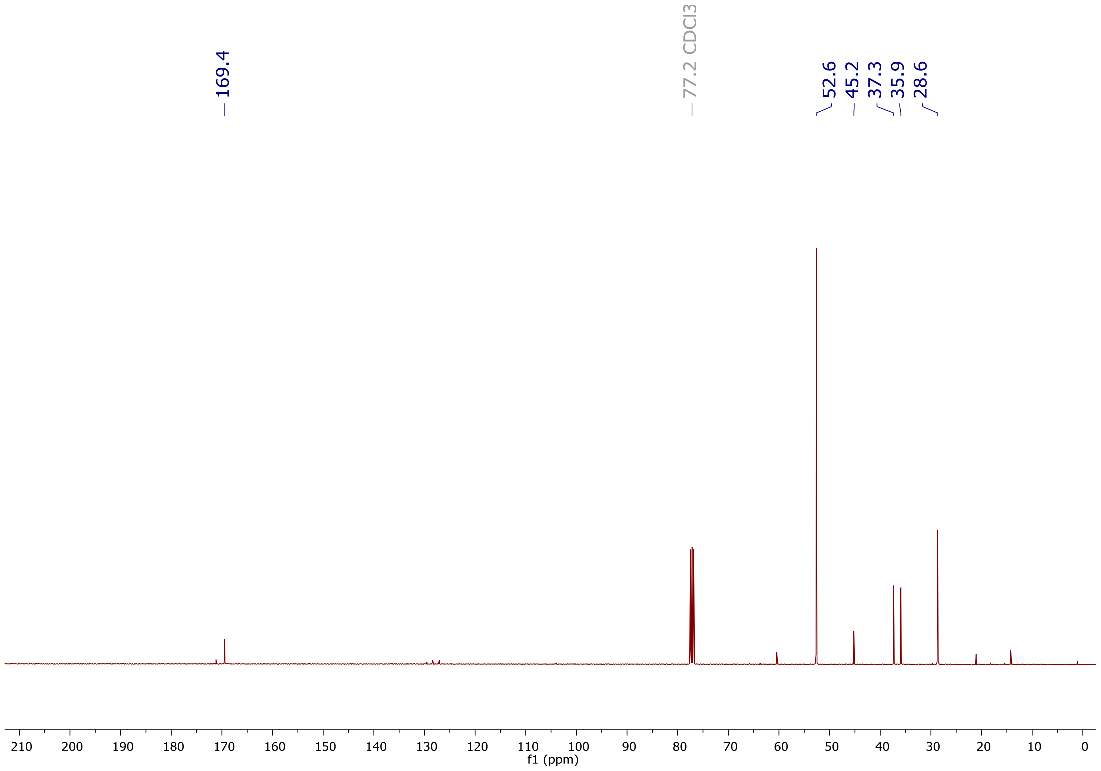

4-Cyano-*N,N*-dimethylbenzamide (6a)

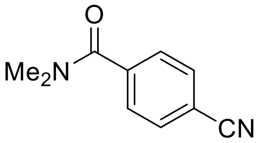

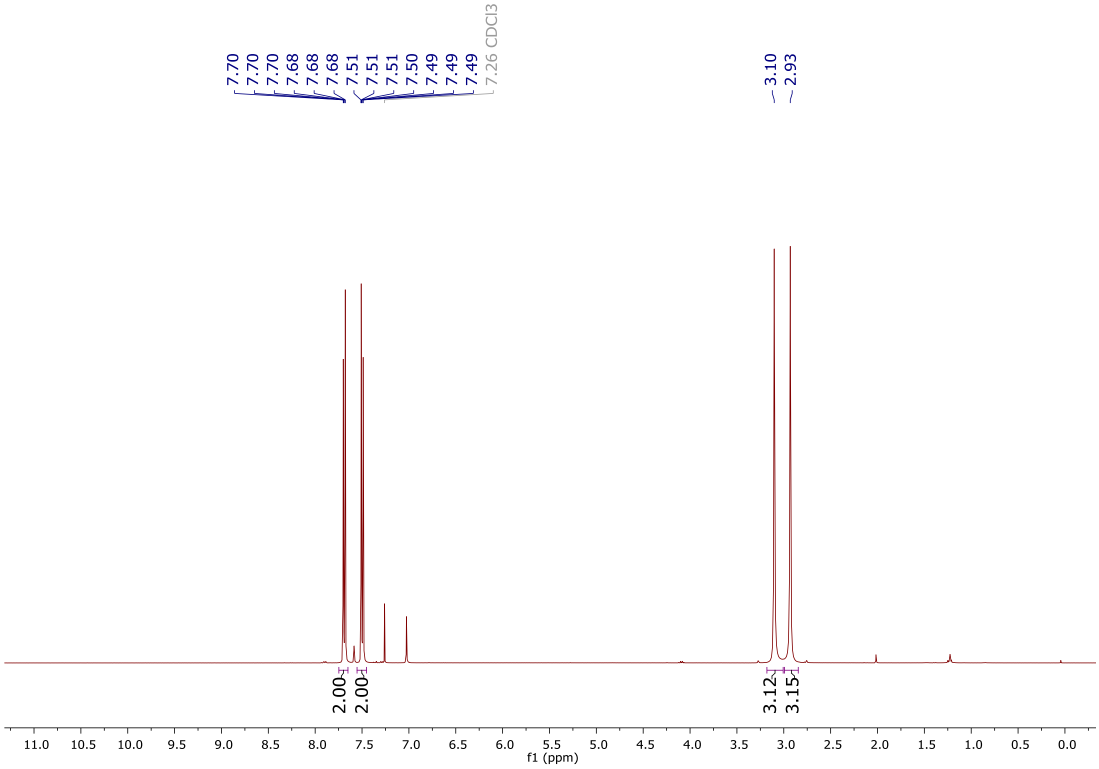

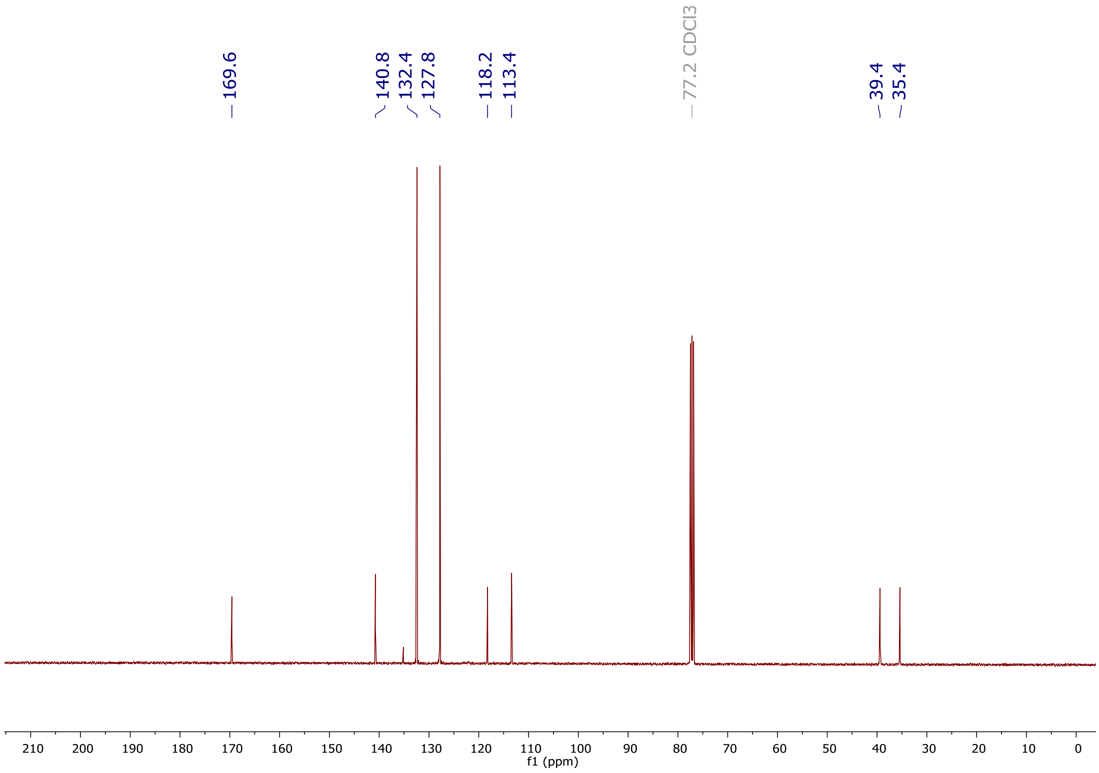

4-Iodo-N,N-dimethylbenzamide (6b)

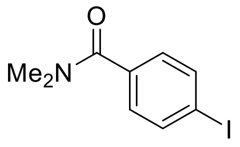

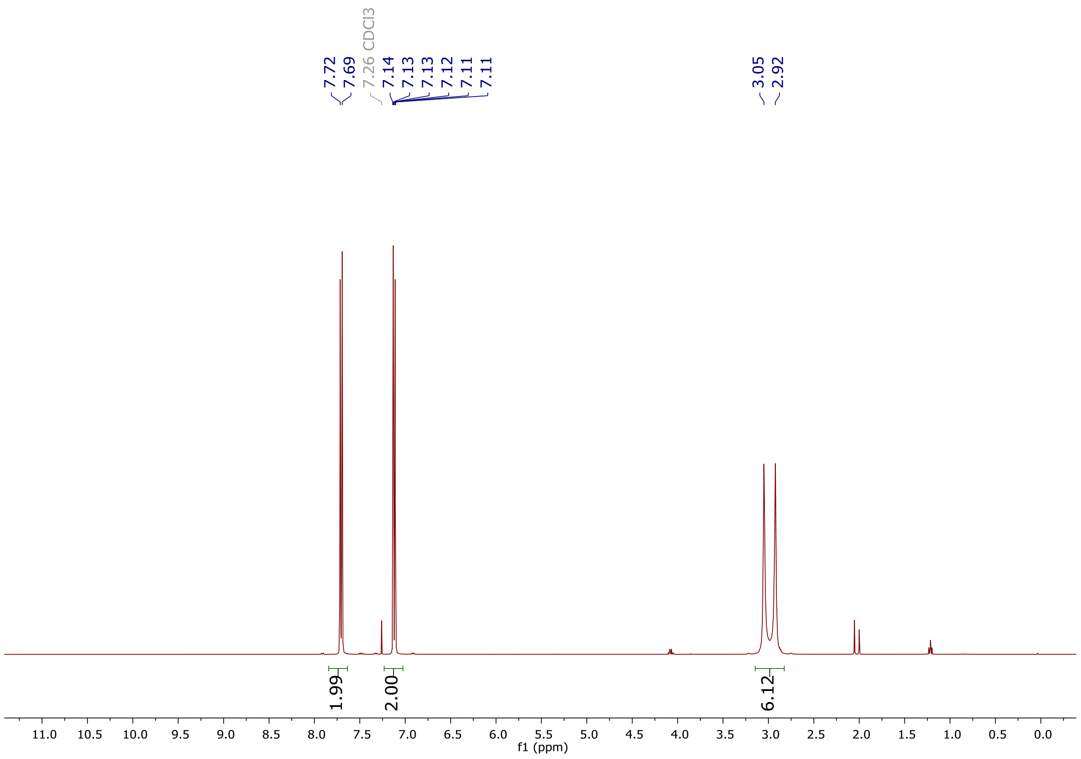

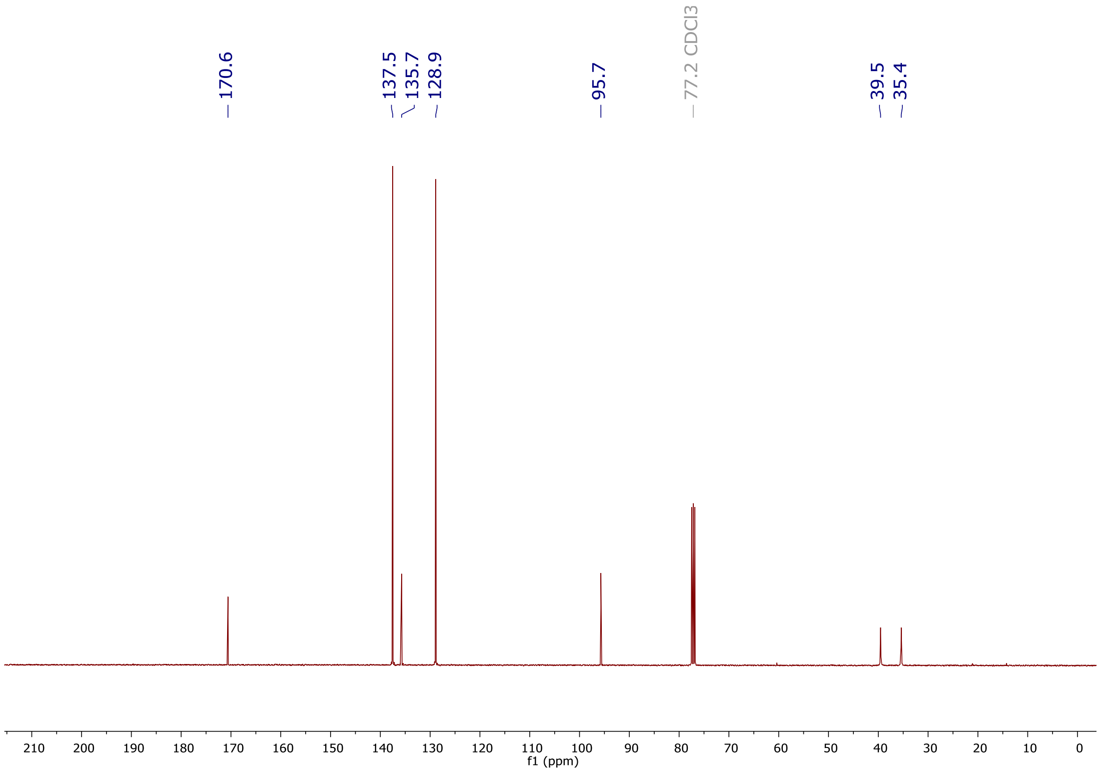

(*S*)-2-(6-Methoxynaphthalen-2-yl)-*N*,*N*-dimethylpropanamide (13a)

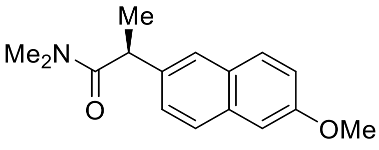

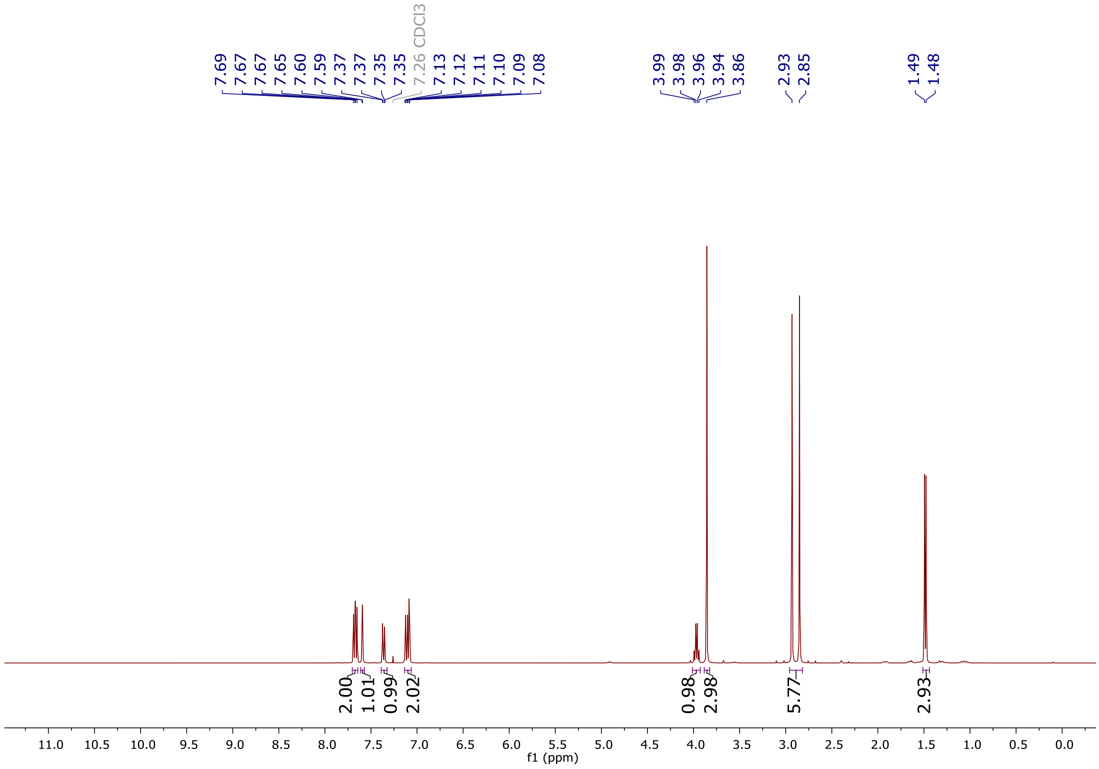

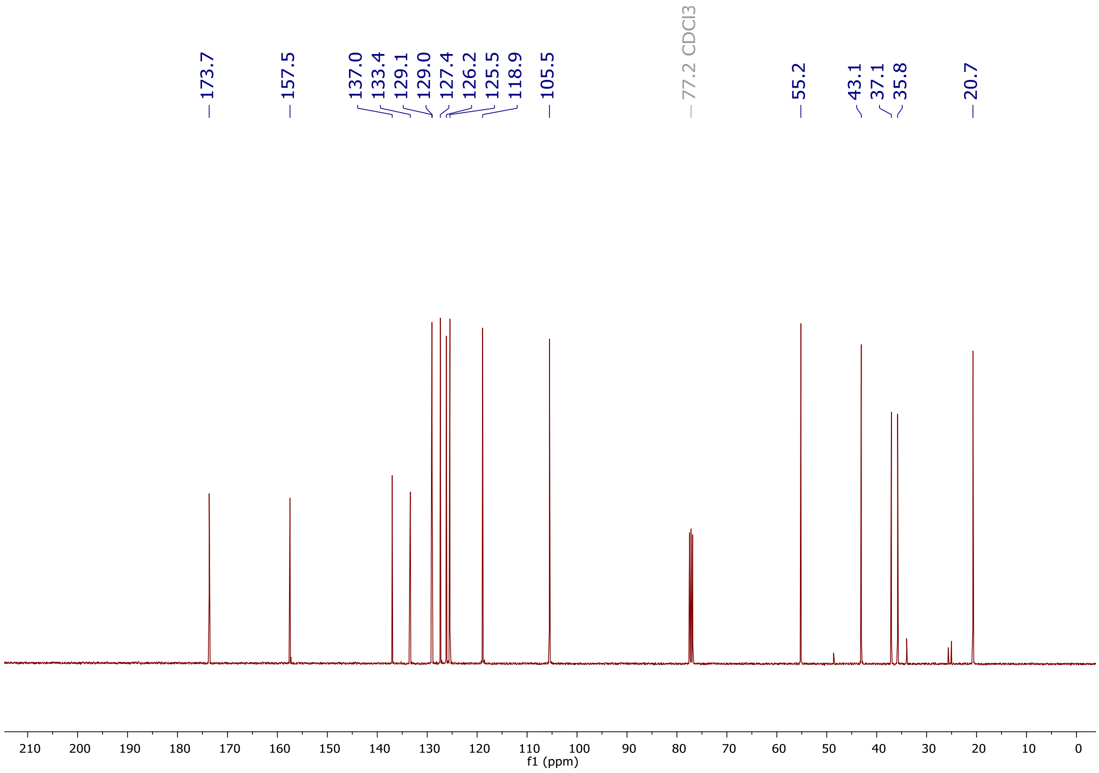

(*S*)-2-(4-*Iso*butylphenyl)-*N*,*N*-dimethylpropanamide (13b)

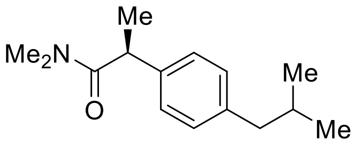

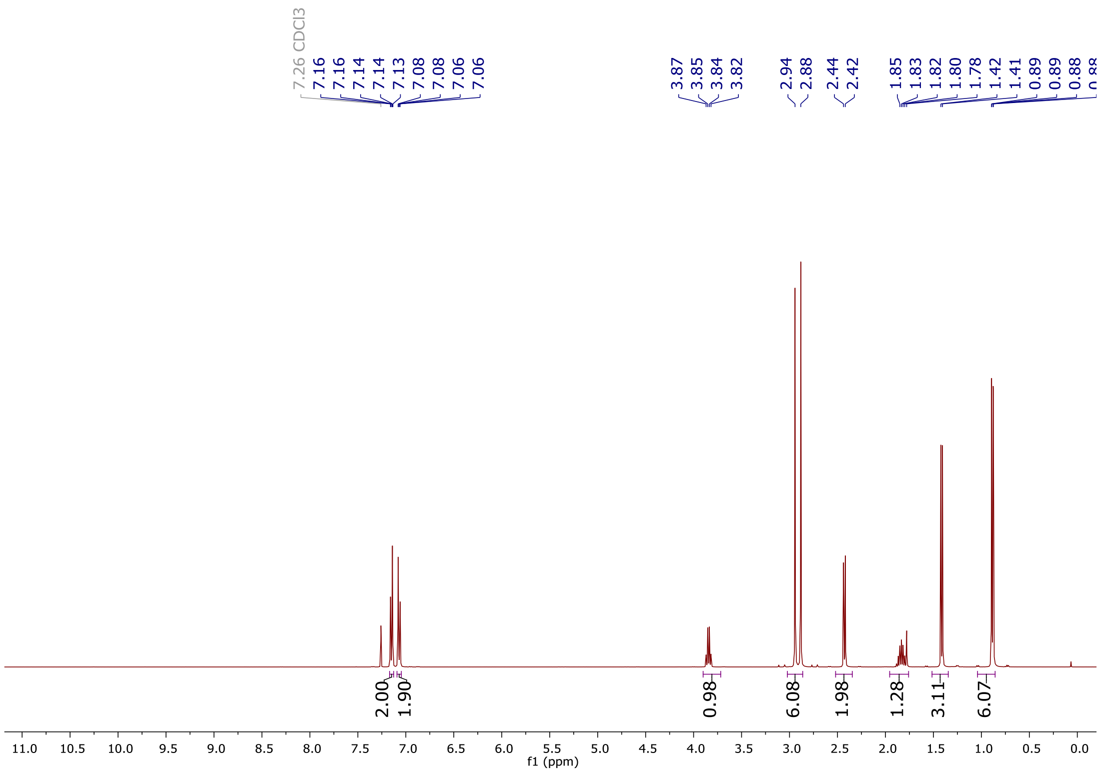

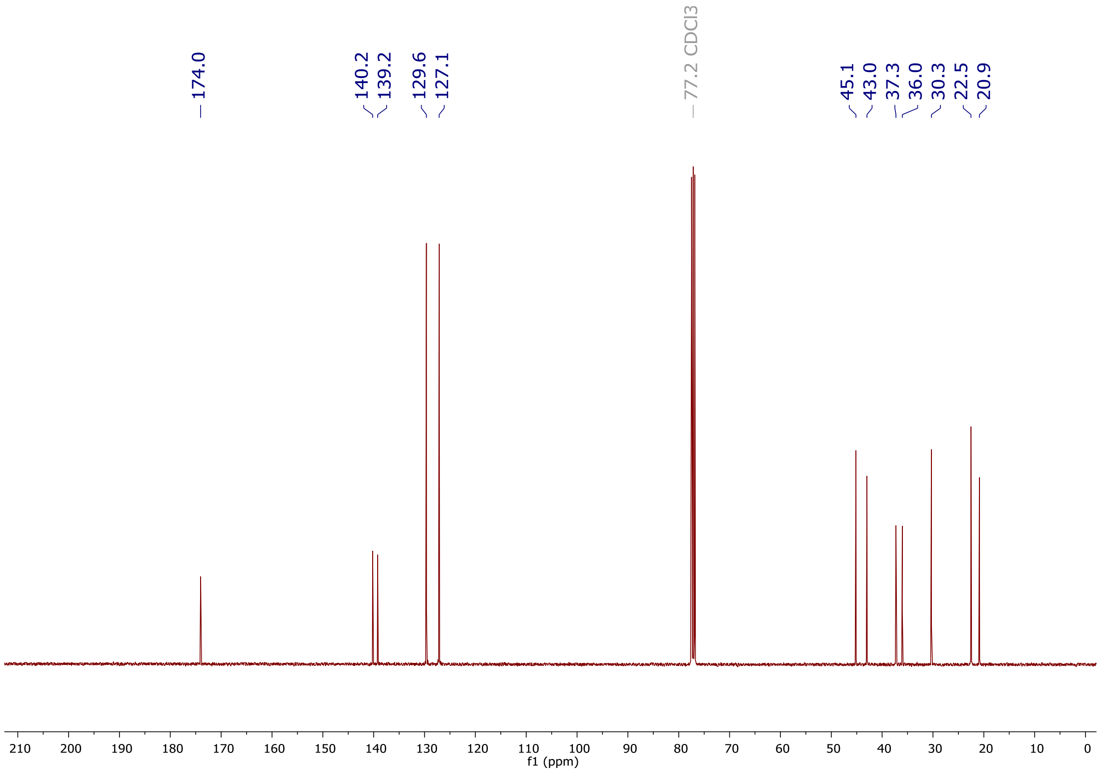

2-Methoxy-1-(4-(methylthio)phenyl)ethan-1-one (3aa)

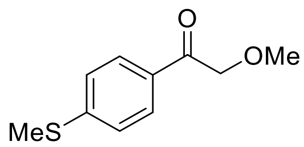

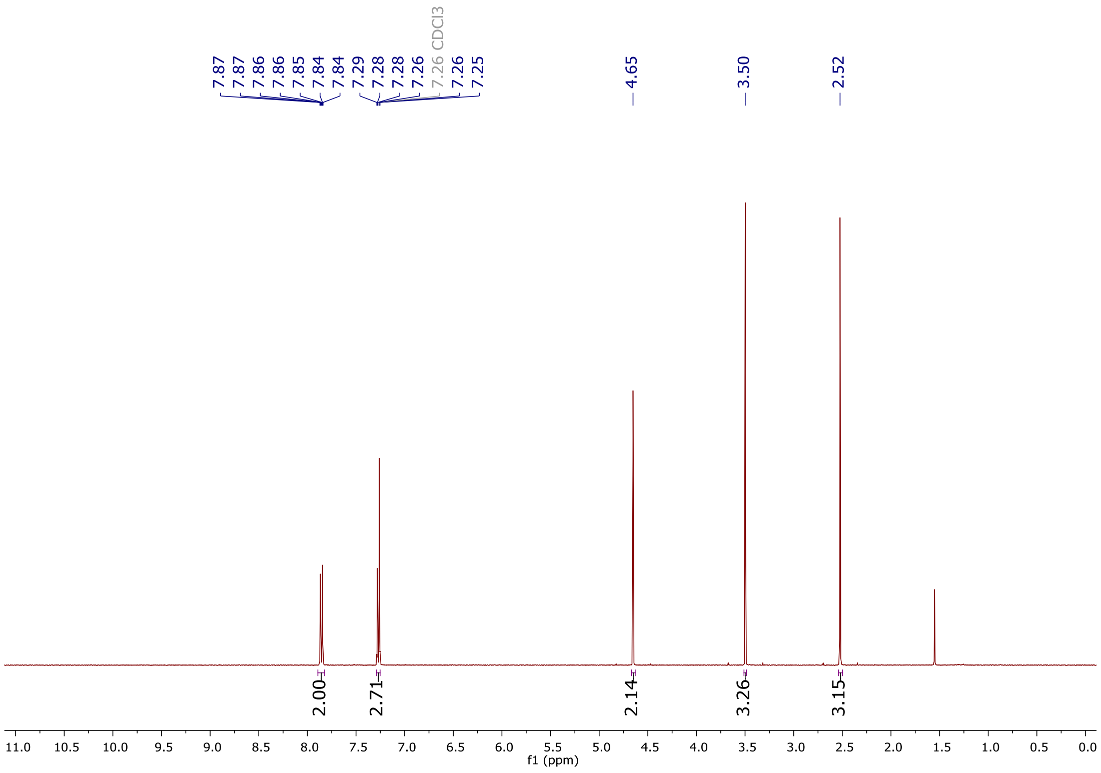

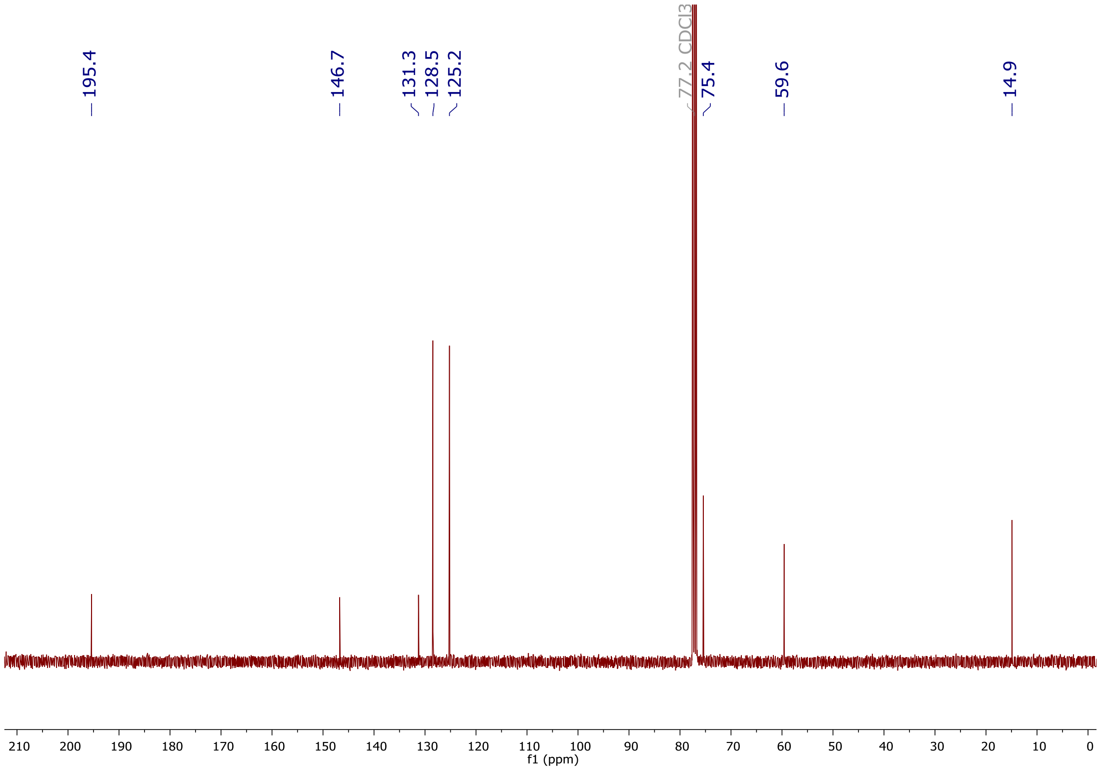

2-Methoxy-1-(2-methoxyphenyl)ethan-1-one (3ab)

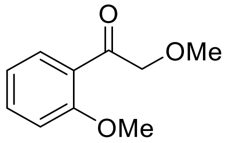

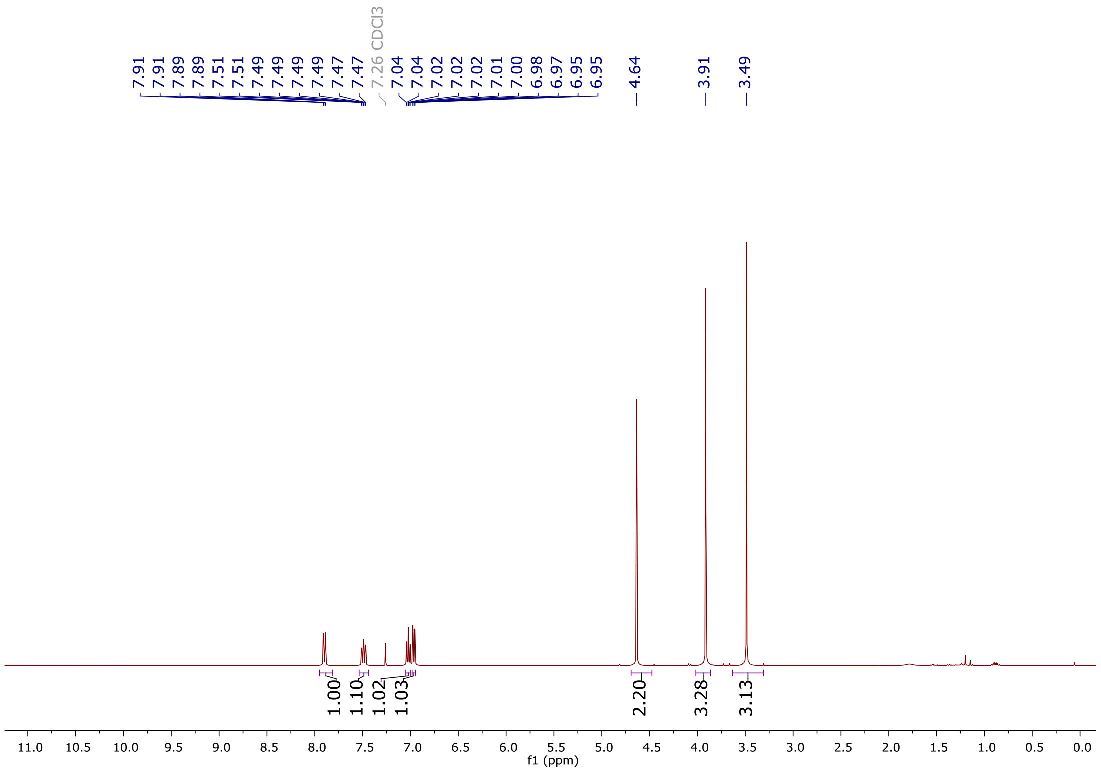

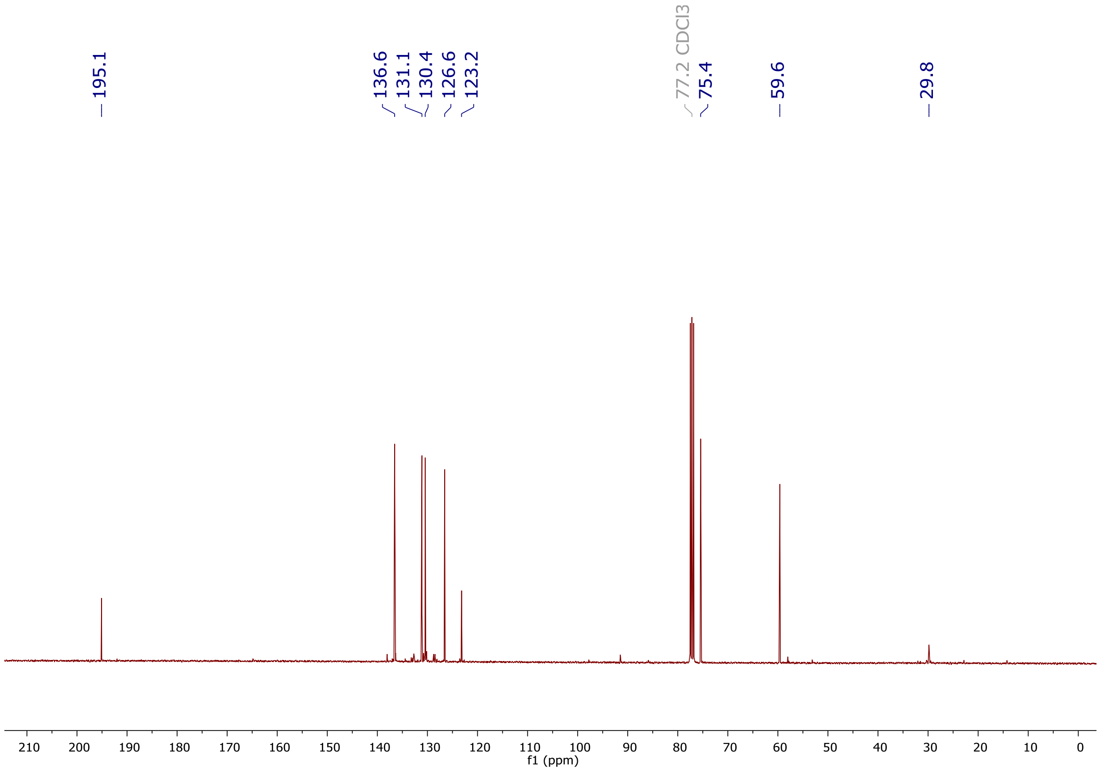

2-Methoxy-1-(2-methoxyphenyl)ethan-1-one (3ac)

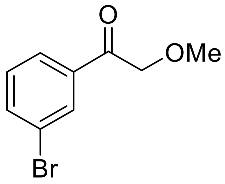

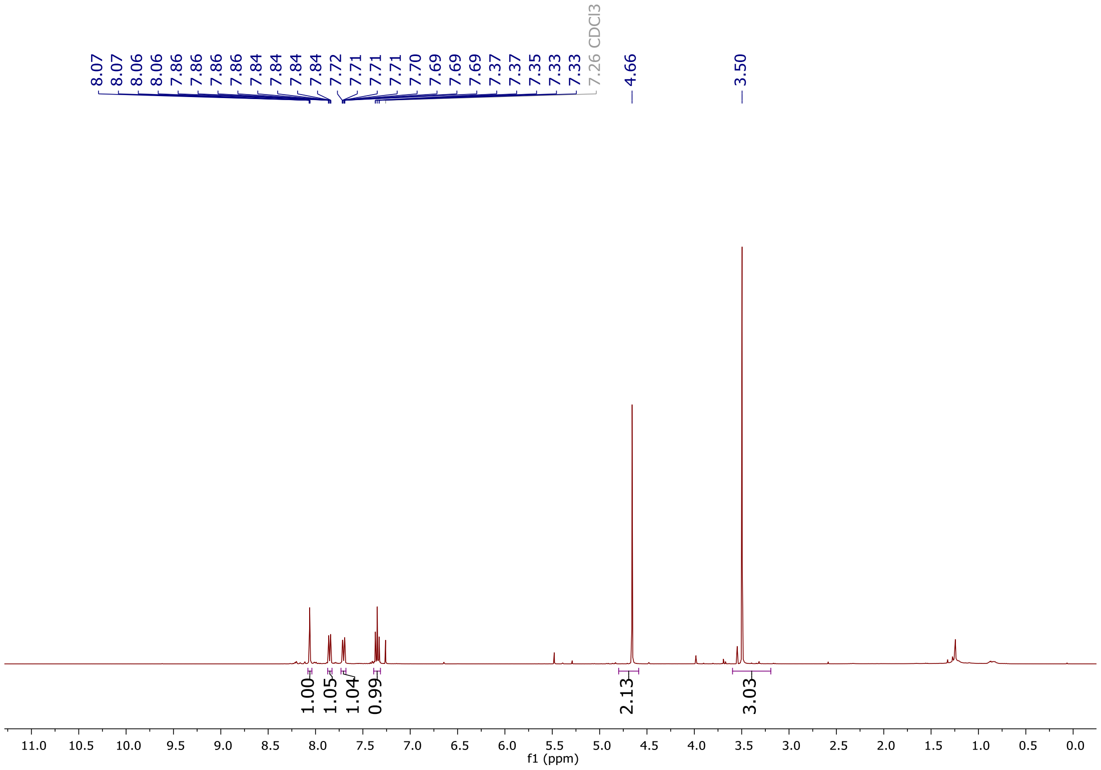

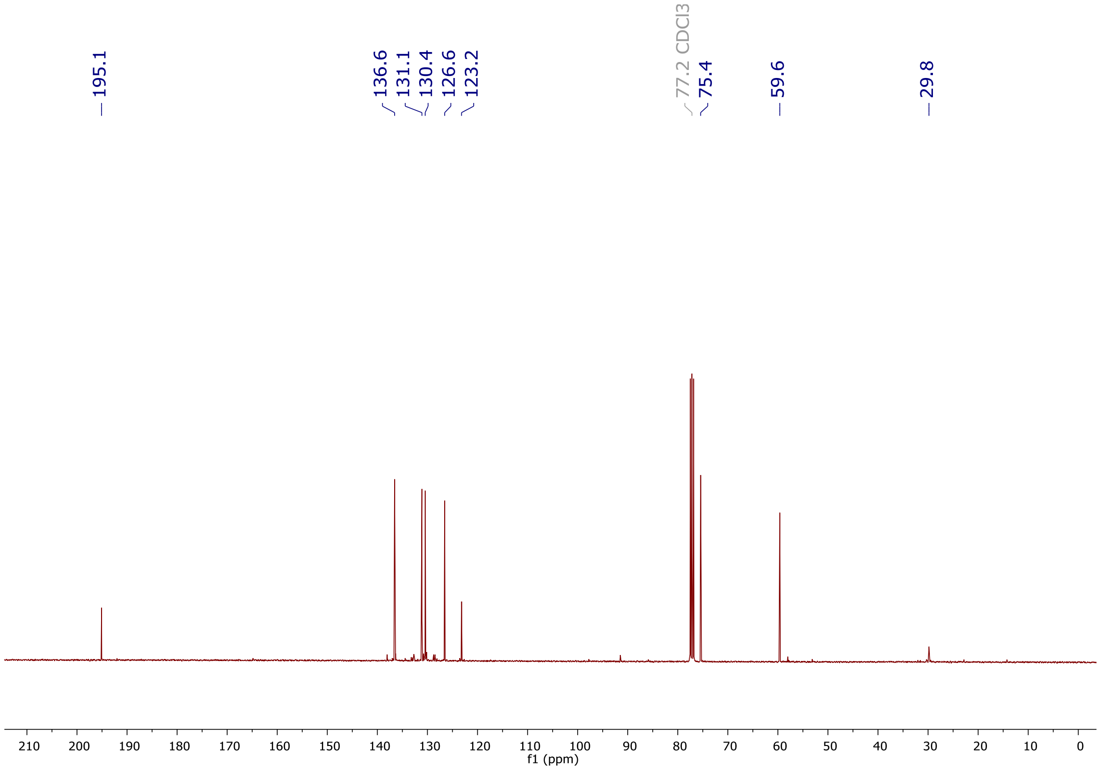

2-Methoxy-1-(2-methoxyphenyl)ethan-1-one (3ad)

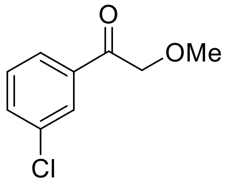

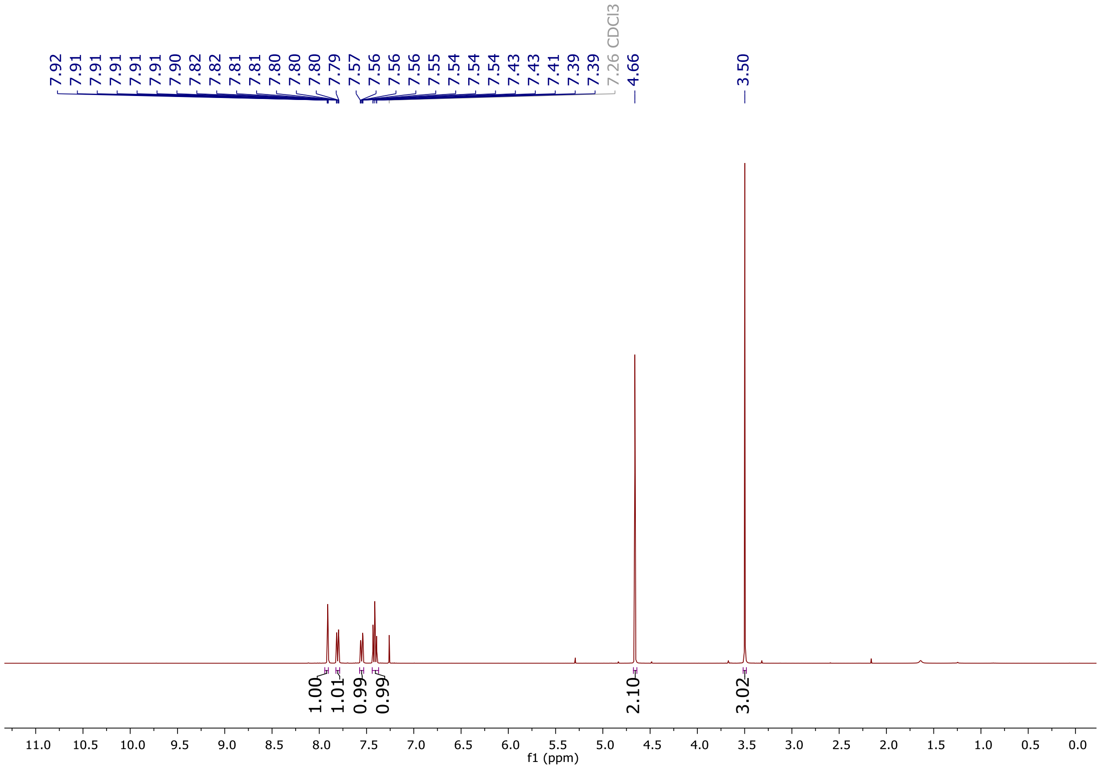

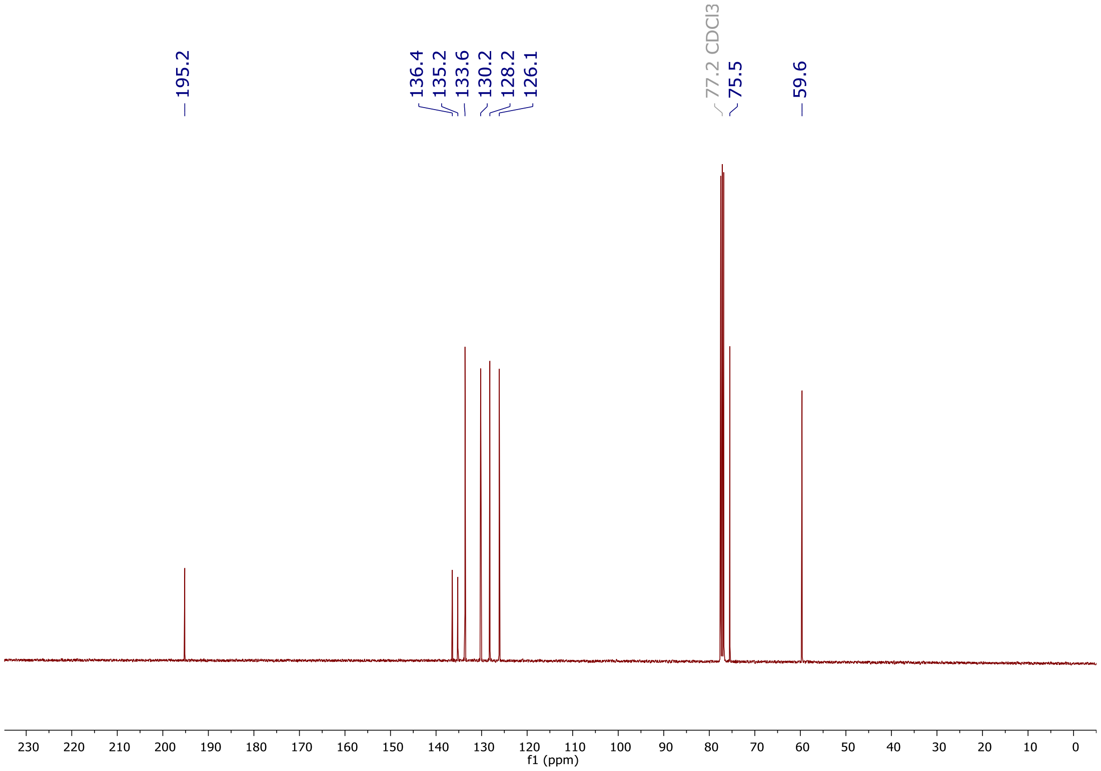

1-(5-Bromo-2-methoxyphenyl)-2-methoxyethan-1-one (3ae)

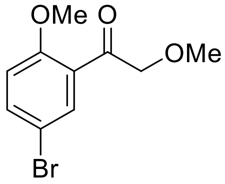

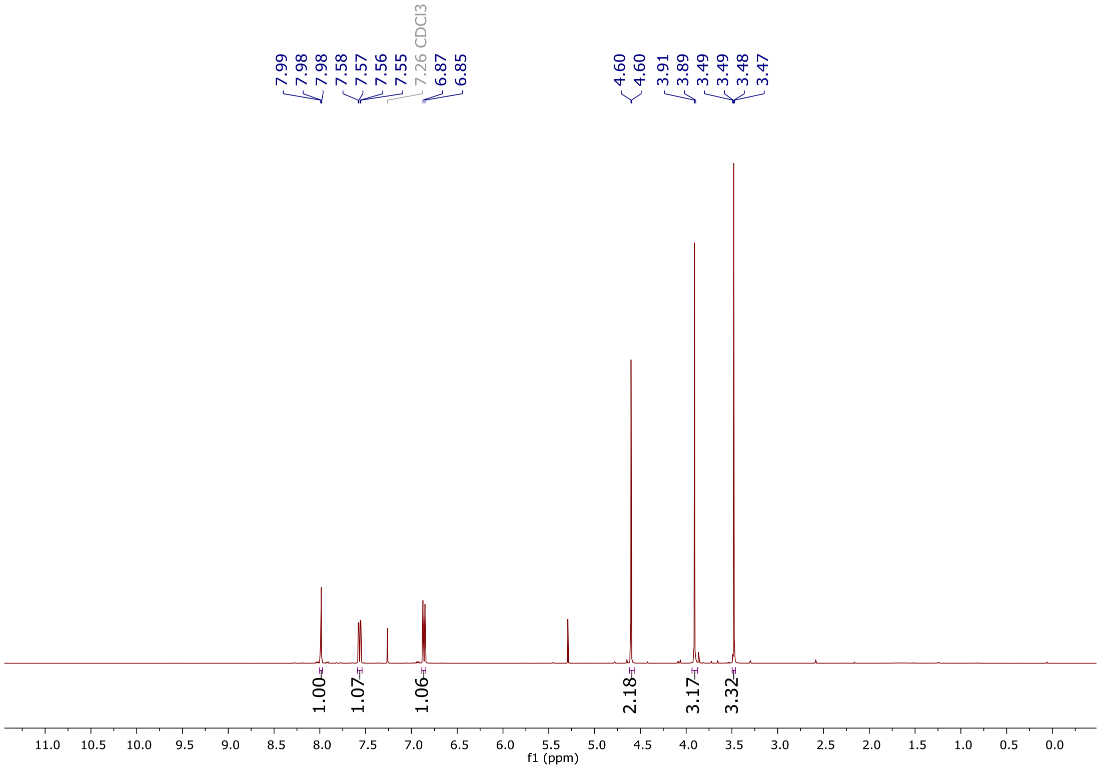

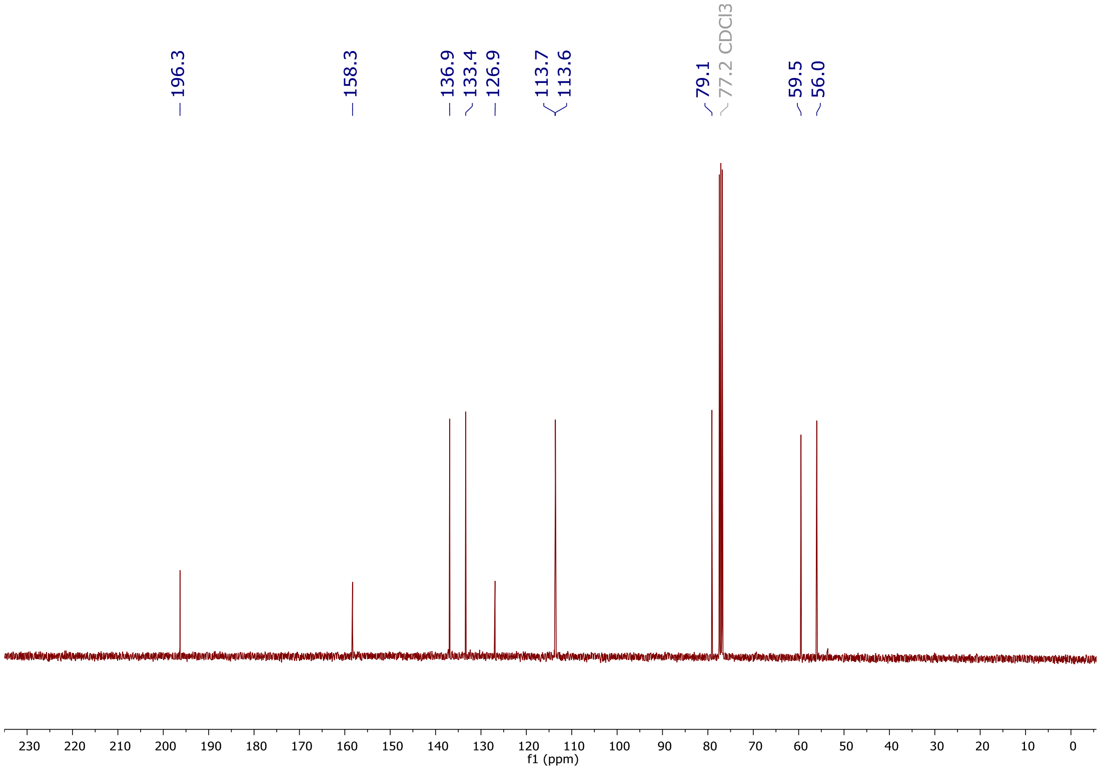

2-Methoxy-1-(6-methoxypyridin-2-yl)ethan-1-one (3af)

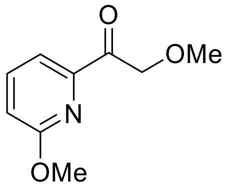

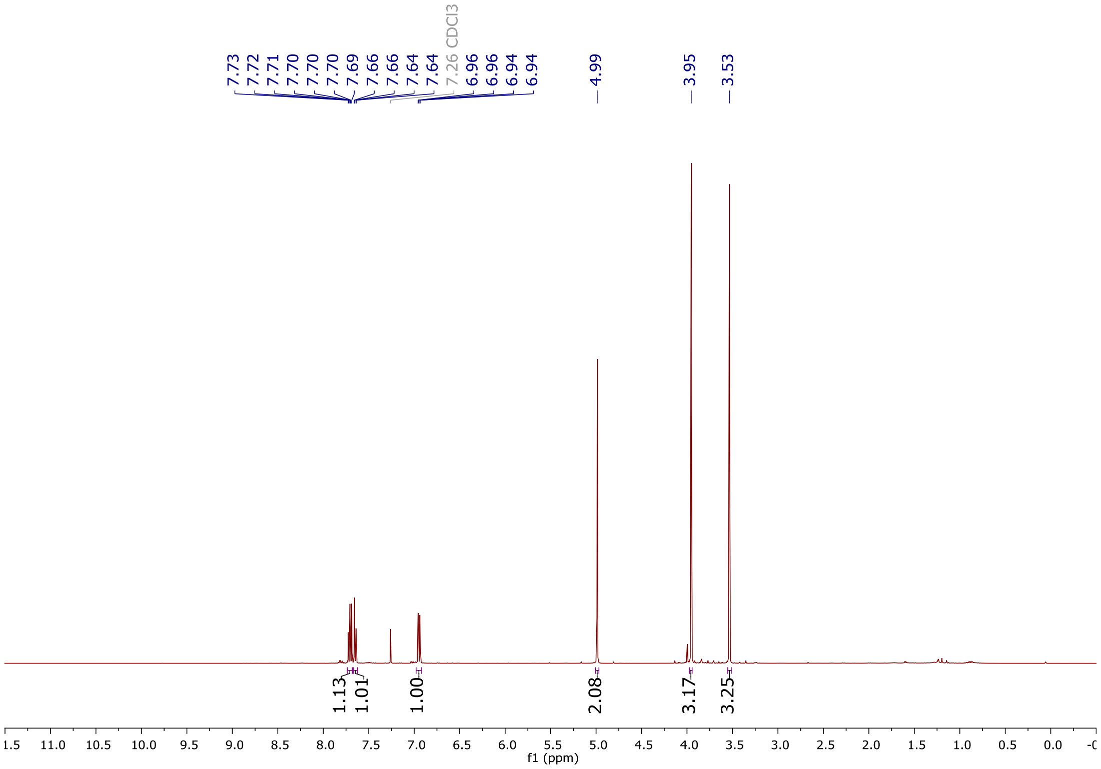

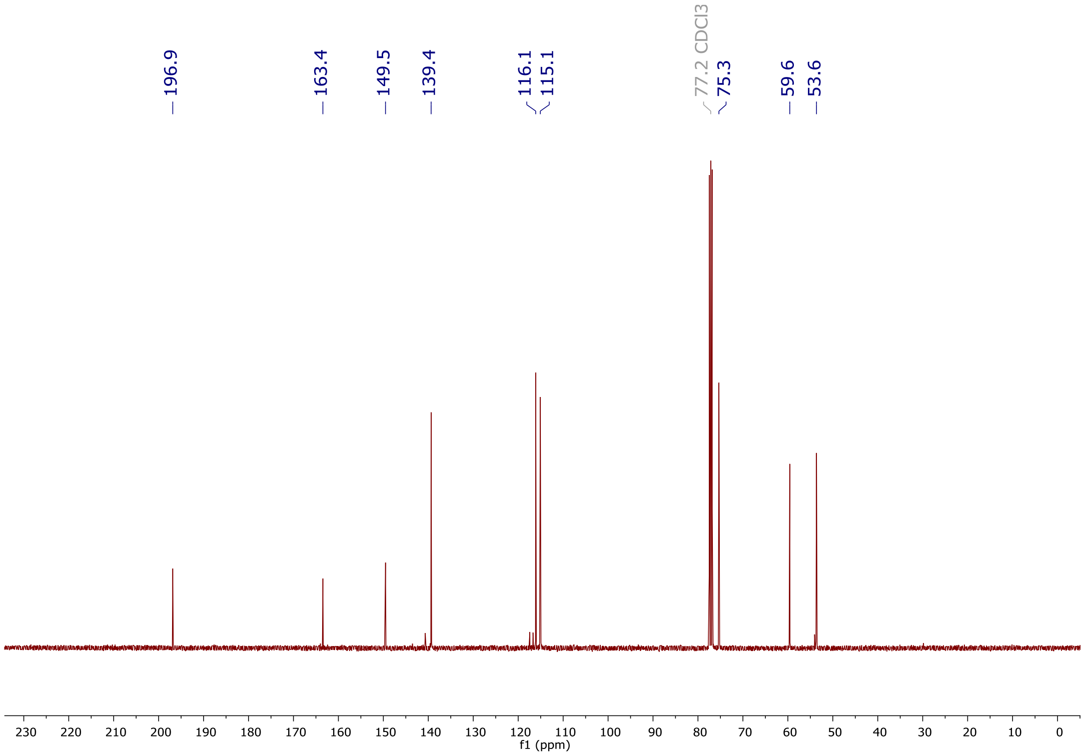

1-(Benzo[*b*]thiophen-5-yl)-2-methoxyethan-1-one (3ag)

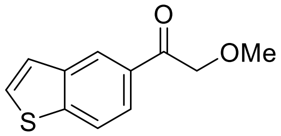

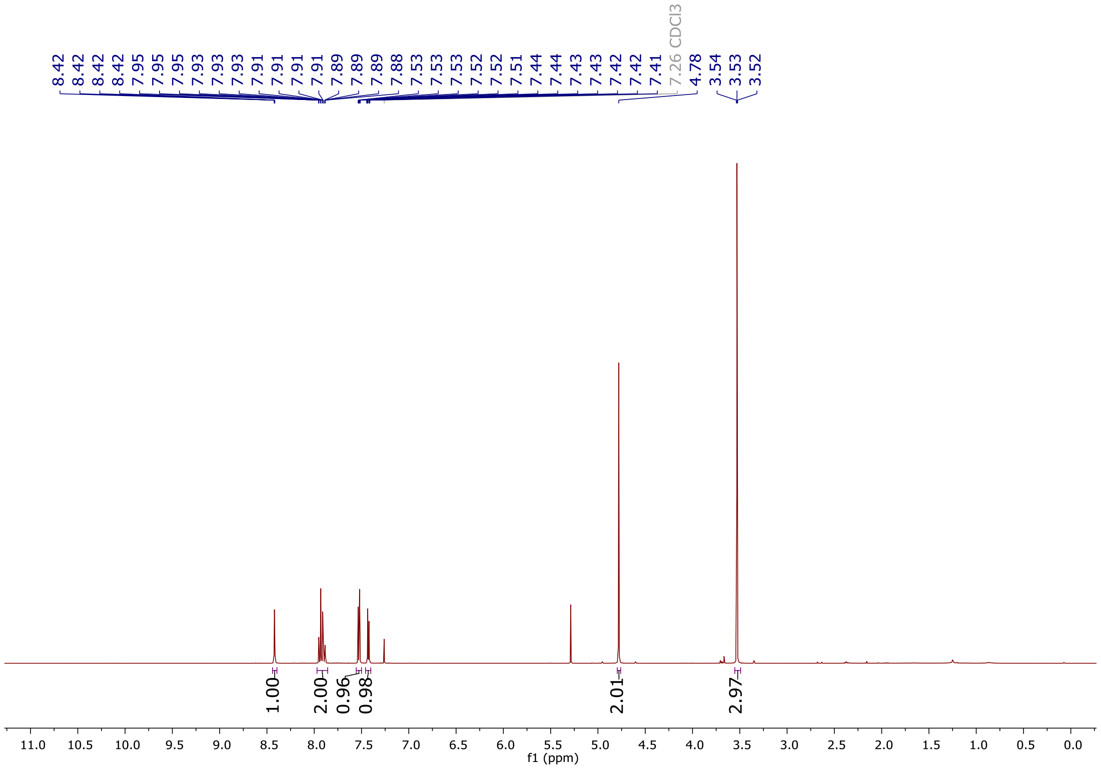

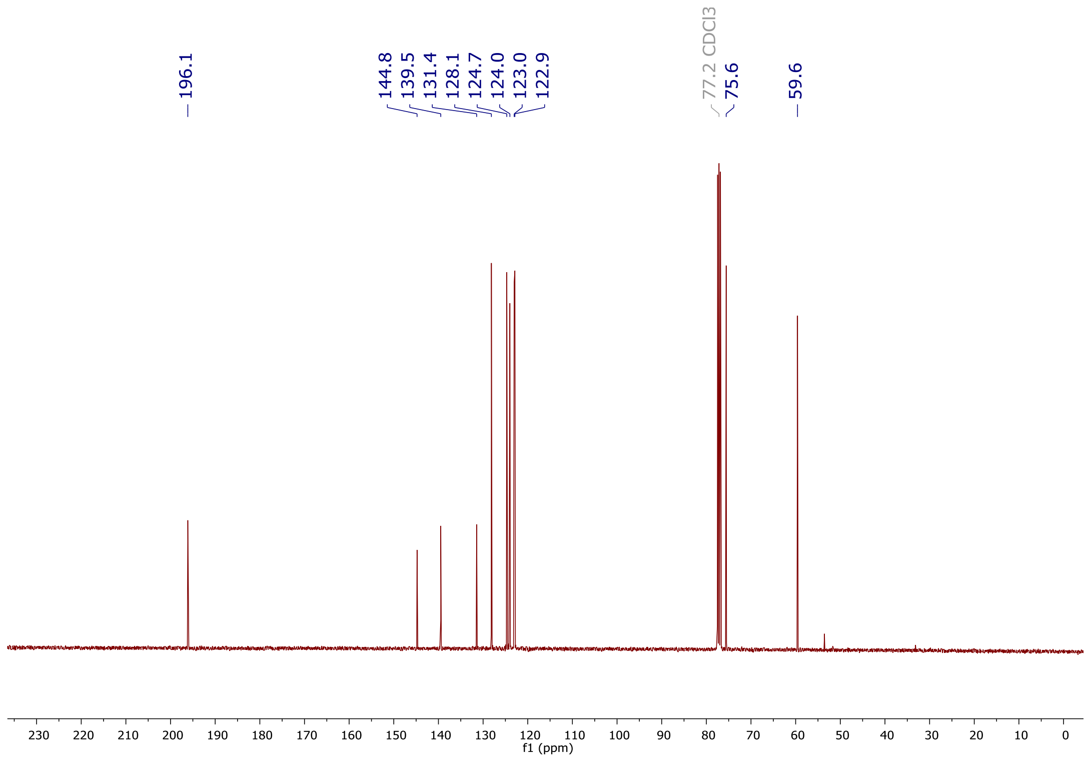

2,2-Diethoxy-1-(4-fluorophenyl)ethan-1-one (3bh)

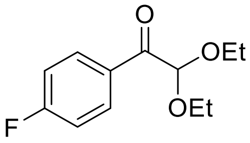

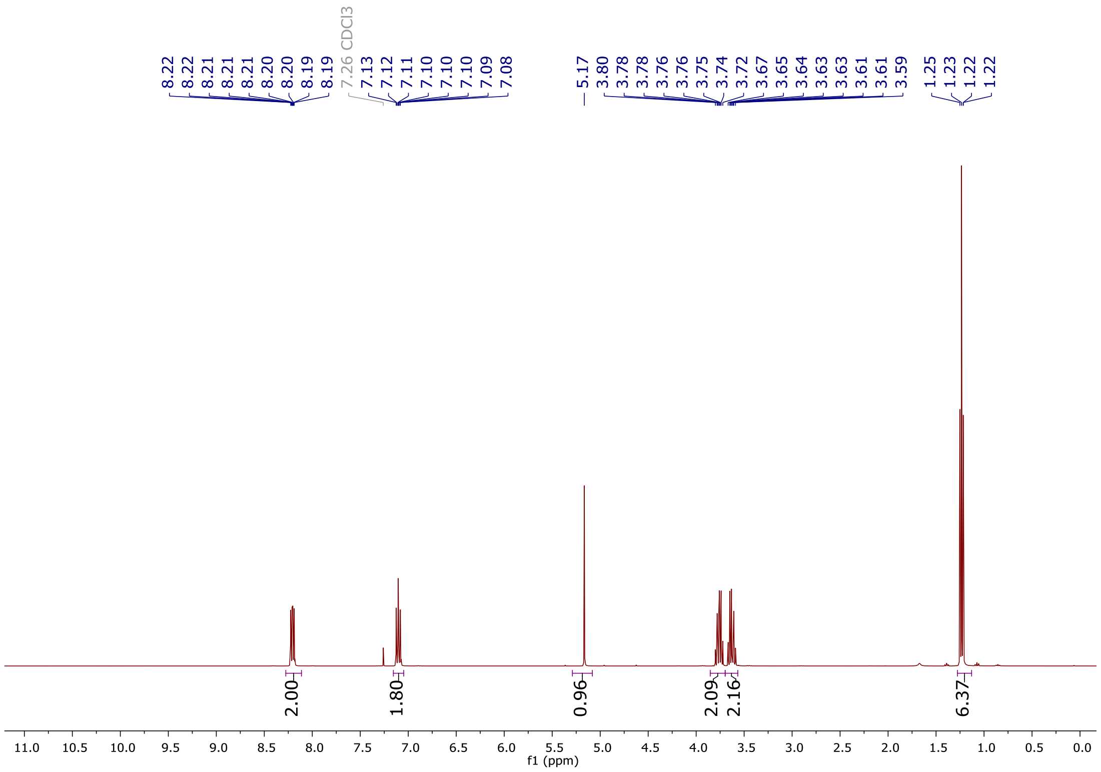

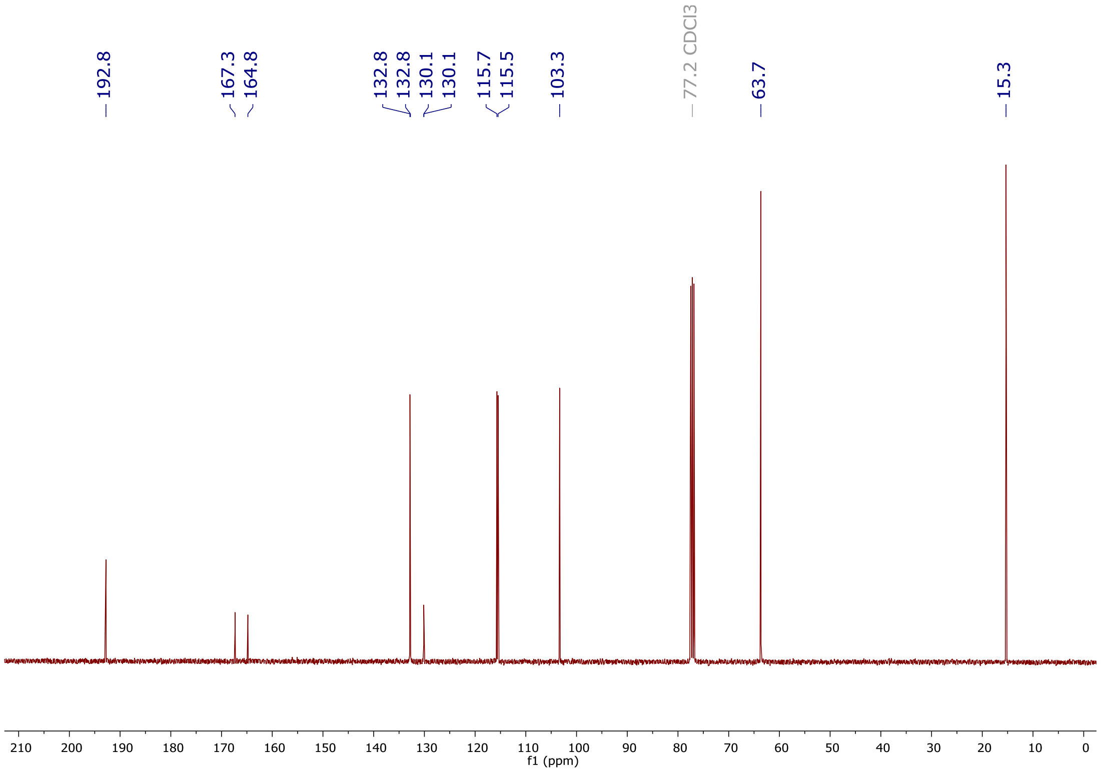

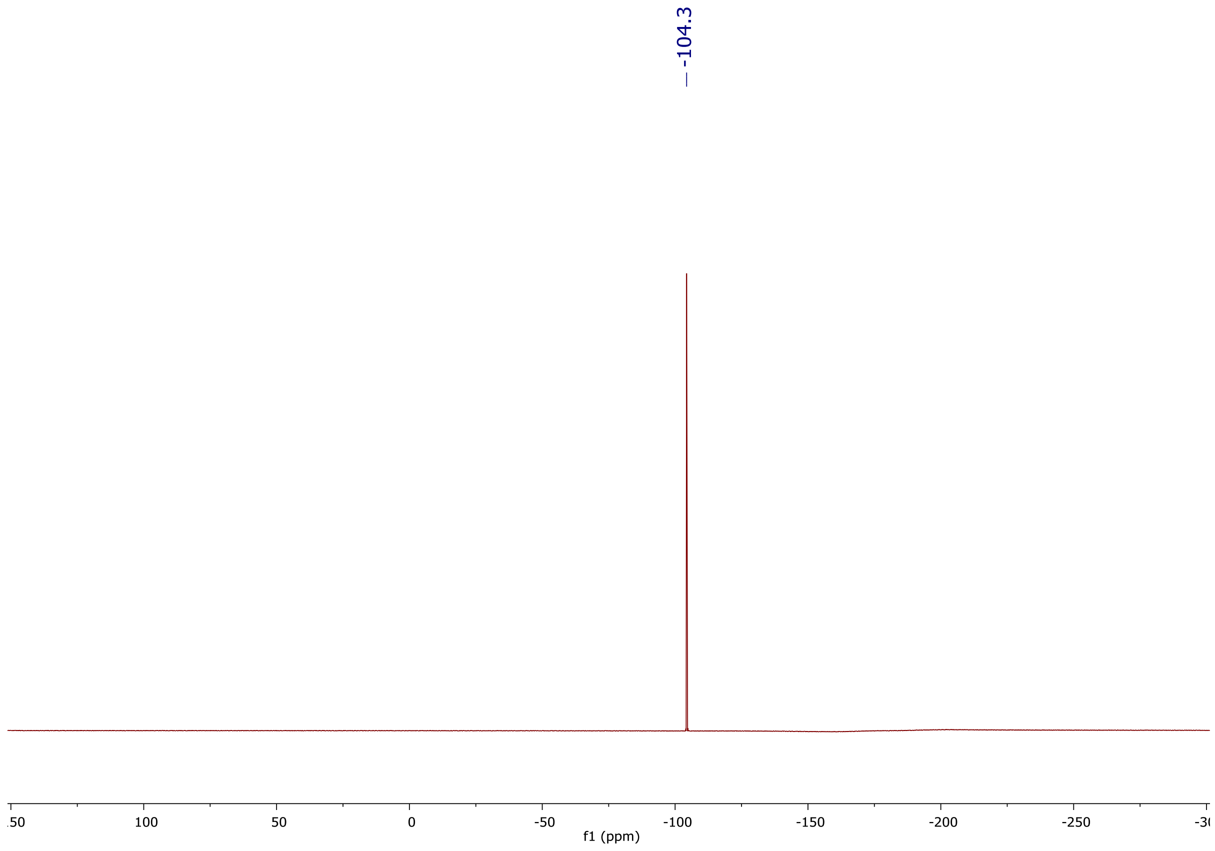
-104.3
150
100
50
0
-50
-100
-150
-200
-250
-300
f1 (ppm)

2,2-Diethoxy-1-(3-(trifluoromethyl)phenyl)ethan-1-one (3bi)

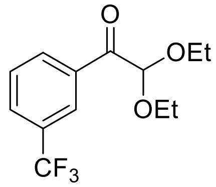

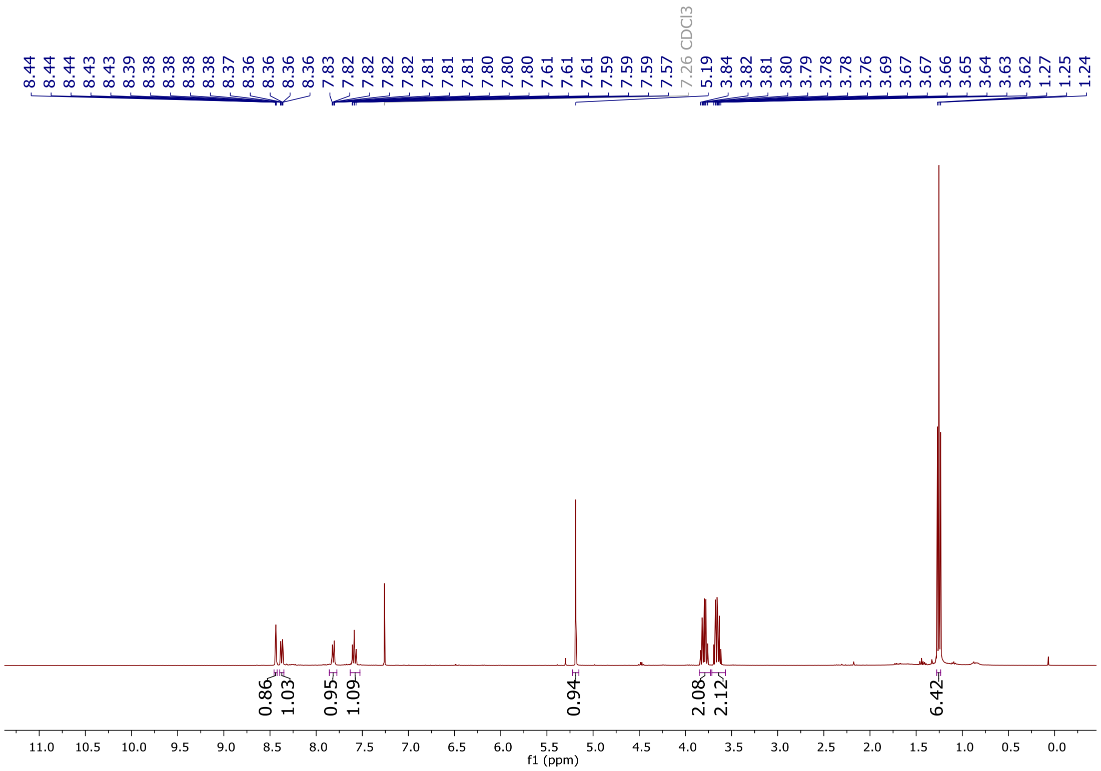

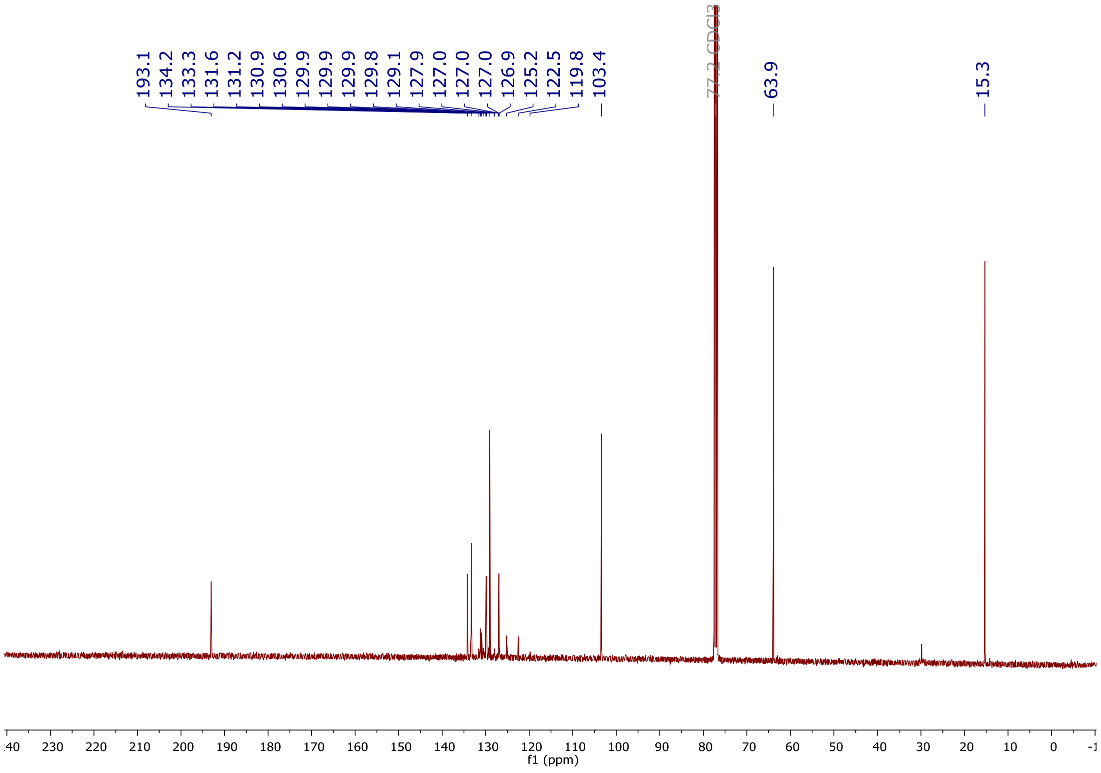

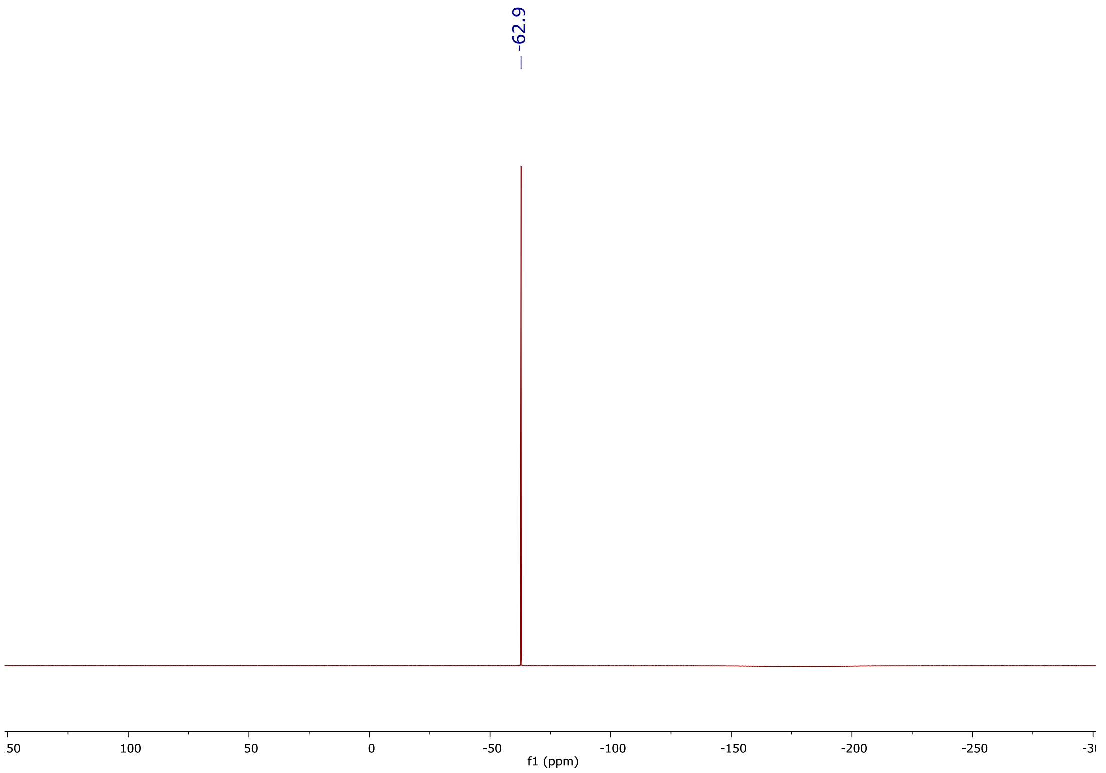
-62.9
50
100
50
0
-50
-100
-150
-200
-250
f1 (ppm)

2,2-Diethoxy-1-(4-(trifluoromethoxy)phenyl)ethan-1-one (3bj)

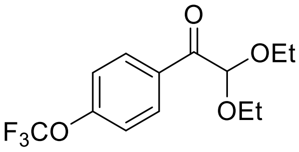

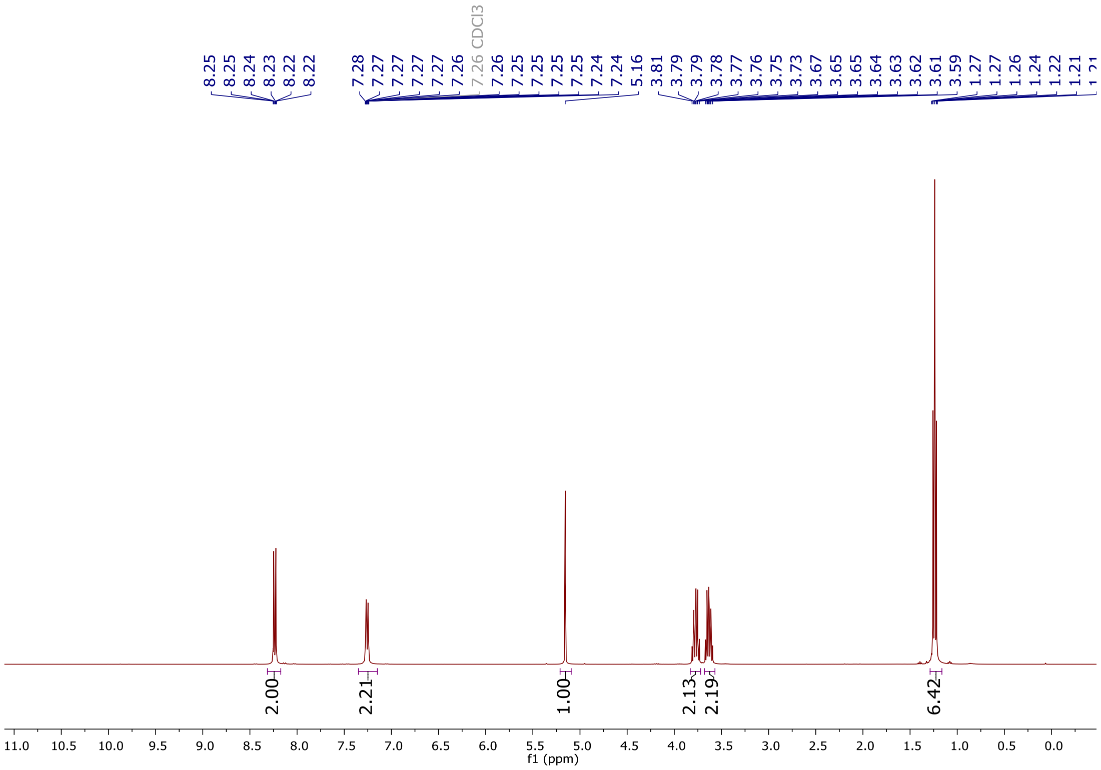

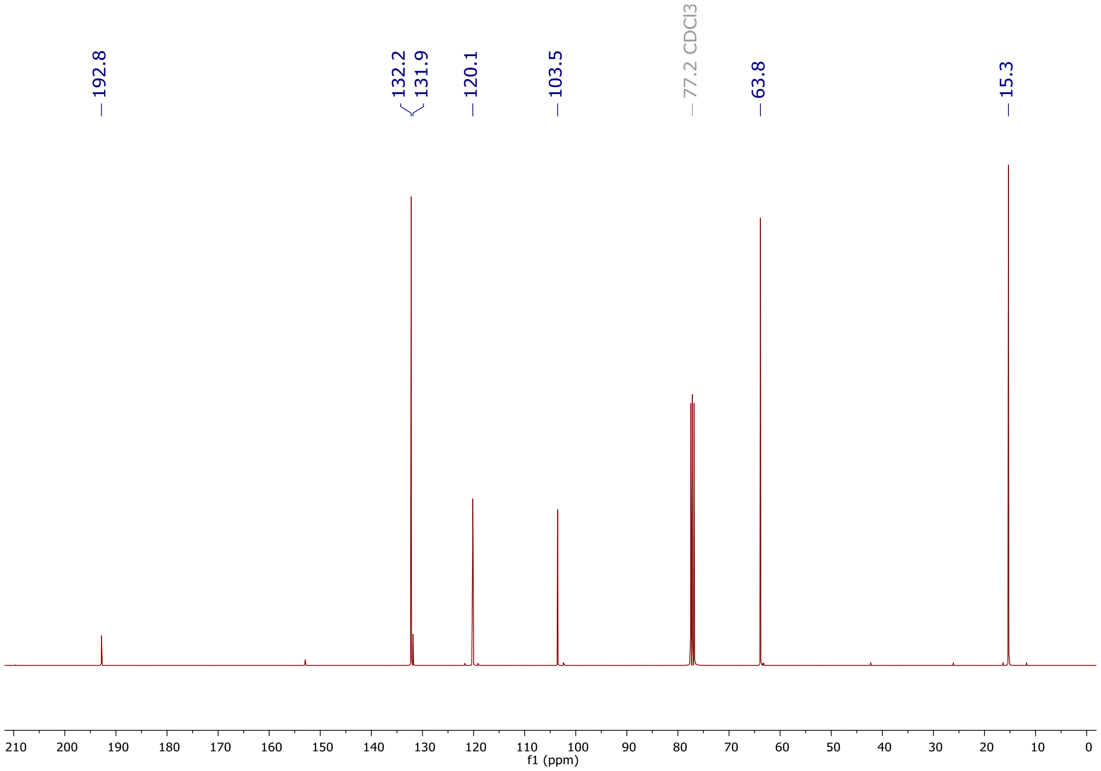

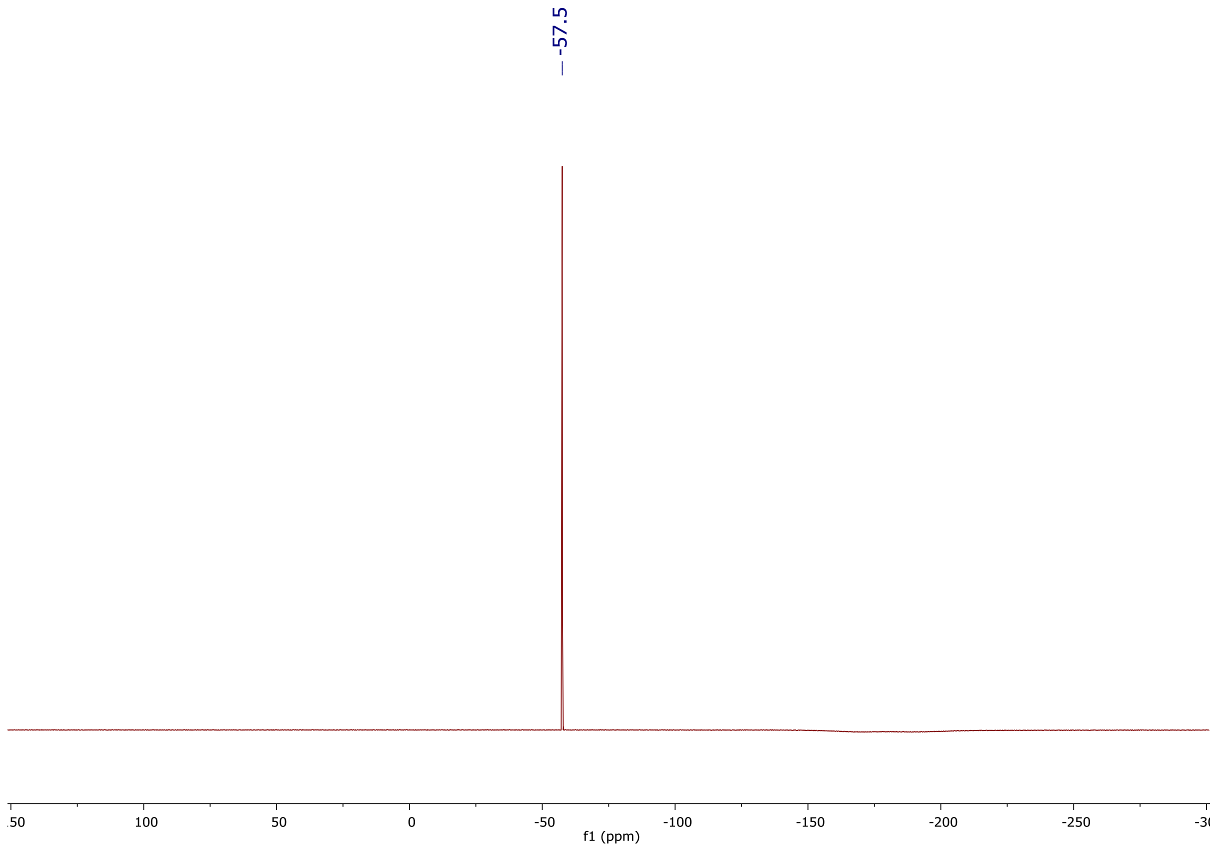

-57.5
50
100
50
0
-50
-100
-150
-200
-250
f1 (ppm)

1-(Benzo[*b*]thiophen-5-yl)-2-fluoroethan-1-one (3cg)

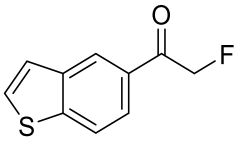

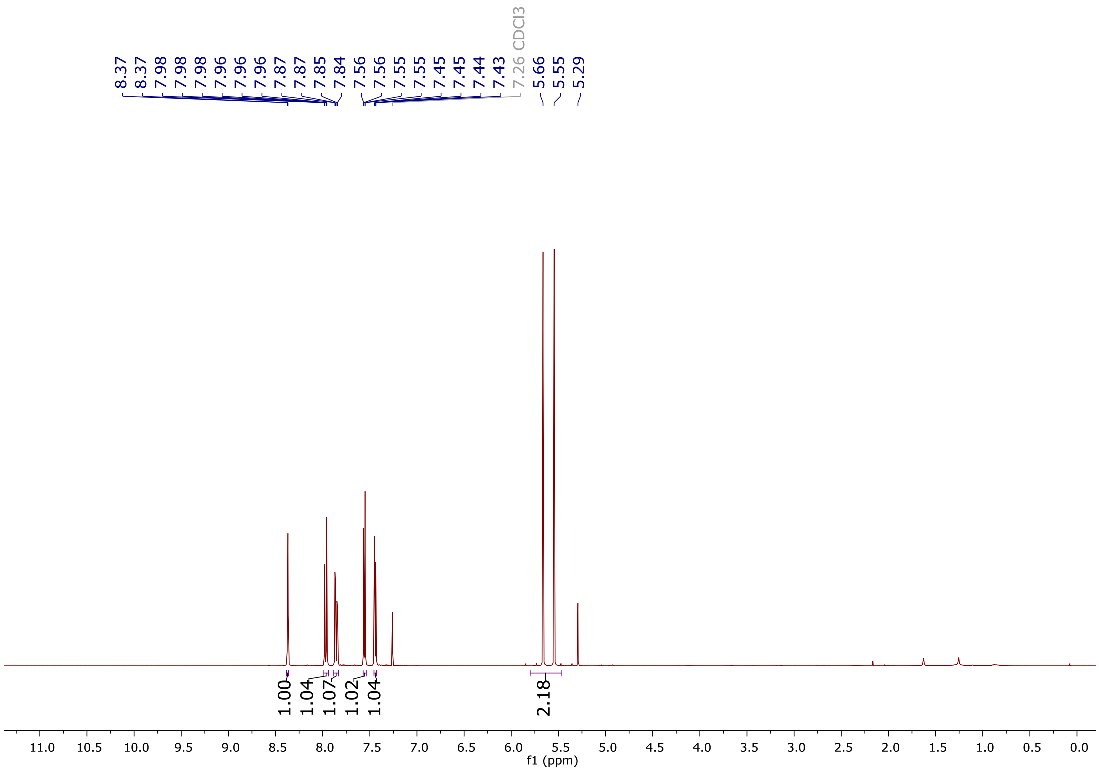

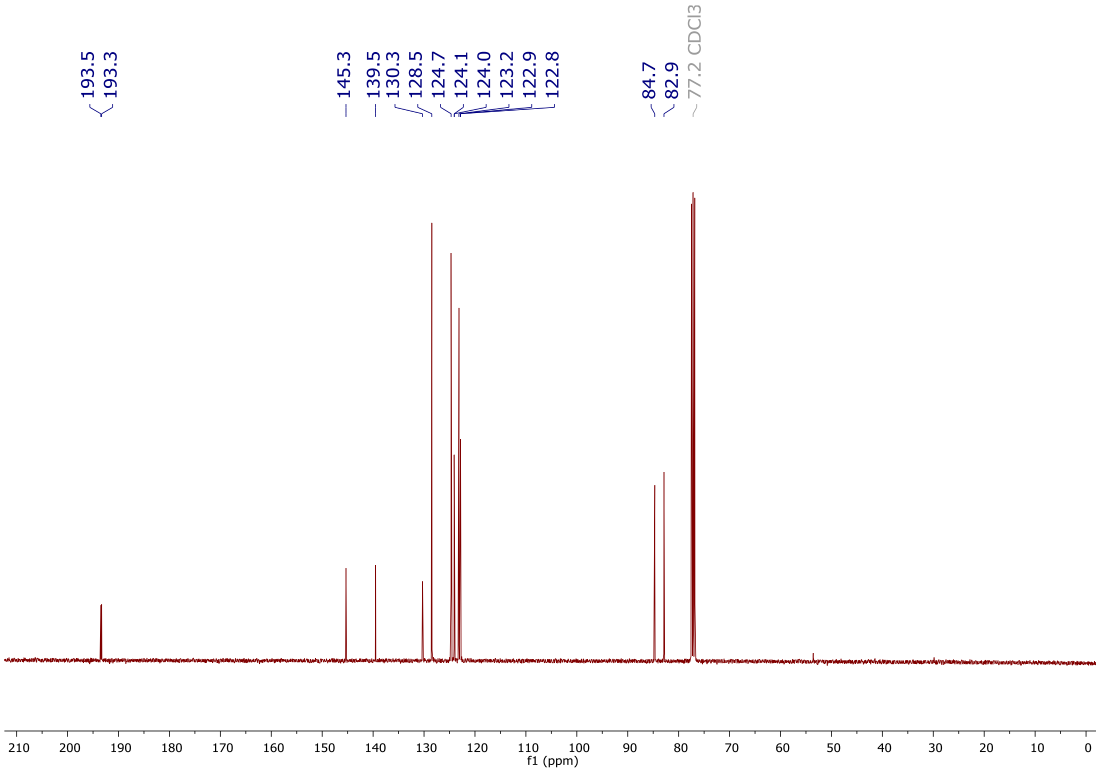

2-Fluoro-1-(4-fluorophenyl)ethan-1-one (3ch)

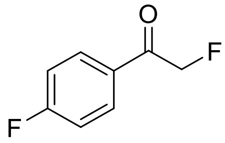

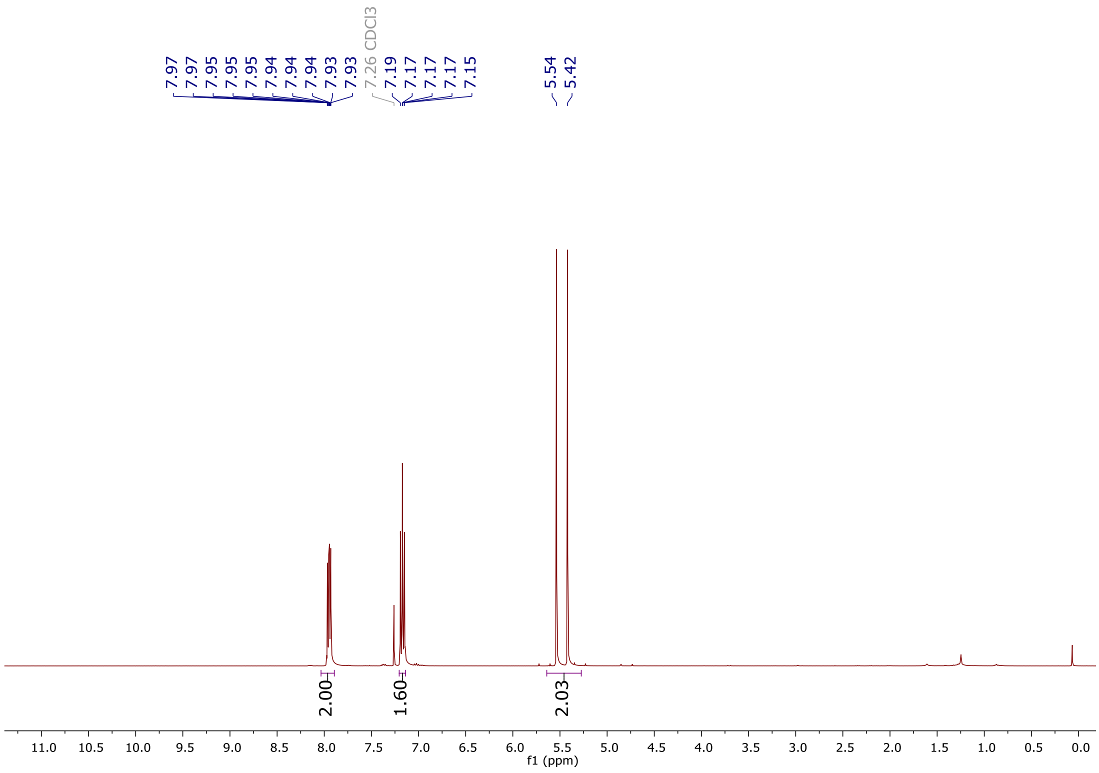

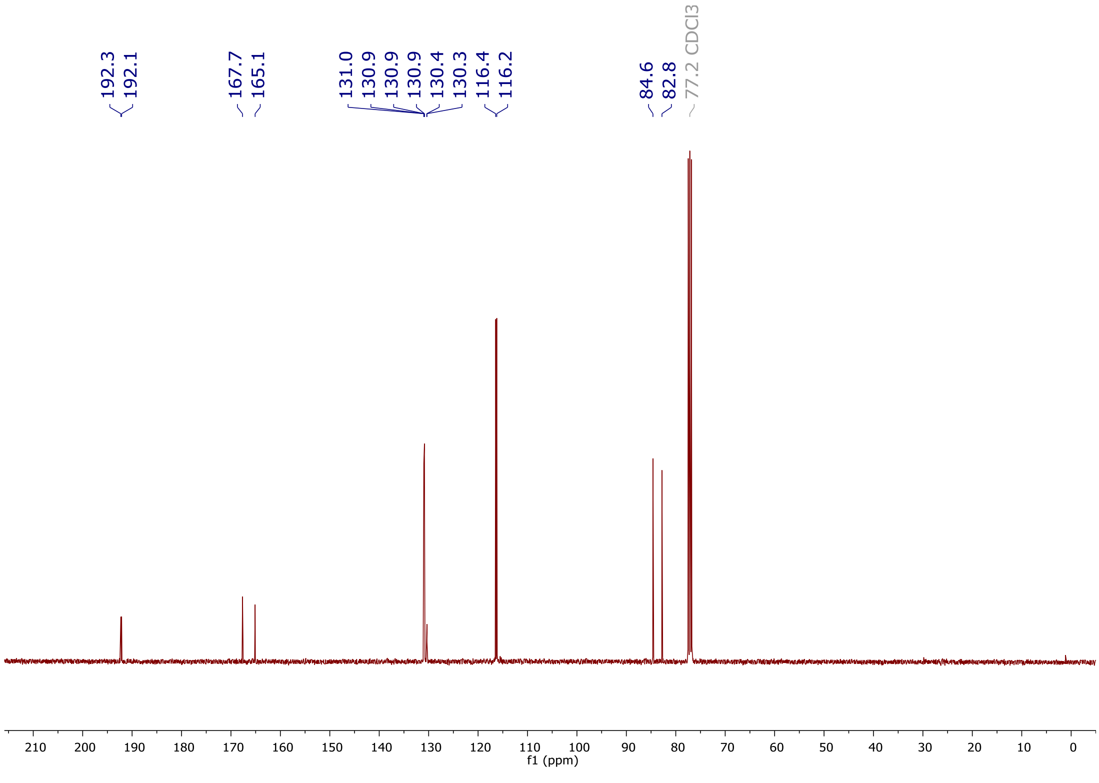

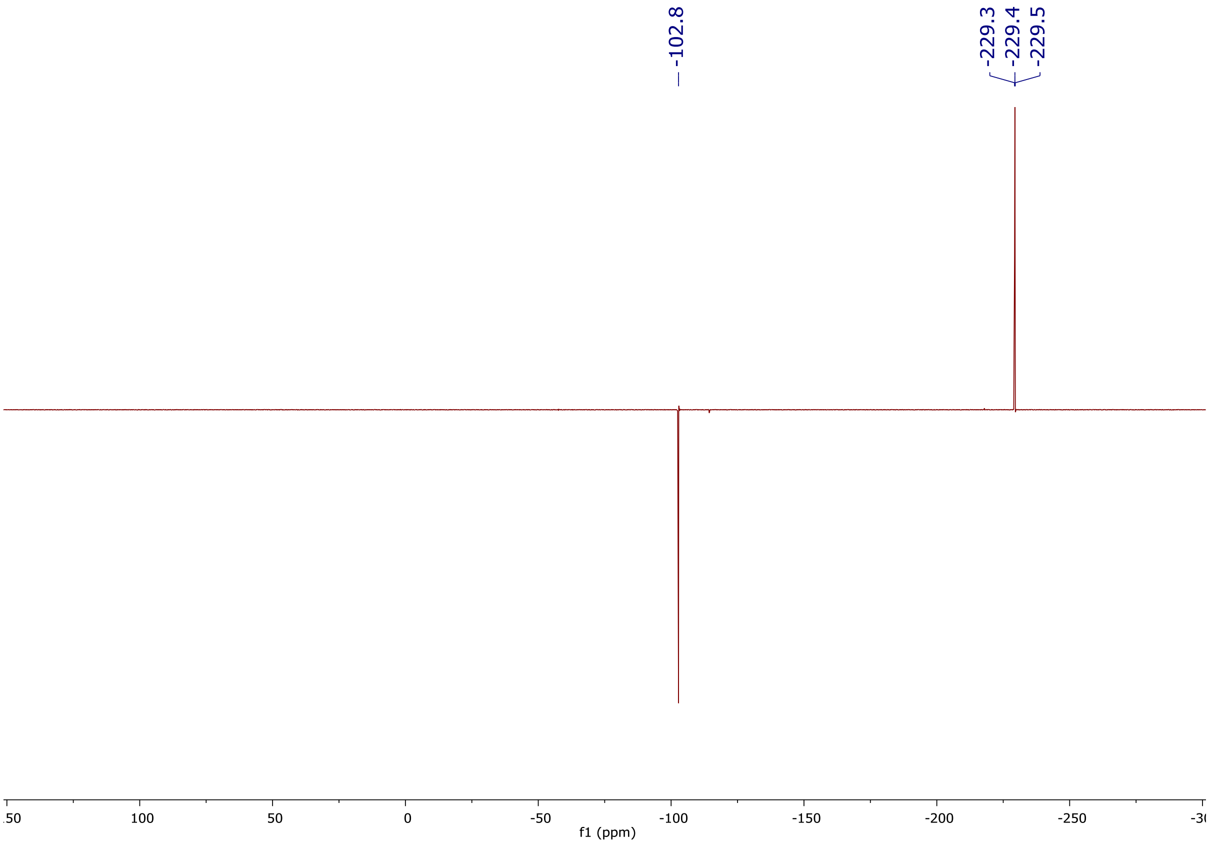

-102.8
-229.3
-229.4
-229.5
150
100
50
0
-50
-100
-150
-200
-250
-300
f1 (ppm)

2-Fluoro-1-(4-(trifluoromethoxy)phenyl)ethan-1-one (3cj)

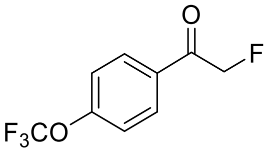

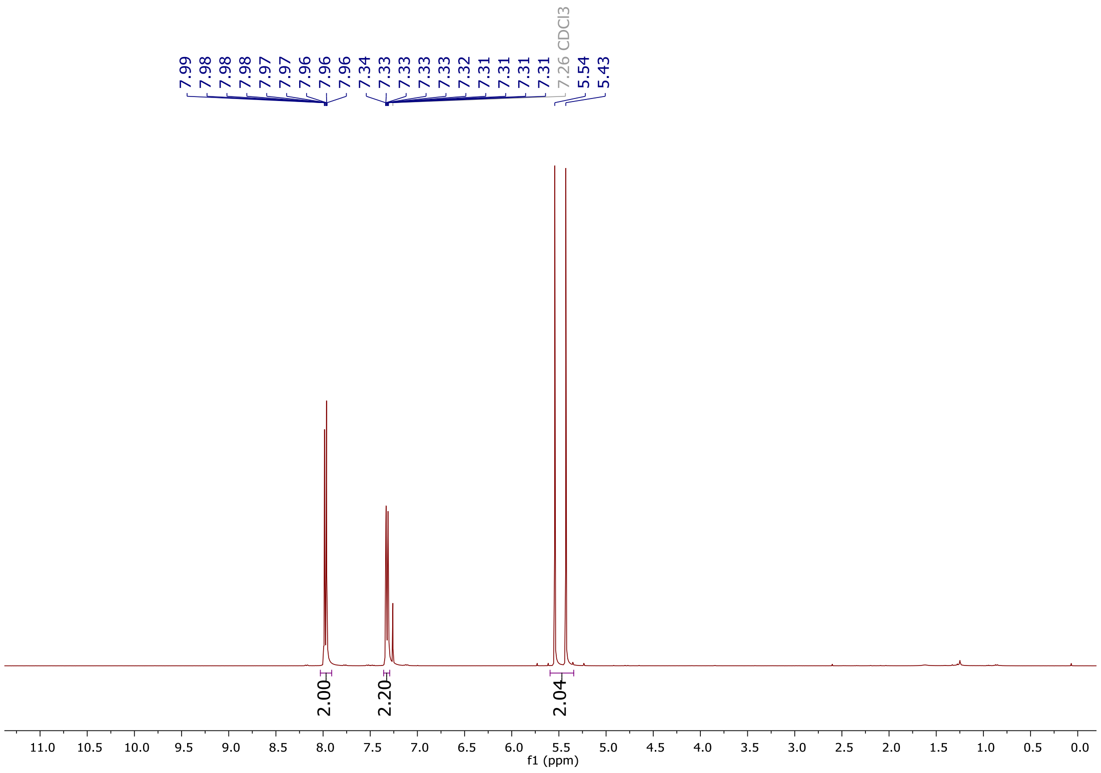

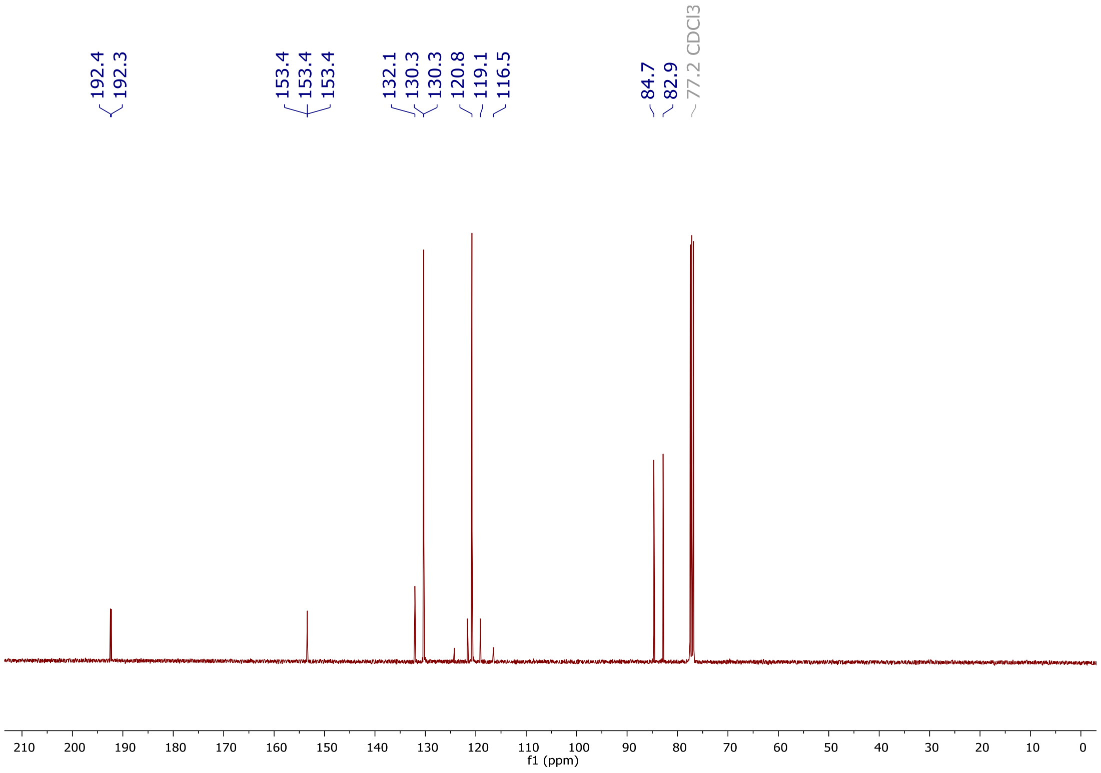

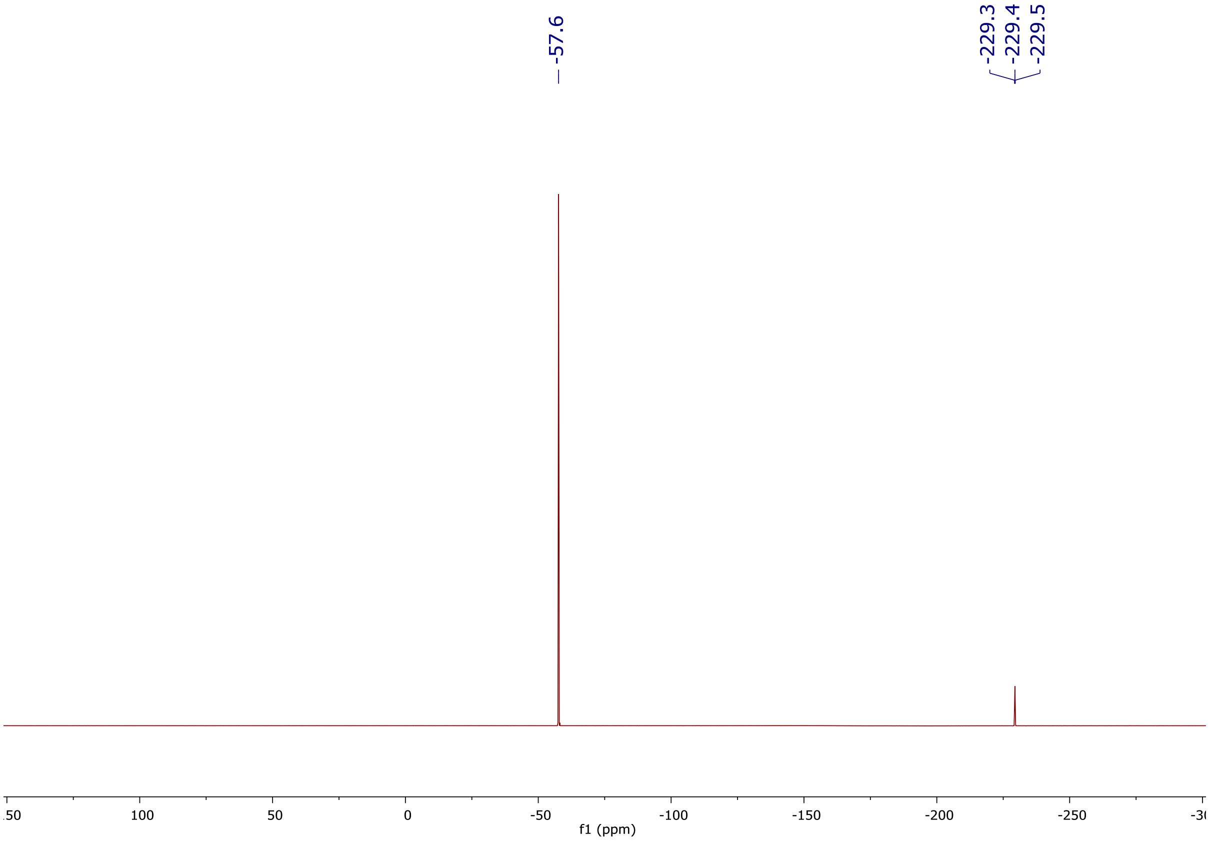

-57.6
-229.3
-229.4
-229.5
100
50
0
-50
-100
-150
-200
-250
f1 (ppm)

2-Fluoro-1-(3-(trifluoromethyl)phenyl)ethan-1-one (3ci)

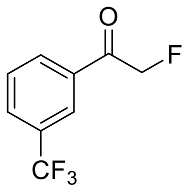

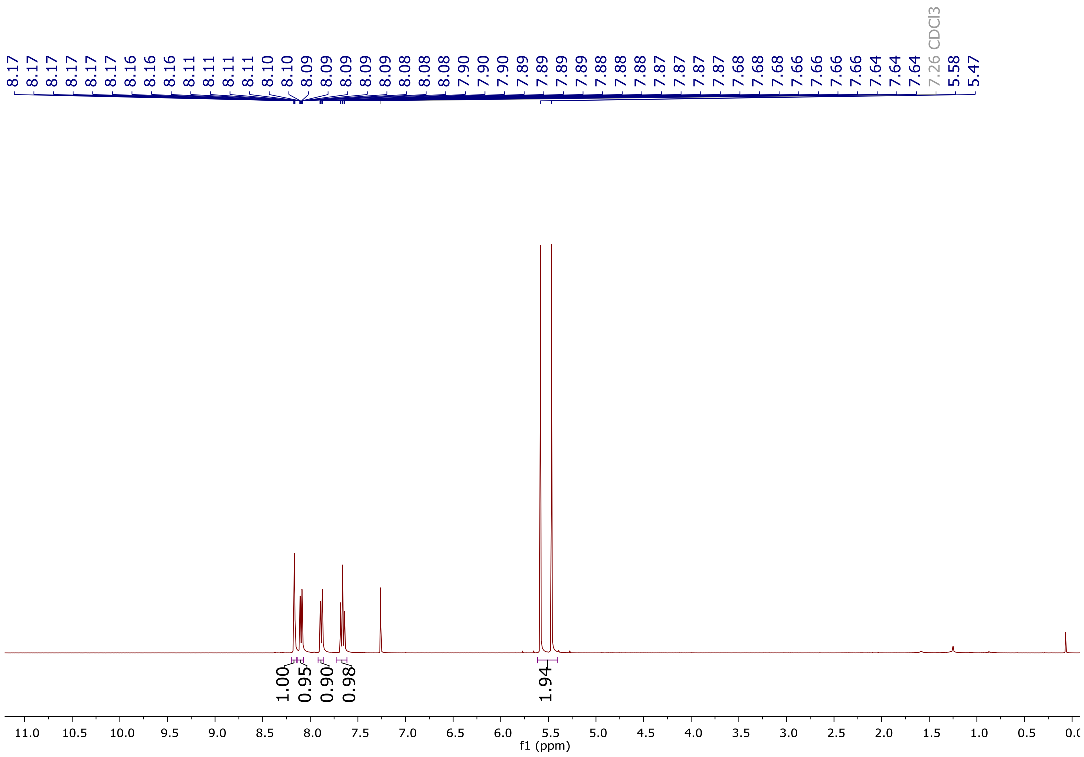

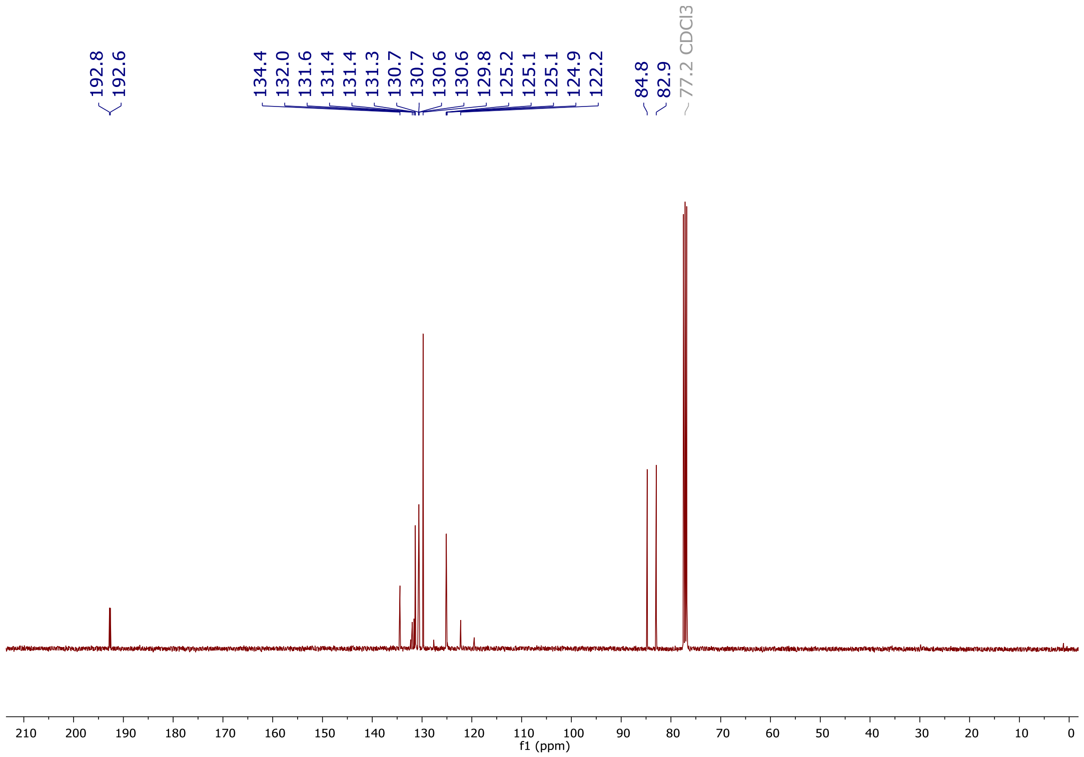

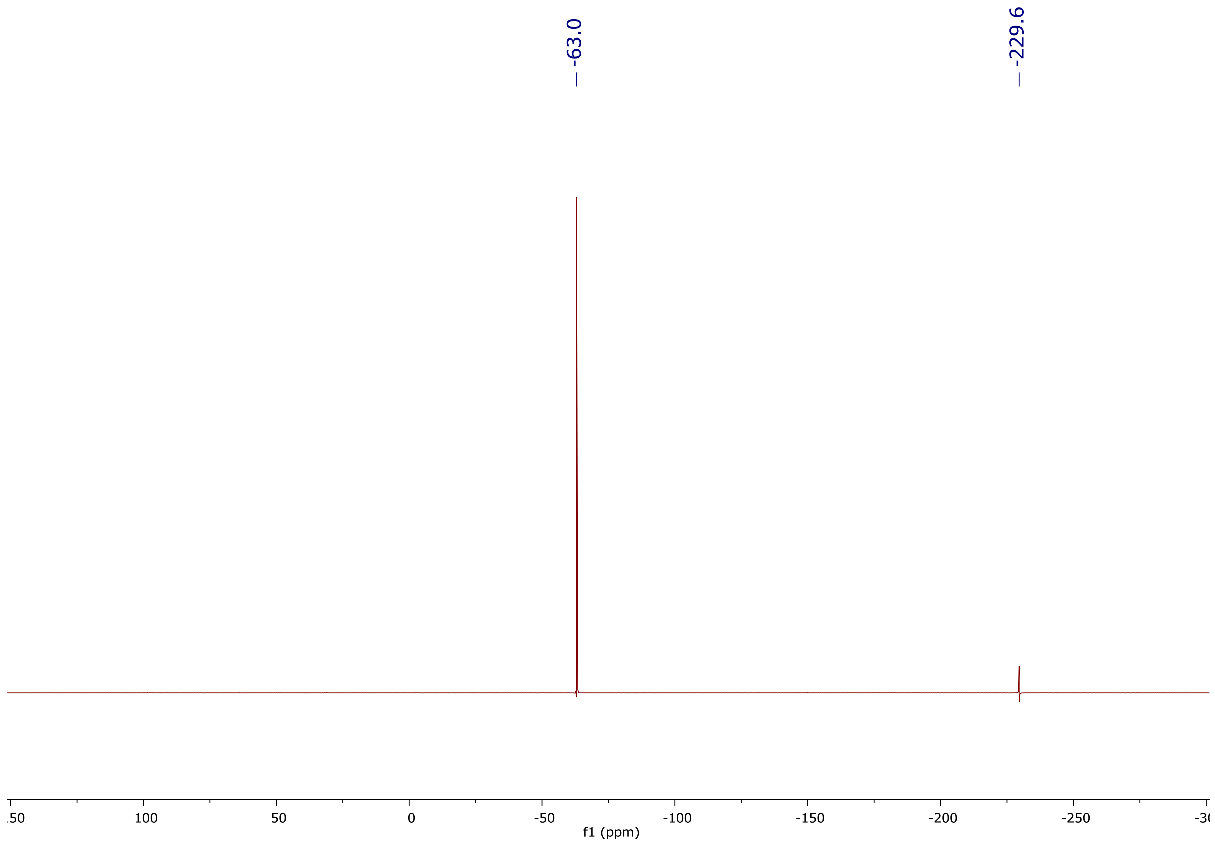
-63.0
-229.6
50
100
50
0
-50
-100
-150
-200
-250
f1 (ppm)

2,2-Difluoro-1-(4-(methylthio)phenyl)ethan-1-one (3da)

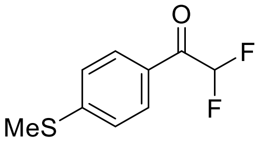

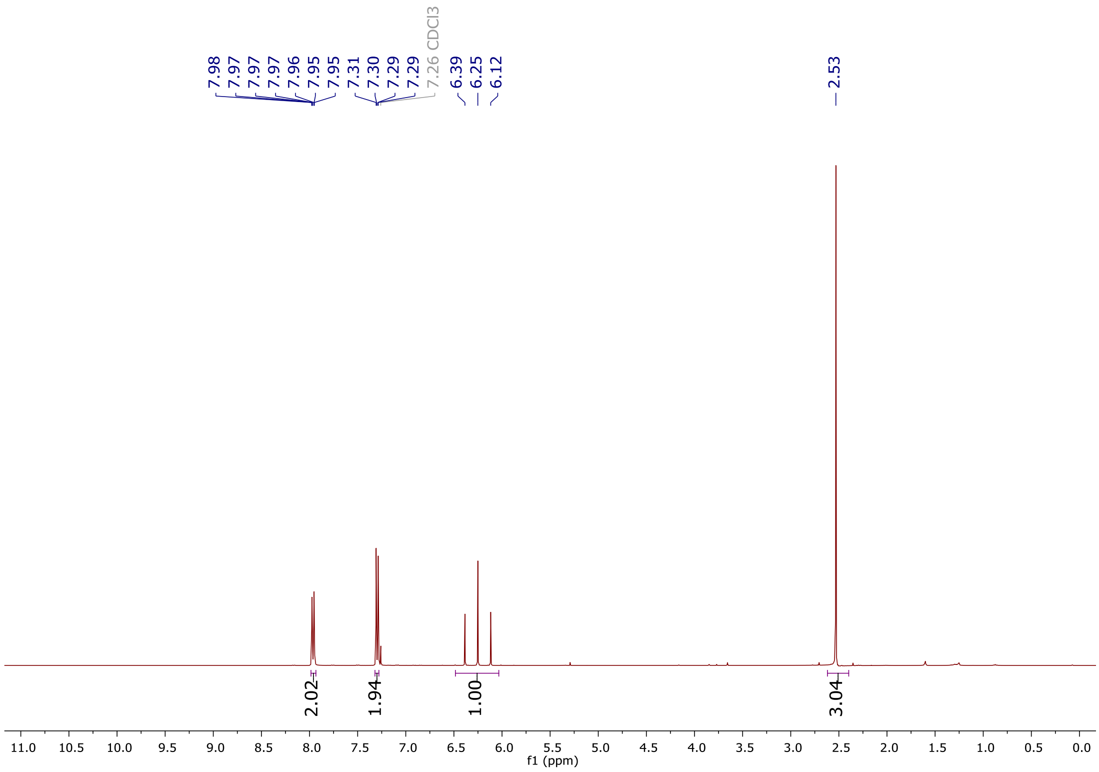

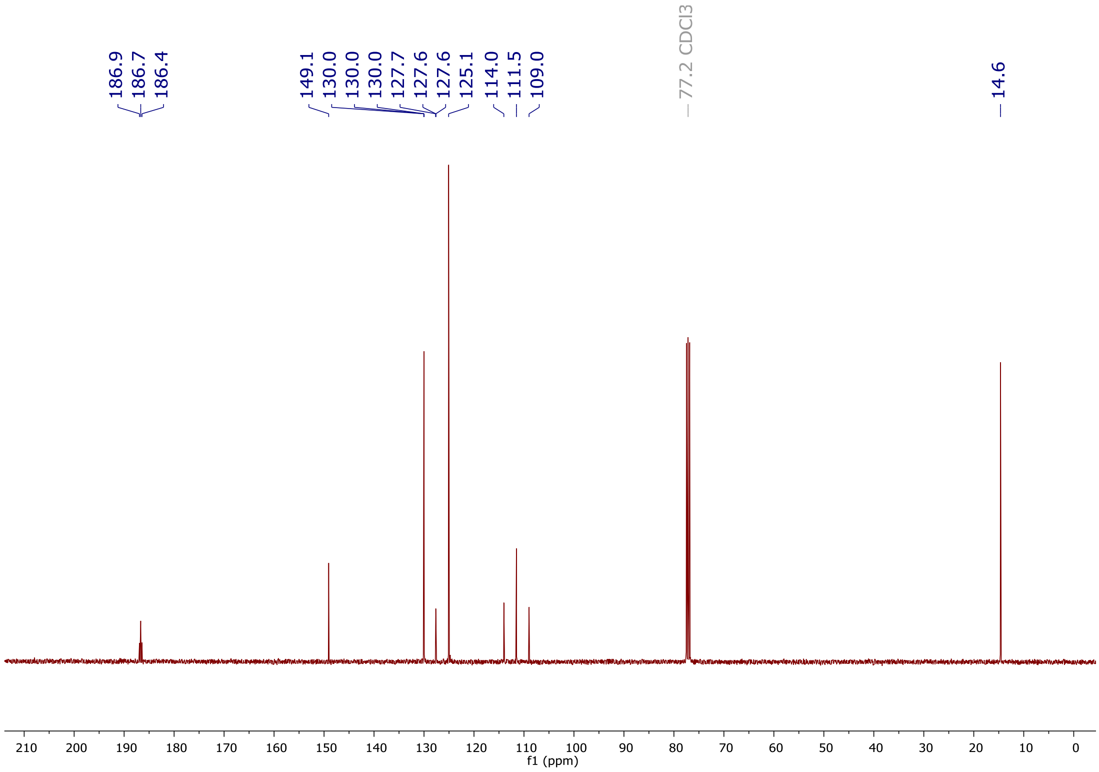

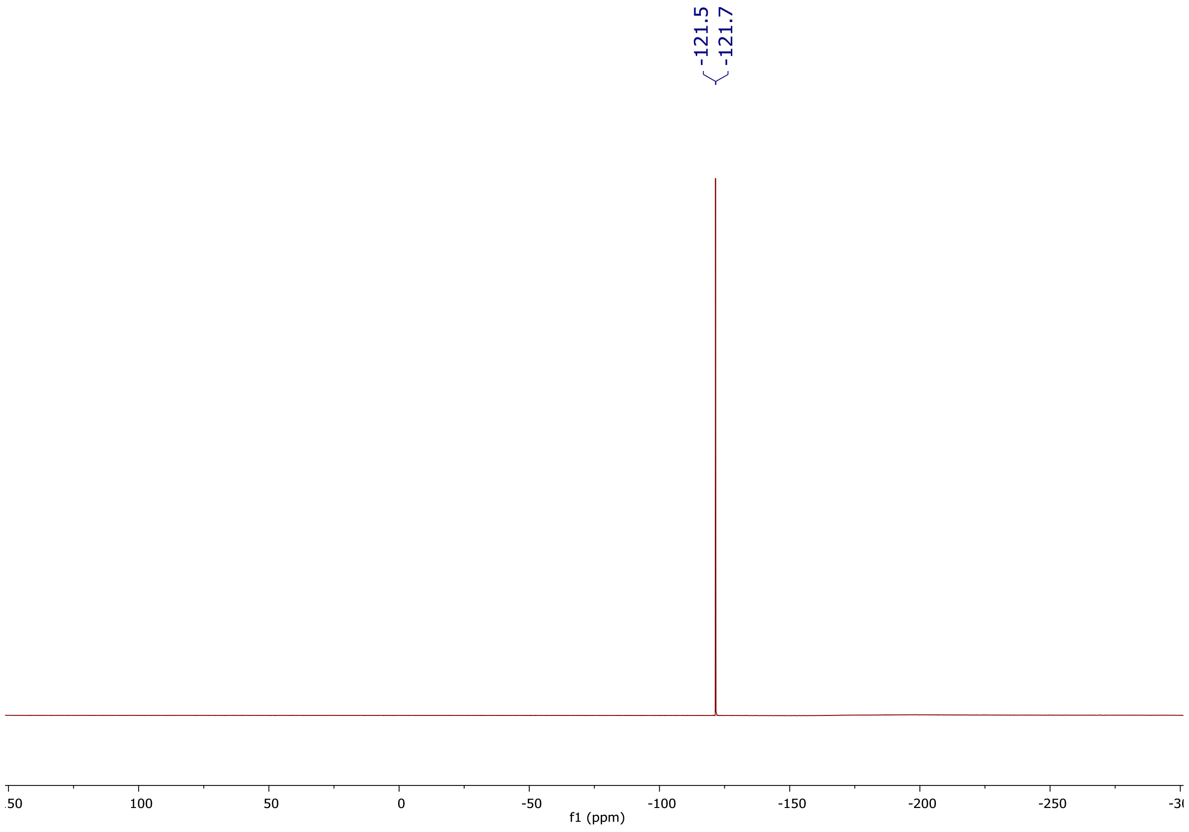

-121.5
-121.7
150
100
50
0
-50
-100
-150
-200
-250
-300
f1 (ppm)

2,2-difluoro-1-(4-methoxyphenyl)ethan-1-one (3dk)

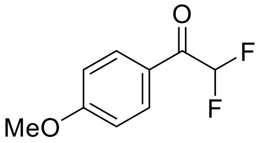

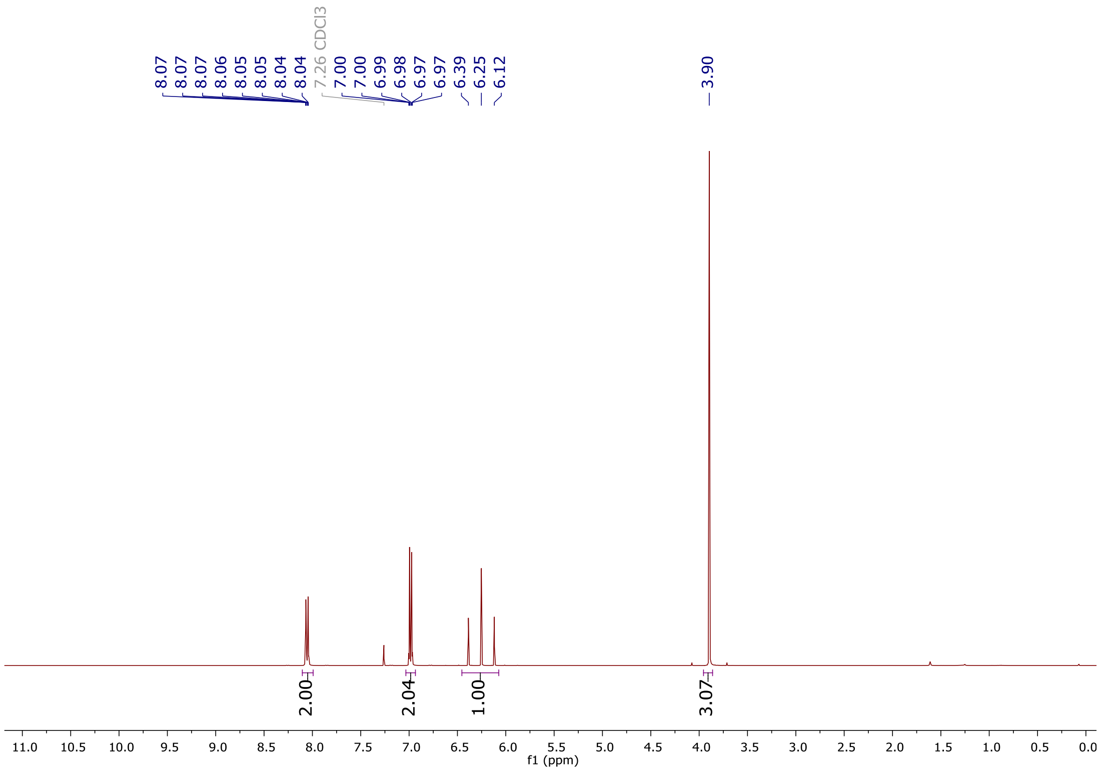

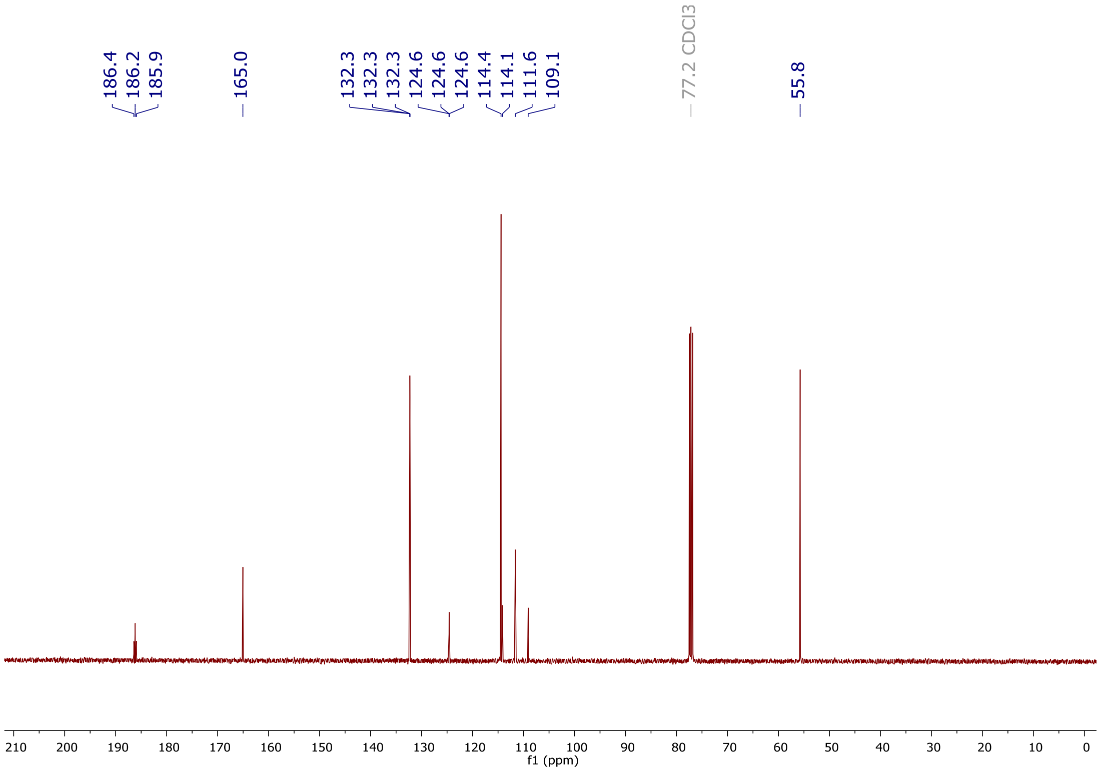

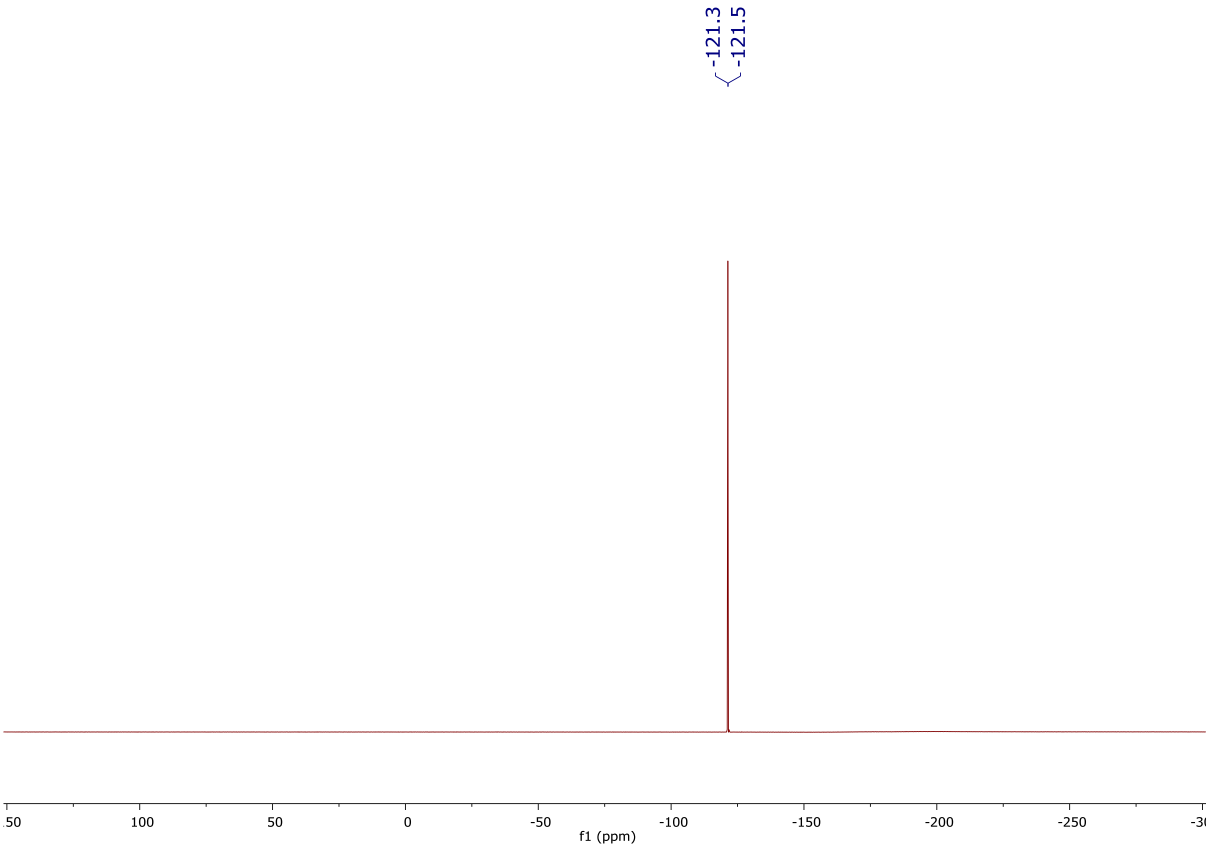

-121.3
-121.5
150
100
50
0
-50
-100
-150
-200
-250
f1 (ppm)

2,2-Difluoro-1-(3-((5-(4-fluorophenyl)thiophen-2-yl)methyl)-4-methylphenyl)ethan-1-one (3dl)

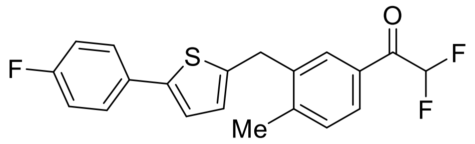

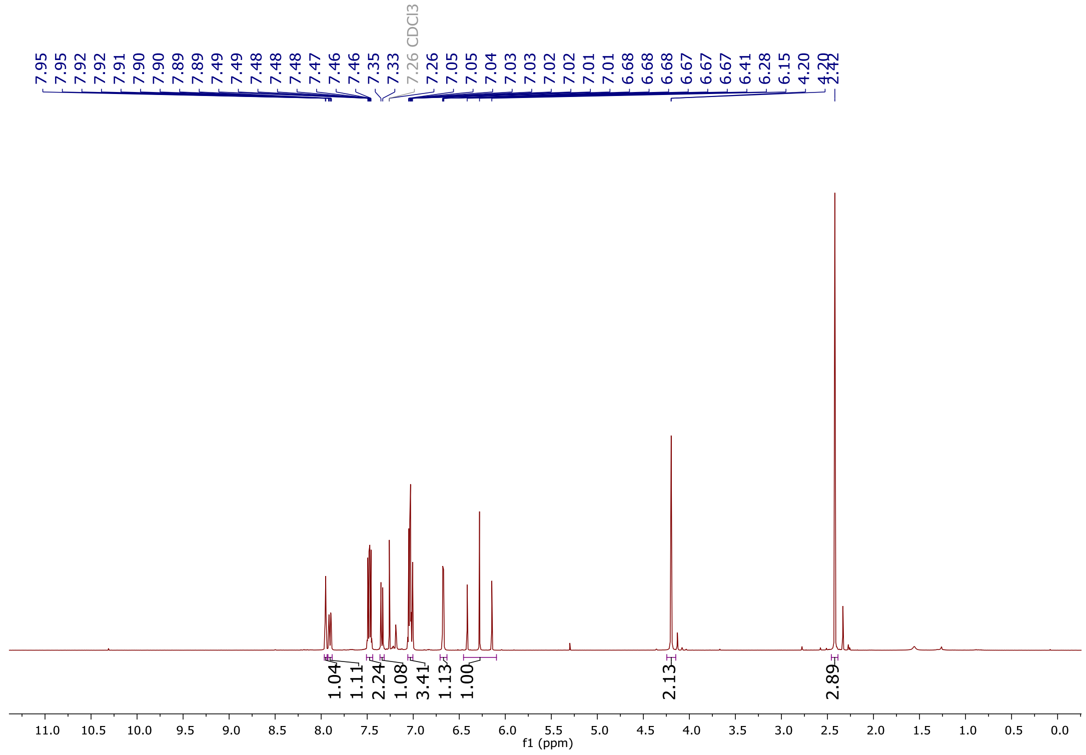

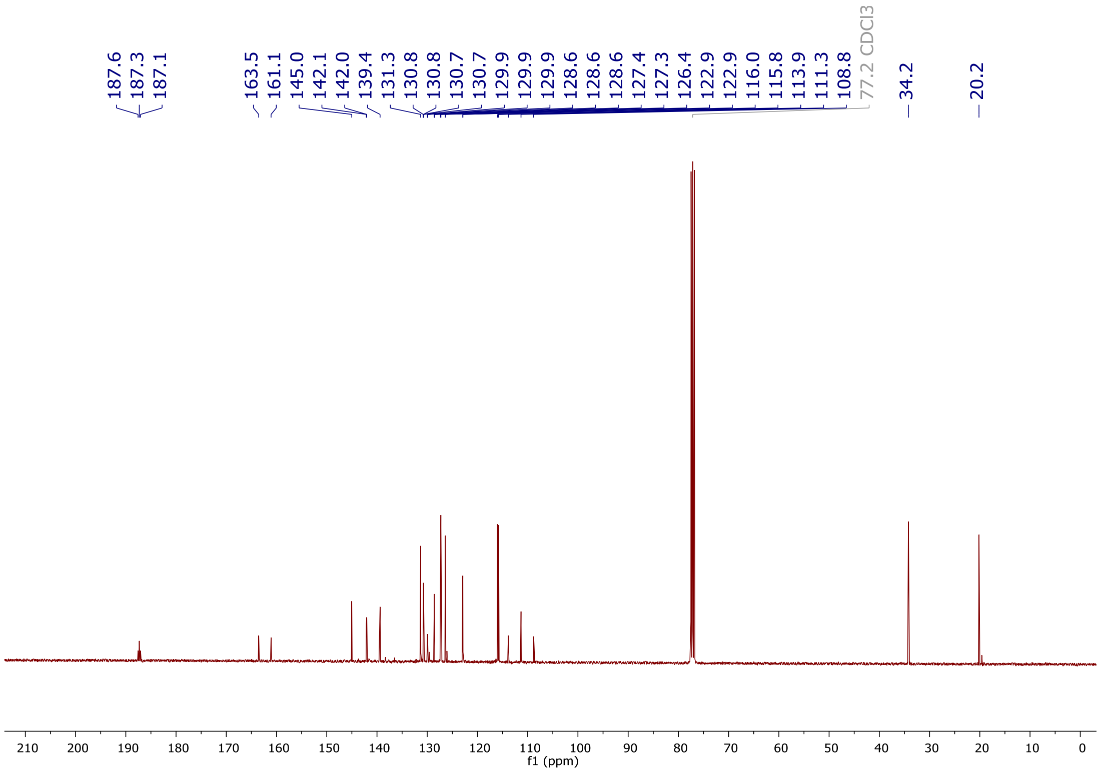

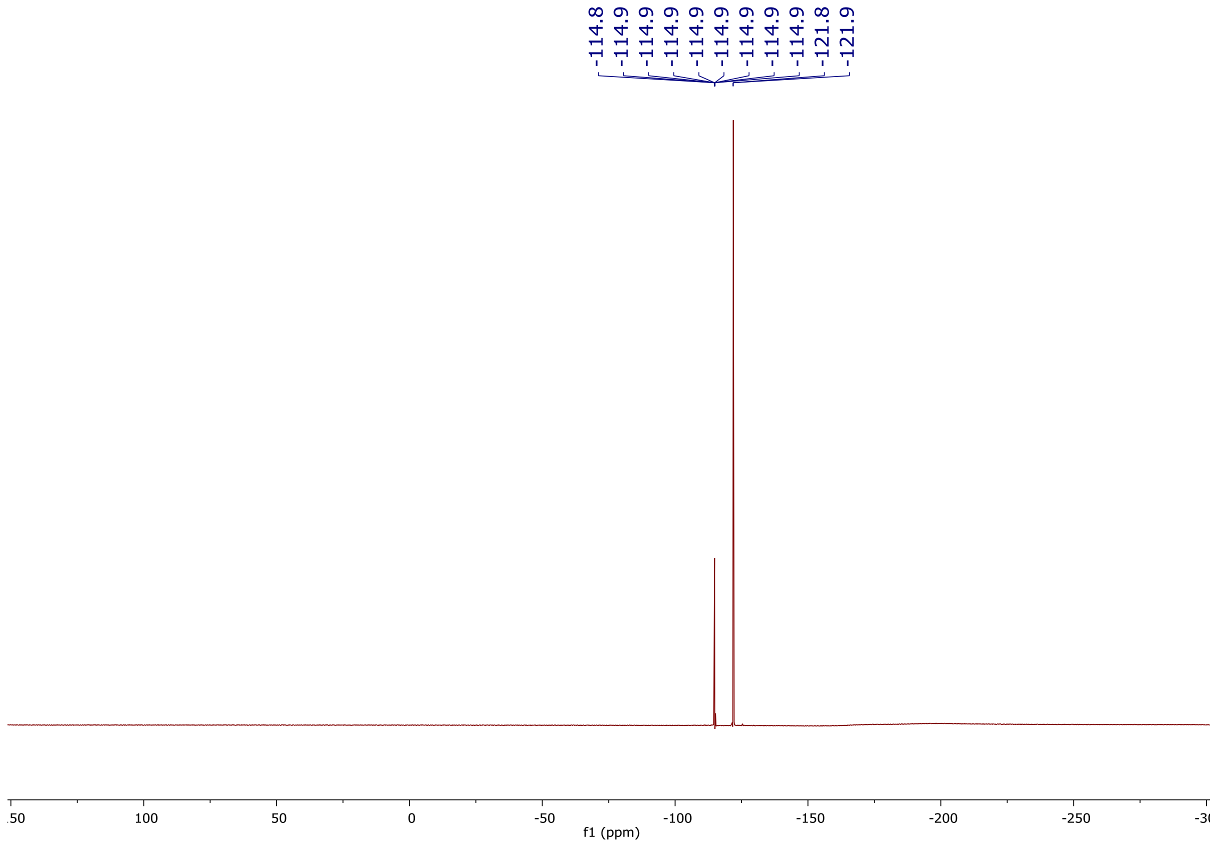

-114.8
-114.9
-114.9
-114.9
-114.9
-114.9
-114.9
-114.9
-114.9
-121.8
-121.9
f1 (ppm)

2-Chloro-1-(6-methoxypyridin-2-yl)ethan-1-one (3ef)

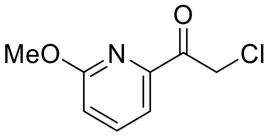

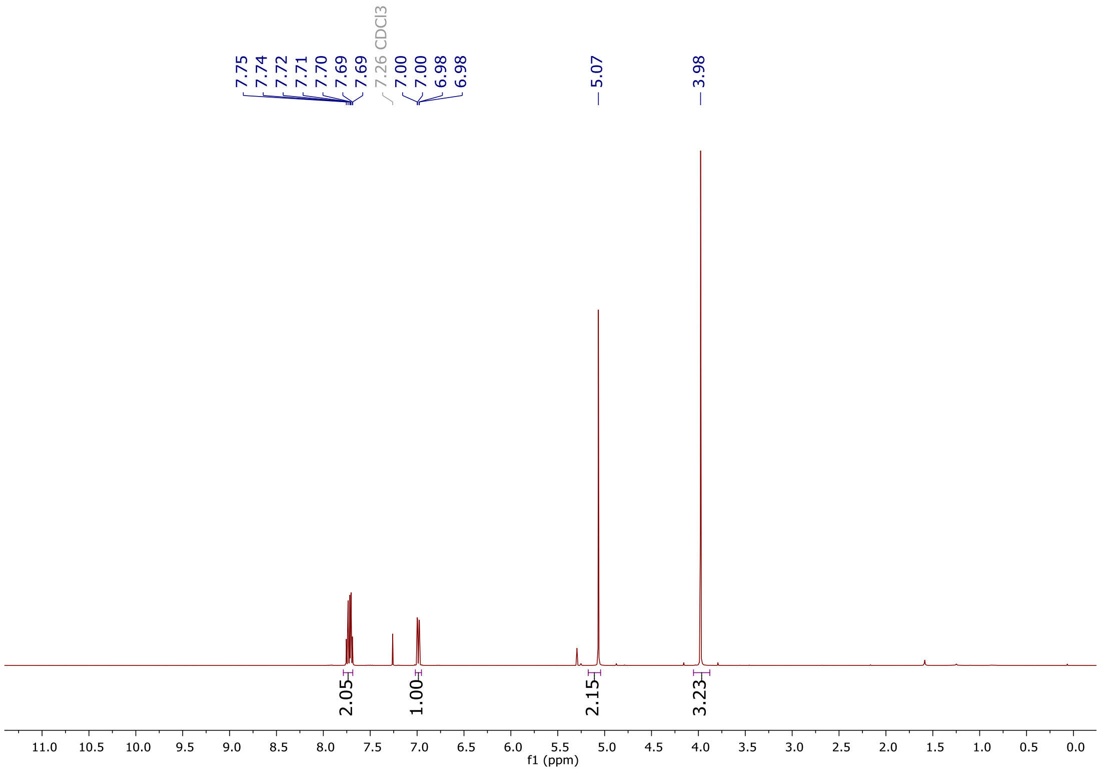

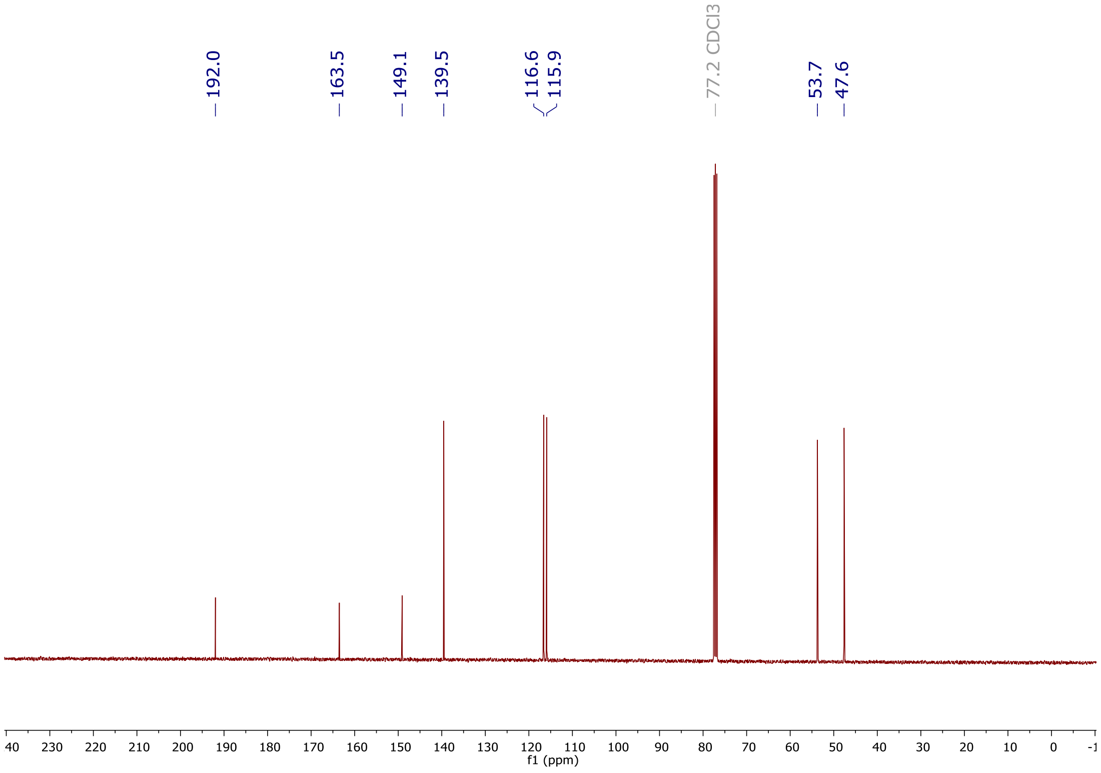

3-(2-((*Tert*-butyldimethylsilyl)oxy)phenyl)-1-(6-methoxypyridin-2-yl)propan-1-one (3ff)

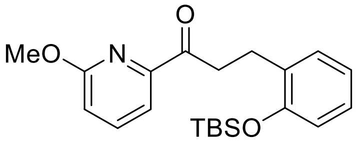

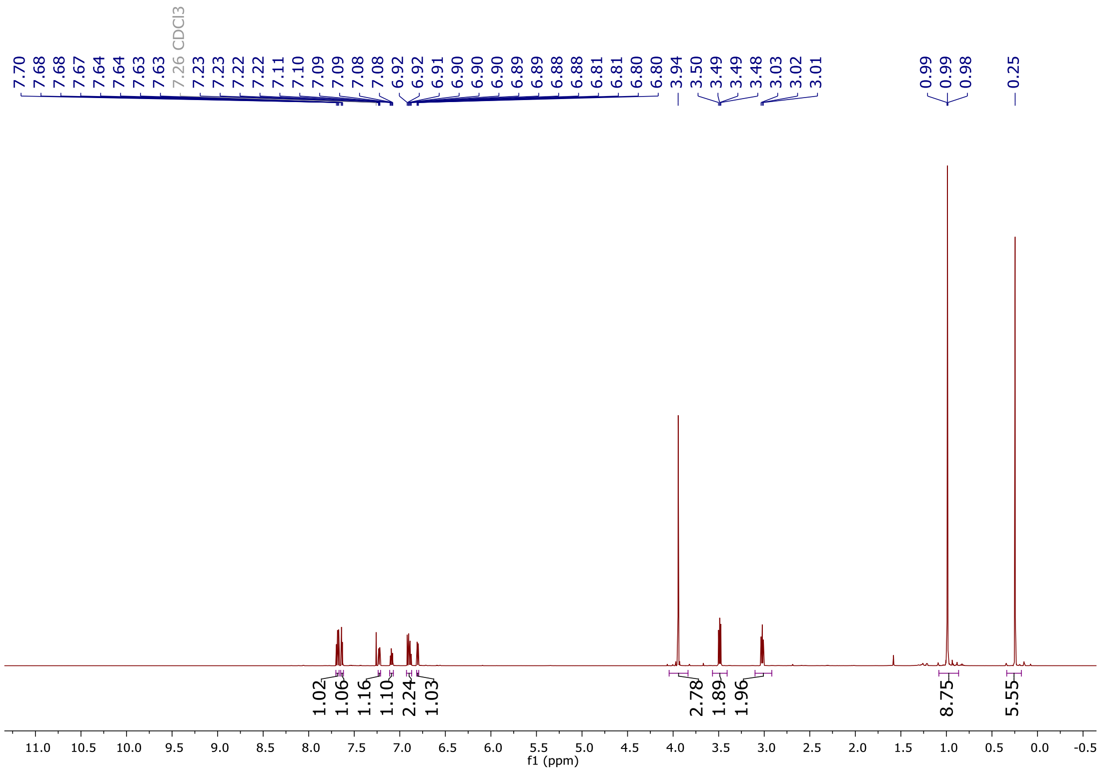

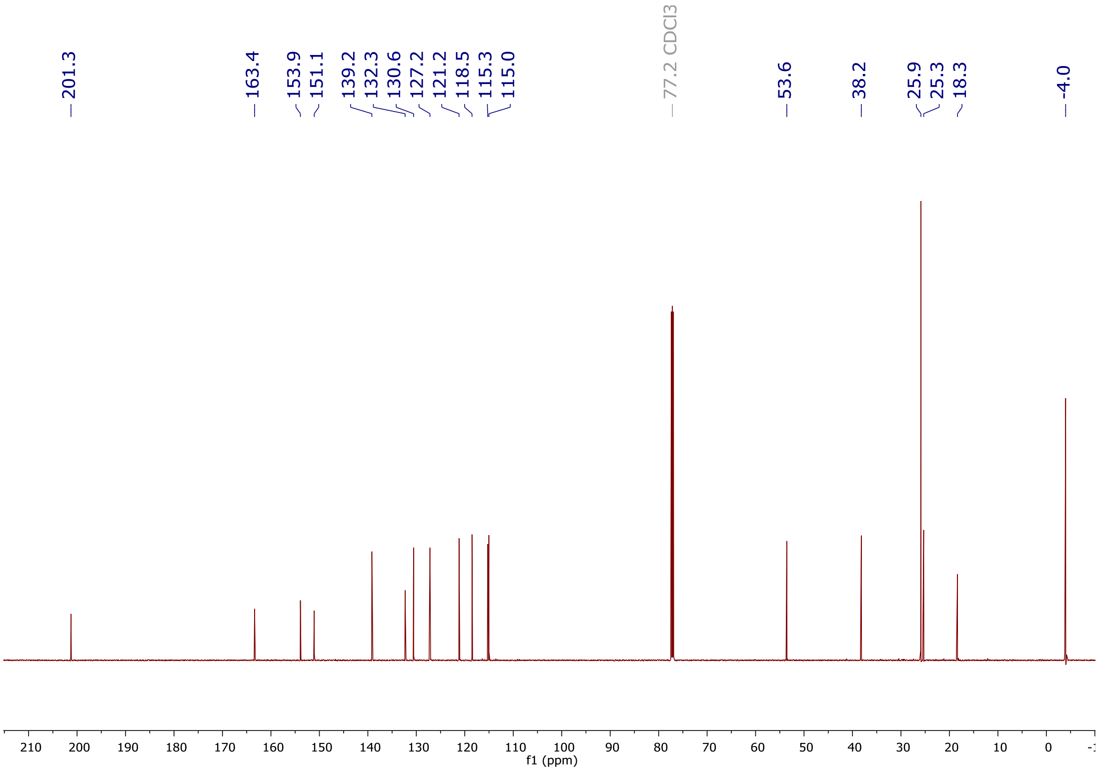

3-(2-((*Tert*-butyldimethylsilyl)oxy)phenyl)-1-(3-(diethoxymethyl)phenyl)propan-1-one (3fm)

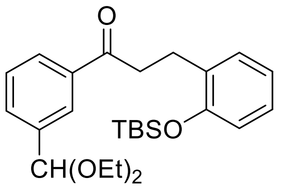

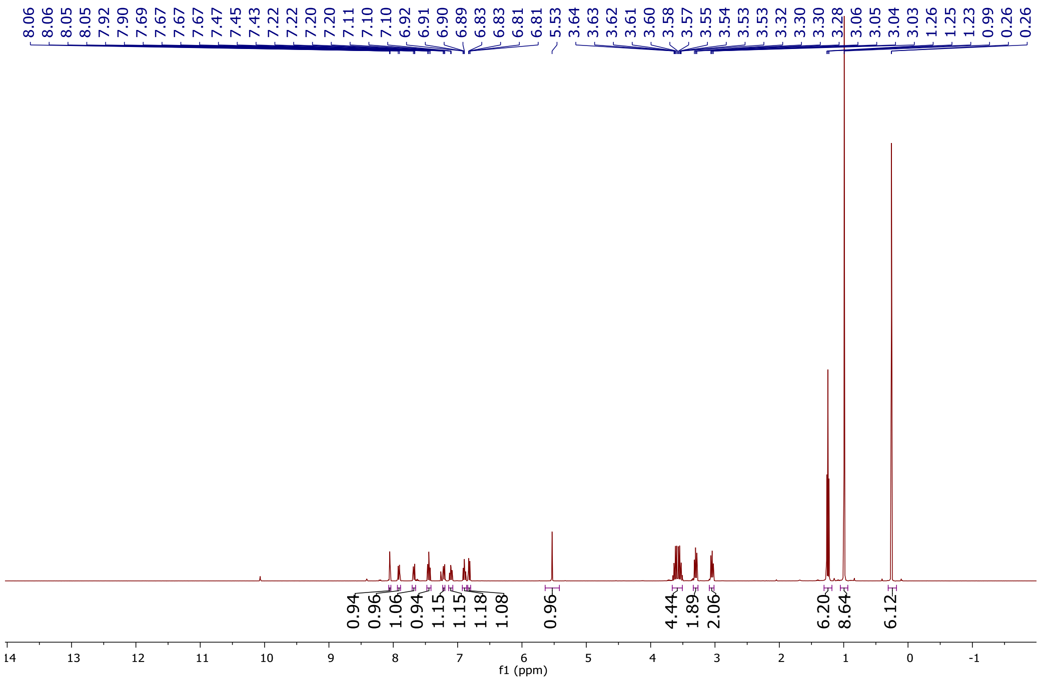

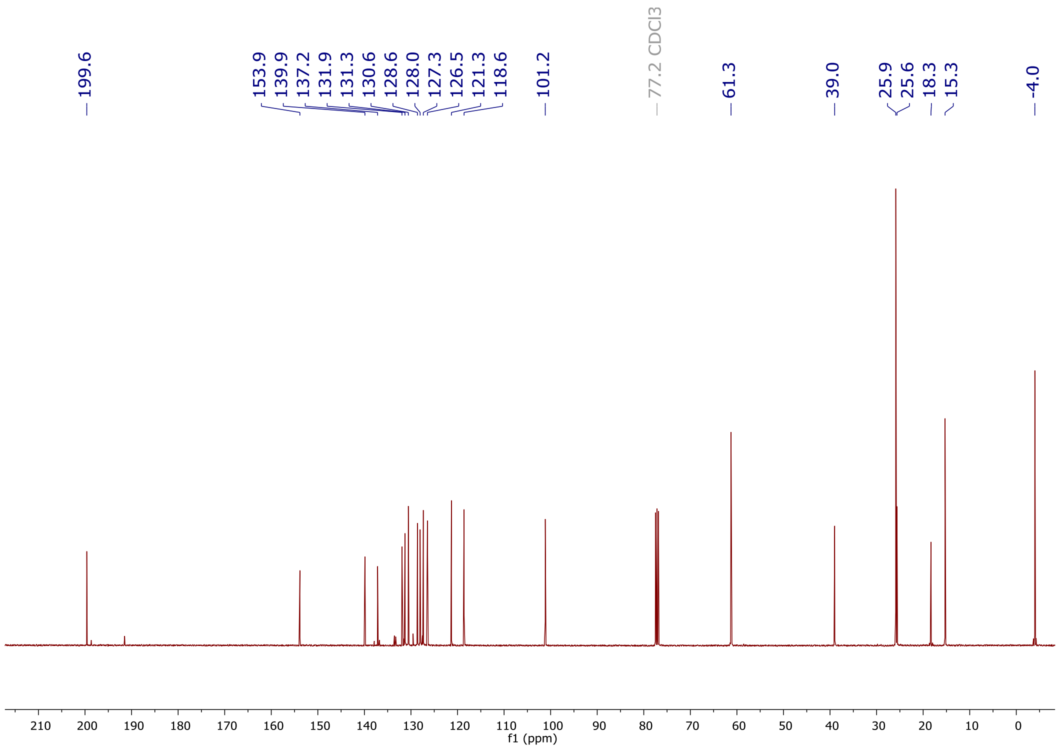

3-(2-((*Tert*-butyldimethylsilyl)oxy)phenyl)-1-(3-fluorophenyl)propan-1-one (3fn)

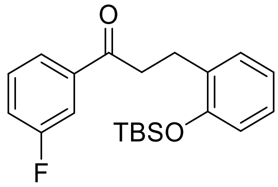

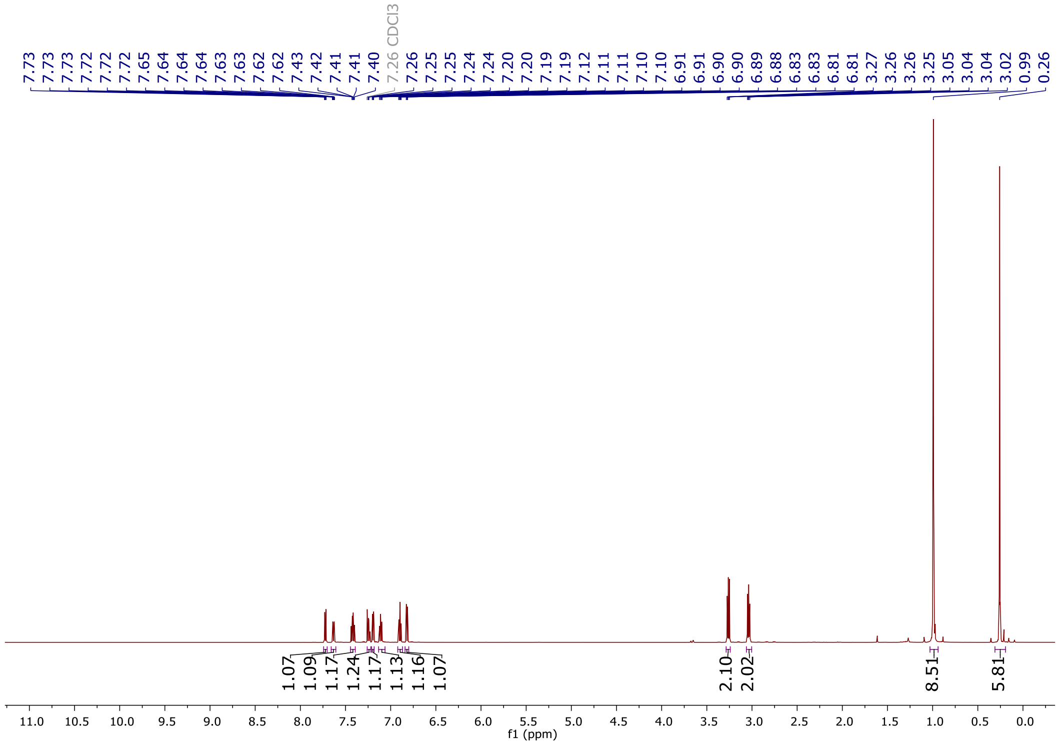

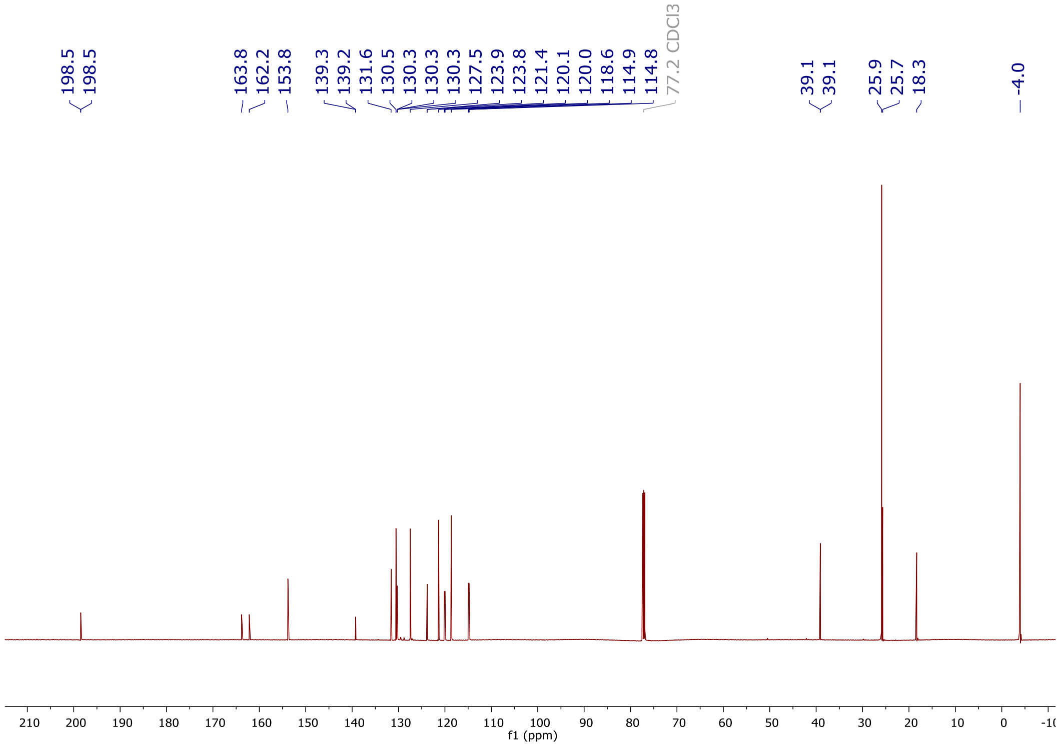

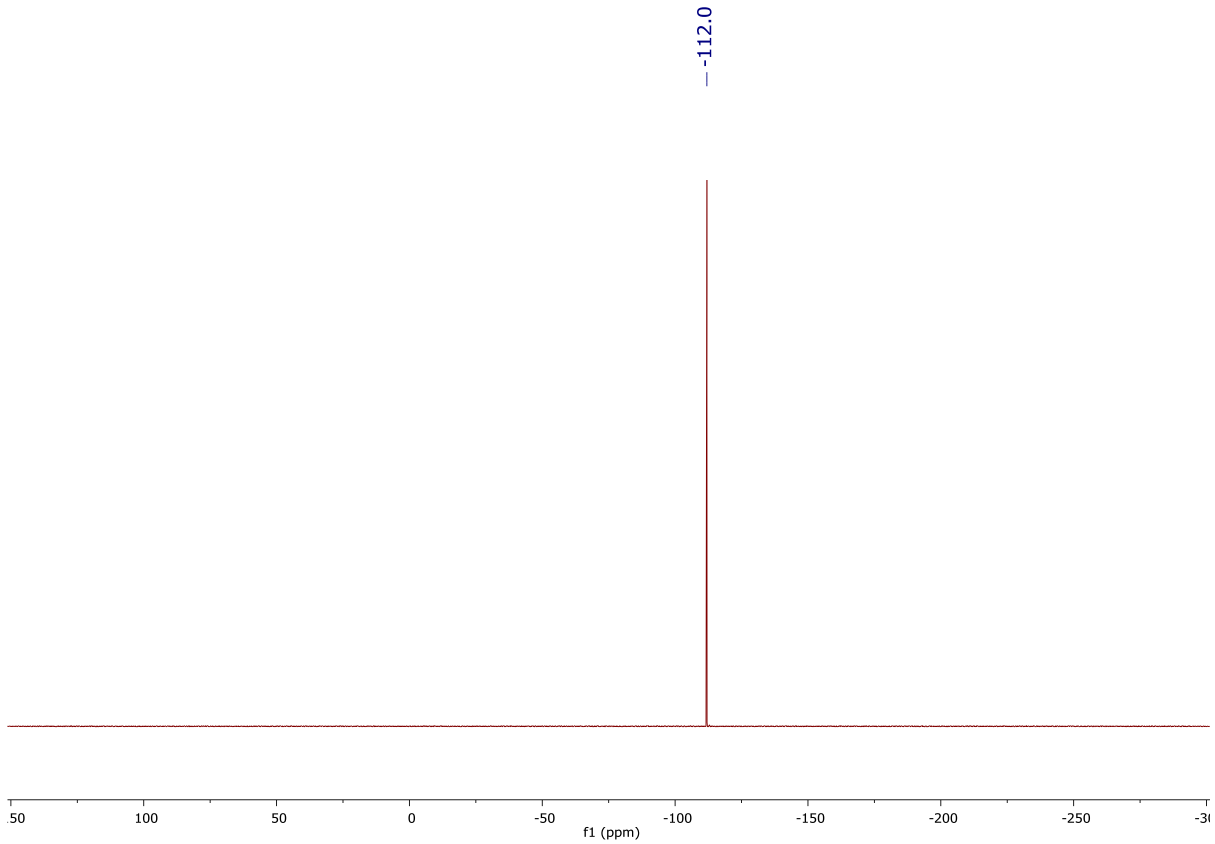
-112.0
150
100
50
0
-50
-100
-150
-200
-250
-300
f1 (ppm)

1-(4-Methoxyphenyl)-3-(piperidin-1-yl)propan-1-one (3gk)

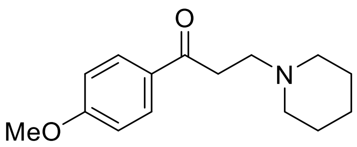

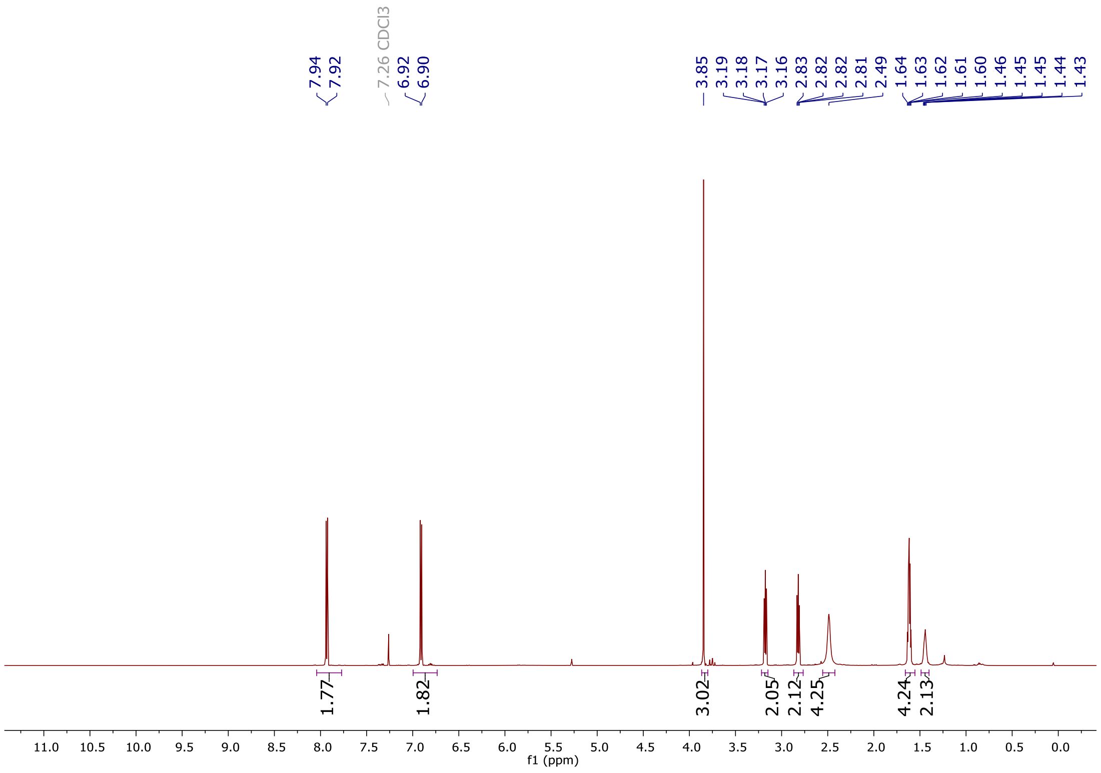

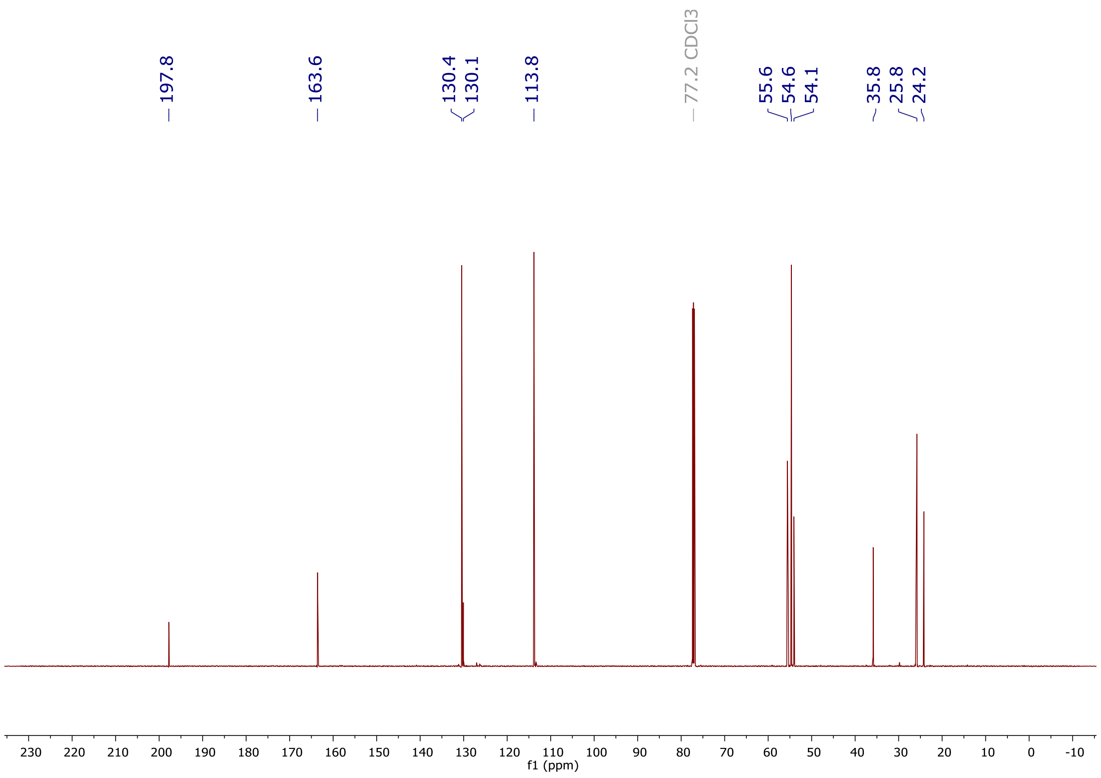

2-((1*r*,4*r*)-4-(4-Chlorophenyl)cyclohexyl)-1-(3,5-dimethoxyphenyl)ethan-1-one (3ho)

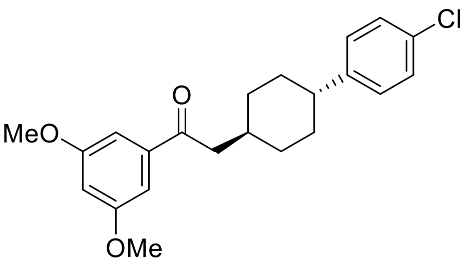

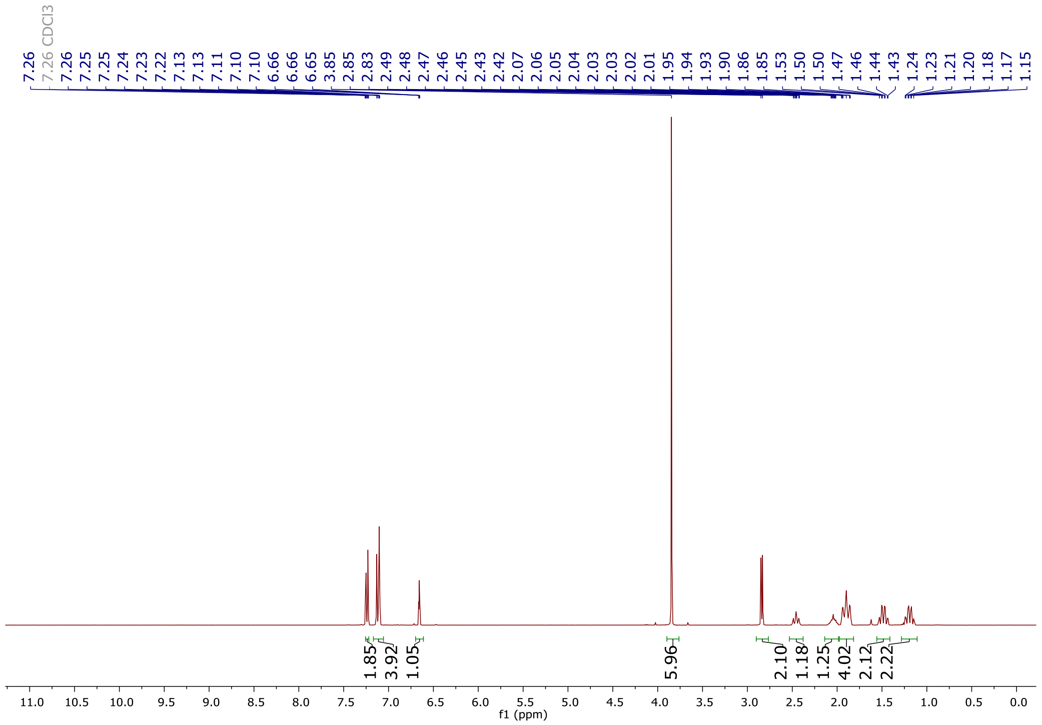

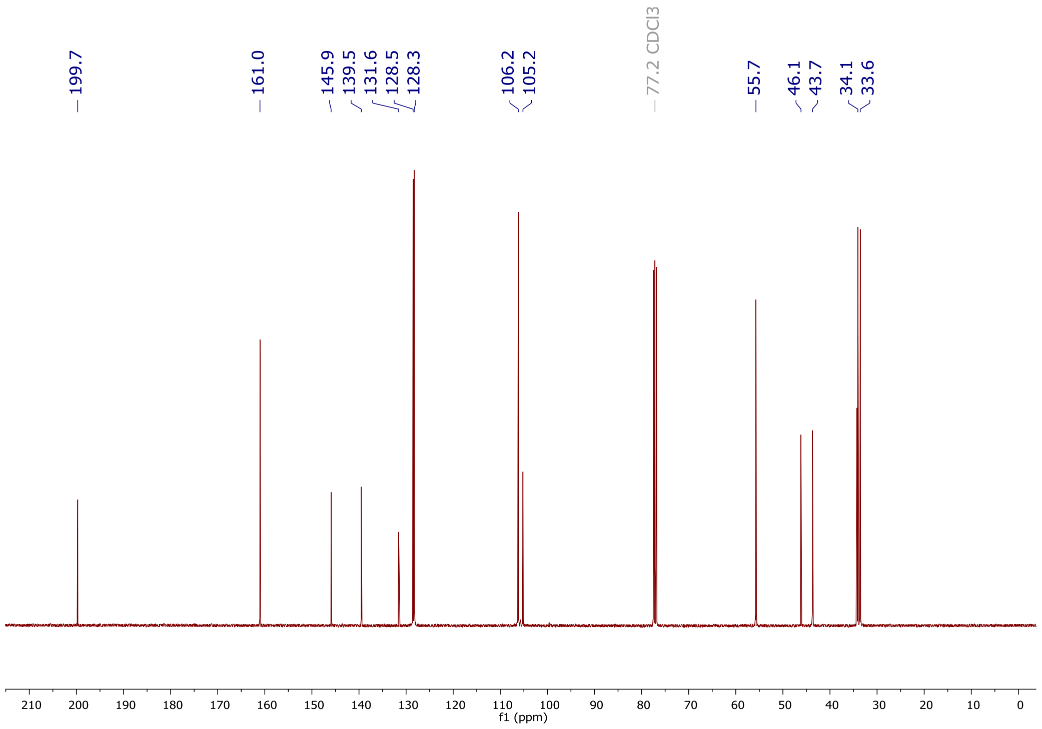

2-(4-Methoxyphenyl)-1-(4-(methylthio)phenyl)ethan-1-one (3ia)

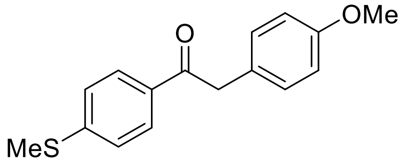

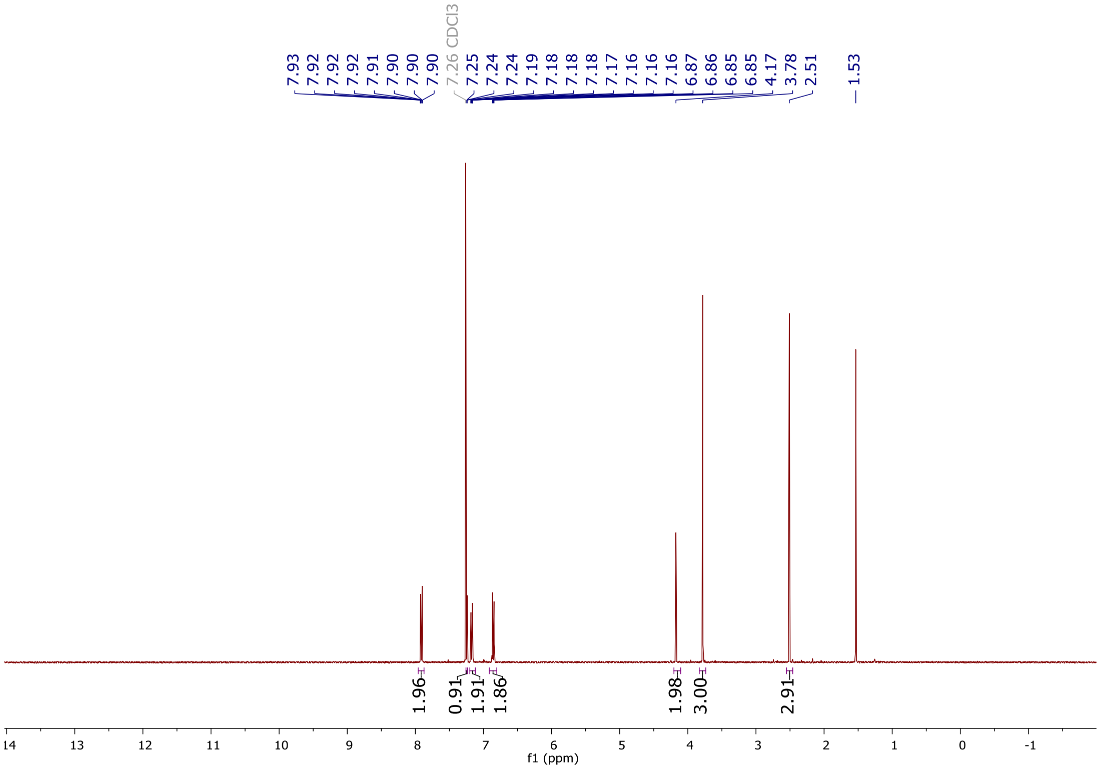

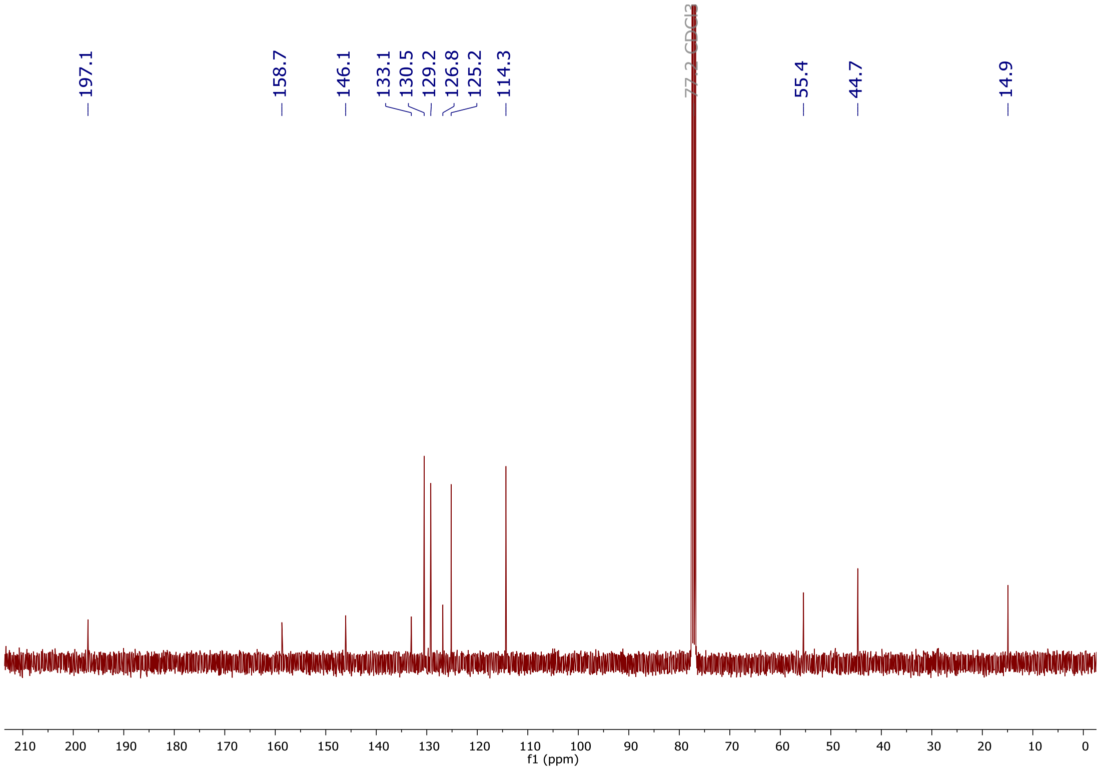

1,2-Bis(4-methoxyphenyl)ethan-1-one (3ik)

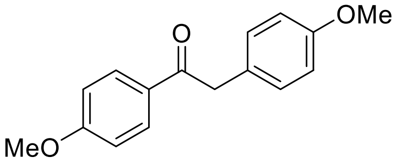

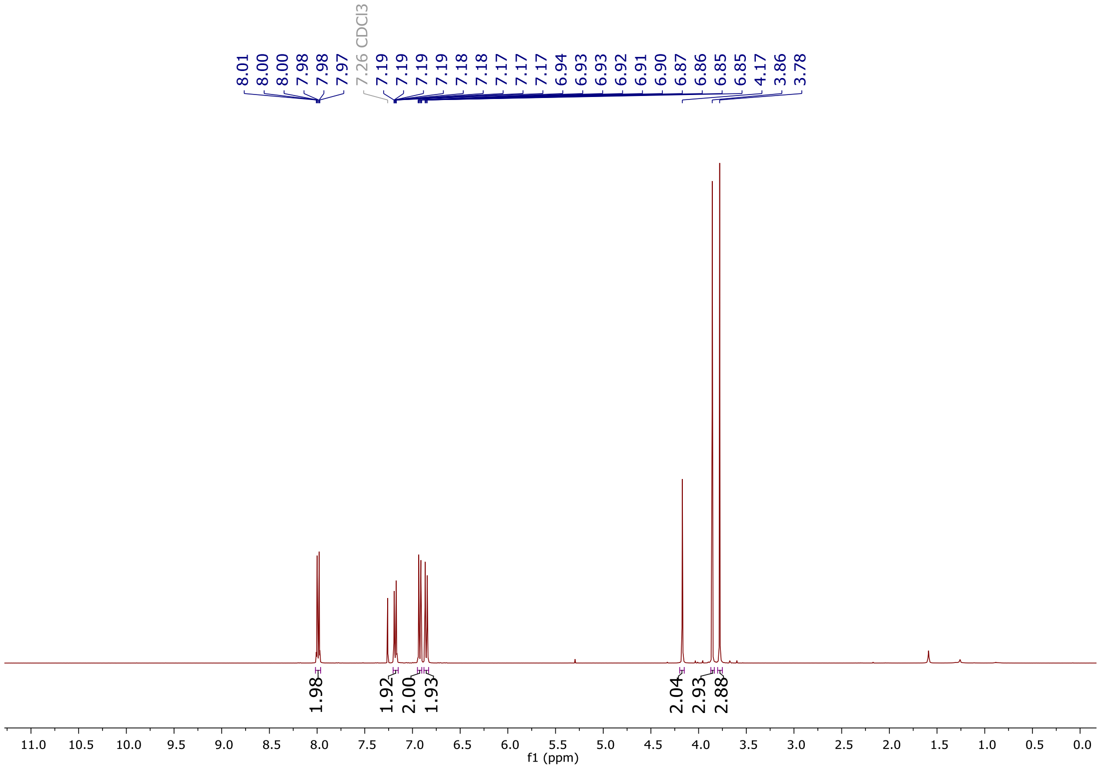

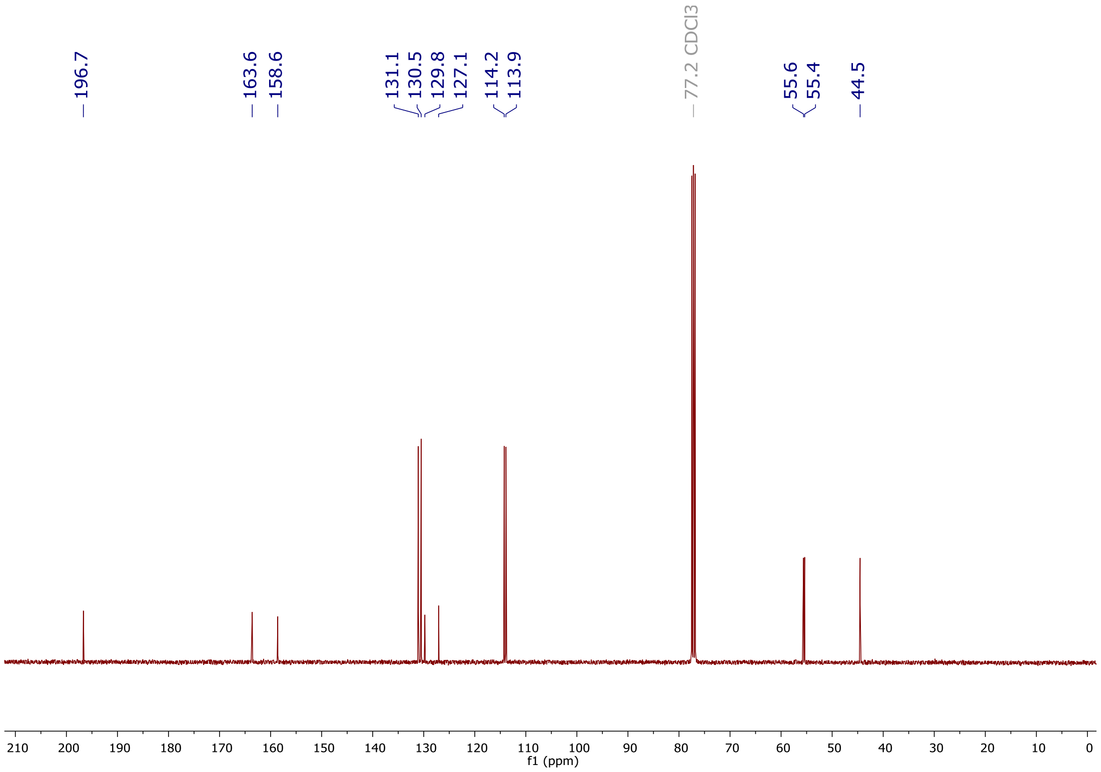

Bicyclo[1.1.1]pentan-1-yl(4-butoxyphenyl)methanone (3jp)

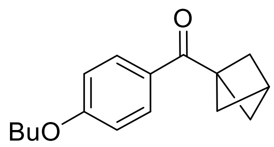

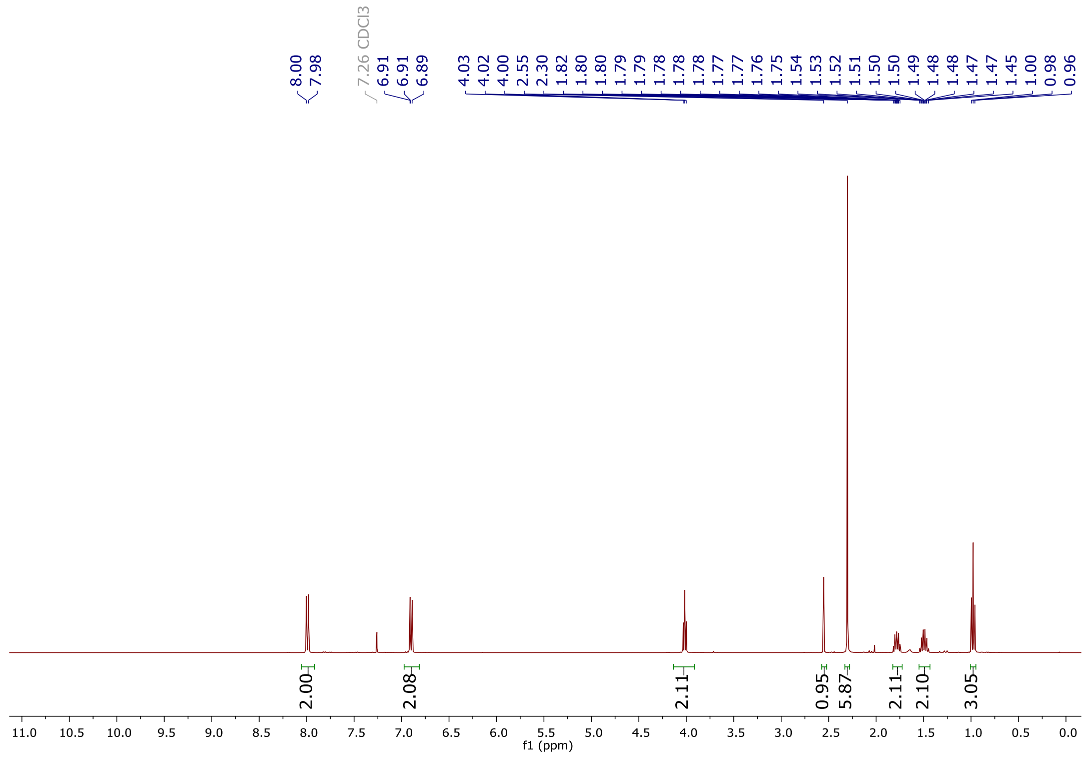

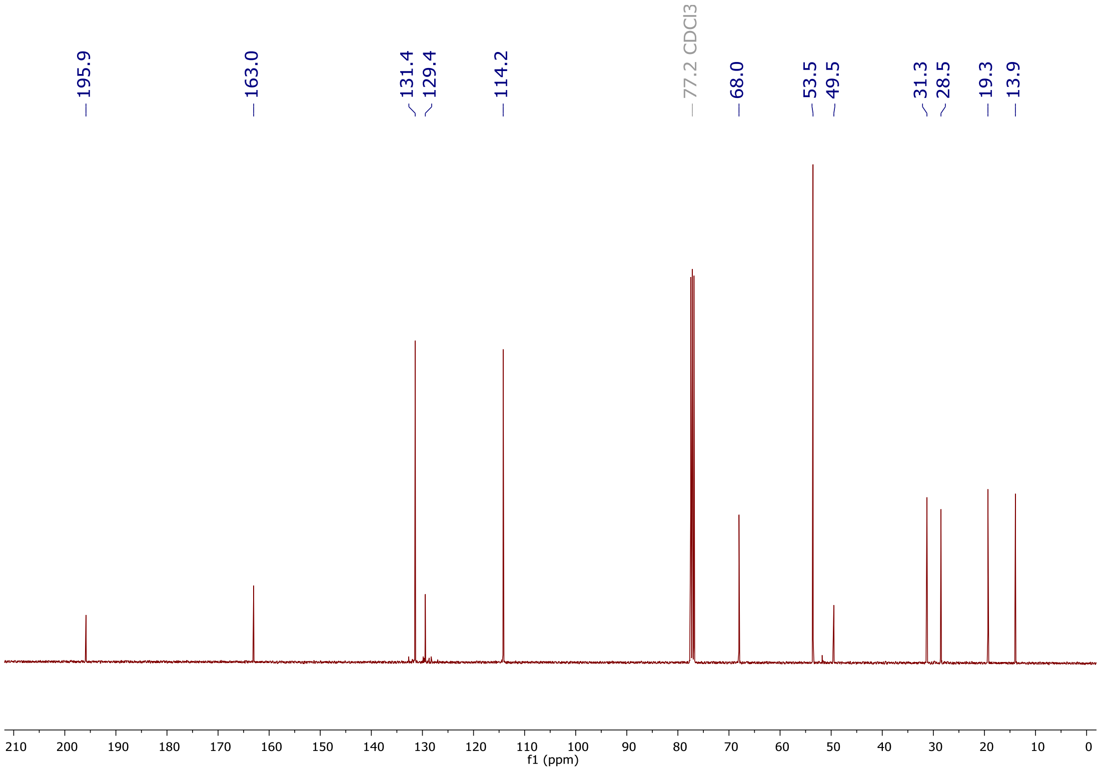

1-((1*r*,4*r*)-4-(4-Chlorophenyl)cyclohexyl)hexan-2-one (3hs)

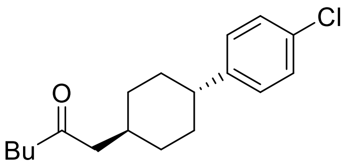

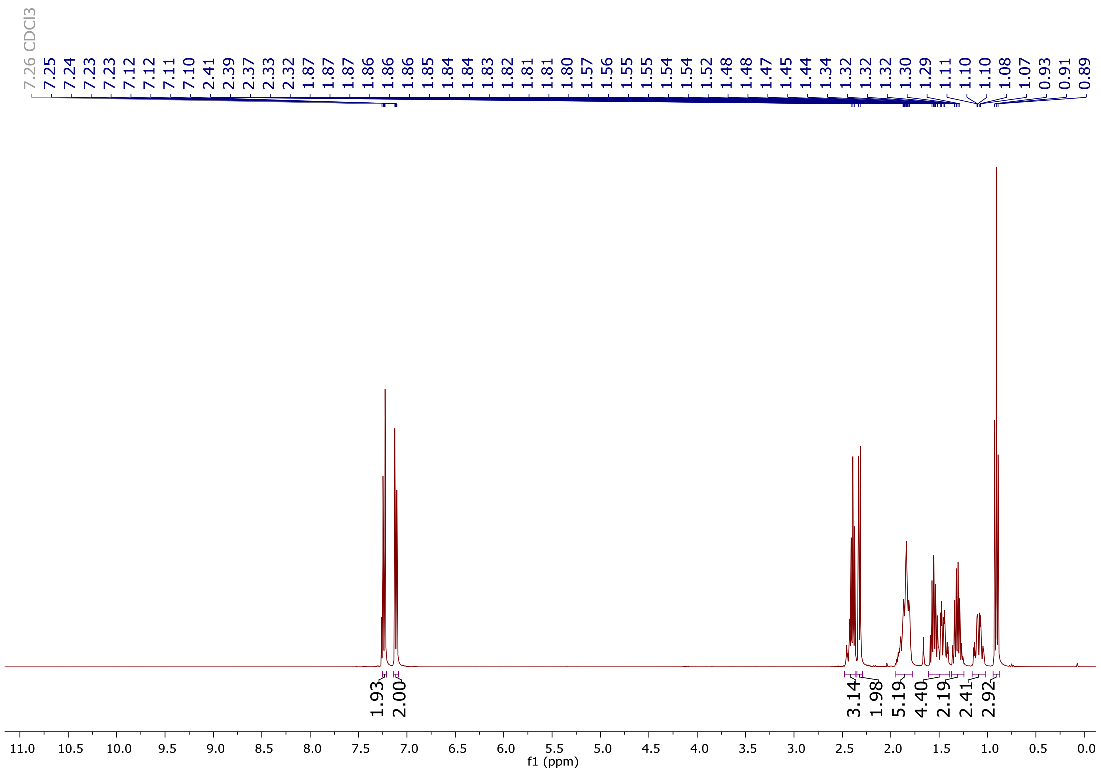

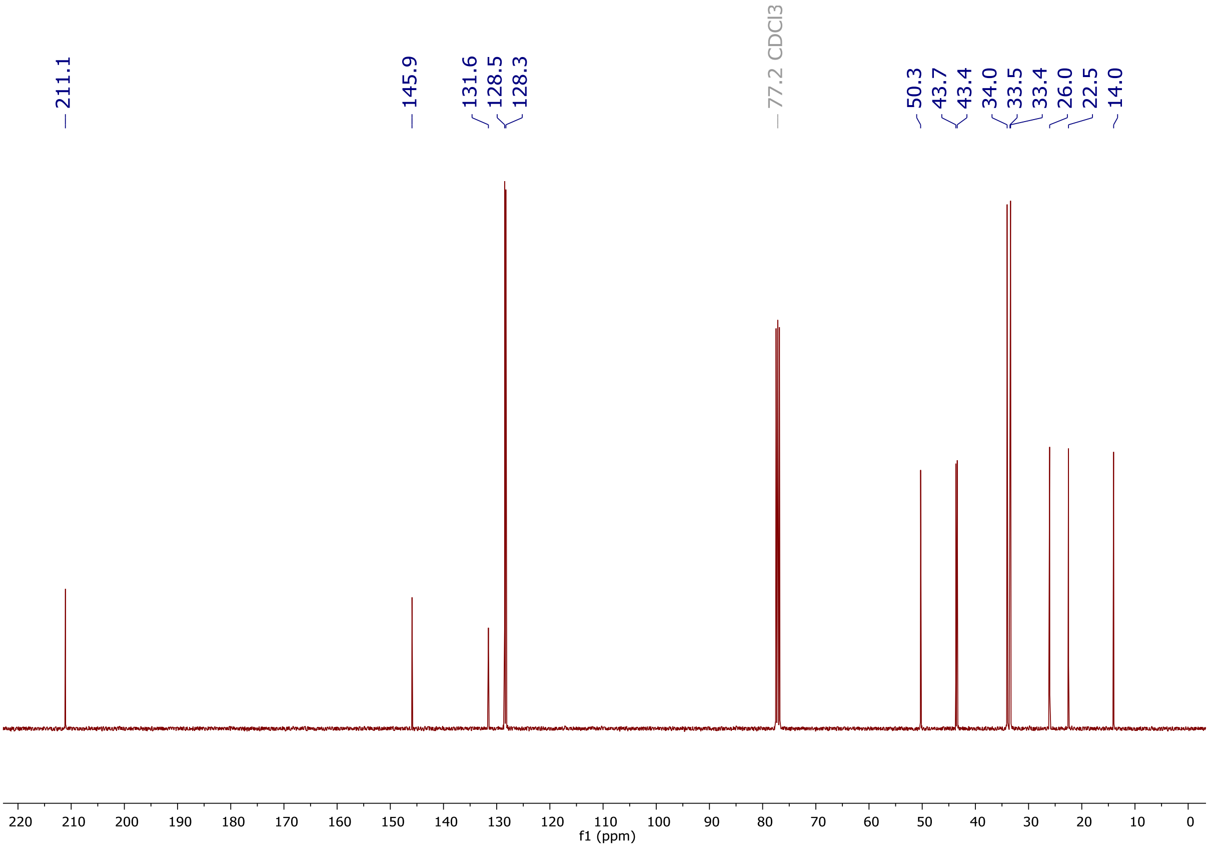

Bicyclo[1.1.1]pentan-1-yl(4-butoxyphenyl)methanone (3jr)

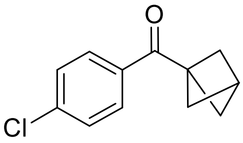

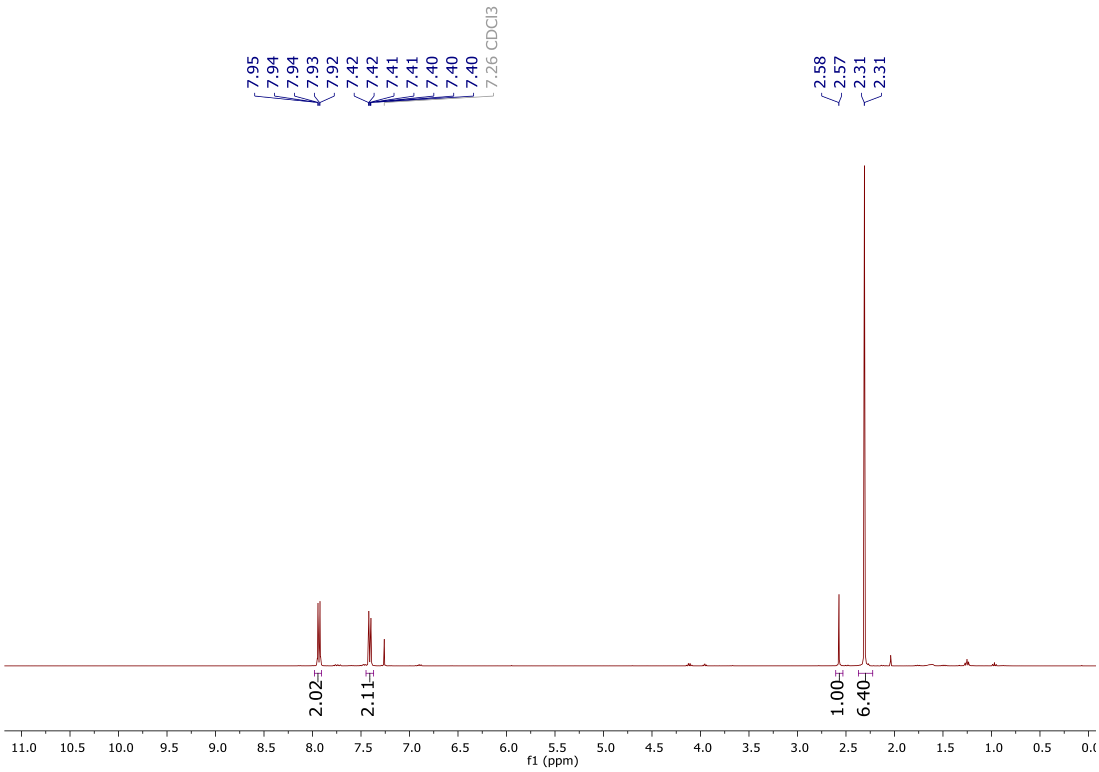

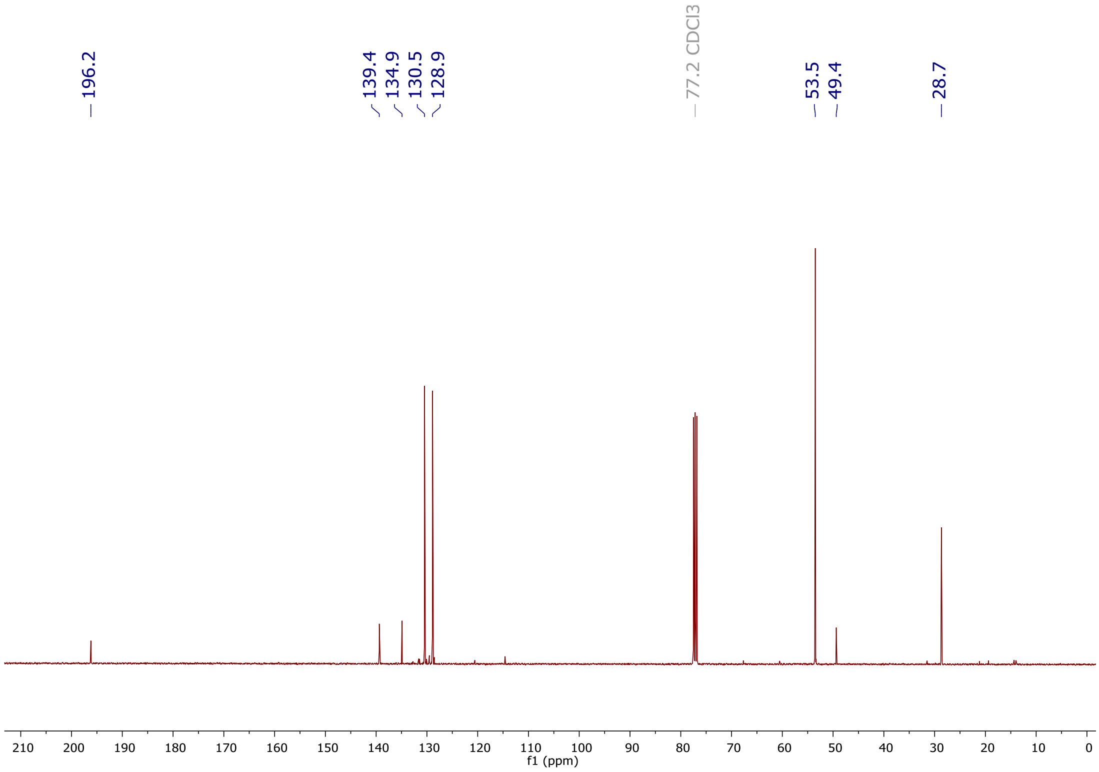

4-(Benzo[*b*]thiophene-5-carbonyl)benzonitrile (12ag)

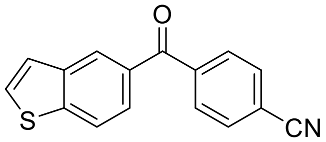

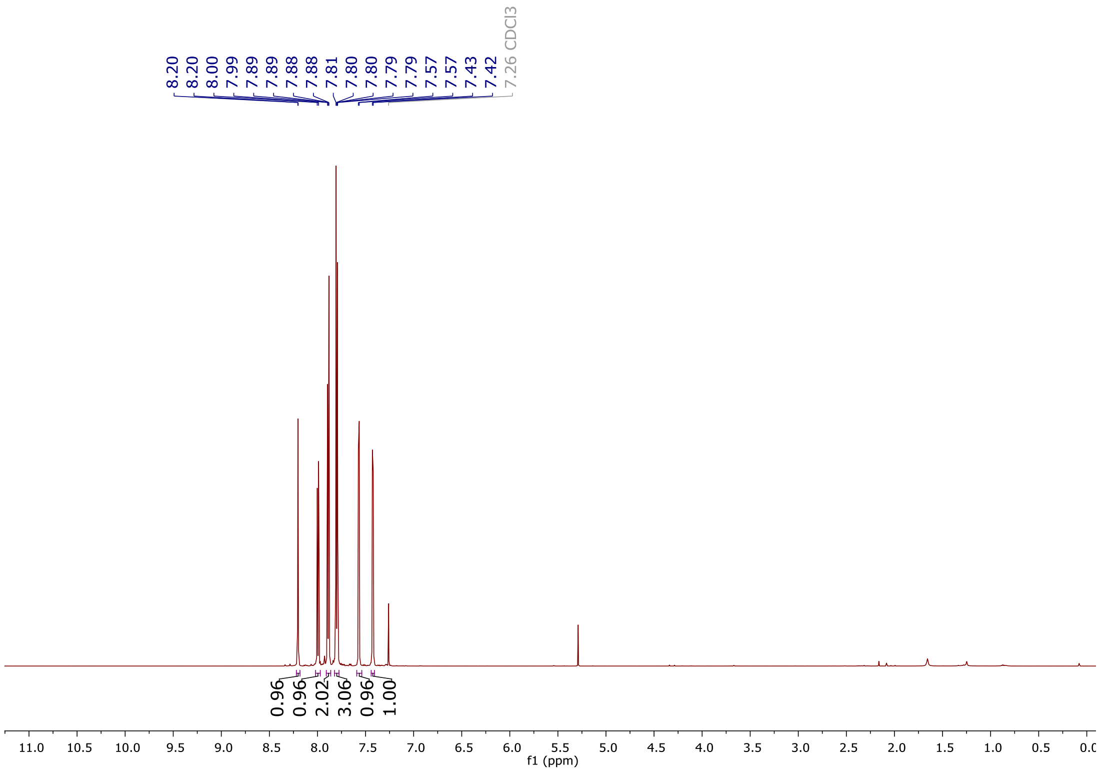

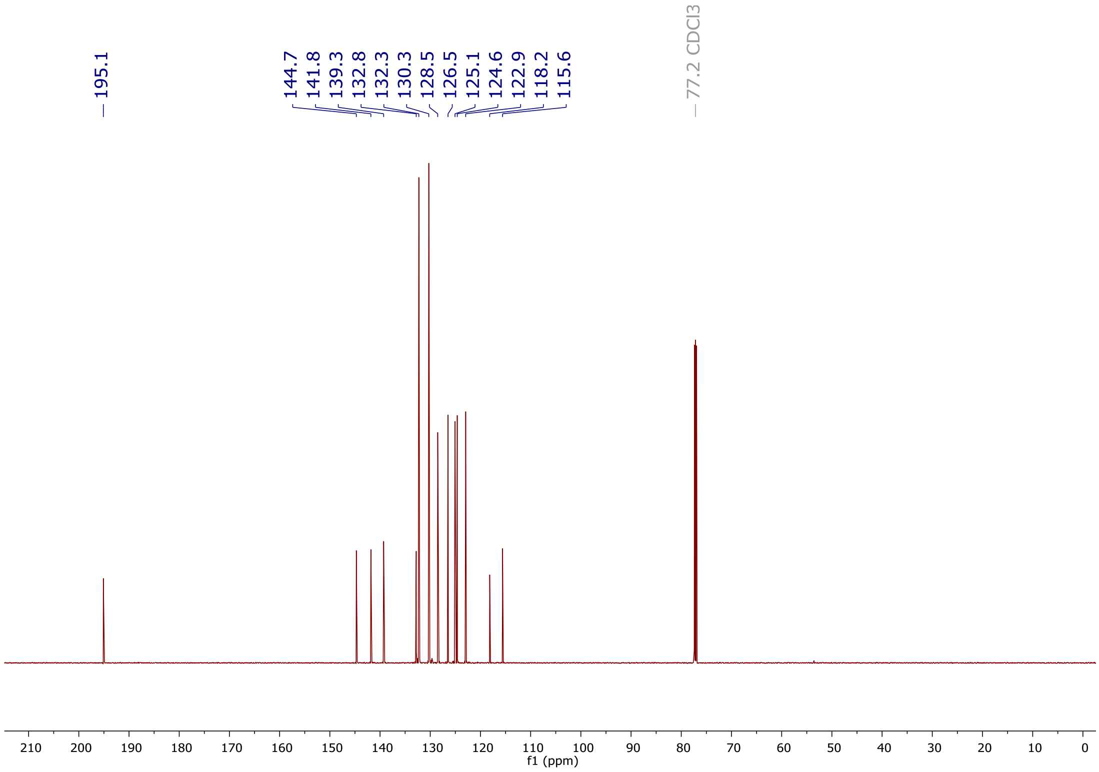

4-(6-Methoxypicolinoyl)benzonitrile (12af)

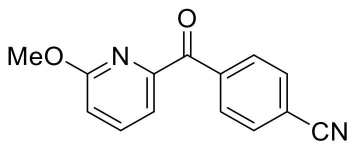

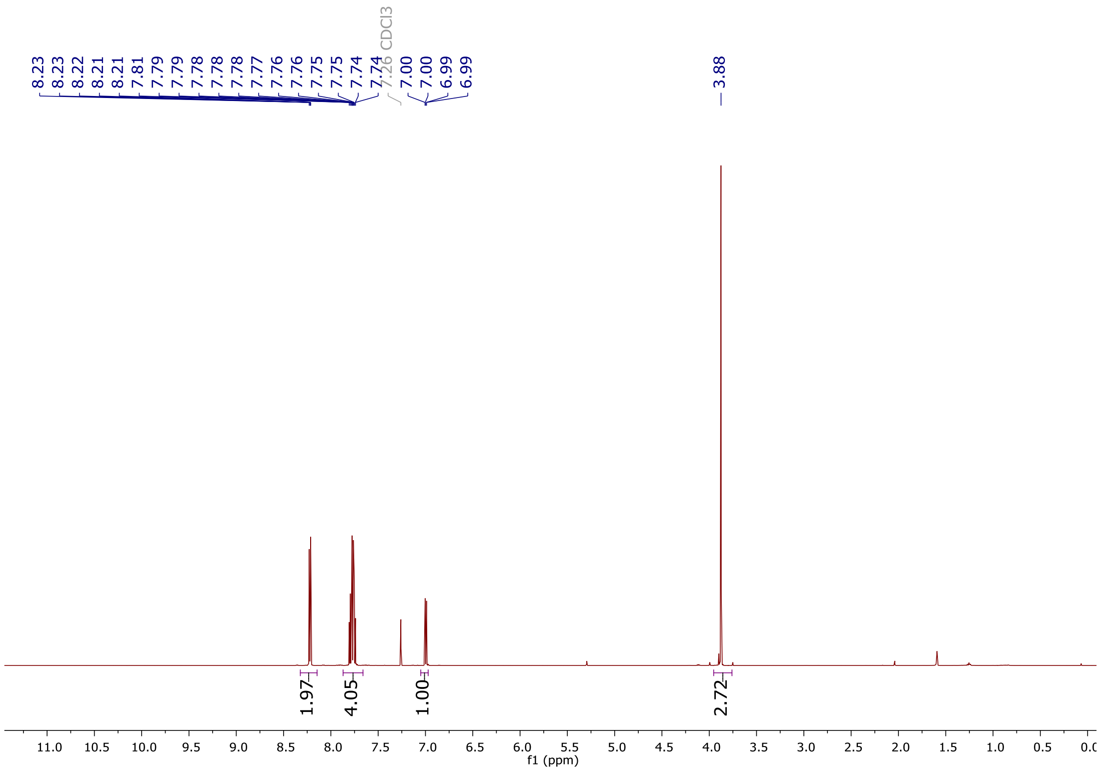

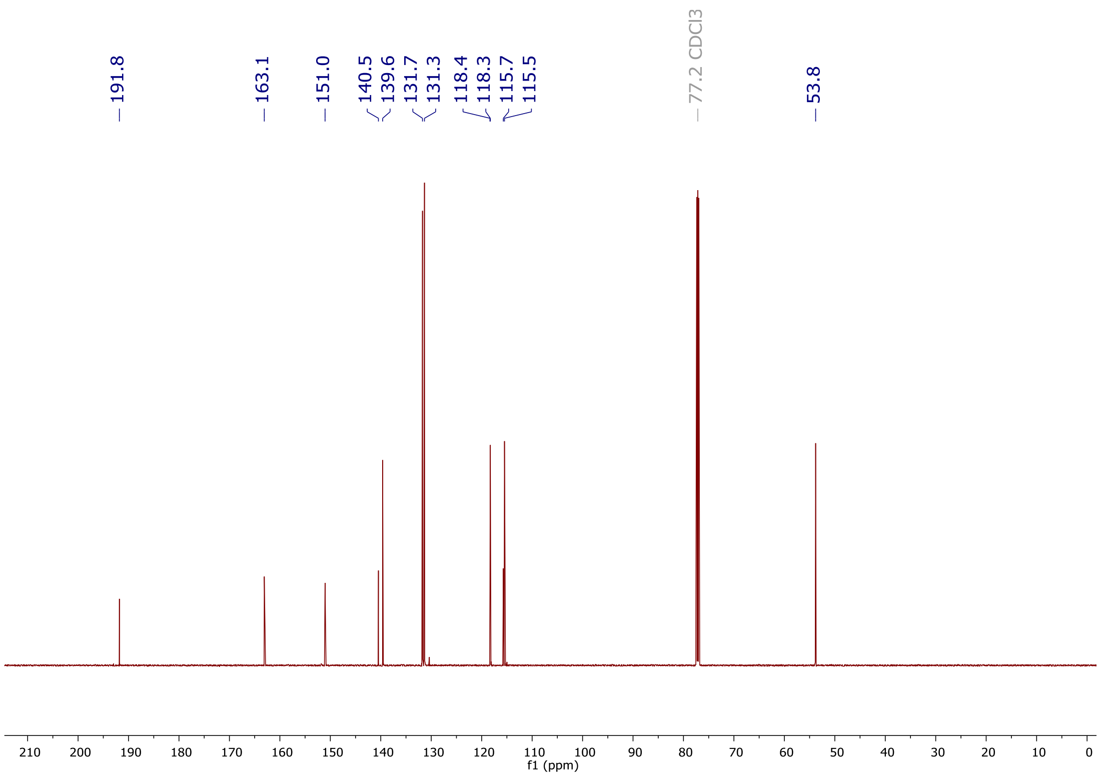

4-(5-Bromo-2-methoxybenzoyl)benzonitrile (12ae)

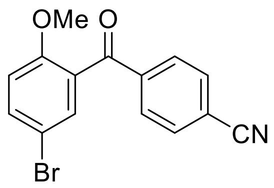

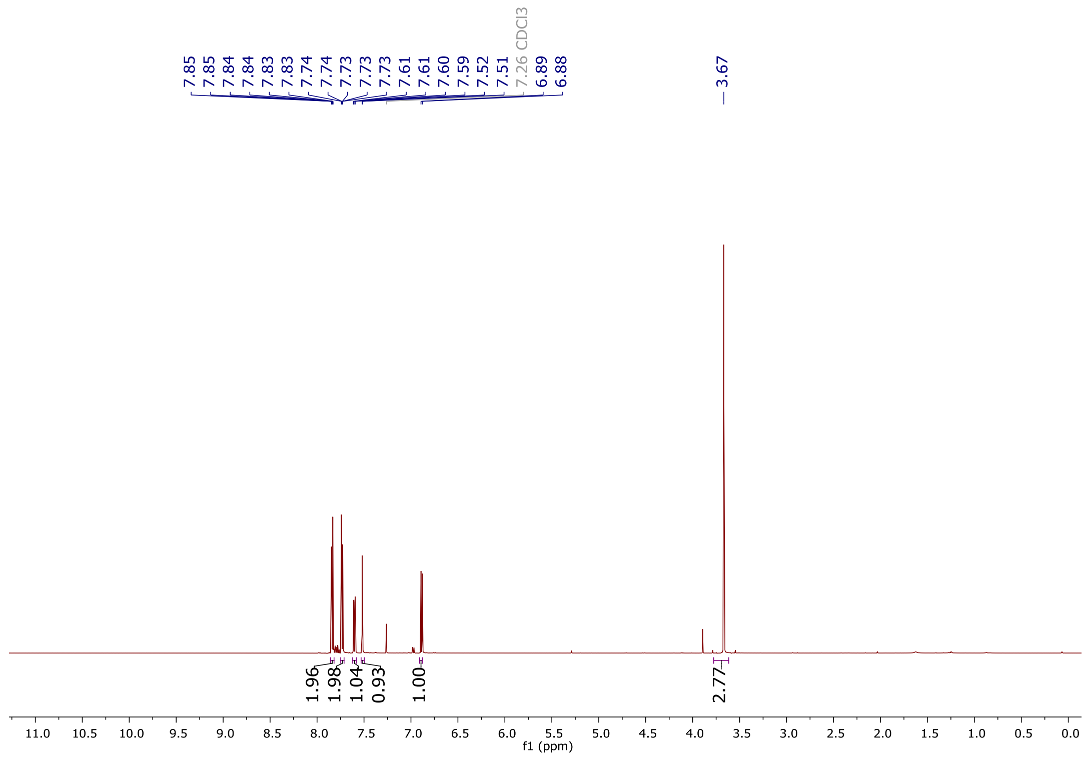

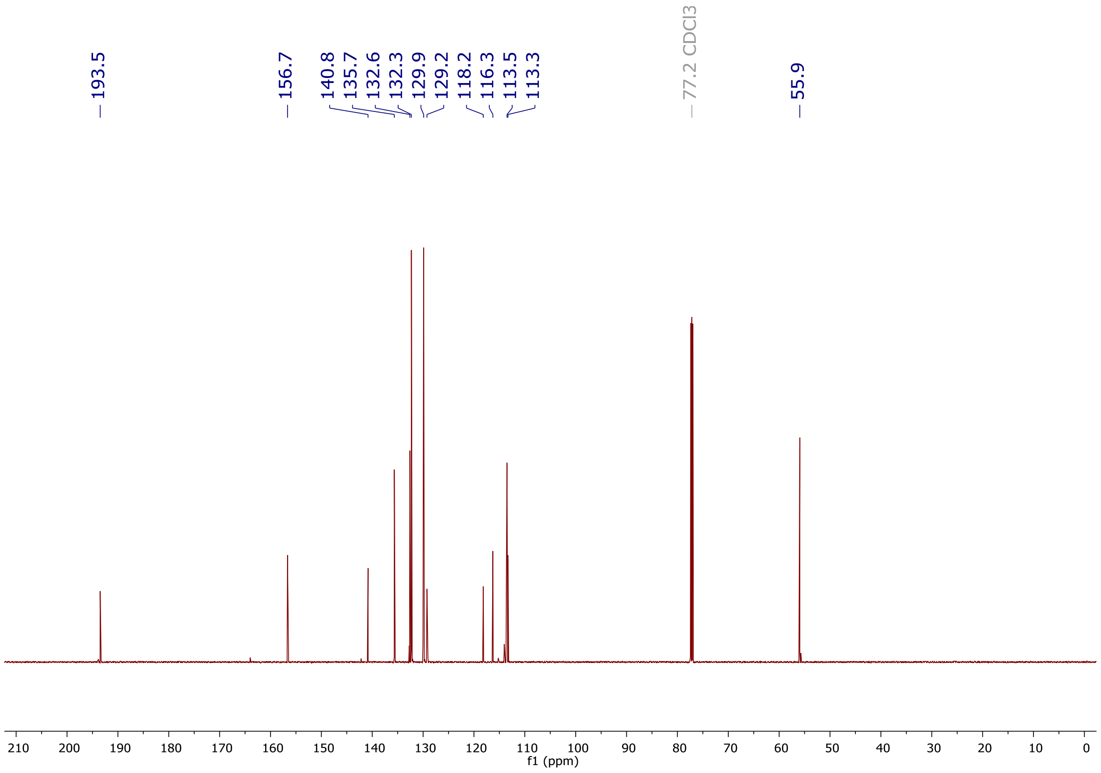

4-(3-Fluorobenzoyl)benzonitrile (12an)

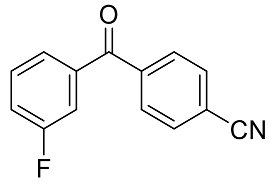

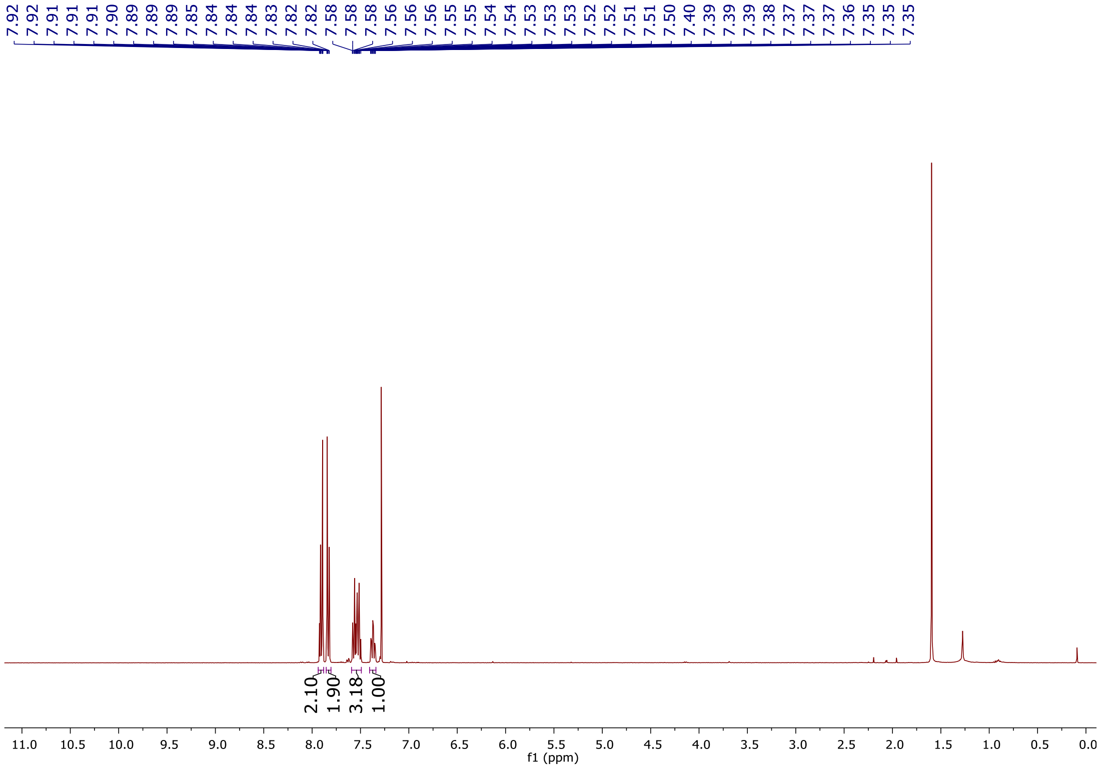

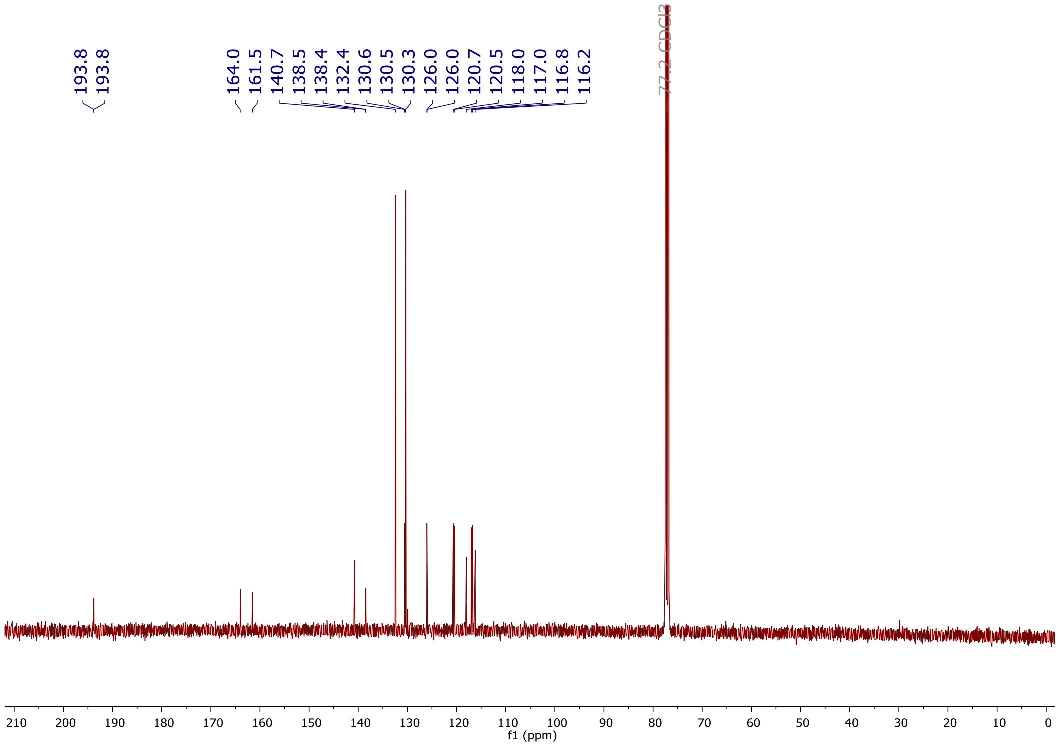

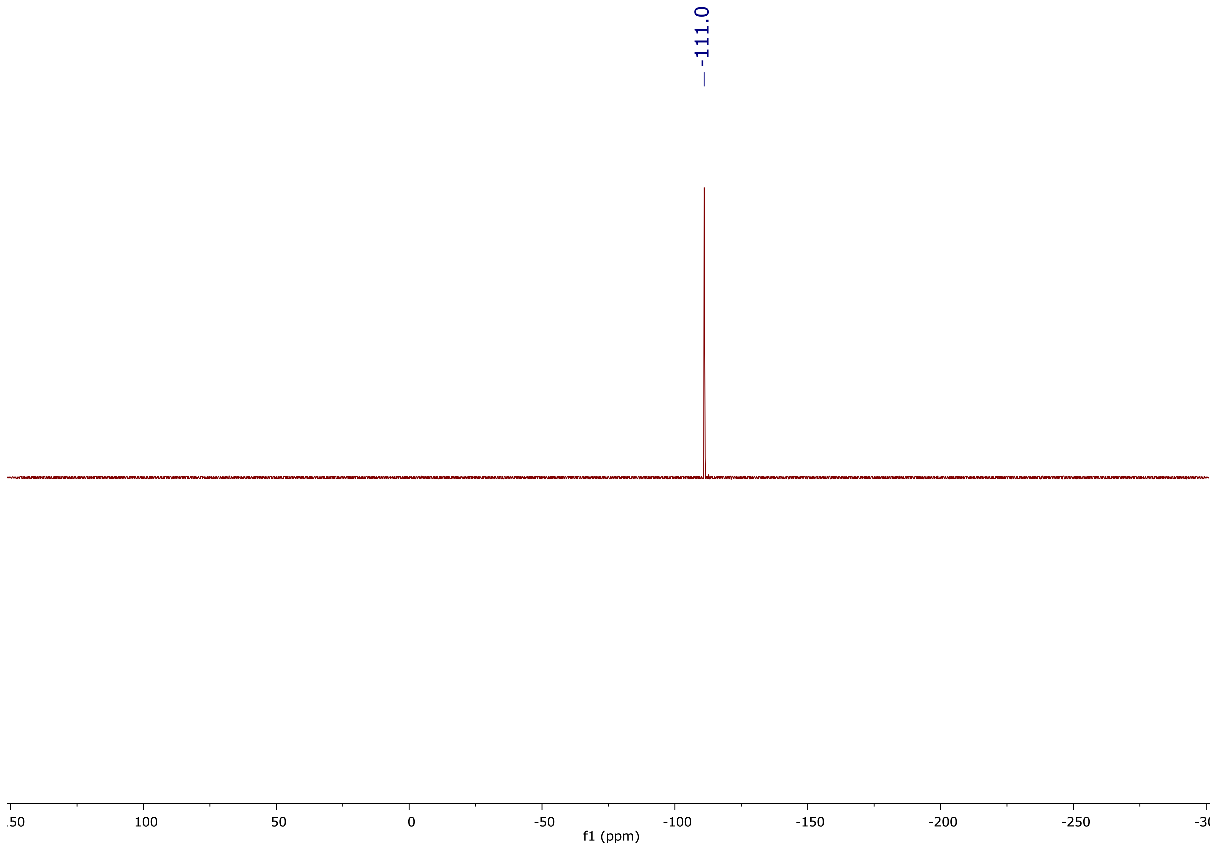

-111.0
150
100
50
0
-50
-100
-150
-200
-250
-30
f1 (ppm)

(3-Bromo-5-fluorophenyl)(4-iodophenyl)methanone (12bq)

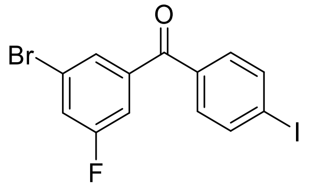

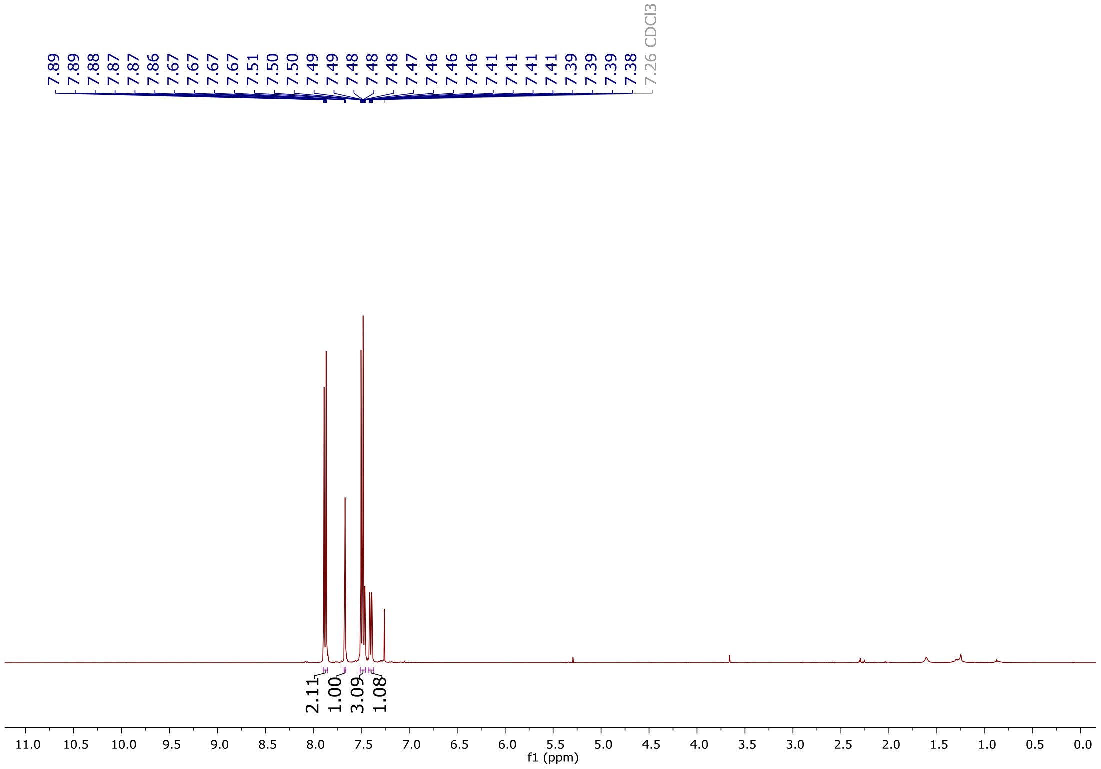

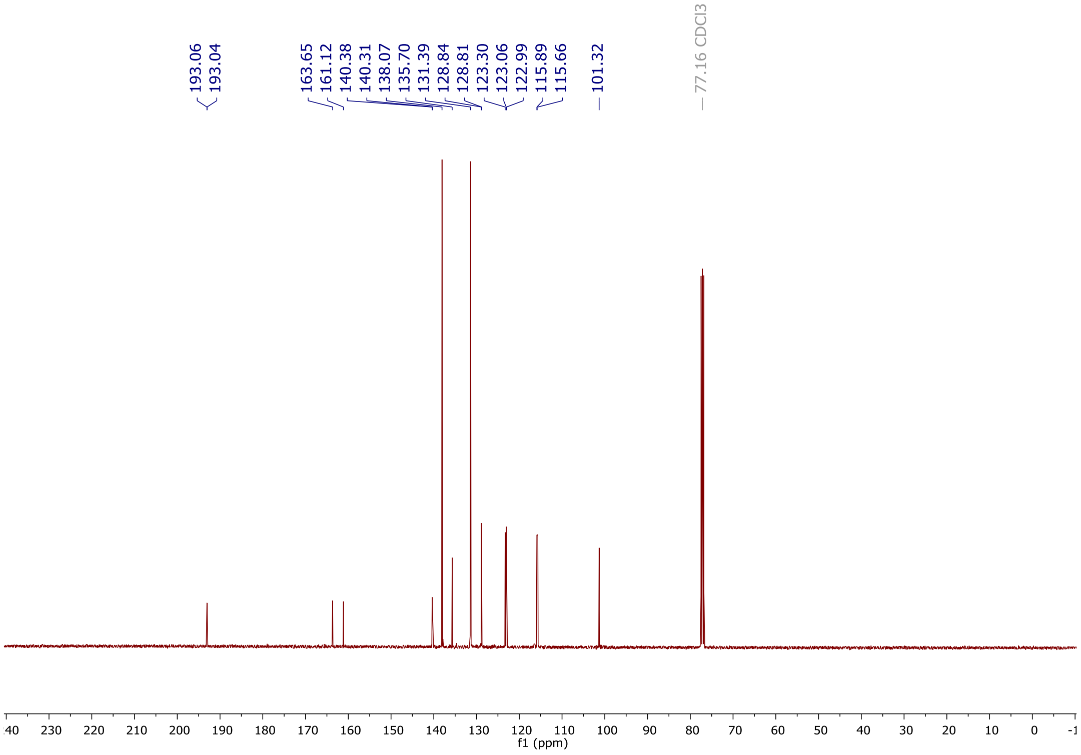

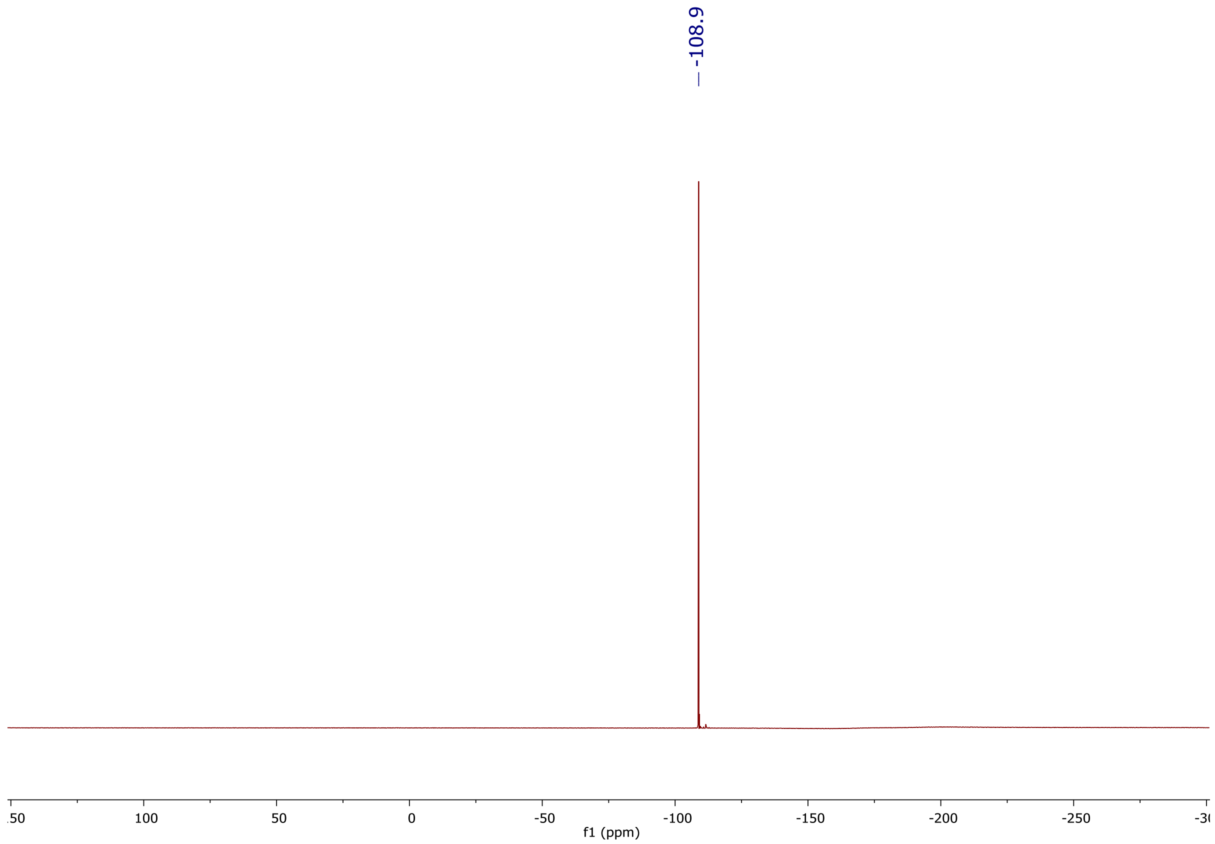
-108.9
50
100
50
0
-50
-100
-150
-200
-250
f1 (ppm)

(4-Chlorophenyl)(4-iodophenyl)methanone (12br)

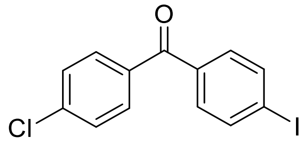

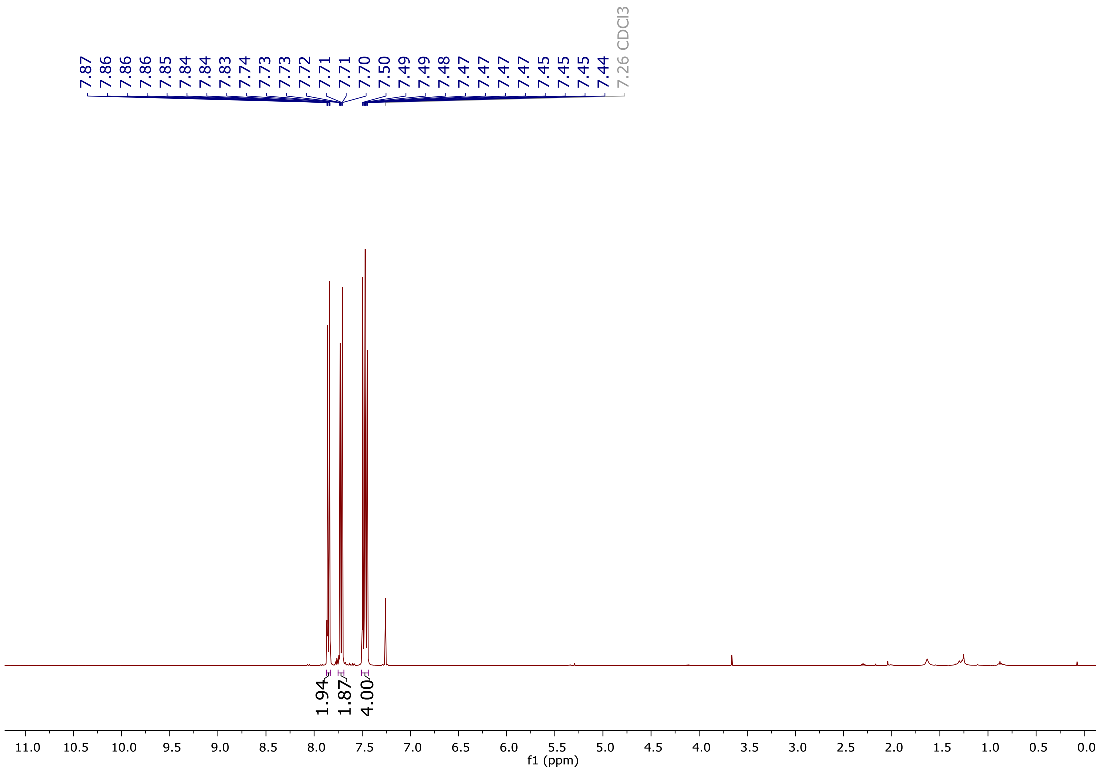

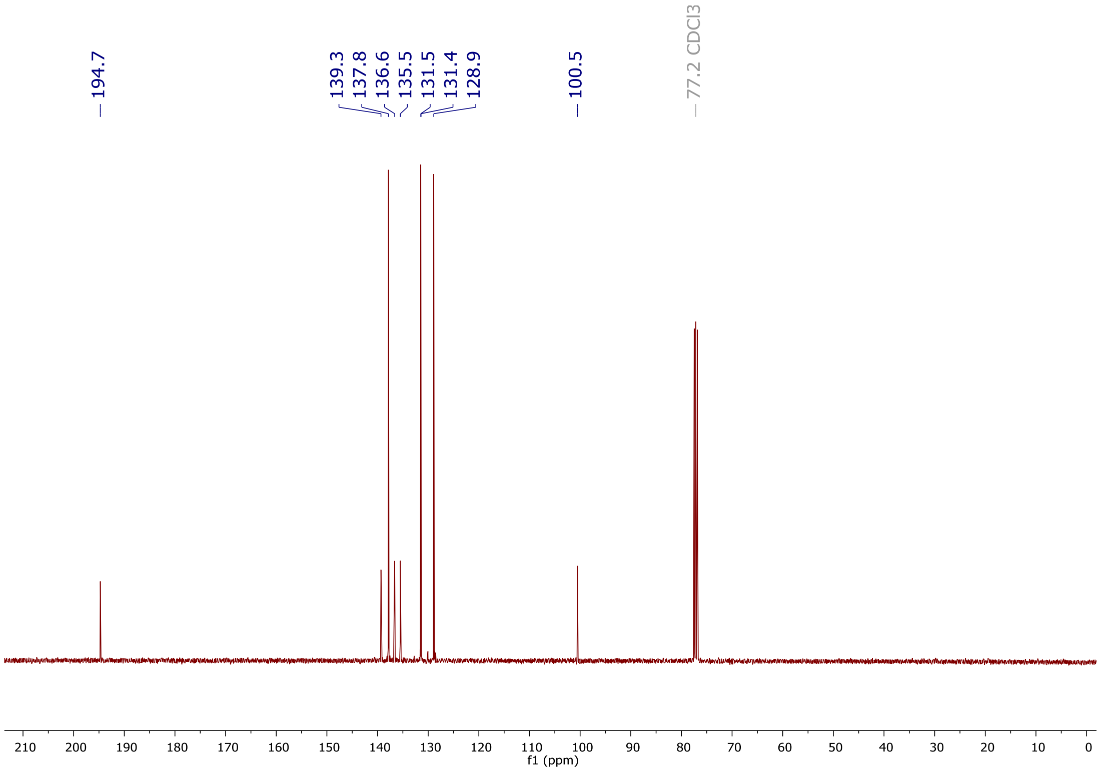

(4-Chlorophenyl)(4-iodophenyl)methanone (12cr)

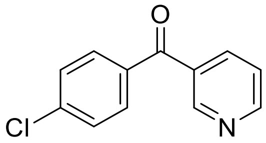

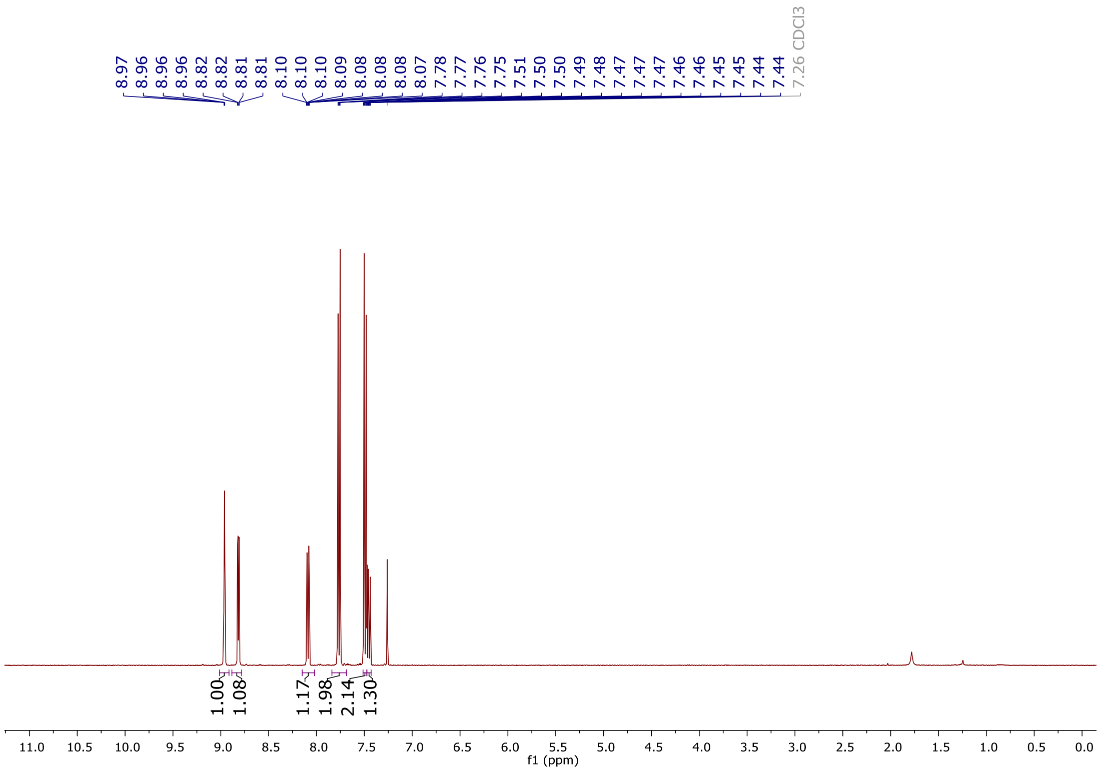

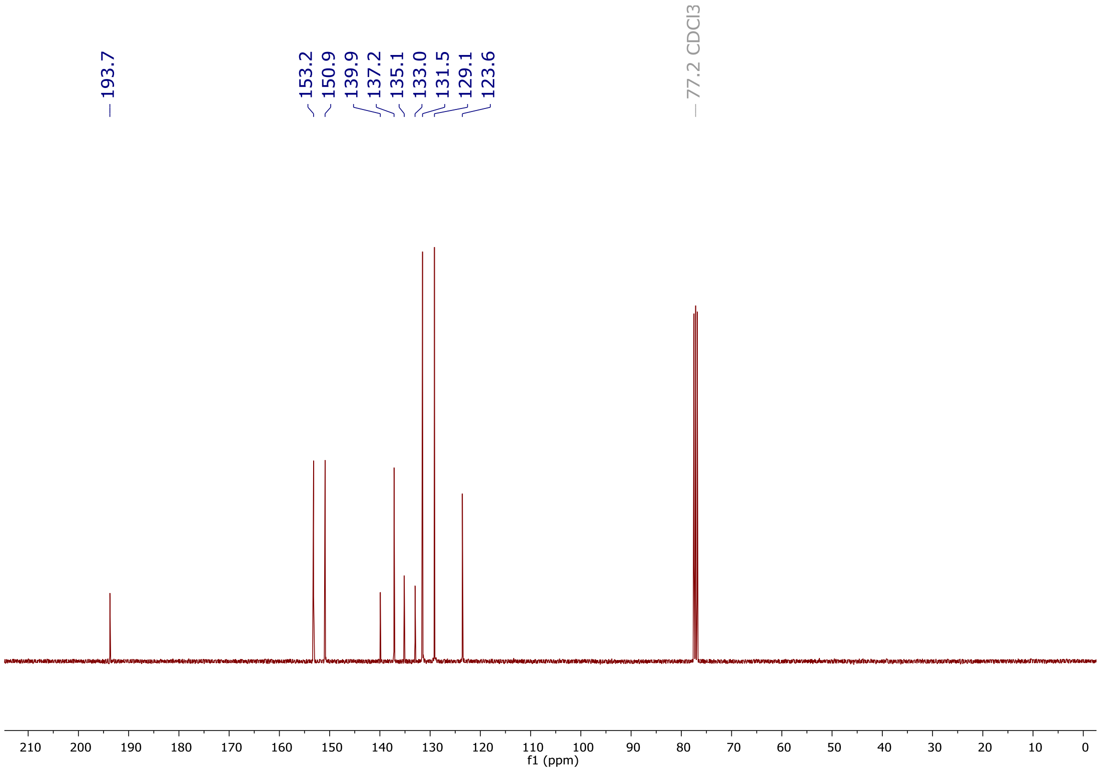

(*S*)-1-(3-Bromophenyl)-2-(6-methoxynaphthalen-2-yl)propan-1-one (14ac)

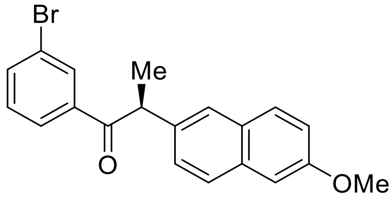

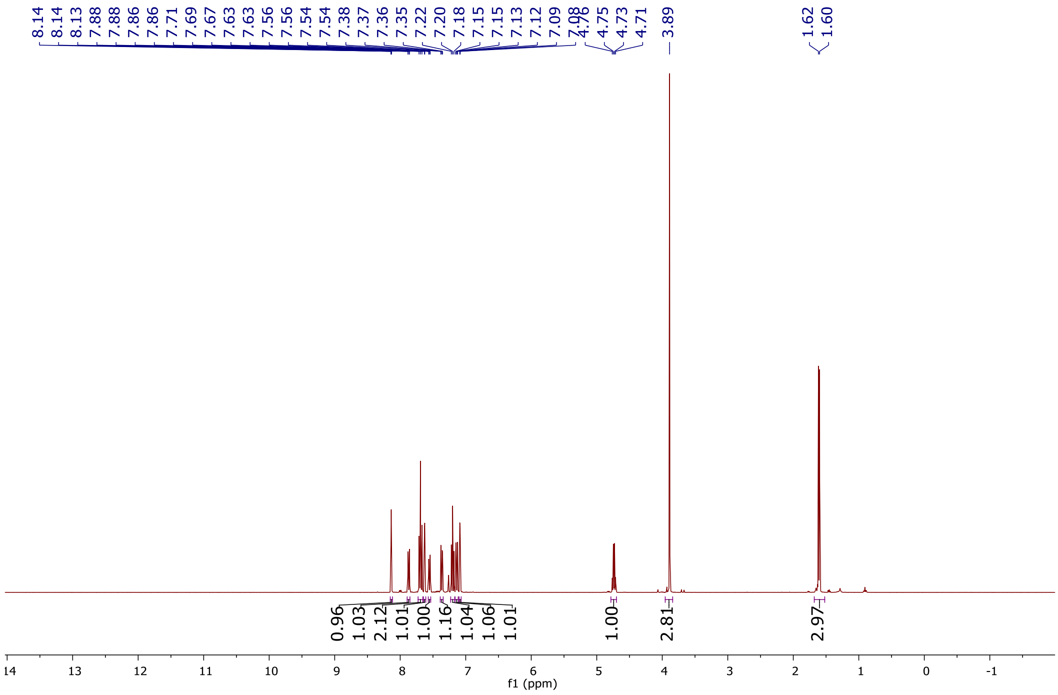

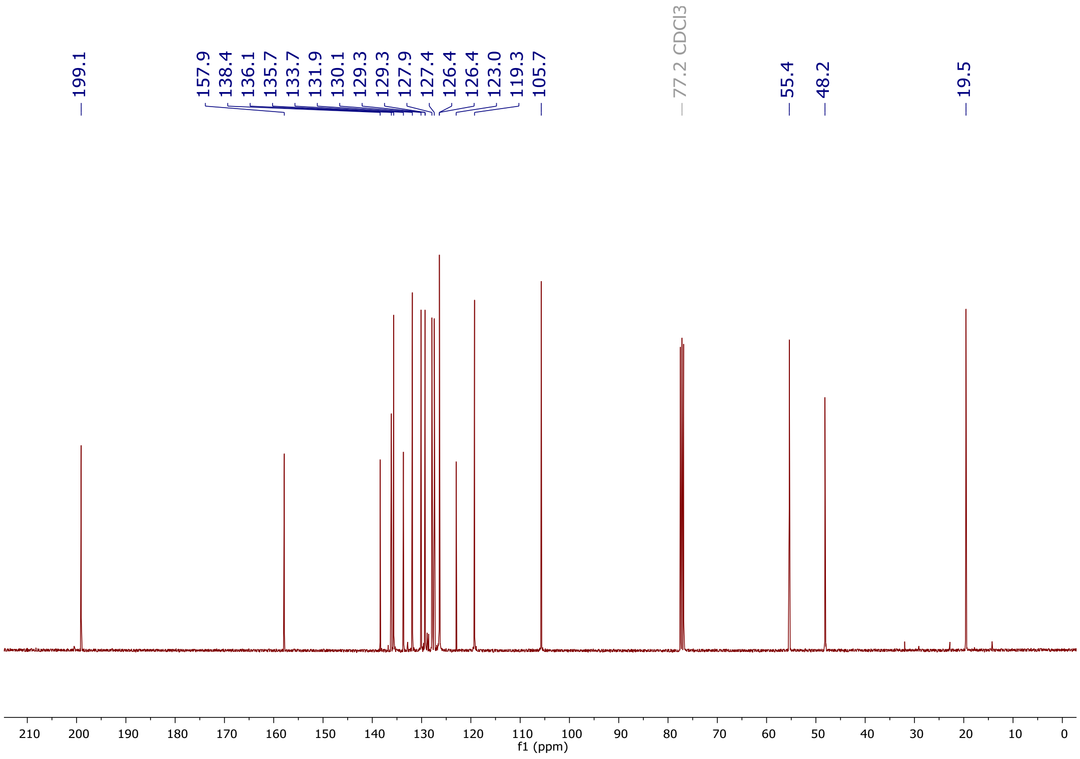

(*S*)-1-(3-(diethoxymethyl)phenyl)-2-(6-methoxynaphthalen-2-yl)propan-1-one (14am)

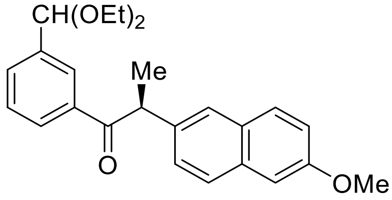

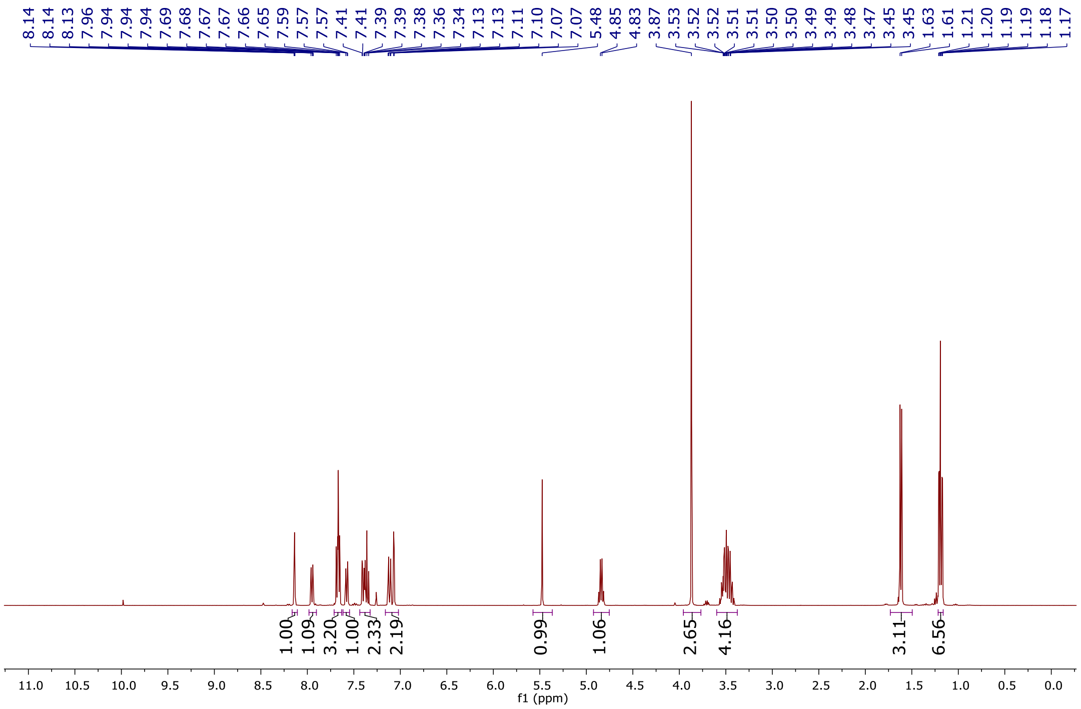

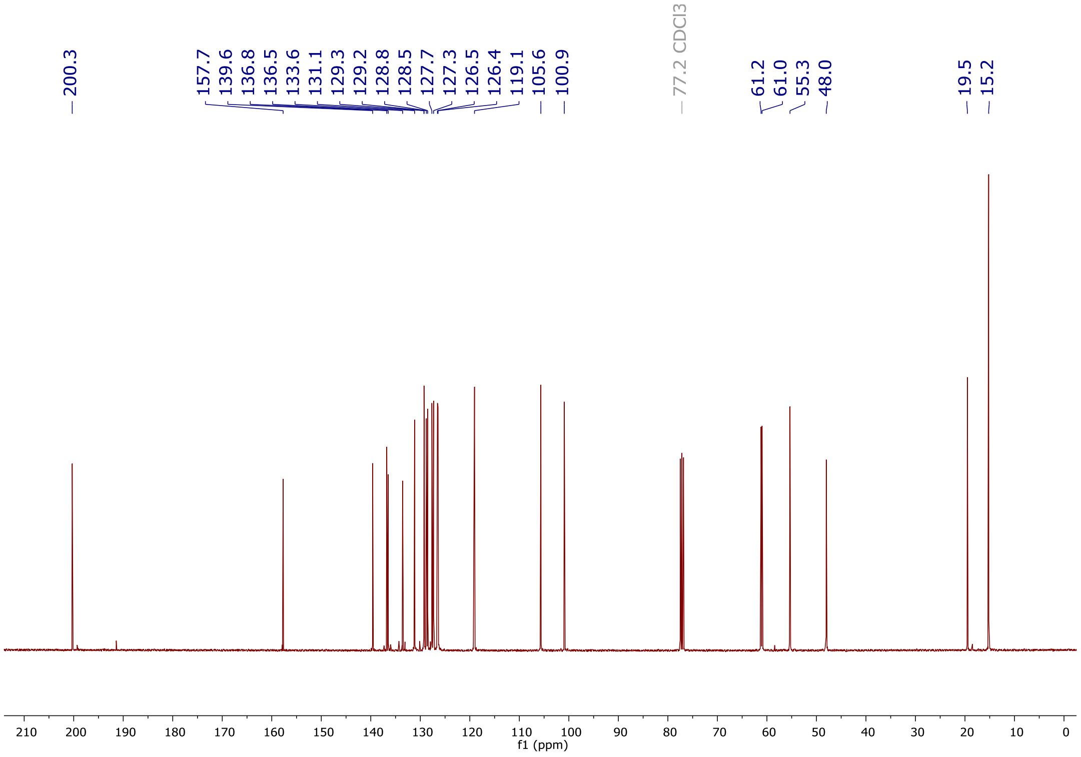

(*S*)-1-(3-Fluorophenyl)-2-(6-methoxynaphthalen-2-yl)propan-1-one (14an)

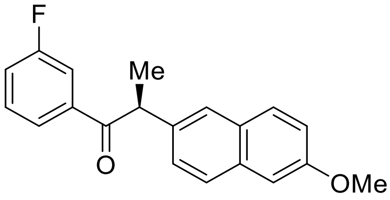

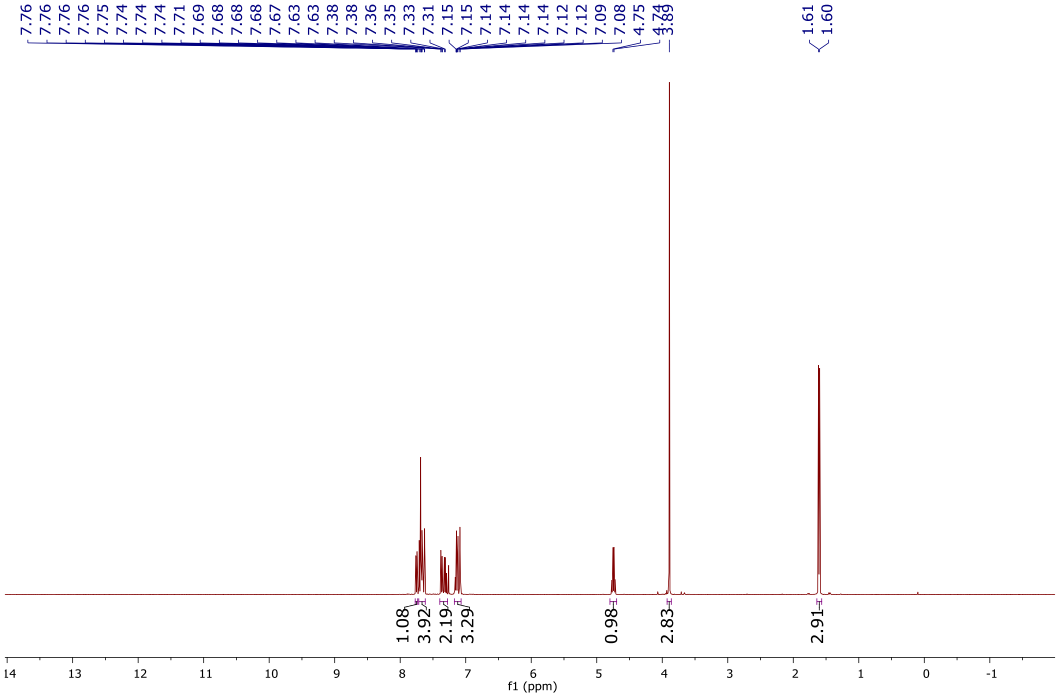

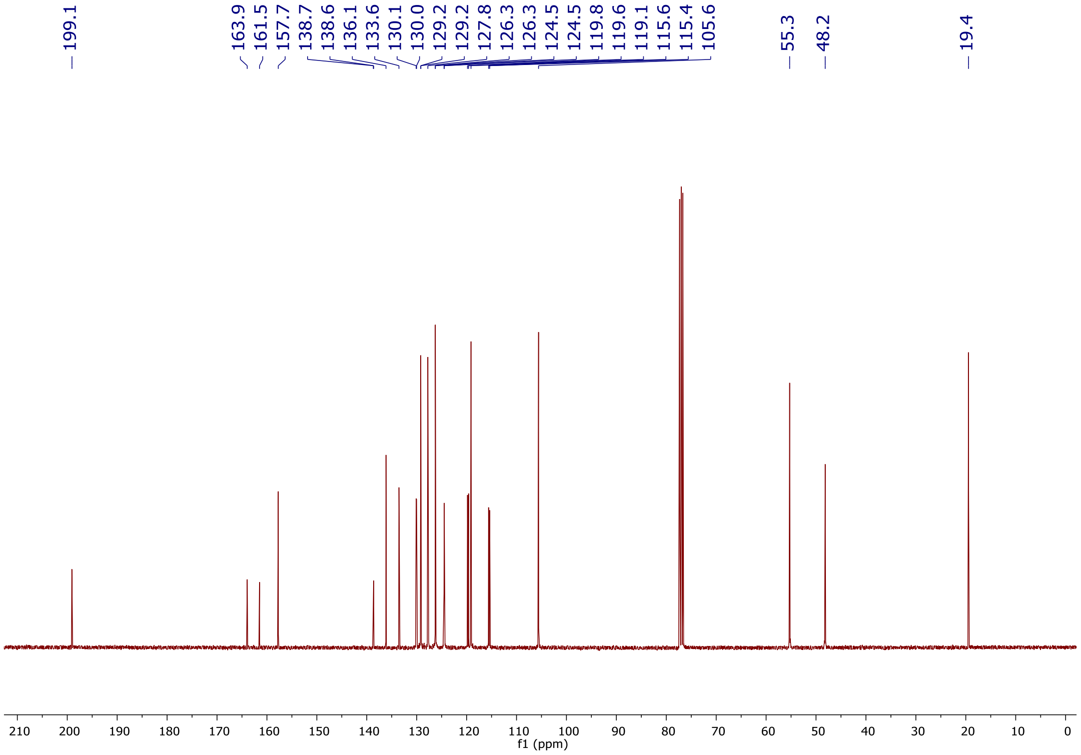

(*S*)-1-(4-Fluorophenyl)-2-(4-(2-methoxypropyl)phenyl)propan-1-one (14bh)

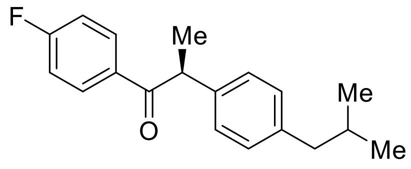

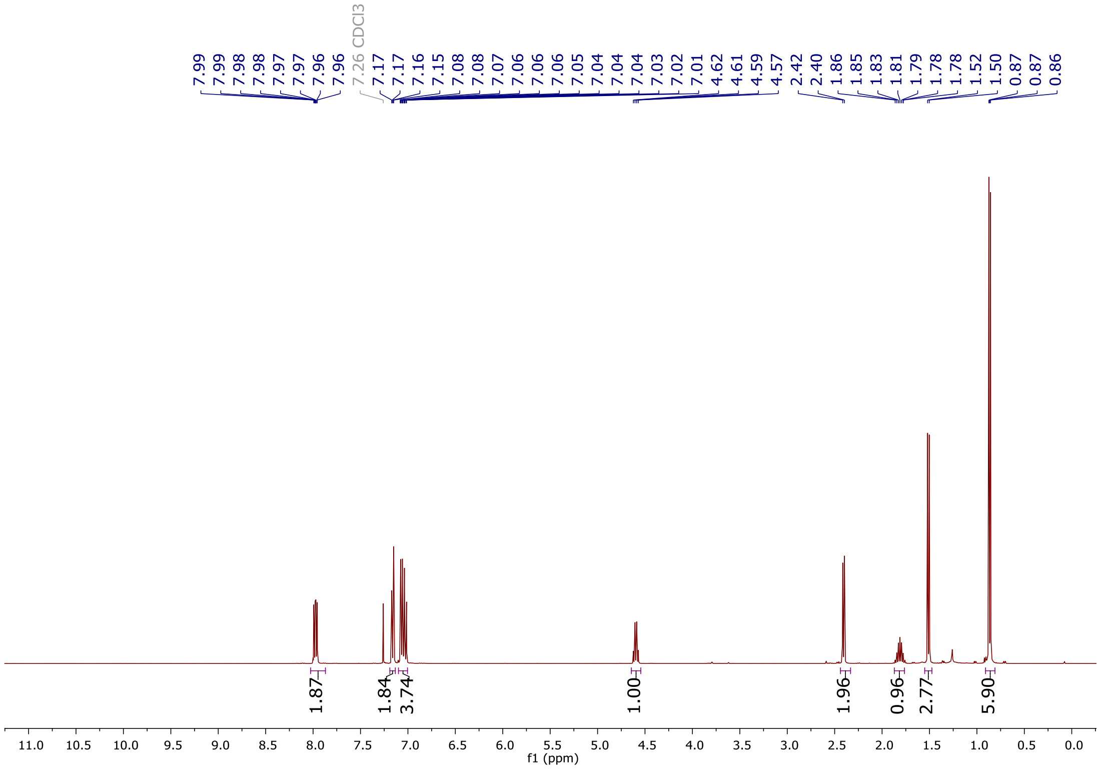

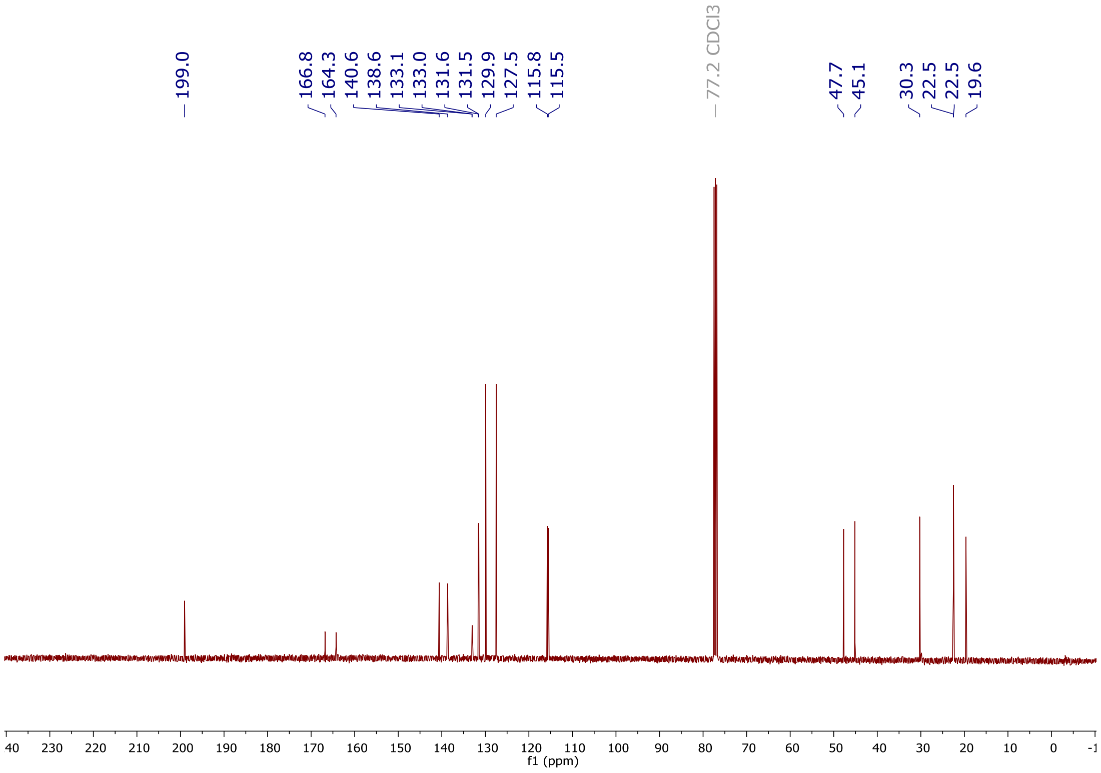

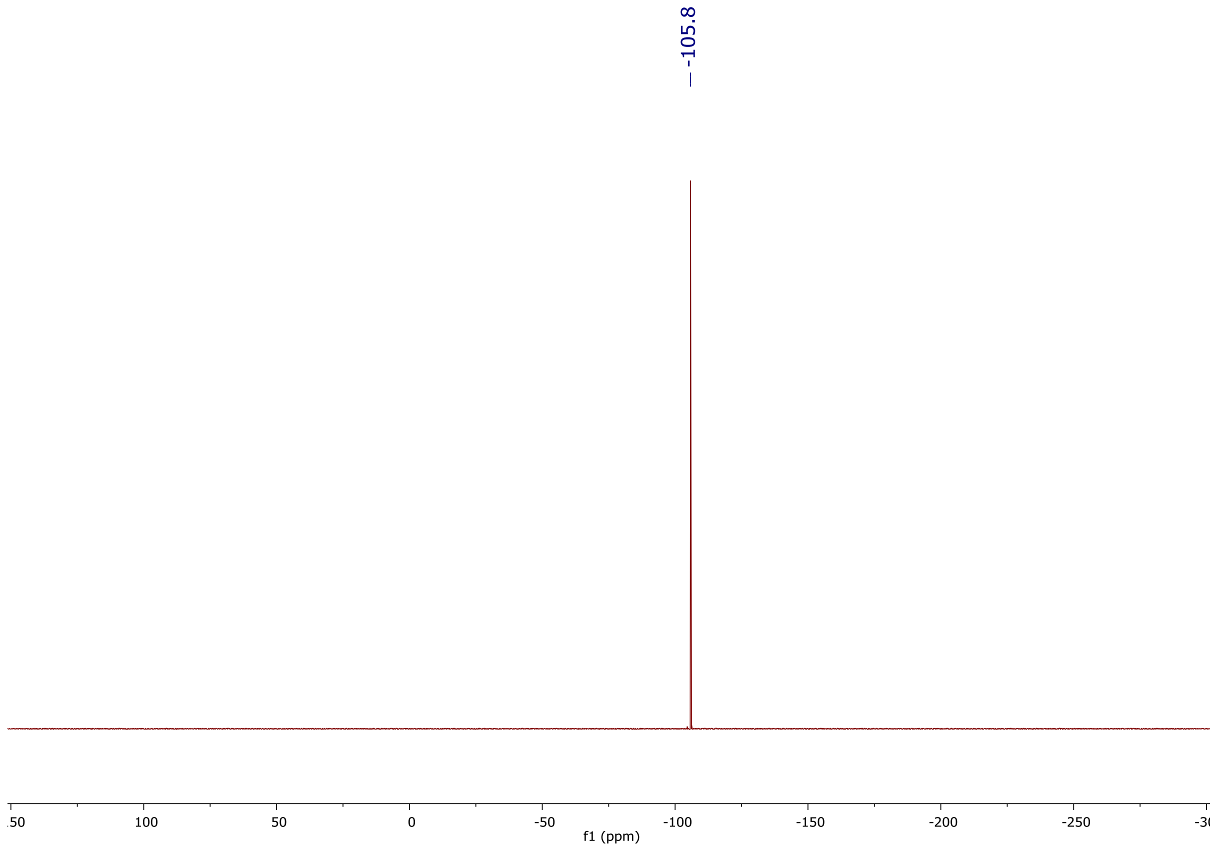
-105.8
150
100
50
0
-50
-100
-150
-200
-250
-300
f1 (ppm)

(*S*)-1-(3,5-Dimethoxyphenyl)-2-(4-(2-methoxypropyl)phenyl)propan-1-one (14bo)

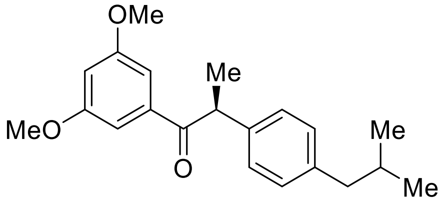

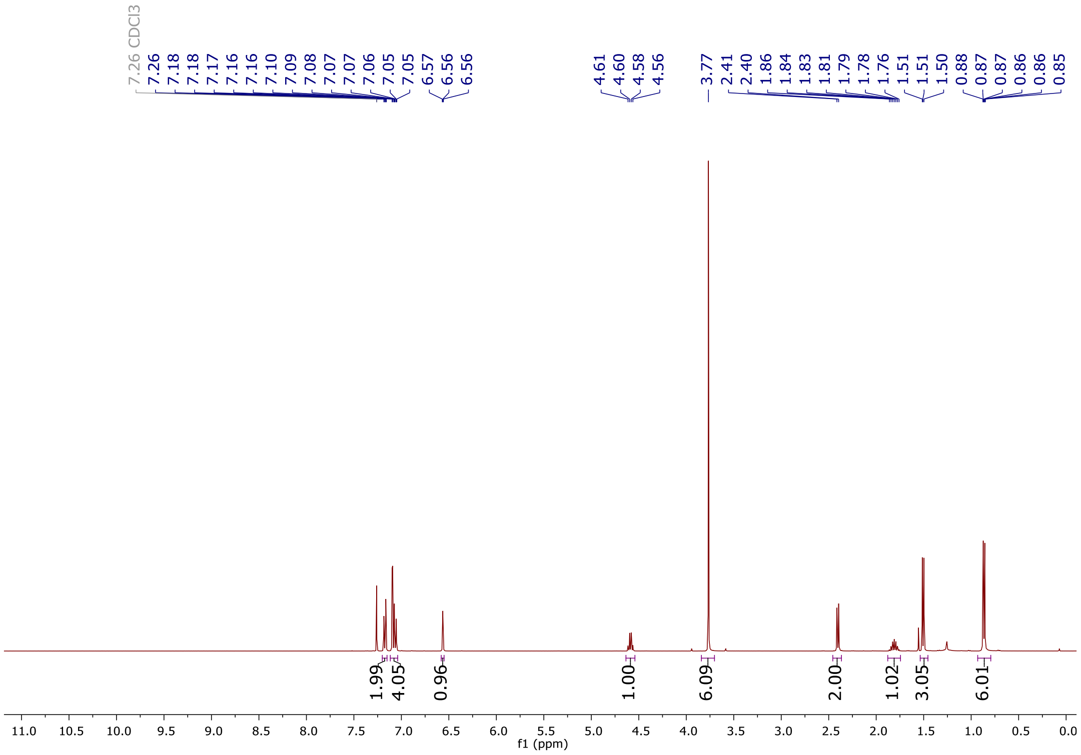

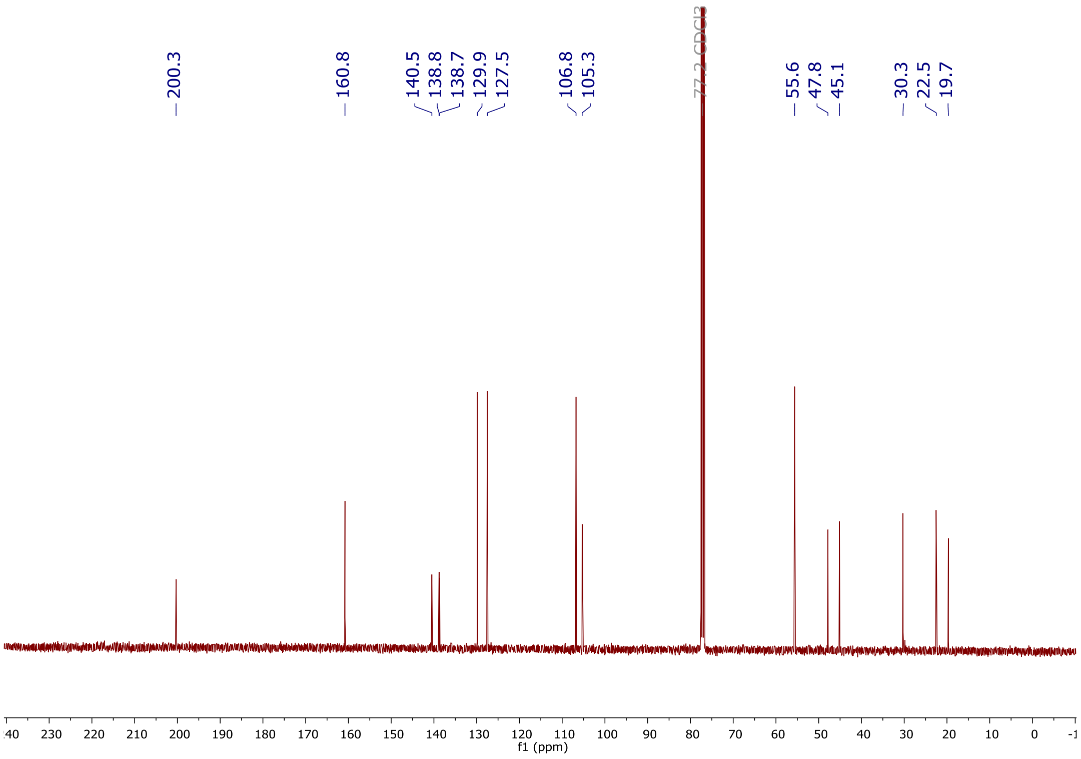

(*S*)-1-(1-butyl-1H-imidazol-2-yl)-2-(4-isobutylphenyl)propan-1-one (14bs)

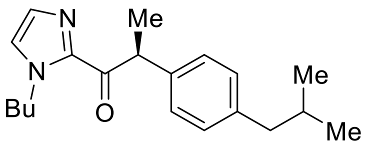

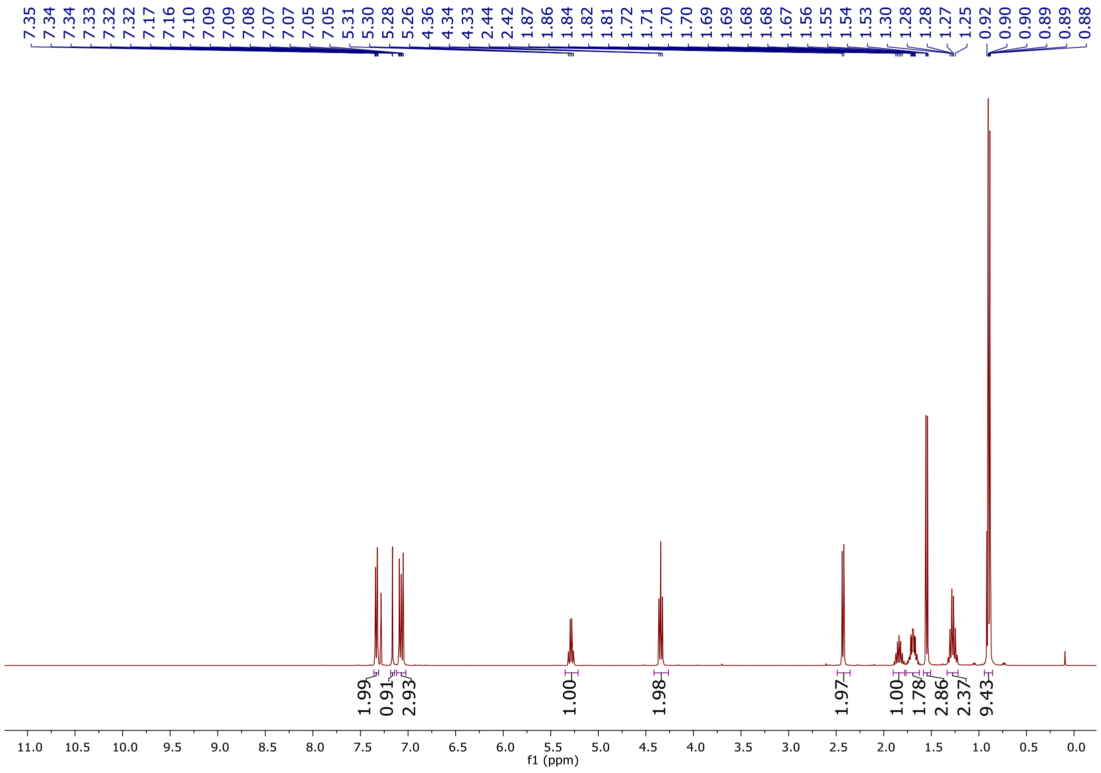

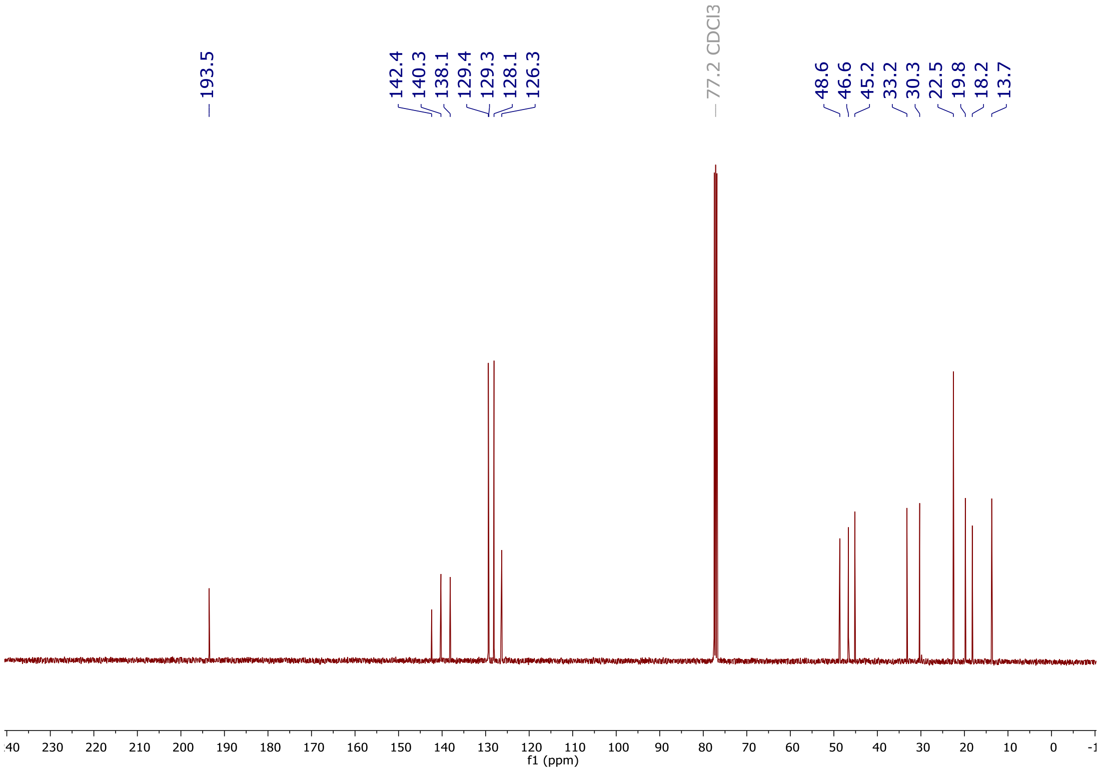

4-Methoxy-*N,N*-dimethylbenzamide (6d)

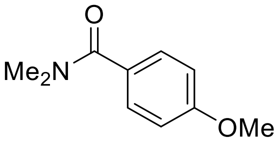

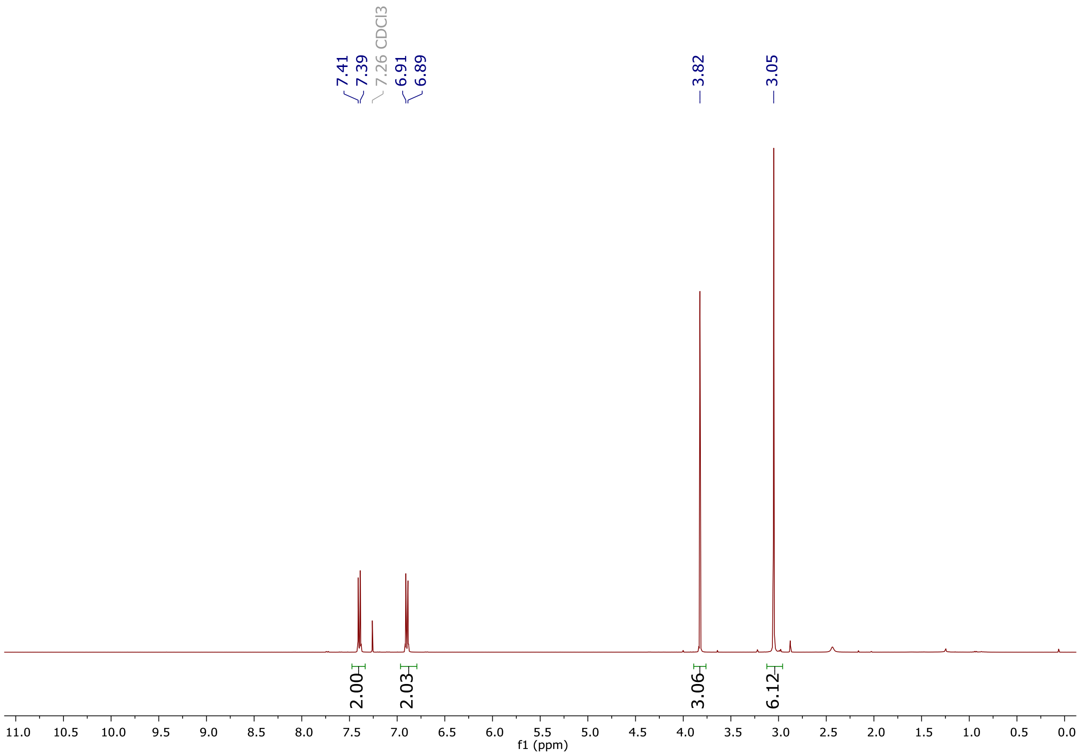

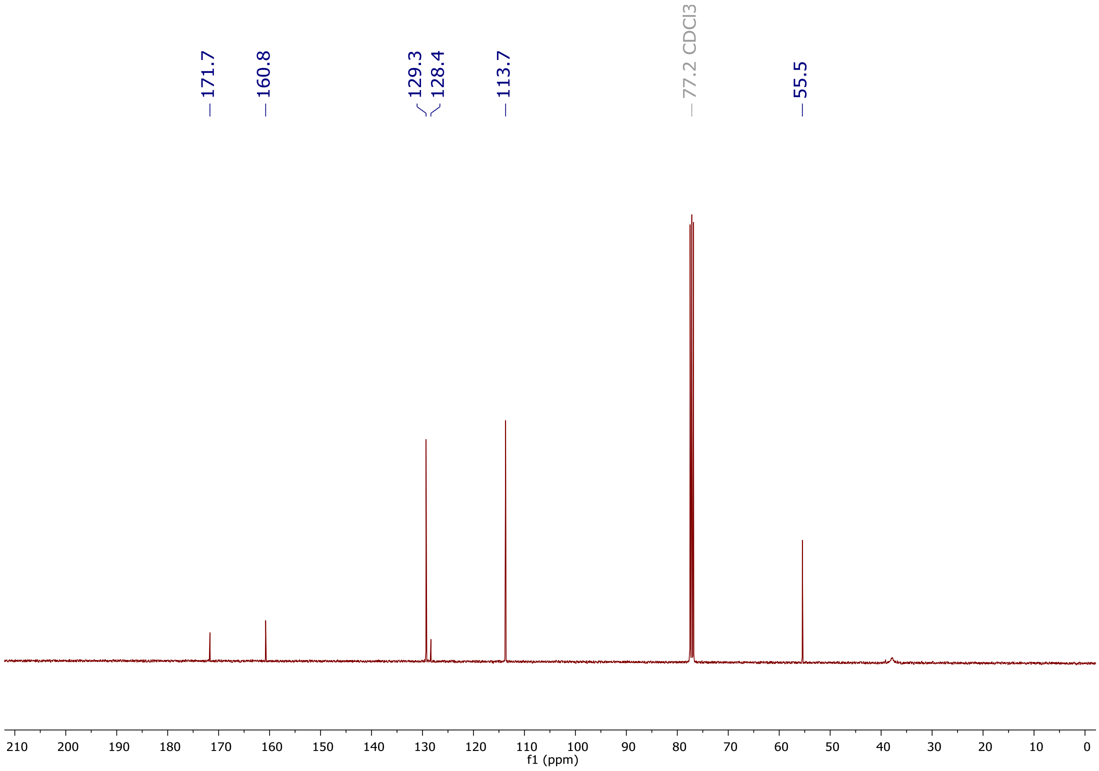

(1-Butyl-1*H*-imidazol-2-yl)(4-methoxyphenyl)methanone (5a)

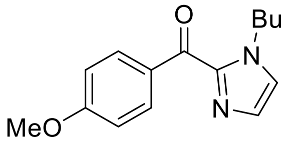

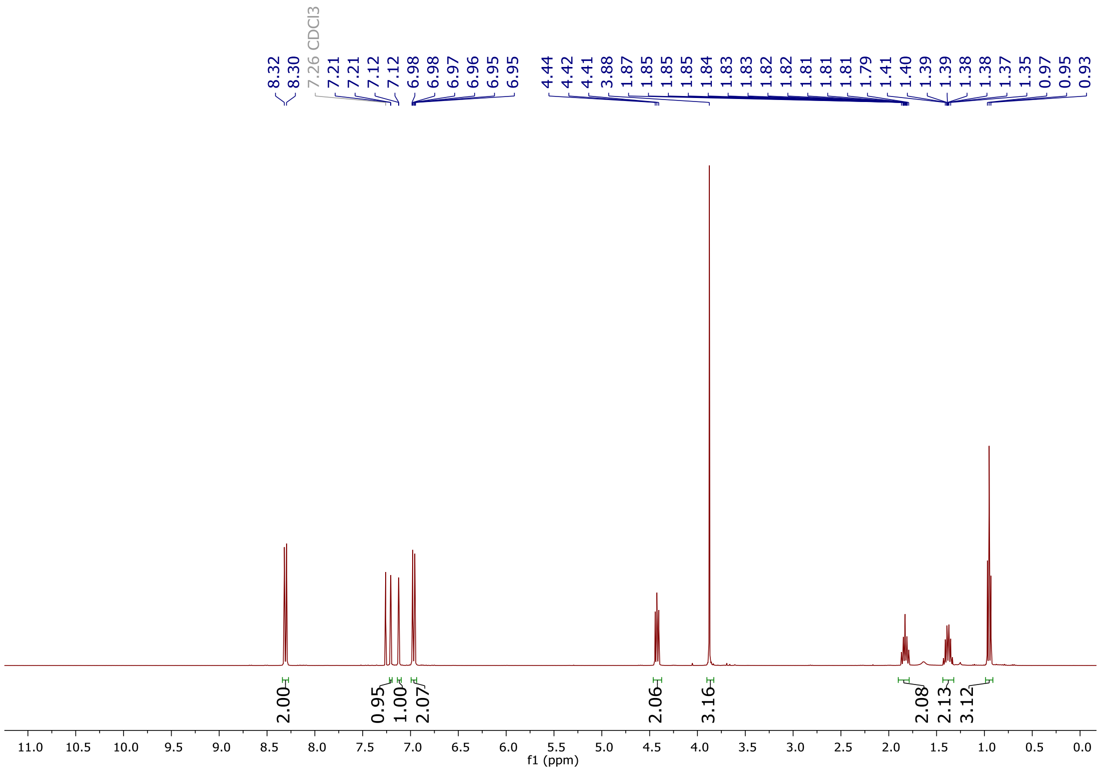

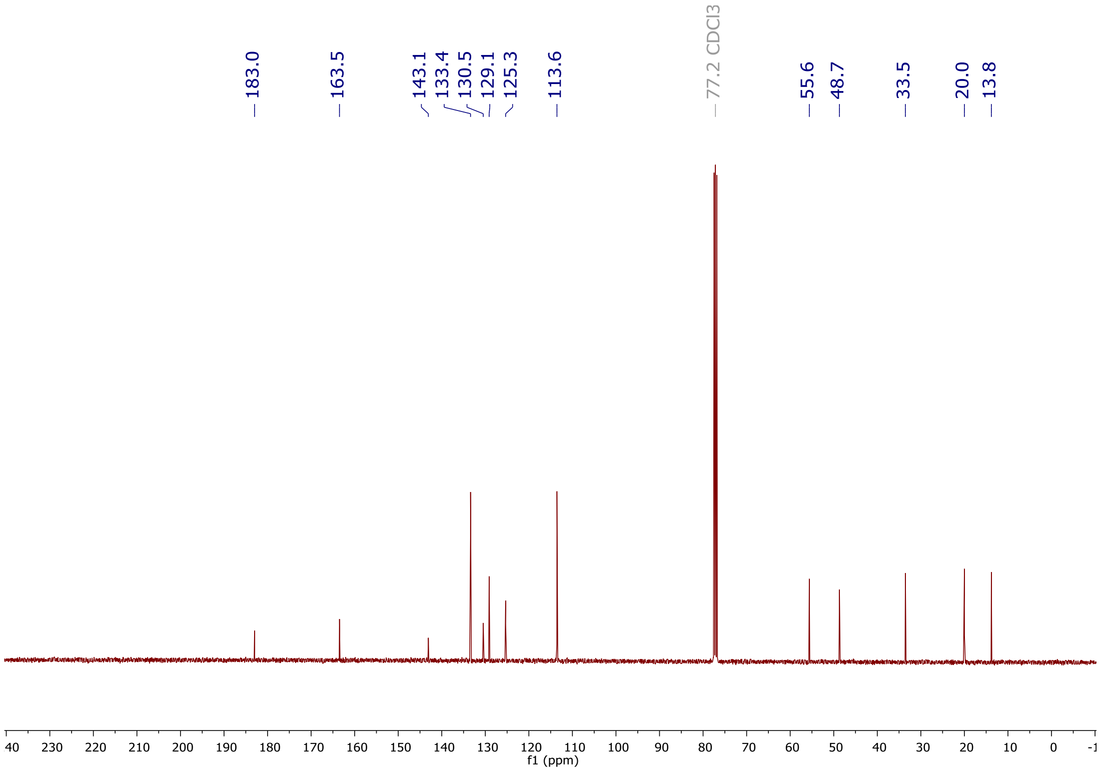

1-(4-Methoxyphenyl)-2-phenylethan-1-one (5b)

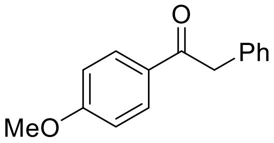

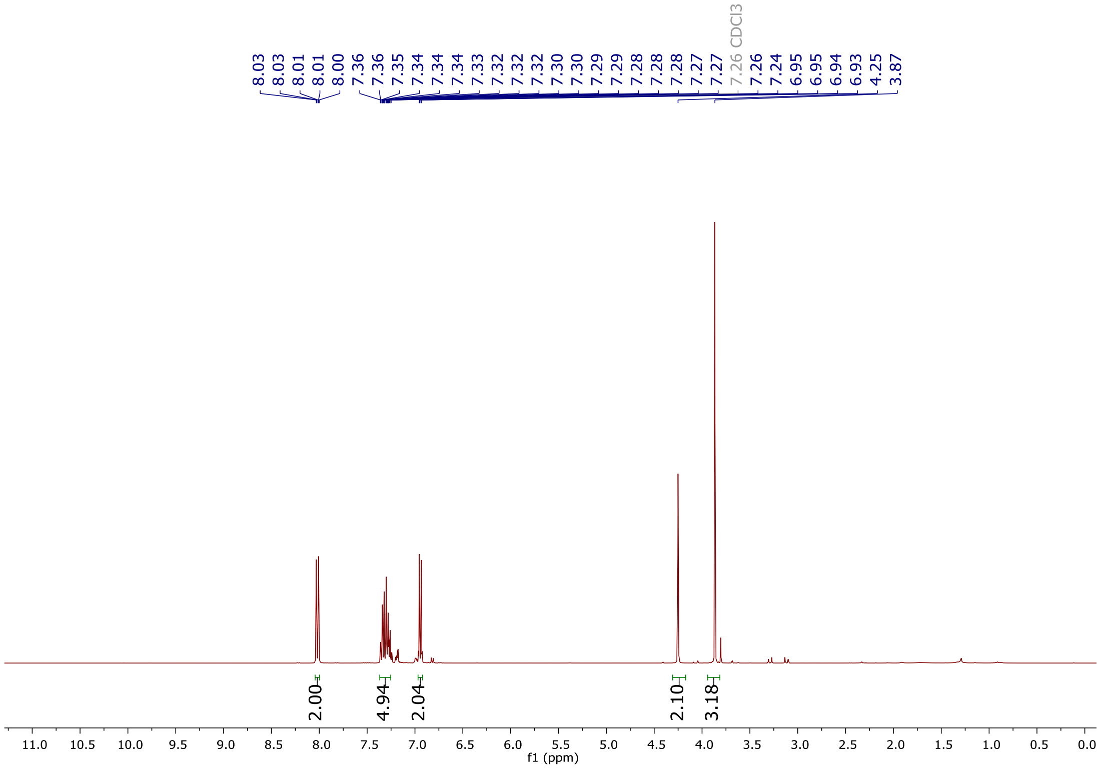

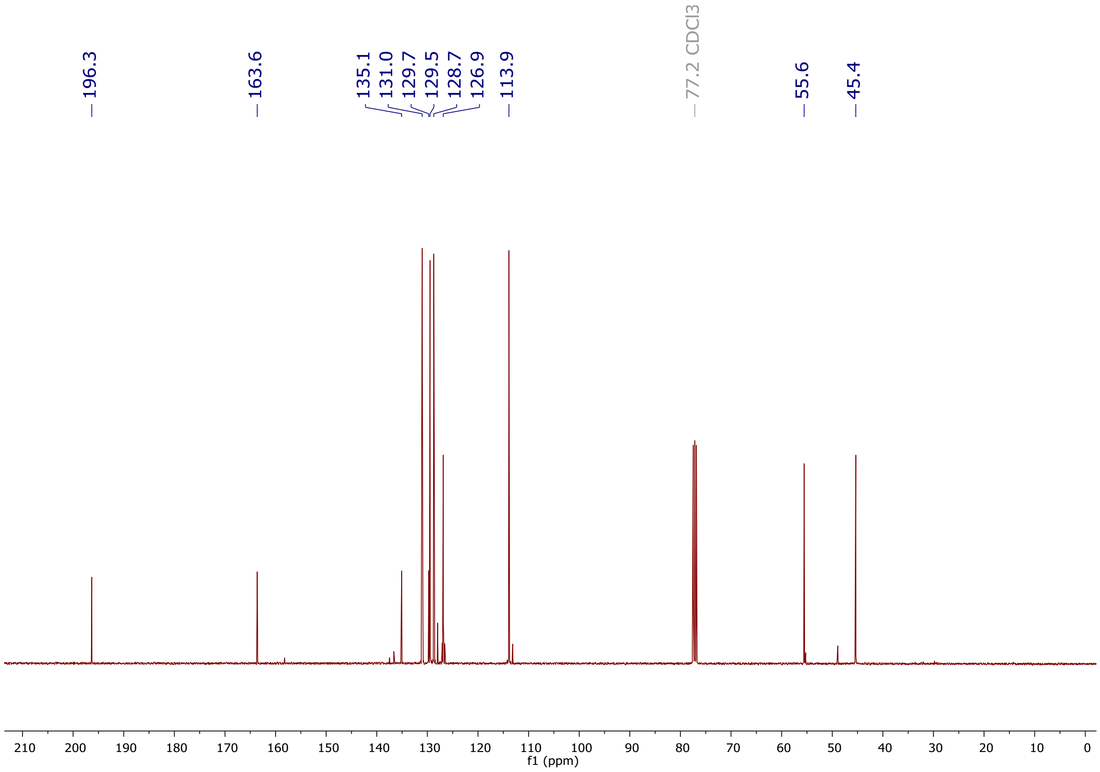

1-(4-Methoxyphenyl)pentan-1-one (5c)

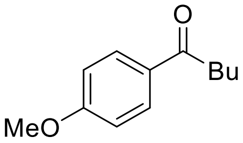

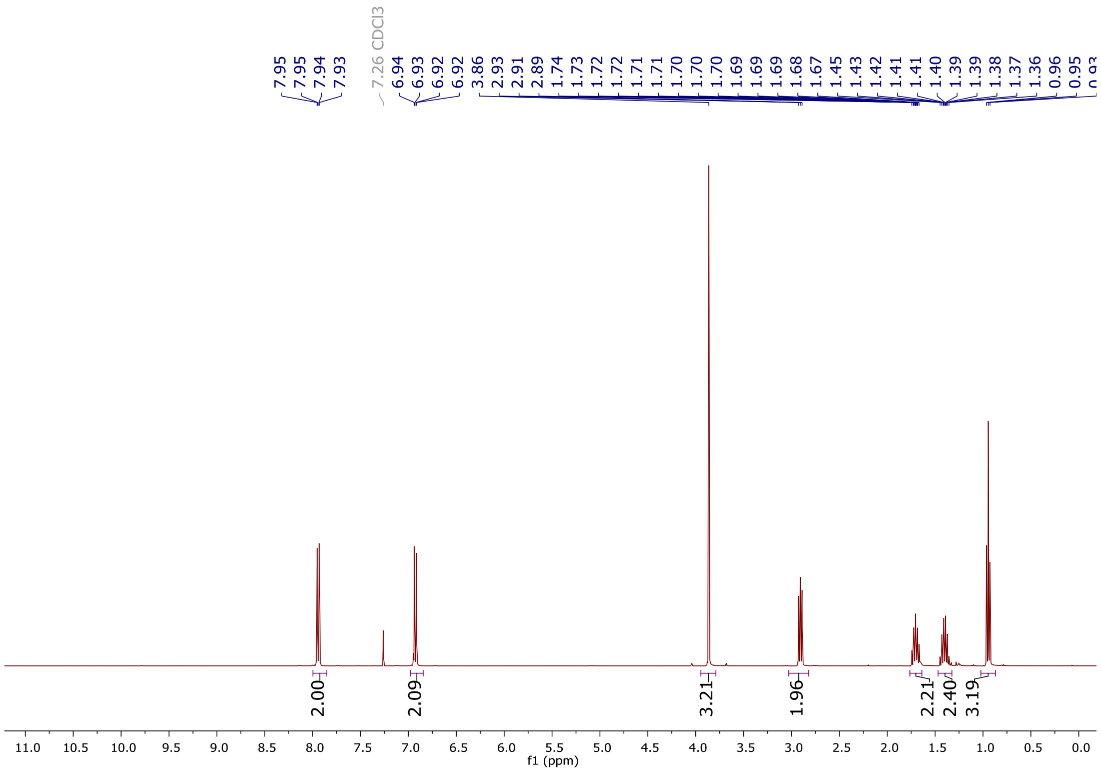

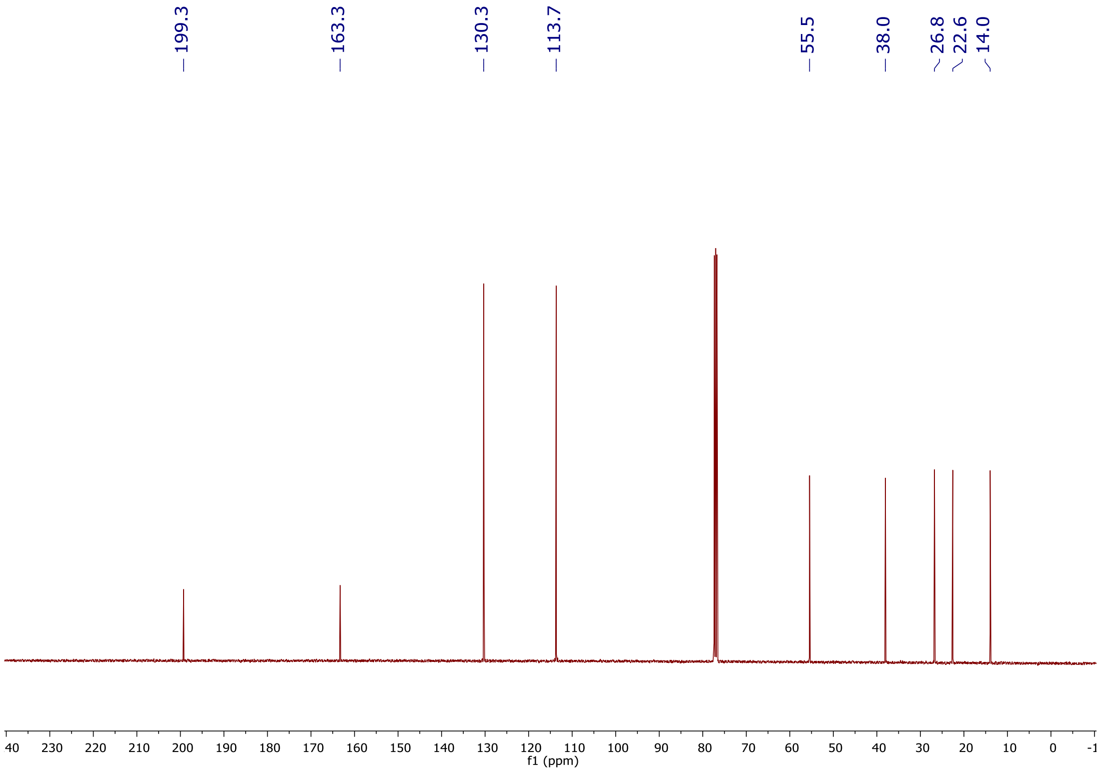

(1-Methyl-1H-benzo[*d*]imidazol-2-yl)(4-(methylthio)phenyl)methanone (5d)

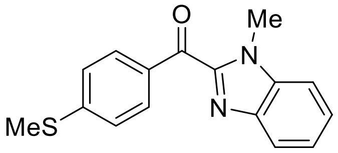

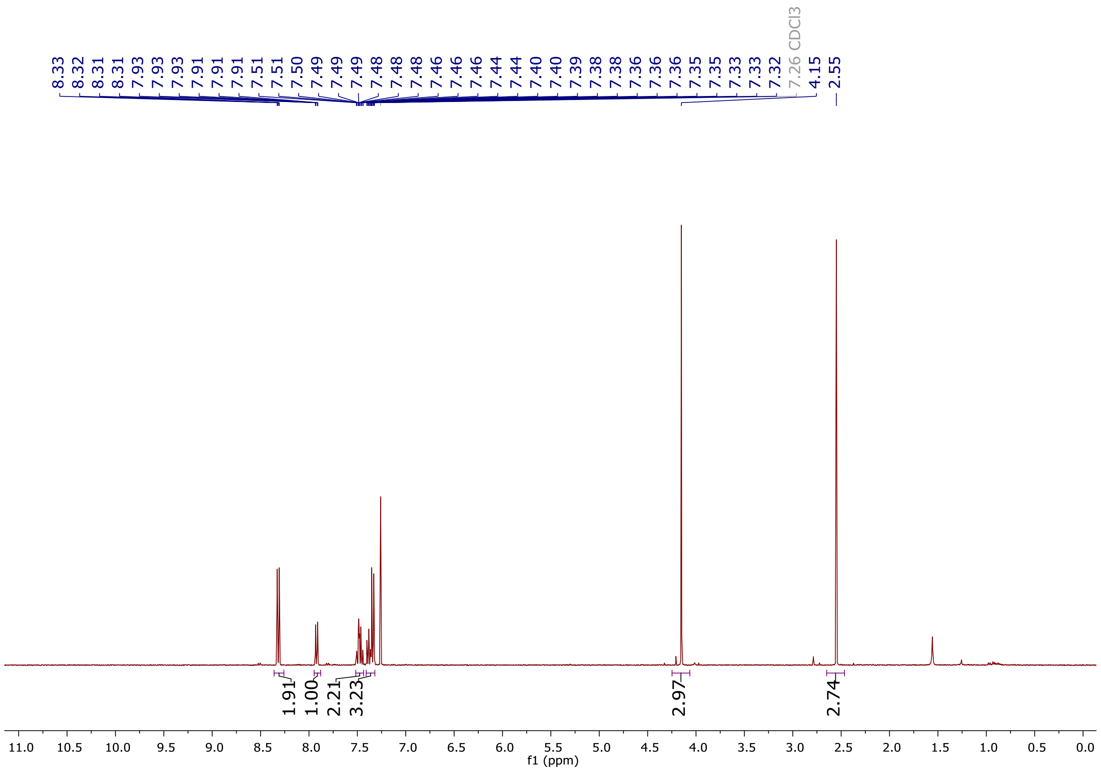

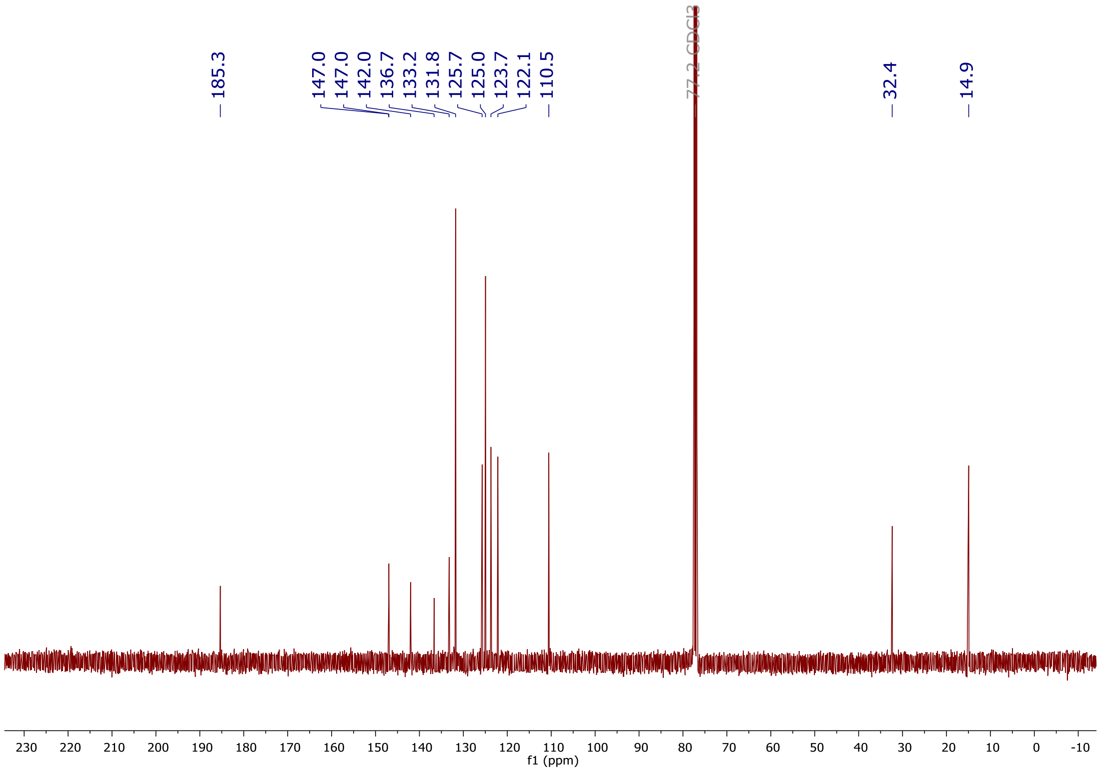

Benzo[*b*]thiophen-2-yl(4-(methylthio)phenyl)methanone (5e)

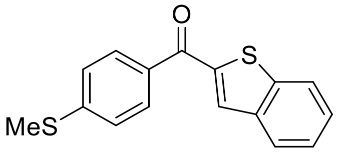

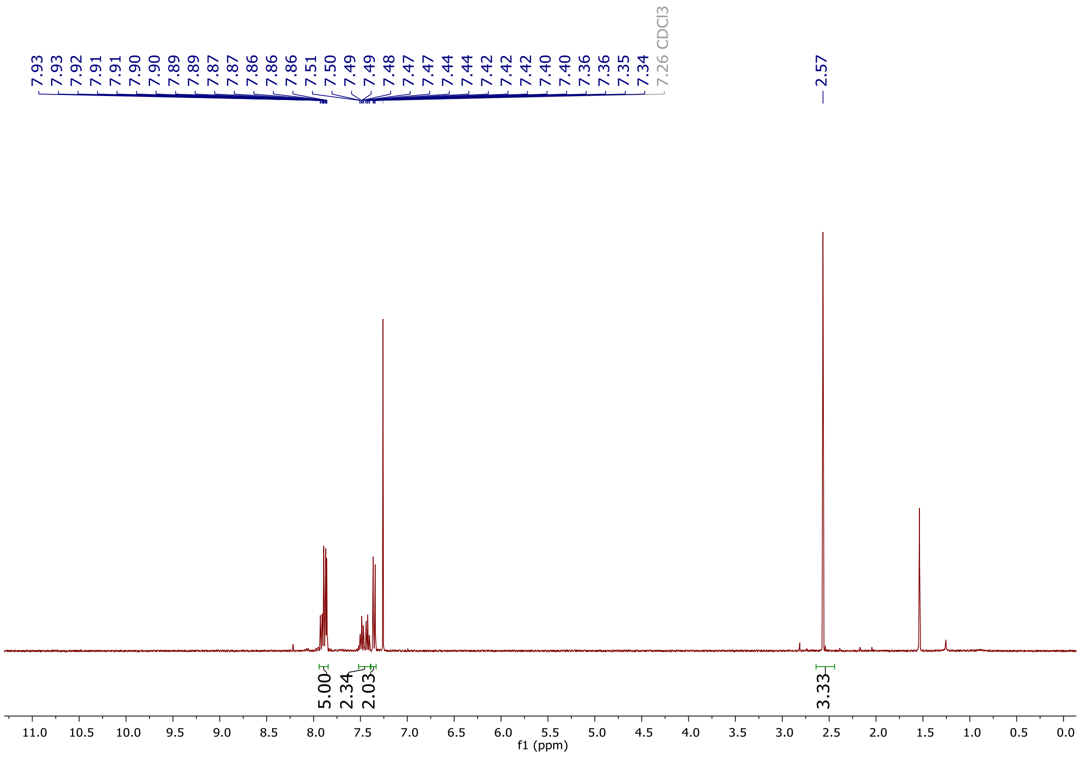

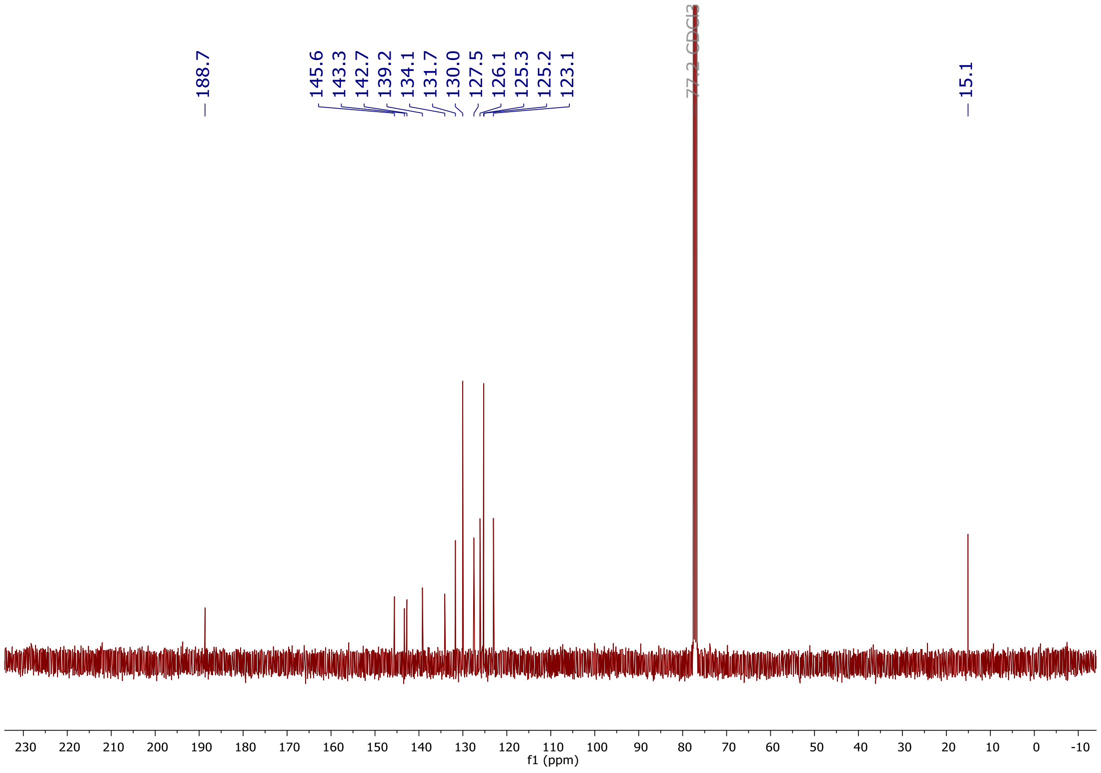

(4-(Methylthio)phenyl)(4-octylthiophen-2-yl)methanone (5f)

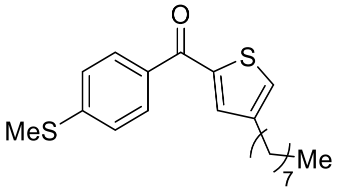

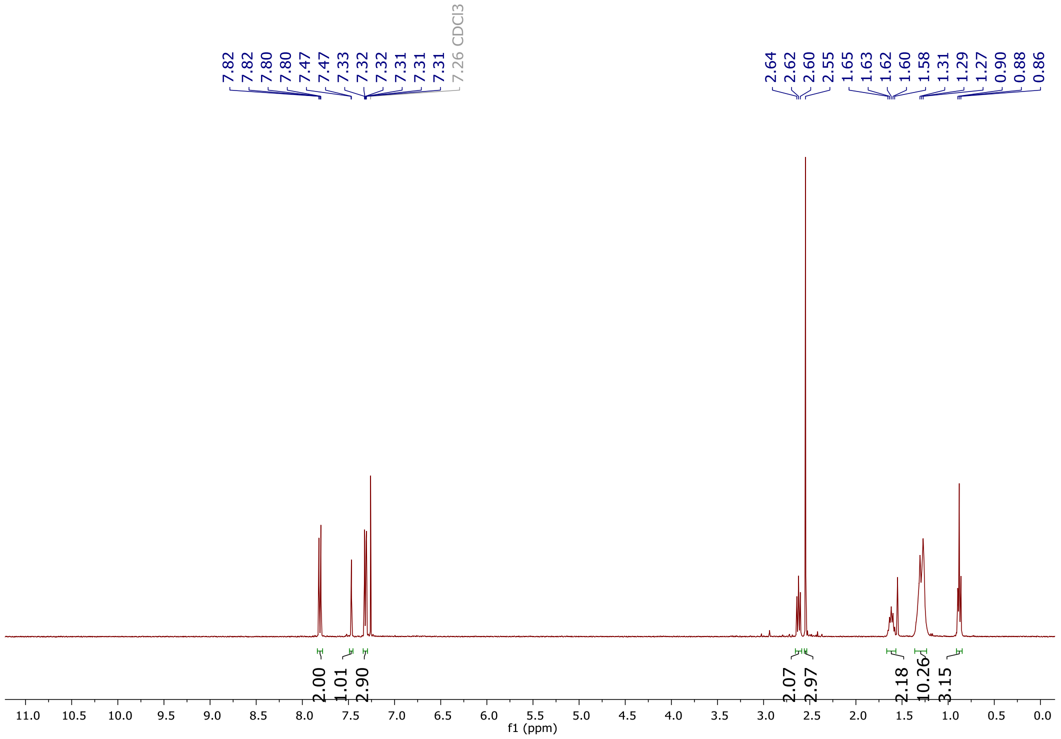

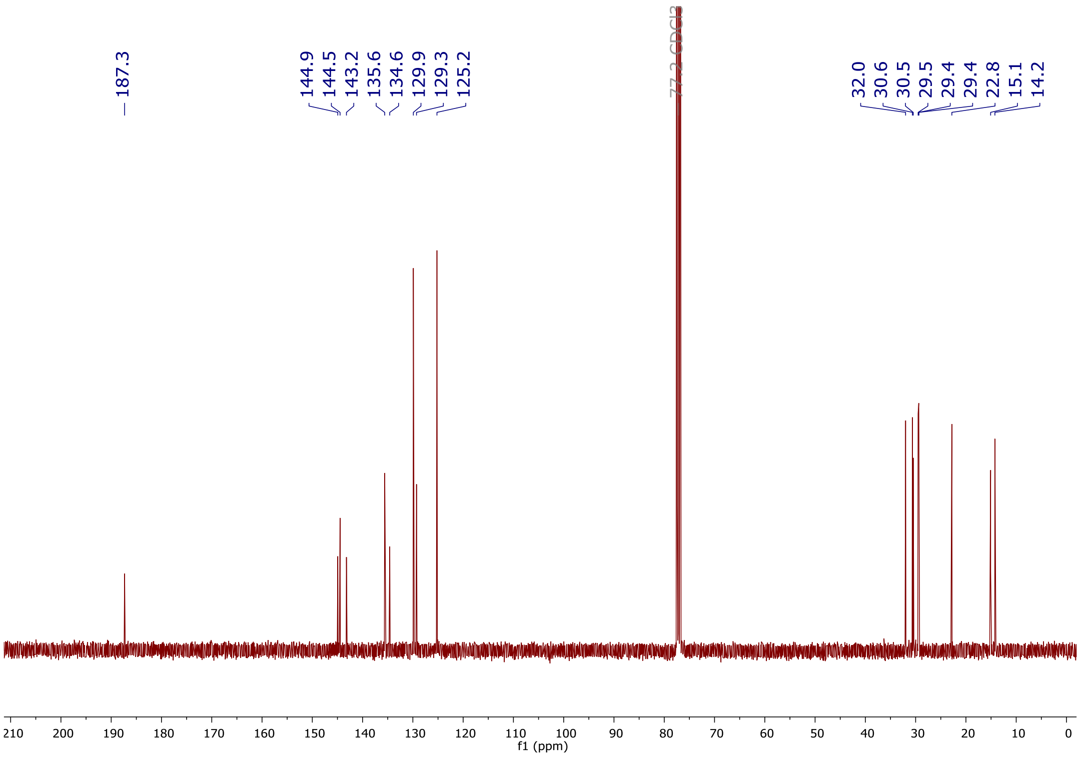

Supplement: Supplementary file 1 — Supporting Information [file CHEM-27-13977-s001.pdf]
